# Silver(I)-Catalyzed Synthesis of Cuneanes from Cubanes and their Investigation as Isosteres

**DOI:** 10.1021/jacs.3c03207

**Published:** 2023-07-21

**Authors:** Elliot Smith, Kieran D. Jones, Luke O’Brien, Stephen P. Argent, Christophe Salome, Quentin Lefebvre, Alain Valery, Mina Böcü, Graham N. Newton, Hon Wai Lam

**Affiliations:** † The GlaxoSmithKline Carbon Neutral Laboratories for Sustainable Chemistry, University of Nottingham, Jubilee Campus, Triumph Road, Nottingham, NG7 2TU, United Kingdom; ‡ School of Chemistry, University of Nottingham, University Park, Nottingham NG7 2RD, United Kingdom; § SpiroChem AG, 4058 Basel, Switzerland

## Abstract

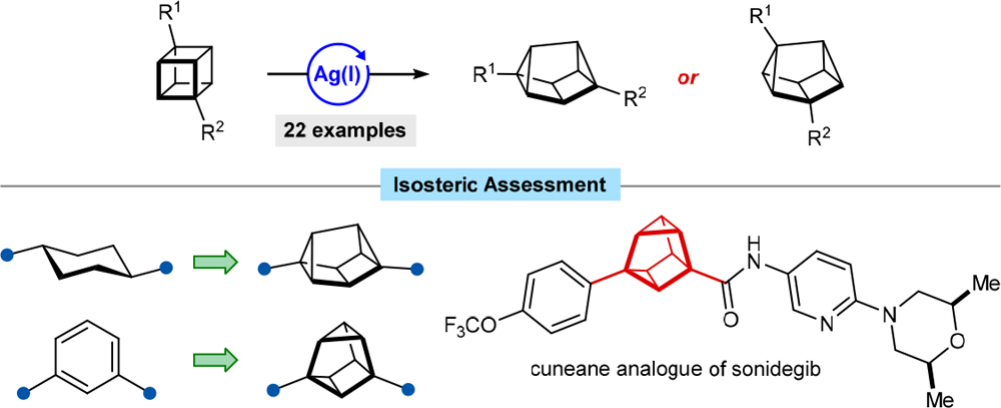

Bridged or caged polycyclic hydrocarbons have rigid structures that project substituents into precise regions of 3D space, making them attractive as linking groups in materials science and as building blocks for medicinal chemistry. The efficient synthesis of new or underexplored classes of such compounds is, therefore, an important objective. Herein, we describe the silver(I)-catalyzed rearrangement of 1,4-disubstituted cubanes to cuneanes, which are strained hydrocarbons that have not received much attention since they were first described in 1970. The synthesis of 2,6-disubstituted or 1,3-disubstituted cuneanes can be achieved with high regioselectivities, with the regioselectivity being dependent on the electronic character of the cubane substituents. A preliminary assessment of cuneanes as scaffolds for medicinal chemistry suggests cuneanes could serve as isosteric replacements of *trans*-1,4-disubstituted cyclohexanes and 1,3-disubstituted benzenes. An analogue of the anticancer drug sonidegib was synthesized, in which the 1,2,3-trisubstituted benzene was replaced with a 1,3-disubstituted cuneane.

## Introduction

**Figure 1:**
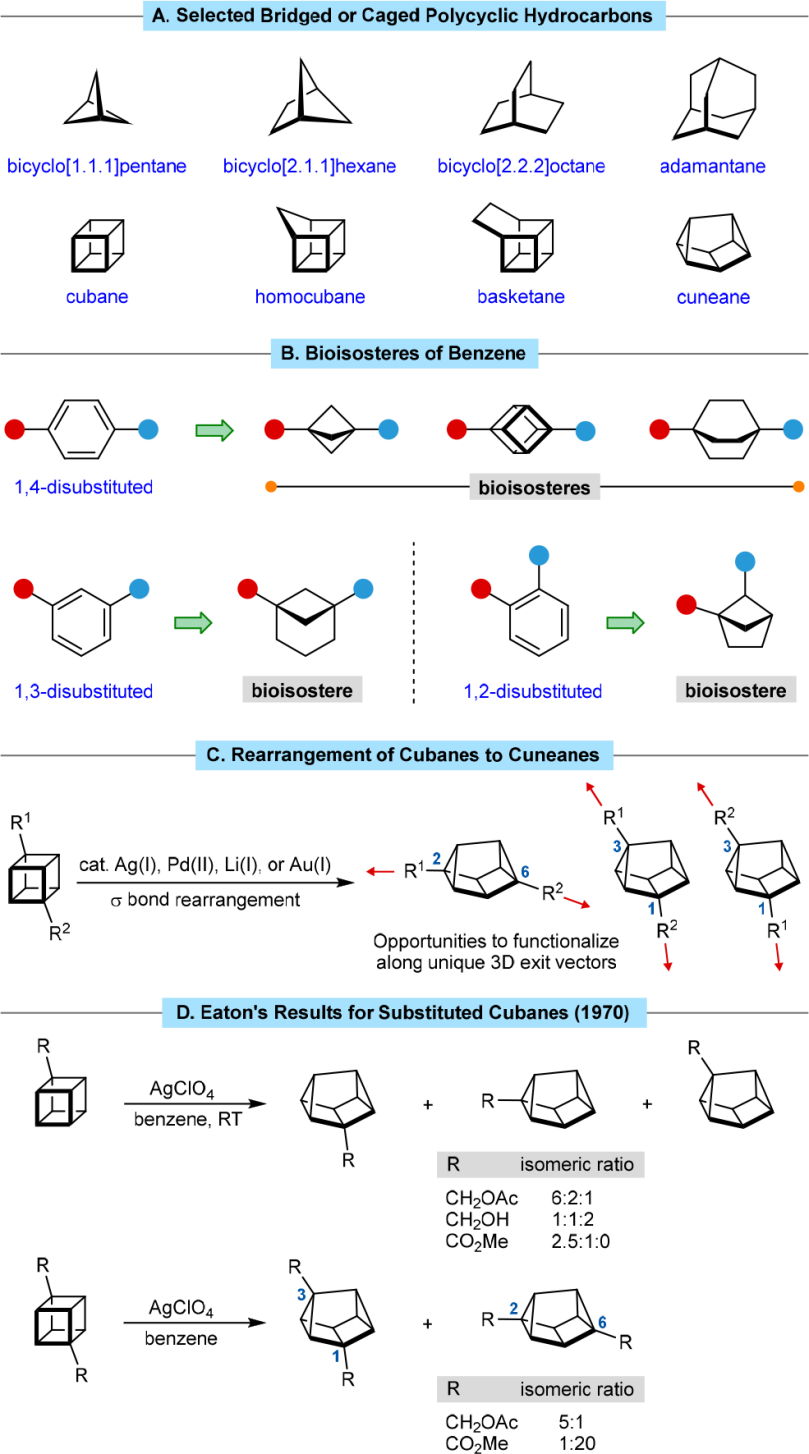
Bridged or caged polycyclic hydrocarbons and the rearrangement of cubanes to cuneanes.

Bridged or caged polycyclic hydrocarbons^1^ are important compounds (see Figure 1A for examples) because: (a) they have topologically interesting structures; (b) their often high ring strain can lead to unique chemical behavior and reactivity; (c) overcoming challenges in their synthesis results in advances in synthetic methodology; and (d) their rigid structures project substituents into precise regions of 3D space, which gives them potential applications as linking groups in materials science and supramolecular chemistry,^1c^ and as scaffolds in medicinal chemistry.^1b,1c^ Regarding the latter point, certain polycyclic hydrocarbons are of particular interest as *sp*^3^-rich,^2^ conformationally restricted^3^ bioisosteres of benzene (Figure 1B),^4−8^ which are increasingly used to generate lead compounds with greater three-dimensionality, in the bid to improve clinical success rates.^2^ Therefore, there is significant interest in developing new synthetic methods to prepare and functionalize bridged or caged polycyclic hydrocarbons and investigating new or underexplored classes of these compounds to survey novel chemical space.

Cuneanes are interesting caged hydrocarbons that have been largely overlooked.^9−11^ First described by Cassar, Halpern, and Eaton in 1970,^9a^ cuneanes are prepared by the σ bond rearrangement of cubanes, catalyzed by Ag(I),^9a,9g,9h,10,11^ Pd(II),^9a,9h^ Li(I),^9b^ or Au(I)^9h^ (Figure 1C). The aqueous media-induced rearrangement of cubane-1,4-dicarboxylic acid to cuneane-2,6-dicarboxylic acid has also been reported.^9f^

The pioneering work of Eaton and co-workers described the rearrangement of cubane itself, as well as two monosubstituted and two symmetrically 1,4-disubstituted cubanes (Figure 1D).^9a^ These results showed that monosubstituted cubanes give mixtures of the three possible cuneane regioisomers, while 1,4-disubstituted cubanes give only two cuneane regioisomers out of the possible ten. Since this first report,^9a^ there had been few studies describing the synthesis of cuneanes,^9b−9g^ and these did not report any notable advances in the substrate scope. During the course of the investigations described herein, Matsubara and co-workers described the Ag(I)-catalyzed rearrangement of seven 1,4-disubstituted cubanes, two of which were nonsymmetrically disubstituted, to give racemic 2,6-disubstituted cuneanes.^9h^ This study also reported the asymmetric Pd(II)- and/or Ag(I)-catalyzed rearrangement of four symmetrically substituted cubane 1,4-diesters to give 2,6-disubstituted cuneanes with moderate enantioselectivities.

In view of the strong interest in *sp*^3^-rich, conformationally restricted scaffolds for developing new functional molecules,^1−8^ greater exploration of the synthesis of cuneanes is valuable and would add to the growing body of knowledge of caged hydrocarbon chemistry. In particular, cuneanes offer the possibility to functionalize along unique 3D exit vectors^12^ (Figure 1C), making them potentially useful building blocks in medicinal chemistry. Furthermore, the availability of procedures to prepare cubanes on a large scale,^13^ as well as recent developments that enable access to diverse cubanes,^6b,14^ should enable greater investigation of the chemistry of cuneanes.

To facilitate potential applications of cuneanes, additional studies in two areas of cuneane synthesis were warranted. First, a greater understanding of the effect of the nature of the cubane substituents on the rate and regiochemical outcome of the rearrangement would be beneficial as this had been studied for only a small range of substrates.^9a,9h^ In addition, greater access to 1,3-disubstituted cuneanes was required as there had been only a single reported example of a 1,3-isomer being obtained as the major product (Figure 1D).^9a^ Herein, we describe our efforts to address these areas and report the synthesis of a range of 2,6-disubstituted and 1,3-disubstituted cuneanes by the silver(I)-catalyzed rearrangement of 1,4-disubstituted cubanes. The regioselectivity of the rearrangement is strongly dependent on the electronic character of the cubane substituents. Potential applications of cuneanes as isosteres in medicinal chemistry are suggested and the synthesis of a cuneane analogue of the anticancer drug sonidegib was achieved.^10^

## Results and Discussion

### Rearrangement of Cubanes to Cuneanes

This investigation began with a survey of reaction conditions for the rearrangement of cubane 1,4-dimethyl ester **1a** to 2,6-disubstituted cuneane **2a** (Table 1). Consistent with results reported in the literature,^9a,9d,9g,10,11^ we found silver salts to be effective in promoting this reaction. In most cases, none of the alternative 1,3-disubstituted cuneane **3a** was detected in these reactions. Although heating **1a** and AgOAc (100 mol %) in toluene at 100 °C for 16 h in a sealed vessel gave minimal (<5%) conversion (entry 1), the use of AgNO_3_ successfully gave **2a**, though in a modest 22% NMR yield (entry 2). Increasing the polarity of the solvent was beneficial, with reactions conducted in 1,4-dioxane and *t*-BuOH giving improved NMR yields of 71% and 76%, respectively (entries 3 and 4). In *t*-BuOH, decreasing the temperature to 80 and 60 °C gave lower yields (entries 5 and 6). AgClO_4_ was also effective^9a^ (entry 7) but inferior to AgNO_3_. The catalyst loading of AgNO_3_ can be decreased to 25 mol %, as shown by a reaction that gave **2a** in 93% isolated yield after 20 h (entry 8). *t*-Amyl alcohol (*t*-AmOH) was used as the solvent for this reaction because of its higher boiling point compared with *t*-BuOH. A further reduction in catalyst loading to 10 mol % led to a lower yield of **2a** (entry 9). AgNTf_2_, which contains a very weakly coordinating anion, allowed the use of a less polar solvent (CH_2_Cl_2_) and a lower temperature of 50 °C, but this reaction gave a mixture of **2a** and **3a** with poor regioselectivity (entry 10). The use of Pd(OAc)_2_ (5 mol %) in *t*-BuOH at 100 °C was also successful, but the NMR yield of **2a** was 23% (entry 11).

**Table 1:**
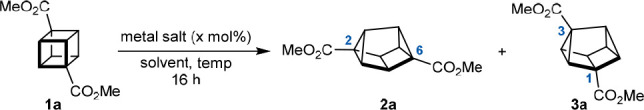
Reaction Optimization^a^

entry	metal salt	x	solvent	temp (°C)	**2a**:**3a**^b^	yield (%)^c^
1	AgOAc	100	toluene	100	>19:1	<5
2	AgNO_3_	100	toluene	100	>19:1	22
3	AgNO_3_	100	1,4-dioxane	100	>19:1	71
4	AgNO_3_	100	*t*-BuOH	100	>19:1	76
5	AgNO_3_	100	*t*-BuOH	80	>19:1	41
6	AgNO_3_	100	*t*-BuOH	60	>19:1	10
7	AgClO_4_	100	*t*-BuOH	100	>19:1	62
**8**	**AgNO**_**3**_	**25**	***t******-*AmOH**	**100**	**>19:1**	**93**^d^
9	AgNO_3_	10	*t*-AmOH	100	>19:1	59
10	AgNTf_2_	10	CH_2_Cl_2_	50	3.5:1	91^e^
11	Pd(OAc)_2_	5	*t*-BuOH	100	>19:1	23

a Reactions were conducted with 0.10 mmol of **1a** in solvent (1.0 mL) in a sealed reaction vial. *t*-AmOH = *tert*-amyl alcohol.

b Determined by ^1^H NMR analysis of the crude reaction mixtures.

c Determined by ^1^H NMR analysis using 1,3-benzodioxole as an internal standard.

d The reaction time was 20 h. The quoted yield is of isolated material for a reaction conducted using 0.20 mmol of **1a** in *t*-AmOH (2.0 mL).

e The quoted yield is of isolated material consisting of a 5:1 mixture of **2a** and **3a**, from a reaction conducted using 0.91 mmol of **1a** in CH_2_Cl_2_ (10.0 mL).

**Scheme 1:**
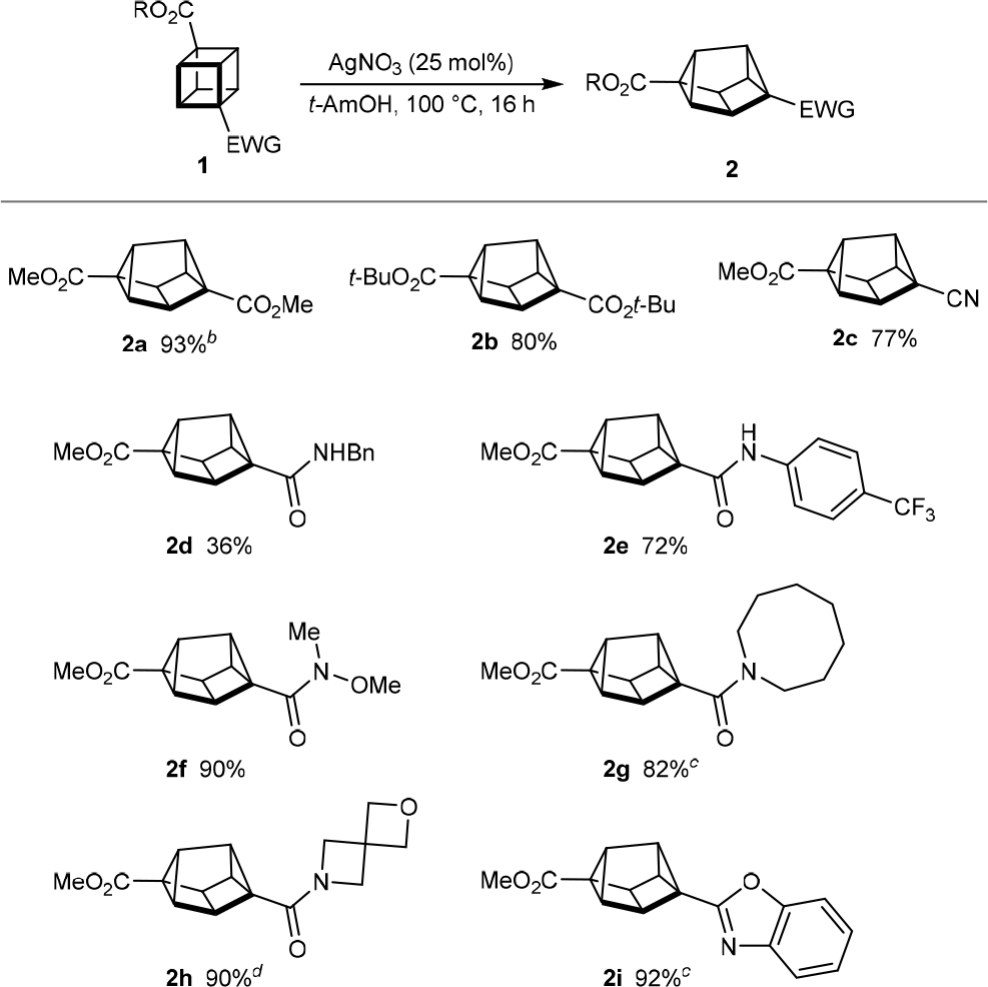
Synthesis of 2,6-Disubstituted Cuneanes Reactions were conducted with 0.20 mmol of **1** in *t*-AmOH (2.0 mL). Yields are of isolated products. The reaction time was 20 h. An attempted reaction using AgNTf_2_ (10 mol %) in CH_2_Cl_2_ at 50 °C for 16 h led to return of unchanged starting material (<5% conversion). Conducted using 40 mol % of AgNO_3_.

Using the conditions of Table 1, entry 8, the scope of the silver(I)-catalyzed rearrangement of 1,4-disubstituted cubanes containing two electron-withdrawing groups was explored (Scheme 1). Cubane 1,4-dimethyl and 1,4-di-*tert*-butyl esters rearranged to give the corresponding 2,6-disubstituted cuneanes **2a** and **2b** in high yields. Replacement of one of the methyl esters in cubane **1a** with a cyano group or various amides gave substrates that also rearranged successfully to give cuneanes **2c**–**2h** in 36–90% yield, with none of the alternative 1,3-disubstituted regioisomer detected. Regarding the amide of products **2d**–**2h**, both secondary (**2d** and **2e**) and tertiary (**2f**–**2h**) amides with various alkyl, aryl, or alkoxy substituents are tolerated. 2-Oxa-6-azaspiro[3.3]heptanes are of interest as less liphophilic bioisosteres of morpholines,^15^ and using 40 mol % of AgNO_3_, a cubane containing this group rearranged readily to give cuneane **2h** in 90% yield. A benzoxazole group is also tolerated to give cuneane **2i** in 92% yield.

**Scheme 2:**
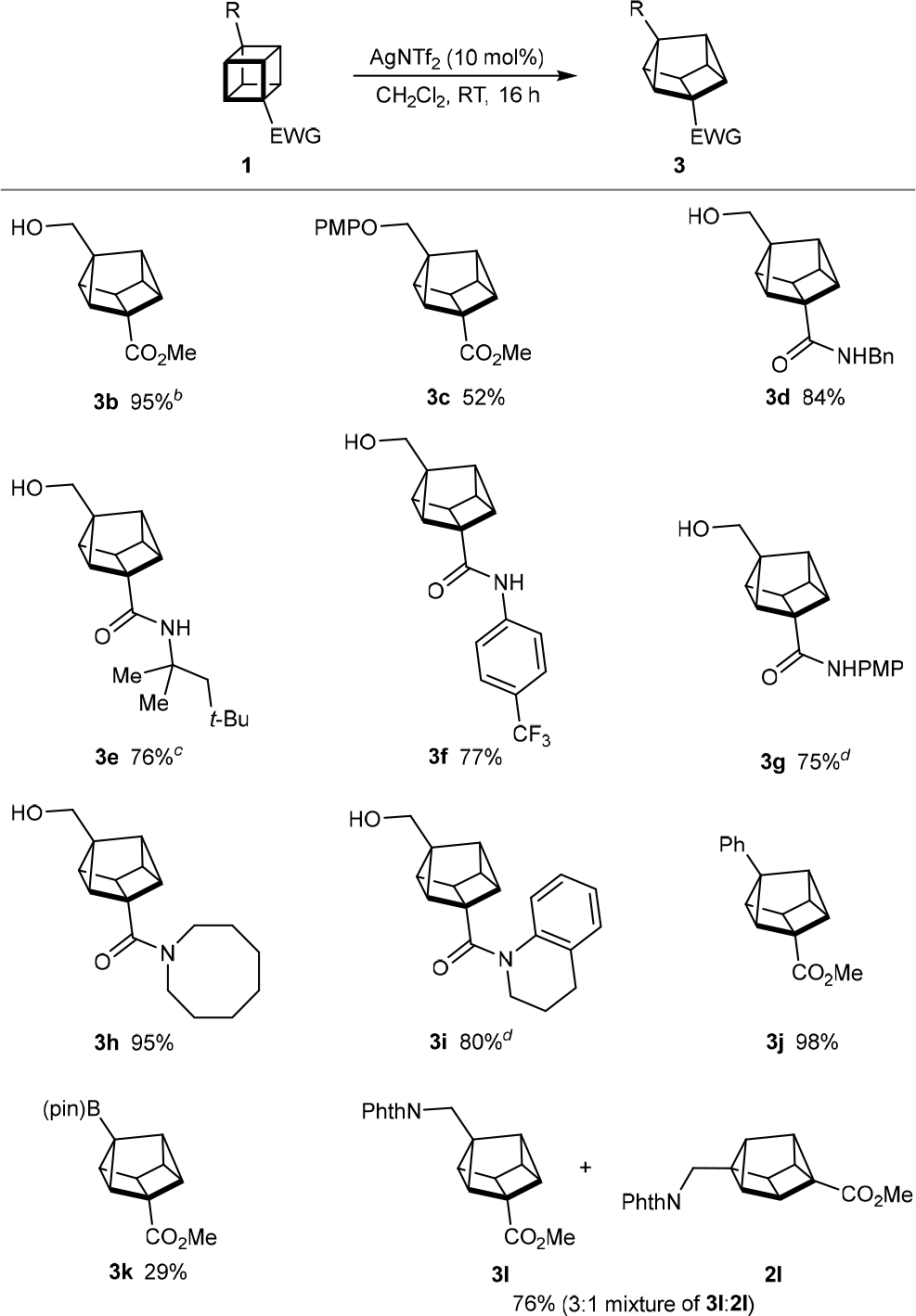
Synthesis of 1,3-Disubstituted Cuneanes Reactions were conducted with 0.20 mmol of **1** in CH_2_Cl_2_ (2.0 mL). Yields are of isolated products. PMP = *para*-methoxyphenyl. The reaction time was conducted using 1.04 mmol of cubane in CH_2_Cl_2_ (10 mL) for 10 min. Cuneane **3b** was isolated together with what appeared to be the corresponding 2,6-disubstituted regioisomer in a 24:1 ratio. Reaction conducted using AgNO_3_ (25 mol %) in toluene (1.0 mL) at 70 °C. The reaction time was 32 h.

Next, we examined the silver(I)-catalyzed rearrangements of 1,4-disubstituted cubanes containing only one electron-withdrawing group (Scheme 2). When we initiated this study, rearrangements of this class of cubane to cuneanes had not been described, and it was therefore of interest to determine the efficiency and regiochemical outcomes of these reactions.^10,11^ We found that these reactions occur much more readily than the reactions shown in Scheme 1 and give 1,3-disubstituted cuneanes as the major products, rather than 2,6-disubstituted cuneanes.^16^ From a brief examination of silver(I) salts and solvents,^17^ the use of AgNTf_2_ (10 mol %) in CH_2_Cl_2_ at room temperature was identified as being effective in giving 1,3-disubstituted cuneanes in generally good yields and high regioselectivities, despite the same combination giving poor regioselectivity in the rearrangement of cubane **1a** at 50 °C (Table 1, entry 10). These results are of significance because there were no prior examples of the selective synthesis of 1,3-disubstituted cuneanes containing two different substituents.^10,11^

Similar to the results shown in Scheme 1, the process is tolerant of a methyl ester (**3b**, **3c**, and **3j**–**3l**) and various amides (**3d**–**3i**) as the electron-withdrawing group. With respect to the second substituent, cubanes with hydroxymethyl (**3b** and **3d**–**3i**), (4-methoxyphenoxy)methyl (**3c**), or phenyl groups (**3j**) are tolerated, with the rearrangement being particularly efficient in the latter case (**3j** obtained in 98% yield). However, a cubane containing a B(pin) group gave a complex mixture of products, from which 1,3-disubstituted cuneane **3k** was the only product that could be isolated cleanly, in 29% yield. It was not possible to determine whether the corresponding 2,6-disubstituted isomer of **3k** was also formed in this reaction. A lower regioselectivity was observed in the rearrangement of a cubane with a phthalimide-protected aminomethyl group; this reaction gave a 3:1 mixture of 1,3-disubstituted isomer **3l** and 2,6-disubstituted isomer **2l**, respectively, which were isolated together in 76% yield.

Interestingly, attempts to apply the conditions used in the preparation of 1,3-disubstituted cuneanes (Scheme 2) to 2,6-disubstituted cuneanes **2g** and **2i** (Scheme 1) were unsuccessful. Heating the corresponding cubane precursors **1g** and **1i** at 50 °C for 16 h in the presence of AgNTf_2_ (10 mol %) in CH_2_Cl_2_ led to the return of unchanged starting materials (<5% conversion), despite these conditions giving a good yield in the rearrangement of cubane **1a** (Table 1, entry 10). We speculate that the coordination of Ag(I) to the Lewis basic amide or benzoxazole groups inhibits the rearrangement.

**Scheme 3:**
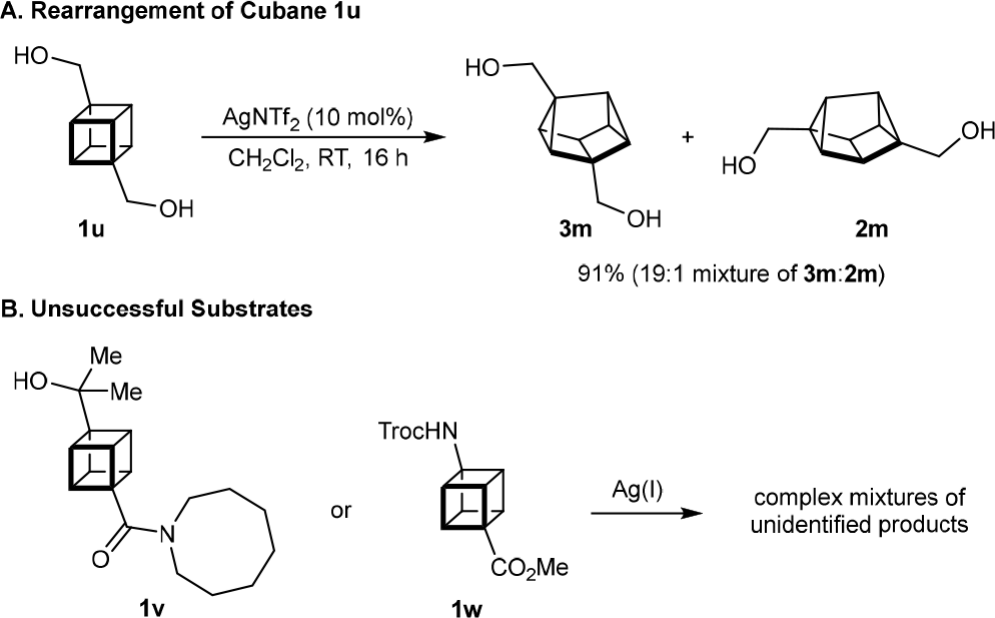
Additional Substrates

The rearrangement of cubane **1u**, which contains two hydroxymethyl groups, gave a 19:1 inseparable mixture of 1,3-disubstituted cuneane **3m** and 2,6-disubstituted cuneane **2m**, respectively, in 91% yield (Scheme 3A). Unfortunately, cubanes containing a tertiary alcohol (**1v**) or a Troc-protected amino group (**1w**) did not rearrange successfully and gave only complex mixtures of unidentified products (Scheme 3B).

**Scheme 4:**
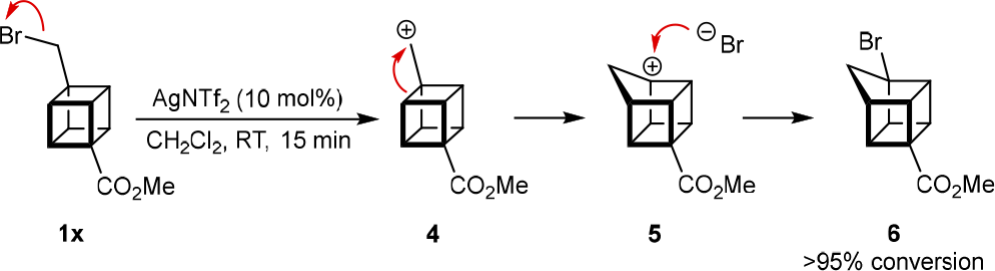
Formation of a Homocubane

The attempted rearrangement of cubane **1x**, which contains a bromomethyl group, led to rapid conversion into homocubane **6**, rather than a cuneane (Scheme 4).^18^ This reaction likely occurs through formation of the primary carbocation **4**, which undergoes a Wagner–Meerwein shift to give the homocubyl carbocation **5**, followed by trapping with a bromide anion.^19^

### Mechanistic Discussion

**Scheme 5:**
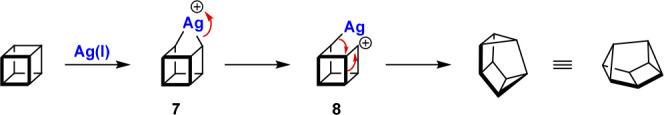
Original Mechanistic Hypothesis (Halpern, 1971)

Although previous papers describing the metal-promoted rearrangement of cubanes to cuneanes had provided tentative speculations about certain aspects of the reaction mechanism,^9f,9h,20^ no detailed mechanistic studies had been carried out until recently.^10^ In 1971, Halpern and co-workers suggested the silver(I)-promoted rearrangement of unsubstituted cubane proceeds by the oxidative addition of Ag(I) into a C–C bond to give Ag(III) species **7**, followed by heterolytic Ag–C bond cleavage to give carbocation **8**, which then undergoes σ bond rearrangement to give cuneane (Scheme 5).^20,21^ Eaton and co-workers observed that electron-withdrawing groups inhibit the reaction,^9a^ which is supported by our results (the reactions shown in Scheme 2 occur more readily that those shown in Scheme 1) and those of others.^9h,10,11^

**Scheme 6:**
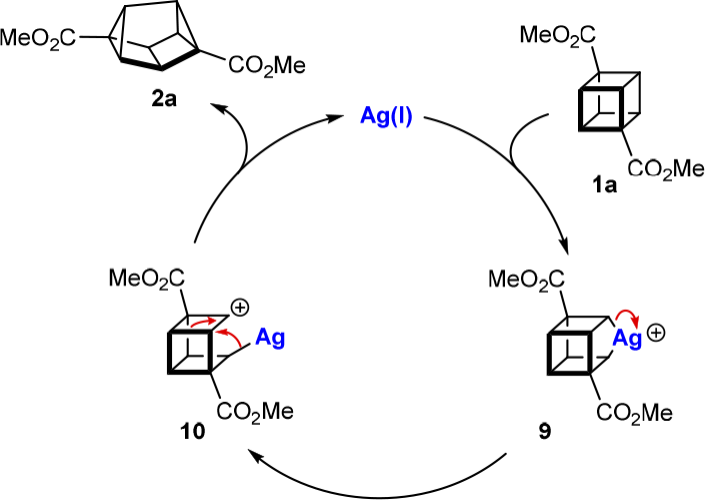
Tentative Catalytic Cycle for the Formation of 2,6-Disubstituted Cuneanes

**Scheme 7:**
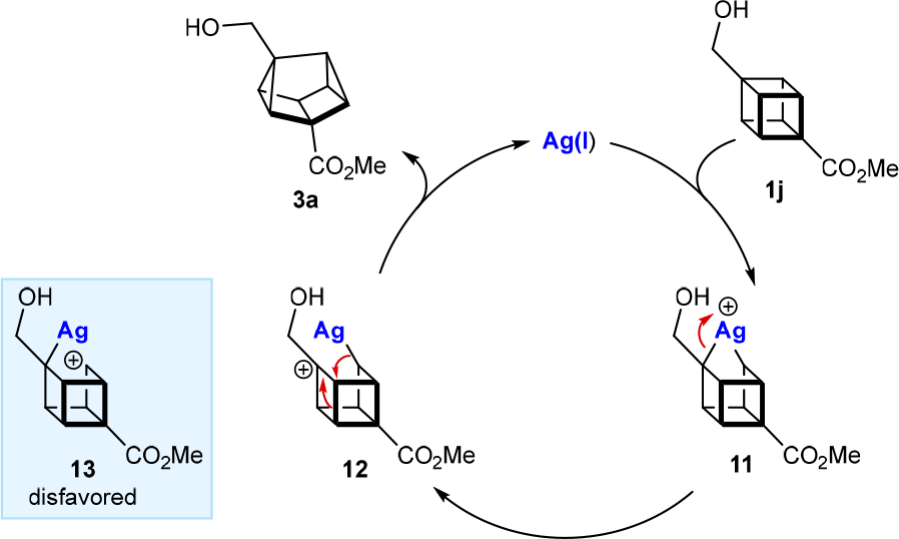
Tentative Catalytic Cycle for the Formation of 1,3-Disubstituted Cuneanes

Presumably, electron-withdrawing groups reduce the ability of the cubane C–C bonds to coordinate to Ag(I), prior to oxidative addition. It appeared reasonable to assume that the regioselectivity of the rearrangement of 1,4-disubstituted cubanes would be controlled by which of the inequivalent cubane C–C bonds would engage preferentially in oxidative addition with Ag(I), as well as which of the resulting two Ag–C bonds undergoes heterolysis. With these considerations in mind, we formulated tentative catalytic cycles for the silver(I)-catalyzed rearrangement of representative cubanes **1a** and **1j**, omitting the silver counterion for simplicity (Schemes 6 and 7). With cubane **1a**, oxidative addition of Ag(I) into one of the more electron-rich cubane C–C bonds (not adjacent to the electron-withdrawing esters) gives Ag(III) species **9** (Scheme 6).^10,20^ Heterolysis of one of the two equivalent Ag–C bonds gives carbocation **10**, which undergoes σ bond rearrangement to give 2,6-disubstituted cuneane **2a** with the release of Ag(I).

With cubane **1j**, oxidative addition of Ag(I) into one of the more electron-rich C–C bonds adjacent to the electron-donating hydroxymethyl group gives Ag(III) species **11**, which undergoes heterolysis to give the tertiary carbocation **12**, rather than the less stable secondary carbocation **13** (Scheme 7). The σ bond rearrangement of **12** then gives the 1,3-disubstituted cuneane **3a**.^22^

### Structural Analysis and Potential Applications in Medicinal Chemistry

**Figure 2:**
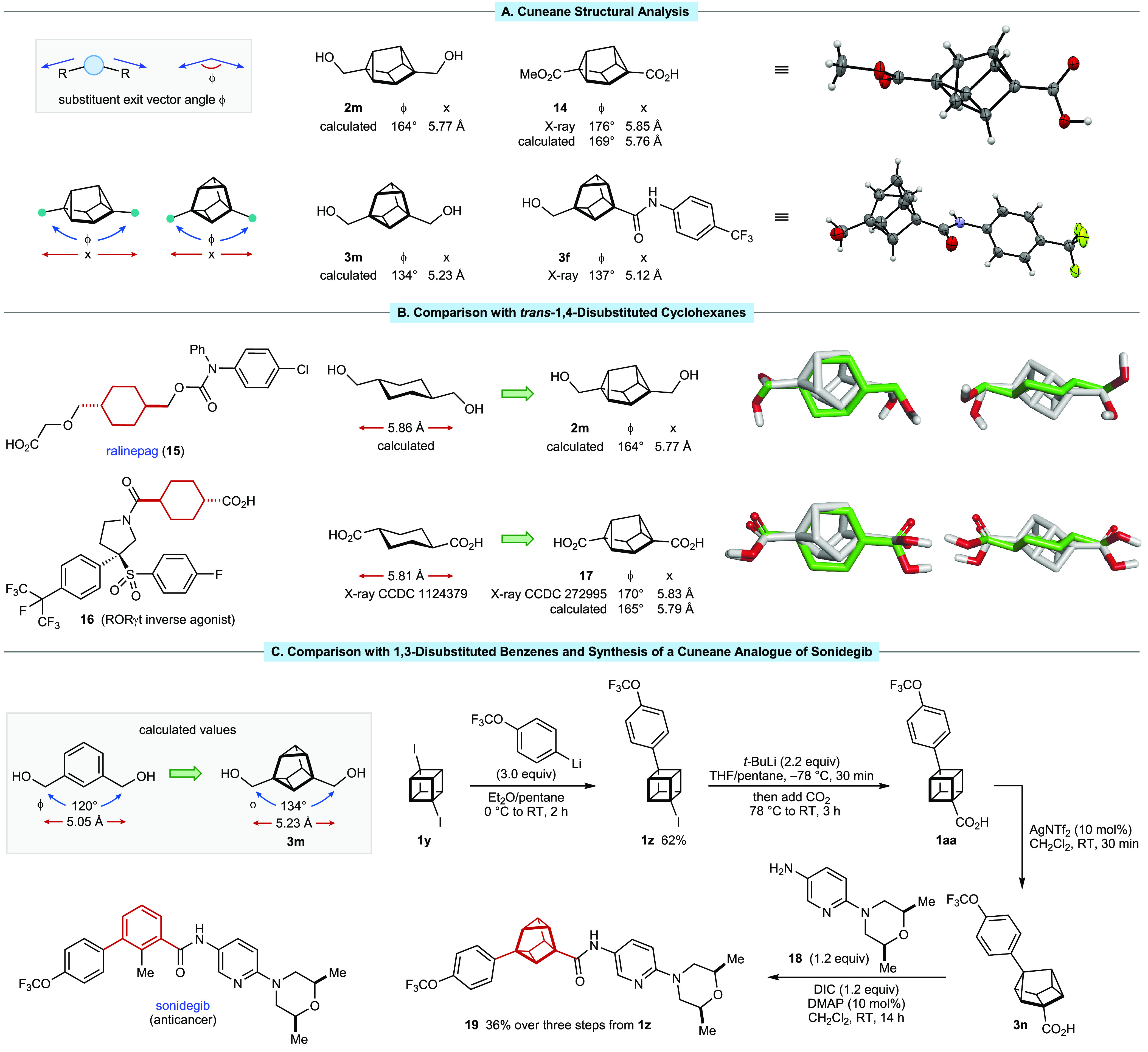
Structural analysis of cuneanes, comparison with common structures in drugs, and synthesis of a cuneane analogue of sonidegib.

With methods to prepare 2,6-disubstituted and 1,3-disubstituted cuneanes available, an assessment of their potential as scaffolds for medicinal chemistry was undertaken. First, computational studies were conducted on cuneane regioisomers **2m** and **3m** (Figure 2A).^23^ For 2,6-disubstituted cuneane **2m**, the substituent exit vector angle was calculated to be 164°, while the distance between the carbon atoms of the two hydroxymethyl groups is 5.77 Å. For 1,3-disubstituted cuneane **3m**, the exit vector angle is 134°, and the distance between the carbon atoms of the two hydroxymethyl groups is 5.23 Å. The values for cuneanes **14** (prepared by the monohydrolysis of **2a**) and **3f** were also obtained from their X-ray structures.^16^ The exit vector angle for **3f** from the X-ray data (137°) is close to the calculated value for **3m** (134°), while there is a slightly larger difference between the corresponding values for **14** (176°) and **3m** (164°). The calculated exit vector angle for **14** (169°) is lower than the value from the X-ray data (176°).

Next, a preliminary comparison of cuneanes with structures commonly seen in drugs was conducted to assess whether cuneanes could serve as isosteric replacements. This study suggested that 2,6-disubstituted cuneanes could function as rigid mimics of the diequatorial conformation of *trans*-1,4-disubstituted cyclohexanes, which appear in compounds such as ralinepag (**15**),^24^ a prostacyclin receptor (IP) agonist, and the RORγt inverse agonist **16**(25) (Figure 2B). Superimposition of energy-minimized conformations of the bis(hydroxymethyl) derivatives, as well as comparison of the calculated distances between the two hydroxymethyl groups, suggested a reasonable similarity between the two structures.^23,26^ This was confirmed by a similar comparison for *trans*-cyclohexane-1,4-dicarboxylic acid^27^ and 2,6-disubstituted cuneane **17**.^28^ Interestingly, for the dicarboxylic acid **17**, the values of the exit vector angle obtained from X-ray data and calculation were slightly lower than those of the corresponding monomethyl ester **14**.

In addition, 1,3-disubstituted cuneanes could serve as isosteric replacements for 1,3-disubstituted benzenes,^8,11^ which are ubiquitous structures in medicinal chemistry (Figure 2C).^29^ For the bis(hydroxymethyl) derivatives, although there is a difference in the exit vector angles, there is a good match in the distances between the carbon atoms of the two hydroxymethyl groups.

To demonstrate the potential of cuneanes as benzene isosteres in medicinal chemistry, we prepared an analogue **19** of sonidegib, an anticancer drug (Figure 2C).^30,31^ Following a procedure developed by Eaton and co-workers,^32^ 1,4-diiodocubane (**1y**)^33^ was reacted with 3.0 equiv of [4-(trifluoromethoxy)phenyl]lithium to give arylated cubane **1z** in 62% yield. Lithium/halogen exchange of **1z**, followed by reaction with CO_2_ gave carboxylic acid **1aa**. Smooth rearrangement of **1aa** was achieved with AgNTf_2_ (10 mol %) in CH_2_Cl_2_ to give 1,3-disubstituted cubane **3n**, which demonstrates the tolerance of a free carboxylic acid in this reaction. Finally, amide formation of **3n** with amine **18** using DIC in the presence of catalytic DMAP gave **19** in 36% yield over the three steps from **1z**.^16^

Selected physicochemical properties of a small set of compounds were then measured and compared (Table 2). LogP, p*K*_a_ in water, and aqueous solubility were chosen because they are common parameters targeted for modification in medicinal chemistry to improve the properties of lead compounds. First, 4-(methoxycarbonyl)benzoic acid (**20**), 1,4-disubstituted cubane **S1**, and 2,6-disubstituted cuneane **14** were compared to examine the effect of changing the core scaffold linking the methyl ester and carboxylic acid, which are functional groups commonly seen in drugs. Compared with the aromatic compound **20**, both its cubane (**S1**) and 2,6-disubstituted cuneane (**14**) analogues are less lipophilic and more soluble in water, whereas there are only small differences in the p*K*_a_ values. Cubane **S1** is less lipophilic than cuneane **14** and has a lower aqueous solubility.

**Table 2:**
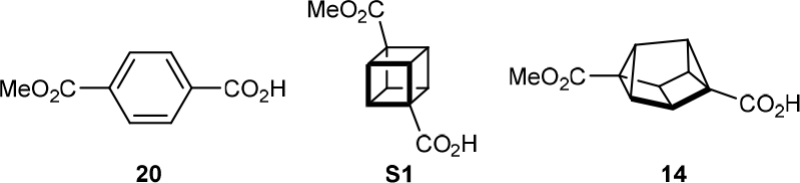
Comparison of Physicochemical Properties of a Benzene, Cubane, and Cuneane Series^a^

	20	S1	14
MW	180.16	206.20	206.20
logP	2.02 ± 0.02	0.02 ± 0.01	0.72 ± 0.01
p*K*_a_ in H_2_O	3.84 ± 0.01	3.96 ± 0.01	3.99 ± 0.02
aqueous solubility (mM)	1.384	148.8	209.8

a See the Supporting Information for experimental details.

The cuneane analogue **19** of sonidegib was then compared with sonidegib itself (Table 3). The measured logP value of **19** is comparable to a literature value for sonidegib,^34^ while its aqueous solubility was too low to be measured. Compared with sonidegib, **19** showed higher intrinsic clearance rates and shorter half-lives in human and mouse liver microsomes.^8b^ Interestingly, this observation is in contrast with a bicyclo[3.1.1]heptane (BCHep) analogue of sonidegib, which showed increased metabolic stability in human and mouse liver microsomes compared with sonidegib.^8b^

**Table 3:**
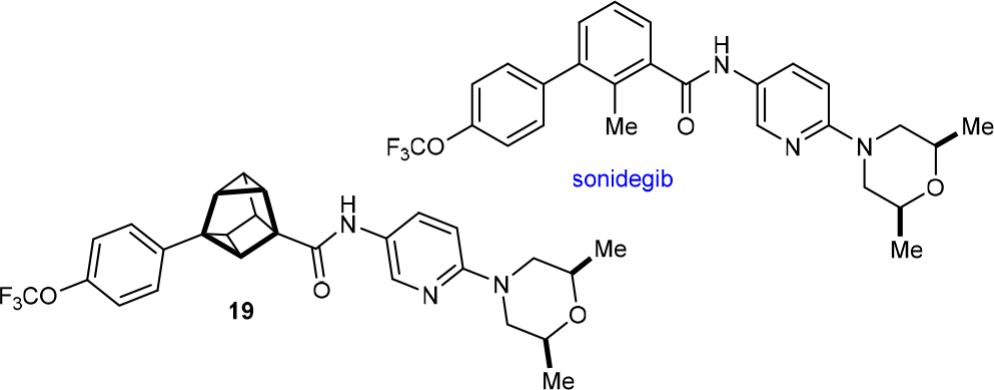
Comparison of Physicochemical and Metabolic Properties of Sonidegib and a Cuneane Analogue^a^

	sonidegib	**19**
MW	485.51	497.52
logP	4.26 (ref (32))	4.74 ± 0.23
aqueous solubility (mM)	<1.6 × 10^–3^ (ref ^8b^)	–
human liver microsome stability	20/70 (ref ^8b^)	76/19
Cl_int_ (μL/min/mg)/*t*_1/2_ (min)		
mouse liver microsome stability	26/53 (ref ^8b^)	61/23
Cl_int_ (μL/min/mg)/*t*_1/2_ (min)		

a See the Supporting Information for experimental details.

b The solubility was too low to be measured.

## Conclusions

We described the silver(I)-catalyzed rearrangement of 1,4-disubstituted cubanes to give cuneanes. The regioselectivity of the rearrangement is dependent on the nature of the cubane substituents: cubanes with two electron-withdrawing groups rearrange to give 2,6-disubstituted cuneanes, while cubanes containing one or more electron-donating groups rearrange more readily to give 1,3-disubstituted cuneanes. A preliminary assessment of cuneanes as scaffolds for medicinal chemistry was also performed, which suggests cuneanes could have applications as isosteres of *trans*-1,4-disubstituted cyclohexanes and 1,3-disubstituted benzenes. An analogue of the anticancer drug sonidegib was prepared, in which the 1,2,3-trisubstituted benzene was replaced with a 1,3-disubstituted cuneane. We hope this investigation will inform the continued study of this underexplored class of strained hydrocarbon.^10,11^

## Data Availability

The data underlying this study are openly available in the Nottingham Research Data Management Repository at: 10.17639/nott.7314.

## Supporting Information Available

Experimental procedures and full spectroscopic data for new compounds (PDF)
