# Supplementary material for: Silver(I)-Catalyzed Synthesis of Cuneanes from Cubanes and their Investigation as Isosteres
Source: J Am Chem Soc. 2023 Jul 21;145(30):16365–73. doi: 10.1021/jacs.3c03207 (PMC10401713; doi:10.1021/jacs.3c03207)
Supplement: Supplementary file 1 — ja3c03207_si_001.pdf [file ja3c03207_si_001.pdf]

Silver(I)-Catalyzed Synthesis of Cuneanes from Cubanes and their Investigation as Isosteres

Elliot Smith,[1,2] Kieran D. Jones,[1,2] Luke O'Brien,[1,2] Stephen P. Argent,[2] Christophe Salome,[3] Quentin Lefebvre,[3] Alain Valery,[3] Mina Böcü,[3] Graham N. Newton,[1,2] and Hon Wai Lam*,[1,2]

[1] The GlaxoSmithKline Carbon Neutral Laboratories for Sustainable Chemistry, University of Nottingham, Jubilee Campus, Triumph Road, Nottingham, NG7 2TU, United Kingdom

[2] School of Chemistry, University of Nottingham, University Park, Nottingham, NG7 2RD, United Kingdom

[3] SpiroChem AG, 4058 Basel, Switzerland

Supporting Information

Contents

1. General Information....................2
2. Synthesis of Cubanes....................4
3. Silver(I)-Catalyzed Rearrangements of Cubanes....................19
4. Computational Structural Analysis....................30
5. Synthesis of a Cuneane Analog of Sonidegib....................31
6. Physicochemical Measurements....................35
7. Microsome Stability Measurements for the Cuneane Analog of Sonidegib....................37
8. NMR Spectra....................39
9. References....................85

1. General Information

Reactions. All air-sensitive reactions were carried out under an inert atmosphere using oven-dried apparatus.

Reagents and Solvents. "Petrol" refers to petrol boiling point 40-60 °C. All commercially available reagents were used as received unless otherwise stated.

Chromatography. Thin layer chromatography (TLC) was performed on Merck DF-Alufoilien 60F254 0.2 mm precoated plates. Compounds were visualized by exposure to UV light or by dipping the plates into solutions of ceric ammonium molybdate, potassium permanganate, ninhydrin, phosphomolybdic acid, or vanillin followed by gentle heating. Column chromatography was carried out using silica gel (Fisher Scientific 60 Å particle size 35-70 micron or Fluorochem 60 Å particle size 40-63 micron), or using a CombiFlash Nextgen 100 (Teledyne ISCO) fitted with Redisep® silver silica gel disposable flash columns, or using a Biotage Isolera 4 fitted with Agela Claricep silica gel disposable flash columns.

Melting Points. Melting points are uncorrected. The solvent of recrystallization is reported in parentheses.

IR Spectra. Infrared (IR) spectra were recorded on the neat compound using the attenuated total refraction technique.

NMR Spectra. ^{1}H and ^{13}C NMR spectra were referenced to external tetramethylsilane *via* the residual protonated solvent (^{1}H) or the solvent itself (^{13}C). All chemical shifts are reported in parts per million (ppm). For $CDCl_3$, the shifts are referenced to 7.26 ppm for ^{1}H NMR spectroscopy and 77.16 ppm for ^{13}C NMR spectroscopy. For DMSO-D_6, the shifts are referenced to 2.50 ppm for ^{1}H NMR spectroscopy and 39.52 ppm for ^{13}C NMR spectroscopy. ^{19}F NMR spectra were referenced through the solvent lock (^{2}H) signal according to the IUPAC-recommended secondary referencing method following Bruker protocols. ^{13}C NMR Assignments were made using the DEPT sequence with secondary pulses at 90° and 135° or using 2D NMR spectroscopy techniques including HSQC and HMBC. Coupling constants (J) are quoted to the nearest 0.1 Hz.

Mass Spectra. High-resolution mass spectra were recorded using electrospray ionization (ESI) techniques.

X-ray Crystallography. For compounds **1k**, **1v**, and **17**, single crystal X-ray diffraction data were collected on an Oxford Diffraction GV1000 (TitanS2 CCD area detector, mirror-monochromated Cu-Kα radiation source; $\lambda = 1.54184$ Å, ω scans). For compound **19**·TFA monohydrate, single crystal X-ray diffraction data were collected on an XtaLAB PRO MM007 (PILATUS3 R 200K Hybrid Pixel

Array detector, mirror-monochromated Cu-Kα radiation source; λ = 1.54184 Å, ω scans). Single crystals were selected and mounted using Fomblin® (YR-1800 perfluoropolyether oil) on a polymer-tipped MiTeGen MicroMountTM and cooled rapidly to 120 K in a stream of cold N_2 using an Oxford Cryosystems open flow cryostat.[1] Cell parameters were refined from the observed positions of all strong reflections and absorption corrections were applied using a Gaussian numerical method with beam profile correction (CrysAlisPro).[2] Structures were solved within Olex2[3] by dual space iterative methods (SHELXT)[4] and all non-hydrogen atoms refined by full-matrix least-squares on all unique F2 values with anisotropic displacement parameters (SHELXL).[5] Hydrogen atoms were refined both freely (see refinement experimental section for details) and with constrained riding geometries and thermal parameters linked to Uiso their parent atoms). Structures were checked with checkCIF (http://checkcif.iucr.org). CCDC 2248869, 2248870, 2248872, and 2279035 contain the supplementary data for these compounds. These data can be obtained free of charge from The Cambridge Crystallographic Data Centre via www.ccdc.cam.ac.uk/data_request/cif.

For compound **3e**, X-ray diffraction measurements were performed in Experiments Hutch 1 (EH1) of Beamline I19, at Diamond Light Source.[6] The data were collected at a wavelength of 0.6889 Å on a Fluid Film Devices 3-circle fixed-chi diffractometer using a Dectris Pilatus 2M detector. The crystal was mounted on a MiTeGen micromount using a using Fomblin® (YR-1800 perfluoropolyether oil), flash frozen in liquid nitrogen before being transported to the Synchrotron where it was cooled for data collection by a Cryostream nitrogen-gas stream.[1] The collected frames were integrated using DIALS[7] software and the data were corrected for absorption effects using AIMLESS,[8] an empirical method. The structure was solved by dual-space methods[4] and refined by least-squares refinement on all unique measured F2 values.[5] CCDC 2248871 contains the supplementary data for this compound. These data can be obtained free of charge from The Cambridge Crystallographic Data Centre via www.ccdc.cam.ac.uk/data_request/cif.

2. Synthesis of Cubanes

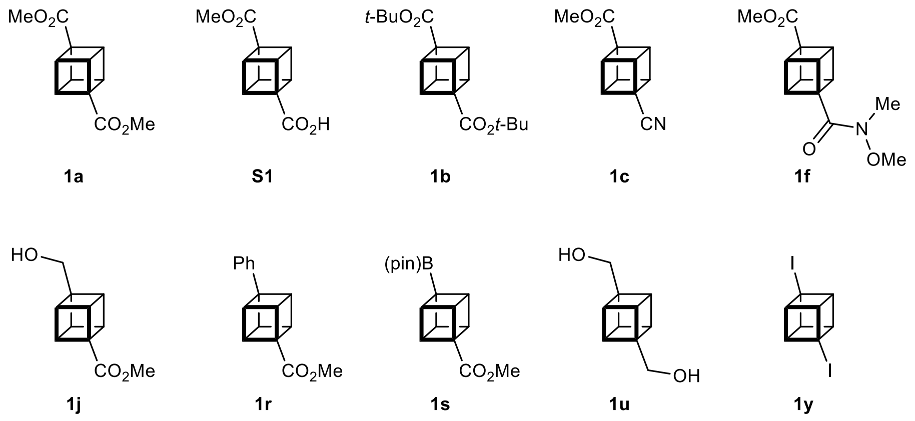

Cubanes **1a**,[9] **S1**,[10] **1b**,[11] **1c**,[10] **1f**,[10] **1j**,[12] **1r**,[13] **1s**,[14] **1u**,[15] and **1y**[16] were prepared according to literature procedures.

Methyl 4-(benzylcarbamoyl)cubane-1-carboxylate (1d)

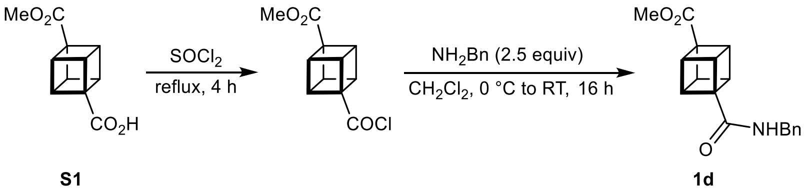

An oven-dried microwave vial equipped with a stirrer bar was charged with cubane **S1**[10] (900 mg, 4.36 mmol). The vial was sealed with a Teflon-lined cap and purged with argon for 5 min. $SOCl_2$ (9 mL) was then added and the resulting solution was heated at reflux for 4 h. The solution was then cooled to room temperature and excess $SOCl_2$ was removed under reduced pressure to afford the crude acid chloride. The crude residue was dissolved in anhydrous CH_2Cl_2 (22.5 mL) and the solution was cooled to 0 °C in an ice bath. Benzylamine (1.20 mL, 10.0 mmol) was added dropwise and the reaction was stirred for 16 h (while allowing to warm slowly to room temperature), diluted with CH_2Cl_2 (22.5 mL) and quenched with saturated aqueous NH_4Cl solution (45 mL). The organic layer was washed with 1 M aqueous HCl solution (45 mL) and brine (45 mL), dried ($MgSO_4$), filtered, and concentrated *in vacuo* to give *cubane* **1d** as an off-white solid (1.14 g, 88%), which was used without further purification. R_f = 0.15 (40% EtOAc/petrol); m.p. 141–142 °C (Et_2O); IR 3362 (NH), 2984, 1709 (C=O), 1628 (C=O), 1452, 1321, 1243, 1086, 692, 612 cm^{-1}; ^1H NMR (400 MHz, $CDCl_3$) δ 7.41–7.25 (5H, m, Ar**H**), 5.89 (1H, br s, N**H**), 4.51–4.44 (2H, d, C**H**$_2$), 4.26–4.17 (6H, m, cubyl C**H**), 3.72 (3H, s, C**H**$_3$); ^{13}C NMR (101 MHz, $CDCl_3$) 172.1 (C), 171.2 (C), 138.2 (C), 128.9 (2 × CH), 128.1 (2 × CH), 127.8 (CH), 57.8 (C), 55.9 (C), 51.8 (CH_3), 47.1 (3 × CH), 46.8 (3 × CH), 43.5 (CH_2); HRMS (ESI) Exact mass calculated for $[C_{18}H_{17}NNaO_3]^+$ $[M+Na]^+$: 318.1101, found 318.1098.

Methyl 4-{[4-(trifluoromethyl)phenyl]carbamoyl}cubane-1-carboxylate (1e)

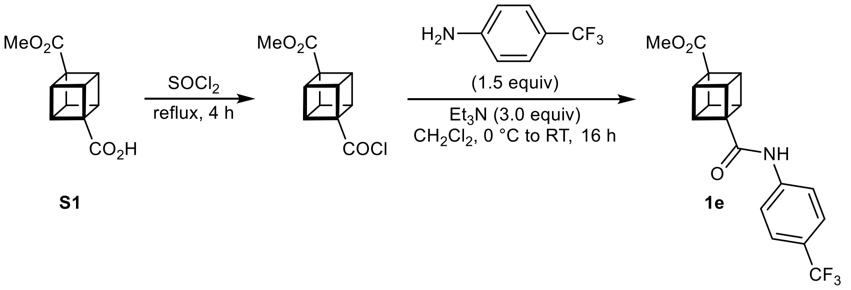

An oven-dried microwave vial equipped with a stirrer bar was charged with cubane **S1**[10] (800 mg, 3.88 mmol). The vial was sealed with a Teflon-lined cap and purged with argon for 5 min. $SOCl_2$ (8 mL) was then added and the resulting solution was heated at reflux for 4 h. The solution was then cooled to room temperature and excess $SOCl_2$ was removed under reduced pressure to afford the crude acid chloride. The crude residue was dissolved in anhydrous CH_2Cl_2 (20 mL) and the solution was cooled to 0 °C in an ice bath. Et_3N (1.6 mL, 11.6 mmol) followed by 4-trifluoromethylaniline (942 mg, 5.82 mmol) were added dropwise. The reaction was stirred for 16 h (while allowing to warm slowly to room temperature), diluted with CH_2Cl_2 (20 mL), and quenched with saturated aqueous NH_4Cl solution (40 mL). The organic layer was washed with 1 M aqueous HCl solution (40 mL) and brine (40 mL), dried ($MgSO_4$), filtered, and concentrated *in vacuo*. Purification of the residue by column chromatography (20% EtOAc/pentane to 40% EtOAc/pentane) gave *cubane* **1e** as a white solid (599 mg, 44%). R_f = 0.33 (40% EtOAc/pentane); m.p. 232–233 °C ($CDCl_3$); IR 3340 (NH), 2978, 1722 (C=O), 1650 (C=O), 1508, 1320, 1112, 1066, 828, 668 cm^{-1}; ^1H NMR (400 MHz, $CDCl_3$) δ 7.72–7.65 (2H, m, Ar**H**), 7.61–7.56 (2H, m, Ar**H**), 4.35–4.26 (6H, m, cubyl C**H**), 3.73 (3H, s, C**H**$_3$); ^{13}C NMR (101 MHz, $CDCl_3$) δ 171.9 (C), 169.7 (C), 140.7 (C), 126.5 (q, $J_{C\text{-}F}$ = 3.7 Hz, 2 × CH), 126.4 (q, $J_{C\text{-}F}$ = 32.6 Hz, C), 124.2 (q, $J_{C\text{-}F}$ = 271.6 Hz, C), 119.4 (2 × CH), 58.5 (C), 56.0 (C), 51.9 (CH_3), 47.4 (3 × CH), 46.8 (3 × CH); ^{19}F NMR (376 MHz, $CDCl_3$) –62.1 (s, 3 × F); HRMS (ESI) Exact mass calculated for $[C_{18}H_{14}F_3NNaO_3]^+$ $[M+Na]^+$: 372.0818, found 372.0811.

Methyl [(4-methoxyphenyl)carbamoyl]cubane-1-carboxylate (S2)

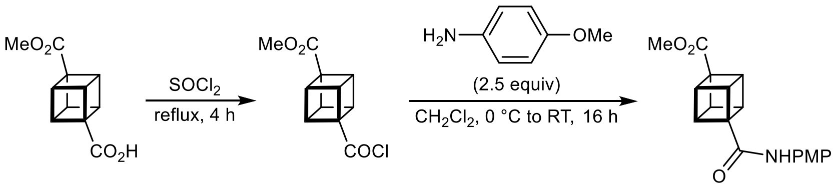

An oven-dried microwave vial equipped with a stirrer bar was charged with cubane **S1**[10] (400 mg, 1.94 mmol). The vial was sealed with a Teflon-lined cap and purged with argon for 5 min. $SOCl_2$ (4 mL) was then added and the resulting solution was heated at reflux for 4 h. The solution was then cooled to room temperature and excess $SOCl_2$ was removed under reduced pressure to afford the

crude acid chloride. The crude residue was dissolved in anhydrous CH_2Cl_2 (10 mL) and the solution was cooled to 0 °C in an ice bath. *p*-Anisidine (526 mg, 4.27 mmol) was added portionwise. The reaction was stirred for 16 h (while allowing to warm slowly to room temperature), diluted with CH_2Cl_2 (10 mL), and quenched with saturated aqueous NH_4Cl solution (20 mL). The organic layer was washed with 1 M aqueous HCl solution (20 mL) and brine (20 mL), dried ($MgSO_4$), filtered, and concentrated *in vacuo*. Purification of the residue by column chromatography (60% EtOAc/pentane to 100% EtOAc) gave *cubane* **S2** as a white solid (479 mg, 79%). R_f = 0.19 (40% EtOAc/petrol); m.p. 224–225 °C (Et_2O/EtOAc); IR 3216 (NH), 2995, 1719 (C=O), 1641 (C=O), 1509, 1219, 1088, 1034, 823, 521 cm^{-1}; ^1H NMR (500 MHz, $CDCl_3$) δ 7.45 (2H, d, J = 9.0 Hz, Ar**H**), 7.19 (1H, br s, N**H**), 6.85 (2H, d, J = 8.9 Hz, Ar**H**), 4.32–4.22 (6H, m, cubyl C**H**), 3.78 (3H, s, C**H**$_3$), 3.72 (3H, s, C**H**$_3$); ^{13}C NMR (126 MHz, $CDCl_3$) δ 172.1 (C), 169.3 (C), 156.6 (C), 130.7 (C), 121.7 (2 × CH), 114.3 (2 × CH), 58.5 (C), 56.0 (C), 55.6 (CH_3), 51.8 (CH_3), 47.3 (3 × CH), 46.8 (3 × CH); HRMS (ESI) Exact mass calculated for $[C_{18}H_{18}NO_4]^+$ $[M+H]^+$: 312.1230, found 312.1230.

Methyl 4-(azocane-1-carbonyl)cubane-1-carboxylate (1g)

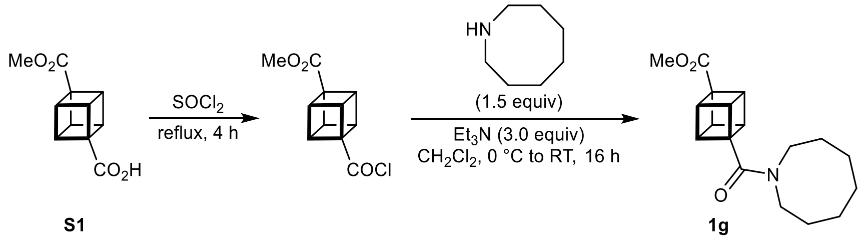

S1 1g

An oven-dried microwave vial equipped with a stirrer bar was charged with cubane **S1**[10] (500 mg, 2.42 mmol). The vial was sealed with a Teflon-lined cap and purged with argon for 5 min. $SOCl_2$ (5 mL) was then added and the resulting solution was heated at reflux for 4 h. The solution was then cooled to room temperature and excess $SOCl_2$ was removed under reduced pressure to afford the crude acid chloride. The crude residue was dissolved in anhydrous CH_2Cl_2 (12.5 mL) and the solution was cooled to 0 °C in an ice bath. Et_3N (1.0 mL, 7.26 mmol) and azocane (412 mg, 3.64 mmol) were added dropwise. The reaction was stirred for 16 h (while allowing to warm slowly to room temperature), diluted with CH_2Cl_2 (12.5 mL) and quenched with saturated aqueous NH_4Cl solution (25 mL). The organic layer was washed with 1 M aqueous HCl solution (25 mL) and brine (25 mL), dried ($MgSO_4$), filtered, and concentrated *in vacuo*. Purification of the residue by column chromatography (40% EtOAc/cyclohexane to 80% EtOAc/cyclohexane) gave *cubane* **1g** as a white solid (474 mg, 65%). R_f = 0.56 (70% EtOAc/cyclohexane); m.p. 74–75 °C (Et_2O); IR 2987, 2924, 2860, 1711 (C=O), 1611 (C=O), 1468, 1238, 1193, 967, 829 cm^{-1}; ^1H NMR (500 MHz, $CDCl_3$) δ 4.35–4.10 (6H, m, cubyl C**H**), 3.70 (3H, s, C**H**$_3$), 3.45–3.34 (2H, m, NC**H**$_2$), 3.21–3.11 (2H, m, NC**H**$_2$), 1.81–1.68 (4H, m, azocanyl C**H**$_2$), 1.60 (2H, p, J = 5.9 Hz, azocanyl C**H**$_2$), 1.56–1.43 (4H,

m, azocanyl C**H**$_2$); ^{13}C NMR (126 MHz, $CDCl_3$) δ 172.3 (C), 170.4 (C), 58.8 (C), 54.8 (C), 51.7 (CH_3), 48.7 (CH_2), 47.2 (3 × CH), 46.6 (3 × CH), 46.0 (CH_2), 26.69 (CH_2), 26.65 (CH_2), 26.5 (CH_2), 25.9 (CH_2), 23.7 (CH_2); HRMS (ESI) Exact mass calculated for $[C_{18}H_{24}NO_3]^+$ $[M+H]^+$: 302.1751, found 302.1751.

Methyl 4-(2-oxa-6-azaspiro[3.3]heptane-6-carbonyl)cubane-1-carboxylate (1h)

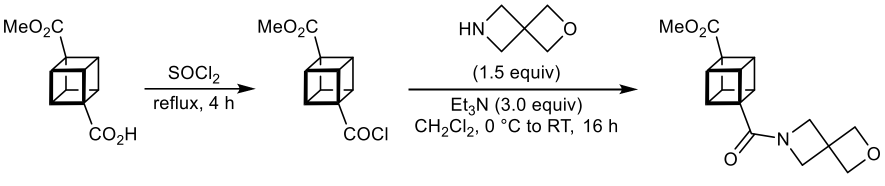

An oven-dried microwave vial equipped with a stirrer bar was charged with cubane **S1**[10] (500 mg, 2.42 mmol). The vial was sealed with a Teflon-lined cap and purged with argon for 5 min. $SOCl_2$ (5 mL) was then added and the resulting solution was heated at reflux for 4 h. The solution was then cooled to room temperature and excess $SOCl_2$ was removed under reduced pressure to afford the crude acid chloride. The crude residue was dissolved in anhydrous CH_2Cl_2 (12.5 mL) and the solution was cooled to 0 °C in an ice bath. Et_3N (1.0 mL, 7.26 mmol) followed by 2-oxa-6-azaspiro[3.3]heptane (361 mg, 3.64 mmol) were added dropwise. The reaction was stirred for 16 h (while allowing to warm slowly to room temperature), diluted with CH_2Cl_2 (12.5 mL) and quenched with saturated aqueous NH_4Cl solution (25 mL). The organic layer was washed with 1 M aqueous HCl solution (25 mL) and brine (25 mL), dried ($MgSO_4$), filtered, and concentrated *in vacuo* to leave *cubane* **1h** as a white solid, which was used without further purification. **Note:** Cubane **1h** was found to be unstable on silica gel. m.p. 158–159 °C (Et_2O); IR 2985, 2870, 1714 (C=O), 1614 (C=O), 1449, 1319, 1201, 1085, 953, 889 cm^{-1}; ^{1}H NMR (500 MHz, $CDCl_3$) δ 4.80–4.73 (4H, m, 2 × C**H**$_2$O), 4.28 (2H, br s, NC**H**$_2$), 4.25-4.17 (6H, m, cubyl C**H**), 4.18 (2H, br s, NC**H**$_2$), 3.70 (3H, s, C**H**$_3$); ^{13}C NMR (126 MHz, $CDCl_3$) δ 172.0 (C), 170.9 (C), 80.9 (2 × CH_2), 59.4 (CH_2), 58.3 (CH_2), 56.9 (C), 55.6 (C), 51.7 (CH_3), 46.9 (3 × CH), 46.7 (3 × CH), 38.9 (C); HRMS (ESI) Exact mass calculated for $[C_{16}H_{17}NO_4]^+$ $[M+H]^+$: 288.1230, found 288.1231.

Methyl 4-[(2-hydroxyphenyl)carbamoyl]cubane-1-carboxylate (S3)

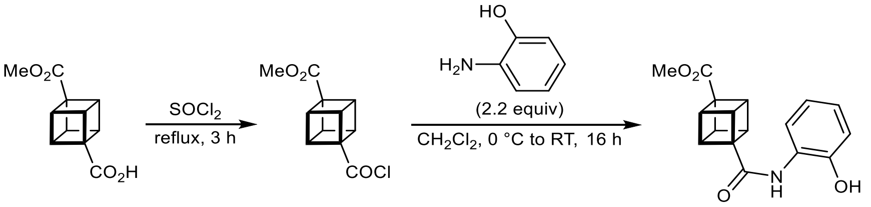

An oven-dried microwave vial equipped with a stirrer bar was charged with cubane **S1**[10] (500 mg, 2.42 mmol). The vial was sealed with a Teflon-lined cap and purged with argon for 5 min. $SOCl_2$

(5 mL) was then added and the resulting solution was heated at reflux for 3 h. The solution was then cooled to room temperature and excess $SOCl_2$ was removed under reduced pressure to afford the crude acid chloride. The crude residue was dissolved in anhydrous CH_2Cl_2 (12.5 mL) and the solution was cooled to 0 °C in an ice bath. 2-Aminophenol (freshly recrystallized from H_2O, 581 mg, 5.32 mmol) was then added portionwise. The reaction was stirred for 16 h (while allowing to warm slowly to room temperature), diluted with CH_2Cl_2 (12.5 mL) and quenched with saturated aqueous NH_4Cl solution (25 mL). The organic layer was washed with 1 M aqueous HCl solution (25 mL) and brine (25 mL), dried (Na_2SO_4), filtered, and concentrated *in vacuo*. Purification of the residue by column chromatography (100% CH_2Cl_2 to 5% MeOH/ CH_2Cl_2) followed by recrystallization from hot *i*-PrOH gave *cubane* **S3** as a white solid (381 mg, 53%). R_f = 0.21 (3% MeOH/CH_2Cl_2); m.p. 200–201 °C (*i*-PrOH), IR 3243 (OH), 1709 (C=O), 1635 (C=O), 1595, 1537, 1452, 1334, 1232, 1206, 1103 cm^{-1}; ^{1}H NMR (400 MHz, DMSO-D_6) δ 9.66 (1H, br s, O**H** or N**H**), 9.01 (1H, br s, O**H** or N**H**), 7.60 (1H, d, *J* = 7.9 Hz, Ar**H**), 7.02–6.95 (1H, m, Ar**H**), 6.87 (1H, dd, *J* = 8.1, 1.5 Hz, Ar**H**), 6.78 (1H, td, *J* = 7.6, 1.5 Hz, Ar**H**), 4.26–4.24 (3H, cubyl C**H**), 4.19–4.16 (3H, cubyl C**H**), 3.64 (3H, s, C**H**$_3$); ^{13}C NMR (151 MHz, DMSO-D_6) δ 171.3 (C), 169.7 (C), 148.7 (C), 125.7 (C), 125.4 (CH), 123.3 (CH), 119.1 (CH), 116.1 (CH), 57.7 (C), 54.9 (C), 51.3 (CH_3), 46.7 (3 × CH), 46.0 (3 × CH); HRMS (ESI) Exact mass calculated for $[C_{17}H_{15}NO_4Na]^+$ $[M+Na]^+$: 320.0893, found 320.0894.

Methyl 4-(benzo[d]oxazol-2-yl)cubane-1-carboxylate (1i)

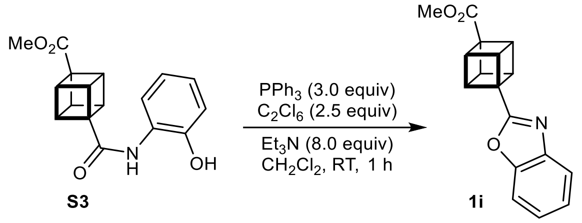

Triphenylphosphine (250 mg, 2.52 mmol) and Et_3N (0.94 mL, 6.73 mmol) were added to a solution of hexachloroethane (498 mg, 2.10 mmol) in CH_2Cl_2 (5 mL). The resulting solution was stirred at room temperature for 5 min. Cubane **S3** (250 mg, 0.84 mmol) was added and the mixture was stirred at room temperature for a further 1 h. The reaction was concentrated *in vacuo* and the resiude was purified by column chromatography (100% cyclohexane to 50% cyclohexane/EtOAc) to give *cubane* **1i** as a white solid (159 mg, 68%). R_f = 0.49 (30% EtOAc/cyclohexane); m.p. 153–154 °C (Et_2O); IR 3002, 1719 (C=O), 1612, 1569, 1455, 1433, 1317, 1240, 1225, 1198 cm^{-1}; ^{1}H NMR (400 MHz, $CDCl_3$) δ 7.74–7.65 (1H, m, Ar**H**), 7.52–7.45 (1H, m, Ar**H**), 7.36–7.28 (2H, m, Ar**H**), 4.50–4.44 (3H, m, cubyl C**H**), 4.42–4.35 (3H, m, cubyl C**H**), 3.74 (3H, s, C**H**$_3$); ^{13}C NMR (151 MHz, $CDCl_3$) δ 172.0 (C), 165.1 (C), 150.8 (C), 141.5 (C), 124.9 (CH), 124.4 (CH), 119.8 (CH), 110.5 (CH), 56.1 (C), 52.5 (C), 51.8 (CH_3), 48.0 (3 × CH_3), 47.5 (3 × CH_3); HRMS (ESI) Exact mass calculated for $[C_{17}H_{14}NO_3]^+$ $[M+H]^+$: 280.0968, found 280.0969.

Methyl 4-[(4-methoxyphenoxy)methyl]cubane-1-carboxylate (1k)

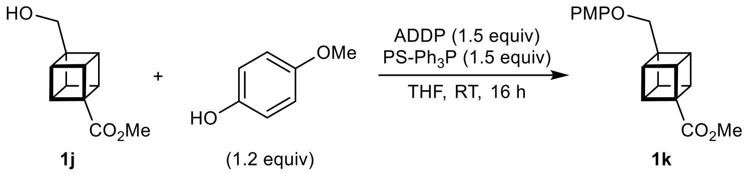

An oven-dried microwave vial equipped with a stirrer bar was charged with cubane **1i**[12] (200 mg, 1.04 mmol), 4-methoxyphenol (155 mg, 1.25 mmol) and polymer-supported triphenylphosphine (~1.6 mmol/g, 975 mg, 1.56 mmol). The vial was sealed, purged with argon, and anhydrous THF (10 mL) was added. A solution of 1,1'-(azodicarbonyl)dipiperidine (ADDP, 394 mg, 1.56 mmol) in anhydrous THF (2 mL) was added dropwise and the reaction was stirred overnight at room temperature for 16 h. The reaction was filtered and concentrated *in vacuo*. Purification of the residue by column chromatography (100% pentane to 20% EtOAc/pentane) gave *cubane* **1k** as a colorless solid (165 mg, 53%). R_f = 0.37 (10% EtOAc/pentane); m.p. 122–123 °C (EtOAc/cyclohexane); IR 1715 (C=O), 1434, 1070, 848, 819, 739, 692, 519, 495 cm^{-1}; ^{1}H NMR (500 MHz, $CDCl_3$) δ 6.87–6.84 (2H, m, Ar**H**), 6.83–6.80 (2H, m, Ar**H**), 4.21–4.16 (3H, m, cubyl C**H**), 4.05 (2H, s, C**H**$_2$), 3.95–3.91 (3H, m, cubyl C**H**), 3.76 (3H, s, C**H**$_3$), 3.71 (3H, s, C**H**$_3$); ^{13}C NMR (126 MHz, $CDCl_3$) δ 172.7 (C), 154.1 (C), 153.5 (C), 116.1 (2 × CH), 114.7 (2 × CH), 69.4 (CH_2), 57.0 (C), 56.3 (C), 55.8 (CH_3), 51.6 (CH_3), 46.8 (3 × CH), 45.2 (3 × CH); HRMS (ESI) Exact mass calculated for $[C_{18}H_{18}NaO_4]^+$ $[M+Na]^+$: 321.1097, found 321.1094.

Slow evaporation of a solution of **1k** in cyclohexane/EtOAc gave crystals suitable for X-ray crystallography.

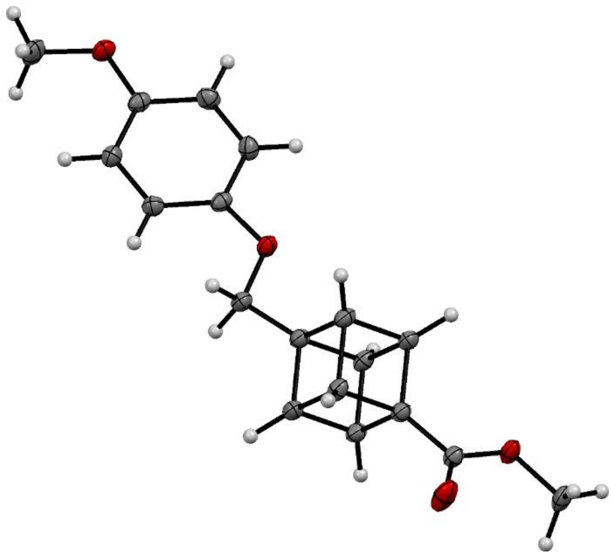

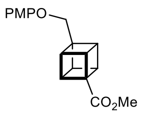

1k
CCDC 2248869

ORTEP with ellipsoid probabilities at 50%

N-Benzyl-4-(hydroxymethyl)cubane-1-carboxamide (1l)

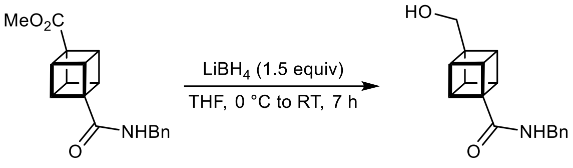

Cubane **1d** (1.00 g, 3.39 mmol) was added to a flame-dried microwave vial equipped with a stirrer bar, which was sealed with a Teflon-lined cap and purged with argon. Anhydrous THF (15 mL) was added and the resulting solution was cooled to 0 °C in an ice bath. $LiBH_4$ (4 M in THF, 1.27 mL, 5.09 mmol) was added dropwise. The reaction was allowed to warm to room temperature and stirred for 16 h. The reaction was quenched by the careful addition of H_2O (20 mL) and extracted with EtOAc (3 × 40 mL). The combined organic layers were washed with brine (30 mL), dried ($MgSO_4$), filtered, and concentrated *in vacuo*. Purification of the residue by column chromatography (100% EtOAc) gave *cubane* **1l** as a white solid (235 mg, 26%). R_f = 0.19 (100% EtOAc); m.p. 125–127 °C (Et_2O/EtOAc); IR 3331 (NH), 3266 (br, OH) 2966, 1623 (C=O), 1514, 1453, 1232, 1027, 694, 504 cm^{-1}; ^1H NMR (500 MHz, $CDCl_3$) δ 7.37–7.34 (2H, m, Ar**H**), 7.31–7.28 (3H, m, Ar**H**), 5.72 (1H, br s, N**H**), 4.48 (2H, d, *J* = 5.7 Hz, C**H**$_2$Ph), 4.12–4.10 (3H, m, cubyl C**H**), 3.89–3.87 (3H, m, cubyl C**H**), 3.78 (2H, s, C**H**$_2$OH); ^{13}C NMR (126 MHz, $CDCl_3$) δ 171.9 (C), 138.5 (C), 128.9 (2 × CH), 128.2 (2 × CH), 127.8 (CH), 63.5 (CH_2), 59.0 (C), 58.5 (C), 46.4 (3 × CH), 44.3 (3 × CH), 43.5 (CH_2); HRMS (ESI) Exact mass calculated for $[C_{17}H_{18}NO_2]^+$ $[M+H]^+$: 268.1332, found 268.1325.

Methyl 4-[(2,4,4-trimethylpentan-2-yl)carbamoyl]cubane-1-carboxylate (S4)

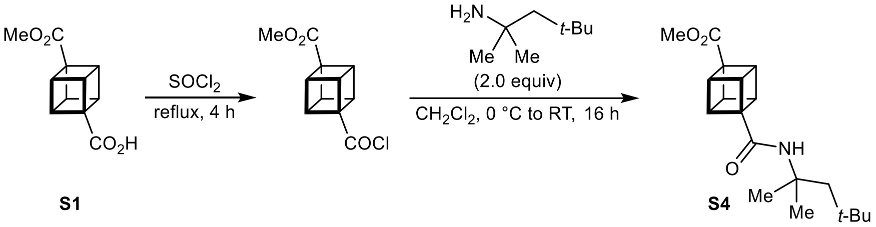

An oven-dried microwave vial equipped with a stirrer bar was charged with cubane **S1**[10] (333 mg, 1.61 mmol). The vial was sealed with a Teflon-lined cap and purged with argon for 5 min. $SOCl_2$ (3.3 mL) was then added and the resulting solution was heated at reflux for 4 h. The solution was then cooled to room temperature and excess $SOCl_2$ was removed under reduced pressure to afford the crude acid chloride. The crude residue was dissolved in anhydrous CH_2Cl_2 (8.5 mL) and the solution was cooled to 0 °C in an ice bath. *tert*-Octylamine (419 mg, 3.22 mmol) was added dropwise. The reaction was stirred for 16 h (while allowing to warm slowly to room temperature), diluted with CH_2Cl_2 (8.5 mL) and quenched with saturated aqueous NH_4Cl solution (15 mL). The organic layer was washed with 1 M aqueous HCl solution (15 mL) and brine (15 mL), dried ($MgSO_4$), filtered, and

concentrated *in vacuo* to give *cubane* **S4** as an off-white solid (479 mg, 93%), which was used without further purification. R_f = 0.42 (40% EtOAc/petrol); m.p. 176–177 °C (Et_2O); IR 3404 (NH), 2958, 1721 (C=O), 1641 (C=O), 1510, 1434, 1317, 1195, 1085, 560 cm^{-1}; ^1H NMR (500 MHz, $CDCl_3$) δ 5.25 (1H, br s, N**H**), 4.21–4.15 (3H, m, cubyl C**H**), 4.15–4.09 (3H, m, cubyl C**H**), 3.70 (3H, s, OC**H**$_3$), 1.74 (2H, s, C**H**$_2$), 1.42 (6H, s, C(C**H**$_3$)$_2$), 0.99 (9H, s, C(C**H**$_3$)$_3$); ^{13}C NMR (126 MHz, $CDCl_3$) δ 172.2 (C), 170.5 (C), 58.8 (C), 56.0 (C), 55.4 (2 × C), 52.0 (CH_2), 51.8 (CH_3), 47.0 (3 × CH), 46.6 (3 × CH), 31.9 (C), 31.6 (3 × CH_3), 29.4 (2 × CH_3); HRMS (ESI) Exact mass calculated for $[C_{19}H_{26}NNaO_3]^+$ $[M+Na]^+$: 340.1883, found 340.1884.

4-[(2,4,4-Trimethylpentan-2-yl)carbamoyl]cubane-1-carboxylic acid (S5)

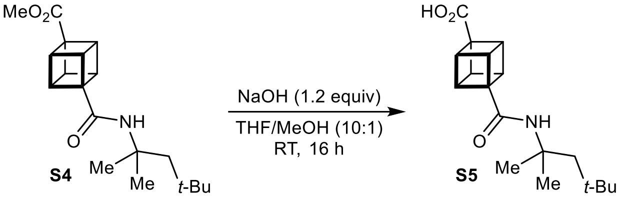

Cubane **S4** (619 mg, 1.95 mmol) was added to an oven-dried round-bottom flask equipped with a stirrer bar and THF (12.5 mL) was added. A solution of NaOH (94 mg) in MeOH (1.2 mL) was added dropwise. The resulting white suspension was stirred at room temperature for 16 h. The reaction was concentrated *in vacuo* and the resulting solid redissolved in H_2O (10 mL). The aqueous solution was washed with CH_2Cl_2 (2 × 10 mL) and then acidified to pH ~2 by the dropwise addition of 12 M HCl. The resulting white suspension was extracted with CH_2Cl_2 (3 × 10 mL). The combined organic layers were dried ($MgSO_4$), filtered, and concentrated *in vacuo* to give *cubane* **S5** as a white solid (485 mg, 82%), which was used without further purification. m.p. 198–199 °C (Et_2O/MeOH); IR 3283 (OH), 2926, 2852, 1736 (C=O), 1651 (C=O), 1535, 1440, 1246, 1194, 1174 cm^{-1}; ^1H NMR (500 MHz, $CDCl_3$) δ 5.29 (1H, s, N**H**), 4.24–4.21 (3H, m, cubyl C**H**), 4.17–4.14 (3H, m, cubyl C**H**), 1.74 (2H, s, C**H**$_2$), 1.43 (6H, s, C(C**H**$_3$)$_2$), 1.00 (9H, s, C(C**H**$_3$)$_3$); ^{13}C NMR (126 MHz, $CDCl_3$) δ 176.3 (C), 170.1 (C), 58.8 (C), 55.8 (C), 55.5 (2 × C), 52.0 (CH_2), 47.1 (3 × CH), 46.6 (3 × CH), 32.0 (C), 31.7 (3 × CH_3), 29.3 (2 × CH_3); HRMS (ESI) Exact mass calculated for $[C_{18}H_{24}NO_3]^-$ $[M-H]^-$: 302.1762, found 302.1759.

4-(Hydroxymethyl)-*N*-(2,4,4-trimethylpentan-2-yl)cubane-1-carboxamide (1m)

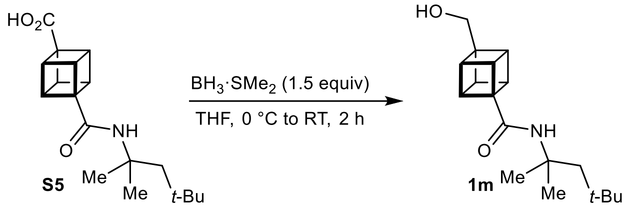

Cubane **S5** (458 mg, 1.51 mmol) was added to a flame-dried microwave vial equipped with a stirrer bar, which was sealed with a Teflon-lined cap and purged with argon. Anhydrous THF (12 mL) was added and the solution was cooled to 0 °C. A solution of $BH_3{\cdot}SMe_2$ (2 M in THF, 1.14 mL, 2.27 mmol) was added dropwise and the reaction was stirred at room temperature for 2 h. The reaction was quenched by the careful addition of H_2O (5 mL) and diluted with Et_2O (12 mL). The organic phase was washed with H_2O (2 × 10 mL) and brine (10 mL), dried ($MgSO_4$), filtered, and concentrated *in vacuo*. Purification of the residue by column chromatography (50% EtOAc/cyclohexane to 80% EtOAc/cyclohexane) gave *cubane* **1m** as a pale yellow solid (330 mg, 76%). R_f = 0.50 (100% EtOAc); m.p. 110–111 °C (Et_2O); IR 3294 (OH), 2957, 1630 (C=O), 1537, 1364, 1311, 1225, 1033, 1002, 841 cm^{-1}; ^{1}H NMR (500 MHz, $CDCl_3$) δ 5.32 (1H, s, N**H**), 4.00–3.96 (3H, m, cubyl C**H**), 3.82–3.77 (3H, m, cubyl C**H**), 3.71 (2H, s, C**H**$_2$OH), 1.70 (2H, s, C(CH_3)$_2$C**H**$_2$), 1.38 (6H, s, C(C**H**$_3$)$_2$), 0.96 (9H, s, C(C**H**$_3$)$_3$); ^{13}C NMR (126 MHz, $CDCl_3$) δ 171.6 (C), 63.1 (CH_2), 59.3 (C), 59.0 (C), 55.2 (C), 51.9 $(CH)_2$, 46.1 (3 × CH), 44.0 (3 × CH), 31.8 (C), 31.6 (3 × CH_3), 29.3 (2 × CH_3); HRMS (ESI) Exact mass calculated for $[C_{18}H_{27}NNaO_2]^+$ $[M+Na]^+$: 312.1934, found 312.1932.

4-Hydroxymethyl-*N*-[4-(trifluoromethyl)phenyl]cubane-1-carboxamide (1n)

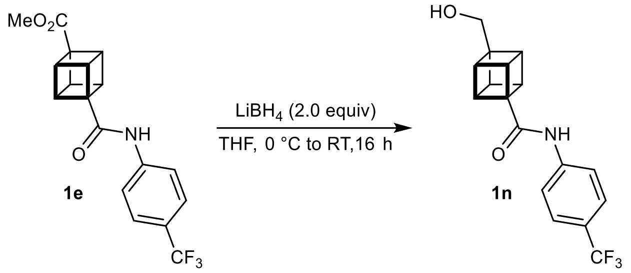

An oven-dried flask equipped with a stirrer bar was charged with cubane **1e** (500 mg, 1.43 mmol), which was sealed with a septum and purged with argon for 30 min. Anhydrous THF (8 mL) was added and the solution was cooled to 0 °C. $LiBH_4$ (4.0 M in THF, 0.72 mL, 2.88 mmol) was dropwise and the reaction was stirred at room temperature for 16 h. The reaction was quenched carefully with H_2O (10 mL), extracted with EtOAc (3 × 10 mL), and the combined organic layers were washed with brine (10 mL), dried ($MgSO_4$), filtered, and concentrated *in vacuo*. Purification of the residue by column chromatography (60% EtOAc/pentane to 80% EtOAc/pentane) gave *cubane* **1n** as an off-white solid (406 mg, 89%). R_f = 0.24 (60% EtOAc/petrol); m.p. 235–236 °C (Et_2O); IR 3342 (OH),

2972, 1648 (C=O), 1598, 1508, 1325, 1211, 1066, 999, 830 cm^{-1}; ^{1}H NMR (400 MHz, DMSO-D$_6$) δ 9.92 (1H, s, N**H**), 7.91 (2H, d, *J* = 8.4 Hz, Ar**H**), 7.67 (2H, d, *J* = 8.4 Hz, Ar**H**), 4.54 (1H, t, *J* = 5.5 Hz, O**H**), 4.18–4.13 (3H, m, cubyl C**H**), 3.83–3.79 (3H, m, cubyl C**H**), 3.55 (2H, d, *J* = 5.5 Hz, C**H**$_2$OH); ^{13}C NMR (101 MHz, DMSO-D$_6$) δ 170.8 (C), 142.8 (C), 125.9 (q, $J_{C\text{-}F}$ = 3.9 Hz, 2 × CH), 124.4 (q, $J_{C\text{-}F}$ = 271.1 Hz, C), 123.1 (q, $J_{C\text{-}F}$ = 31.9 Hz, C), 119.4 (2 × CH), 61.4 (CH$_2$), 58.6 (C), 58.3 (C), 45.8 (3 × CH), 43.7 (3 × CH); ^{19}F NMR (376 MHz, CDCl$_3$) δ –60.3 (s, 3 × F); HRMS (ESI) Exact mass calculated for $[C_{17}H_{14}F_3NNaO_2]^+$ [M+Na]$^+$: 344.0869, found 344.0871.

4-(Hydroxymethyl)-*N*-(4-methoxyphenyl)cubane-1-carboxamide (1o)

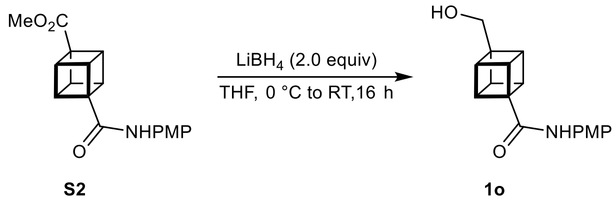

An oven-dried flask equipped with a stirrer bar was charged with cubane **S2** (311 mg, 1.00 mmol), which was sealed with a septum and purged with argon for 30 min. Anhydrous THF (5 mL) was added and the solution was cooled to 0 °C. LiBH$_4$ (2.0 M in THF, 0.75 mL, 1.50 mmol) was dropwise and the reaction was stirred at room temperature for 16 h. The reaction was quenched carefully with H$_2$O (5 mL), extracted with EtOAc (3 × 10 mL), and the combined organic layers were washed with brine (15 mL), dried (MgSO$_4$), filtered, and concentrated *in vacuo*. Purification of the residue by column chromatography (100% EtOAc) gave *cubane* **1o** as a white solid. R$_f$ = 0.31 (100% EtOAc) m.p. 180–182 °C (CH$_2$Cl$_2$); IR 3343 (OH), 2923, 2852, 1634 (C=O), 1525, 1465, 1333, 1237, 1029, 826 cm^{-1}; ^{1}H NMR (500 MHz, CDCl$_3$) δ 7.53–7.40 (2H, m, Ar**H**), 7.05 (1H, s, N**H**), 6.87 (2H, d, *J* = 8.9 Hz, Ar**H**), 4.22–4.20 (3H, m, cubyl C**H**), 3.94–3.92 (3H, m, cubyl C**H**), 3.81 (3H, br s, C**H**$_2$ and O**H**), 3.79 (3H, s, C**H**$_3$); ^{13}C NMR (126 MHz, CDCl$_3$) δ 170.9 (C), 156.5 (C), 130.9 (C), 121.6 (2 × CH), 114.3 (2 × CH), 63.4 (CH$_2$), 59.2 (C), 59.1 (C), 55.6 (CH$_3$), 46.6 (3 × CH), 44.2 (3 × CH); HRMS (ESI) Exact mass calculated for $[C_{17}H_{18}NO_3]^+$ [M+H]$^+$: 284.1281, found 284.1279.

Azocan-1-yl-[4-(hydroxymethyl)cuban-1-yl]methanone (1p)

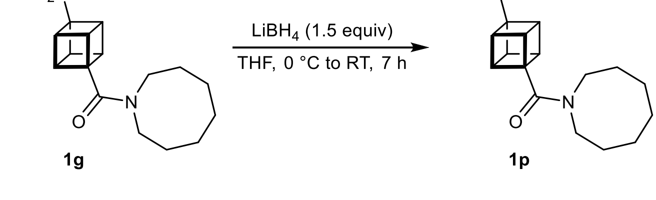

An oven-dried microwave vial equipped with a stirrer bar was charged with cubane **1g** (480 mg, 1.59 mmol), which was sealed with a septum and purged with argon for 30 min. Anhydrous THF (8 mL) was added and the solution was cooled to 0 °C. LiBH$_4$ (2.0 M in THF, 1.2 mL, 2.40 mmol) was

dropwise and the reaction was stirred at room temperature for 7 h. The reaction was quenched carefully with H_2O (8 mL), extracted with EtOAc (3 × 10 mL), and the combined organic layers were washed with brine (25 mL), dried ($MgSO_4$), filtered, and concentrated *in vacuo*. Purification of the residue by column chromatography (100% EtOAc) gave *cubane* **1p** as a white solid (302 mg, 69%). R_f = 0.25 (100% EtOAc), m.p. 172–173 °C (MeOH); IR 3370 (OH), 2975, 2898, 2844, 1602 (C=O), 1469, 1420, 1032, 627, 535 cm^{-1}; ^{1}H NMR (500 MHz, $CDCl_3$) δ 4.17–4.10 (3H, m, cubyl C**H**), 3.89–3.82 (3H, m, cubyl C**H**), 3.76 (2H, br s, C**H**$_2$OH), 3.40 (2H, t, *J* = 6.1 Hz, NC**H**$_2$), 3.20 (2H, t, *J* = 5.7 Hz, NC**H**$_2$), 1.83 (1H, br s, O**H**), 1.78-1.68 (4H, m, azocanyl C**H**$_2$), 1.60 (2H, br p, *J* = 5.7 Hz, azocanyl C**H**$_2$), 1.56-1.44 (4H, m, azocanyl C**H**$_2$); ^{13}C NMR (126 MHz, $CDCl_3$) δ 171.2 (C), 63.4 (CH_2), 59.4 (C), 57.8 (C), 48.6 (CH_2), 46.4 (3 × CH), 46.0 (CH_2), 44.0 (3 × CH), 26.72 (CH_2), 26.71 (CH_2), 26.5 (CH_2), 25.9 (CH_2), 23.7 (CH_2); HRMS (ESI) Exact mass calculated for $[C_{17}H_{24}NO_2]^+$ $[M+H]^+$: 274.1802, found 274.1798

Methyl 4-(1,2,3,4-tetrahydroquinoline-1-carbonyl)cubane-1-carboxylate (S6)

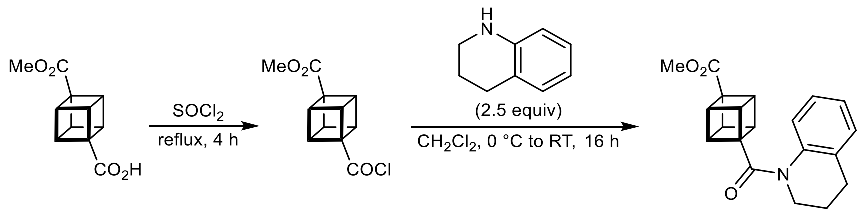

An oven-dried microwave vial equipped with a stirrer bar was charged with cubane **S1**[10] (500 mg, 2.42 mmol). The vial was sealed with a Teflon-lined cap and purged with argon for 5 min. $SOCl_2$ (5 mL) was then added and the resulting solution was heated at reflux for 4 h. The solution was then cooled to room temperature and excess $SOCl_2$ was removed under reduced pressure to afford the crude acid chloride. The crude residue was dissolved in anhydrous CH_2Cl_2 (12.5 mL) and cooled to 0 °C in an ice bath. 1,2,3,4-tetrahydroquinoline (806 mg, 6.05 mmol) was added dropwise. The reaction was stirred for 16 h (while allowing to warm slowly to room temperature), diluted with CH_2Cl_2 (12.5 mL), and quenched with saturated aqueous NH_4Cl solution (25 mL). The organic layer was washed with 1 M aqueous HCl solution (25 mL) and brine (25 mL), dried ($MgSO_4$), filtered, and concentrated *in vacuo* to give *cubane* **S6** as a white solid (617 mg, 79%), which was used without further purification. R_f = 0.35 (40% EtOAc/petrol); m.p. 173–174 °C (Et_2O); IR 2981, 1709 (C=O), 1628 (C=O), 1387, 1321, 1218, 1205, 1087, 761, 437 cm^{-1}; ^{1}H NMR (400 MHz, DMSO-D$_6$, 100 °C) δ 7.36–7.31 (1H, m, Ar**H**), 7.21–7.15 (2H, m, Ar**H**), 7.13–7.07 (1H, m, Ar**H**), 4.11–4.05 (3H, m, cubyl C**H**), 4.05–3.98 (3H, m, cubyl C**H**), 3.64 (3H, s, C**H**$_3$), 3.61 (2H, t, *J* = 6.4 Hz, NC**H**$_2$), 2.73 (2H, t, *J* = 6.7 Hz, ArC**H**$_2$), 1.93 (2H, p, *J* = 6.6 Hz, CH$_2$C**H**$_2$CH$_2$); ^{13}C NMR (101 MHz, DMSO-D$_6$, 100 °C) δ 170.5 (C), 168.9 (C), 138.5 (C), 131.0 (C), 127.7 (CH), 125.5 (CH), 124.1 (CH), 122.0

(CH), 58.4 (C), 53.7 (C), 50.5 (CH_3), 46.7 (3 × CH), 45.3 (3 × CH), 42.6 (CH_2), 25.6 (CH_2), 23.0 (CH_2); HRMS (ESI) Exact mass calculated for $[C_{20}H_{19}NNaO_3]^+$ $[M+Na]^+$: 344.1257, found 344.1259.

3,4-Dihydroquinolin-1(2*H*)-yl-4-(hydroxymethyl)cuban-1-yl)methanone (1q)

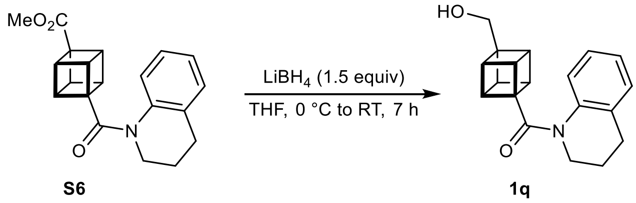

An oven-dried microwave vial equipped with a stirrer bar was charged with cubane **S6** (321 mg, 1.00 mmol), which was sealed with a septum and purged with argon for 30 min. Anhydrous THF (5 mL) was added and the solution was cooled to 0 °C. $LiBH_4$ (4.0 M in THF, 0.38 mL, 1.52 mmol) was added dropwise and the reaction was stirred at room temperature for 16 h. The reaction was quenched carefully with H_2O (5 mL), extracted with EtOAc (3 × 10 mL), and the combined organic layers were washed with brine (15 mL), dried ($MgSO_4$), filtered, and concentrated *in vacuo*. Purification of the residue by column chromatography (100% EtOAc) gave *cubane* **1q** as a white solid (213 mg, 73%). R_f = 0.58 (100% EtOAc); m.p. 171– 172 °C (Et_2O); IR 3394 (OH), 3004, 1619 (C=O), 1595, 1491, 1394, 1058, 1031, 774, 616 cm^{-1}; ^1H NMR (400 MHz, DMSO-D_6, 100 °C) δ 7.43–7.36 (1H, m, Ar**H**), 7.20–7.12 (2H, m, Ar**H**), 7.11–7.04 (1H, m, Ar**H**), 4.10 (1H, t, J = 5.4 Hz, O**H**), 3.95–3.88 (3H, m, cubyl C**H**), 3.76–3.69 (3H, m, cubyl C**H**), 3.60 (2H, t, J = 6.3 Hz, NC**H**$_2$), 3.54 (2H, d, J = 5.4 Hz, C**H**$_2$OH), 2.73 (2H, t, J = 6.7 Hz, ArC**H**$_2$), 1.93 (2H, p, J = 6.6 Hz, CH_2C**H**$_2$$CH_2$); ^{13}C NMR (400 MHz, DMSO-D_6, 100 °C) δ 169.7 (C), 138.6 (C), 130.6 (C), 127.7 (CH), 125.3 (CH), 123.8 (CH), 122.3 (CH), 61.2 (CH_2), 58.9 (C), 57.2 (C), 45.9 (3 × CH), 42.8 (3 × CH), 42.5 (CH_2), 25.7 (CH_2), 23.1 (CH_2); HRMS (ESI) Exact mass calculated for $[C_{19}H_{19}NNaO_2]^+$ $[M+Na]^+$: 316.1308, found 316.1313.

Methyl 4-[(1,3-dioxoisoindolin-2-yl)methyl]cubane-1-carboxylate (1t)

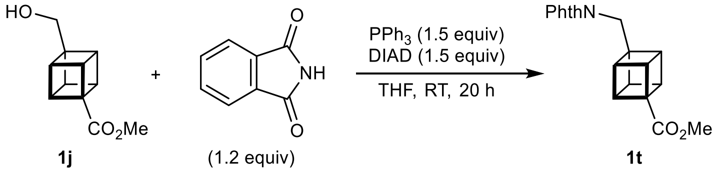

Cubane **1j** (200 mg, 1.04 mmol), triphenylphosphine (409 mg, 1.56 mmol) and phthalimide (230 mg, 1.56 mmol) were added to a microwave vial equipped with a stirrer bar, which was sealed with a Teflon-lined cap and purged with nitrogen for 15 min. Anhydrous THF (10 mL) and DIAD (316 mg, 308 µL, 1.56 mmol) were added and the resulting mixture was stirred at room temperature for 20 h. The reaction was concentrated *in vacuo* and the residue was purified by column chromatography

(100% cyclohexane to 70% EtOAc/cyclohexane) to give *cubane* **1t** as a white solid (276 mg, 83%). R_f = 0.43 (30% EtOAc/cyclohexane); m.p. 166–167 °C (Et_2O); IR 2966, 1771, 1726 (C=O), 1706 (C=O), 1442, 1422, 1391, 1350, 1327, 1215 cm^{-1}; ^{1}H NMR (400 MHz, $CDCl_3$) δ 7.88–7.83 (2H, m, Ar**H**), 7.76–7.71 (2H, m, Ar**H**), 4.14–4.06 (3H, m, cubyl C**H**), 3.95 (2H, C**H**$_2$), 4.14–4.06 (3H, m, cubyl C**H**), 3.69 (3H, s, C**H**$_3$); ^{13}C NMR (75 MHz, $CDCl_3$) δ 172.7 (C), 168.9 (2 × C), 134.1 (2 × CH), 132.2 (2 × C), 132.2 (2 × CH), 57.7 (C), 56.5 (C), 51.6 (CH_3), 46.3 (3 × CH), 46.0 (3 × CH), 40.1 (CH_2); HRMS (ESI) Exact mass calculated for $[C_{19}H_{15}NO_4Na]^+$ $[M+Na]^+$: 344.0893, found 344.0892.

Azocan-1-yl[4-(2-hydroxypropan-2-yl)cuban-1-yl]methanone (1v)

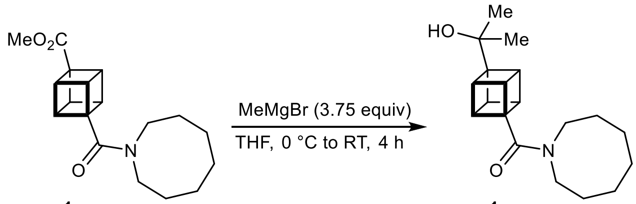

Cubane **1g** (300 mg, 1.00 mmol) was added to a flame-dried round-bottom flask, which was sealed and purged with argon for 15 min. Anhydrous THF (15 mL) was added and the resulting solution was cooled to 0 °C in an ice bath. MeMgBr (3 M in Et_2O, 1.25 mL, 3.75 mmol) was added dropwise, and the reaction was warmed to room temperature and stirred for 4 h. The reaction was quenched carefully with saturated aqueous NH_4Cl solution (10 mL) and diluted with EtOAc (15 mL). The organic layer was washed with brine (15 mL), dried ($MgSO_4$), filtered, and concentrated *in vacuo*. Purification of the residue by column chromatography (40% EtOAc/cyclohexane to 80% EtOAc/cyclohexane) gave *cubane* **1v** as a white solid (170 mg, 57%). R_f = 0.26 (70% EtOAc/cyclohexane); m.p. 176–177 °C (EtOAc/cyclohexane); IR 3347 (OH), 2968, 2916, 1593 (C=O), 1426, 1153, 952, 925,725, 643 cm^{-1}; ^{1}H NMR (500 MHz, $CDCl_3$) δ 4.17–4.00 (3H, m, cubyl C**H**), 3.92–3.78 (3H, m, cubyl C**H**), 3.48–3.33 (2H, m, C**H**$_2$N), 3.26–3.13 (2H, m, C**H**$_2$N), 1.78–1.71 (4H, m, azocanyl C**H**$_2$), 1.61 (2H, m, azocanyl C**H**$_2$), 1.58–1.44 (4H, m, azocanyl C**H**$_2$), 1.16 (6H, s, C(C**H**$_3$)$_2$); ^{13}C NMR (126 MHz, $CDCl_3$) δ 171.1 (C), 70.3 (C), 64.2 (C), 59.6 (C), 48.6 (CH_2), 46.0 (CH_2), 45.3 (3 × CH), 43.3 (3 × CH), 26.7 (2 × CH_2), 26.6 (CH_2), 25.9 (CH_2), 23.7 (CH_2), 23.0 (2 × CH_3); HRMS (ESI) Exact mass calculated for $[C_{19}H_{28}NO_2]^+$ $[M+H]^+$: 302.2120, found 302.2152. *A trimeric species was identified as the major peak.* Exact mass calculated for $[C_{57}H_{81}N_3O_6Na]^+$ $[M_3+Na]^+$: 926.6023, found 926.5989.

Slow evaporation of a solution of **1v** in CH_2Cl_2 gave crystals suitable for X-ray crystallography:

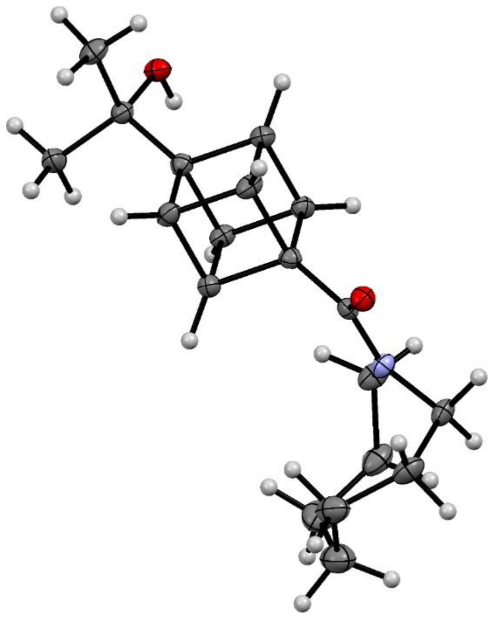

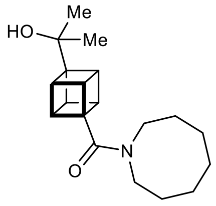

1v
CCDC 2248870

ORTEP with ellipsoid probabilities at 50%

Methyl 4-{[(2,2,2-trichloroethoxy)carbonyl]amino}cubane-1-carboxylate (1w)

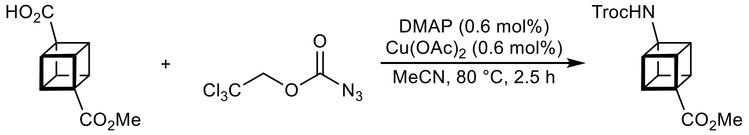

Using conditions adapted from the literature,[17] cuneane **S1**[10] (206 mg, 1.00 mmol), $Cu(OAc)_2$ (1.0 mg, 0.006 mmol) and DMAP (0.8 mg, 0.006 mmol) were added to an oven-dried microwave vial equipped with a stirrer bar. Anhydrous MeCN (10 mL) was added followed by 2,2,2-trichloroethoxycarbonyl azide[17] (262 mg, 1.20 mmol). Additional MeCN (2 mL) was used to wash the inner walls of the vial, which was then sealed and heated to 80 °C for 2.5 h. The reaction was cooled to room temperature and concentrated *in vacuo*. Purification of the residue by column chromatography (50% EtOAc/pentane) gave *cuneane* **1w** as a white solid as a *ca.* 1:1 mixture of rotamers (192 mg, 55%). R_f = 0.19 (20% EtOAc/petrol; m.p. 130–133 °C ($CDCl_3$); IR 3269 (NH), 2998, 2155, 1715 (C=O), 1386, 1304, 1206, 1085, 703, 568 cm^{-1}; ^1H NMR (500 MHz, $CDCl_3$) δ 5.57 (1H, br s, N**H**), 5.30 (1H, br s, N**H**), 4.76 (3H, br s, C**H**$_2$), 4.72 (3H, br s, C**H**$_2$), 4.15–4.13 (12H, m, cubyl C**H** of both rotamers), 3.71 (6H, s, C**H**$_3$ of both rotamers); ^{13}C NMR (126 MHz, $CDCl_3$) δ 172.6 (C of both rotamers), 153.8 (C), 152.5 (C), 95.5 (C), 95.3 (C), 75.2 (CH_2), 74.6 (CH_2), 66.4 (C), 66.1 (C), 56.2 (C), 56.1 (C), 51.7 (CH_3 of both rotamers), 50.9 (3 × CH), 50.2 (3 × CH), 44.9 (3 × CH), 44.6 (3 × CH); HRMS (ESI) Exact mass calculated for $[C_{13}H_{11}Cl_3NO_4]^-$ $[M-H]^-$ 349.9759, found 349.9758.

Methyl-4-(bromomethyl)cubane-1-carboxylate (1x)

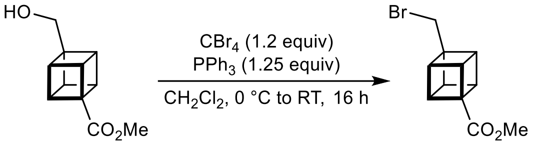

1j **1x**

Cubane **1j**[12] (192 mg, 1.00 mmol) was added to a flask equipped with a stirrer bar, which was sealed with a septum and purged with argon for 30 min. Anhydrous CH_2Cl_2 (2 mL) was added, followed by a solution of CBr_4 (398 mg, 1.20 mmol) in CH_2Cl_2 (1 mL). The mixture was cooled to 0 ºC, a solution of PPh_3 (328 mg, 1.25 mmol) in CH_2Cl_2 (1 mL) was added dropwise, and the resulting solution was stirred at room temperature for 16 h. EtOH (10 ml) was added and the reaction was stirred for an additional 30 min. The solvent was removed under reduced pressure and the residue was purified by column chromatography (10% EtOAc/pentane) to give cubane **1x** (215 mg, 85%) as a colorless oil, which displayed spectroscopic data consistent with those reported previously.[18] R_f = 0.38 (20% EtOAc/petrol); IR 2989, 1721 (C=O), 1434, 1328, 1313, 1209, 1087, 884, 840, 602 cm^{-1}; ^{1}H NMR (500 MHz, $CDCl_3$) δ 4.12–4.08 (3H, m, cubyl C**H**), 3.86–3.83 (3H, m, cubyl C**H**), 3.70 (3H, s, C**H**$_3$), 3.65 (2H, s, C**H**$_2$Br); ^{13}C NMR (126 MHz, $CDCl_3$) δ 172.5 (C), 58.1 (C), 56.6 (C), 51.7 (CH_3), 46.2 (3 × CH), 45.5 (3 × CH), 35.9 (CH_2); HRMS (ESI) Exact mass calculated for $[C_{11}H_{11}BrNaO_2]^+$ $[M+Na]^+$: 276.9835, found 276.9833.

Note: In the NMR spectra of **1x**, signals consistent with the presence of trace quantities (≤5%) of homocubane **6** were observed (for the NMR data of **6**, see page 28).

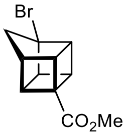

6

3. Silver(I)-Catalyzed Rearrangements of Cubanes

General Procedure A

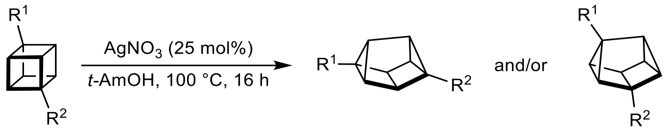

An oven-dried microwave vial equipped with a stirrer bar was charged with the appropriate cubane (0.20 mmol) and $AgNO_3$ (8.5 mg, 0.05 mmol). The vial was sealed with a septum-lined cap and *t*-AmylOH (2 mL) (undried, obtained from commercial vendors used without further purification) was added. The vial was wrapped in foil, and the reaction was stirred at 100 °C for 16 h, cooled to room temperature, and concentrated *in vacuo.* Purification of the residue by column chromatography gave the cuneane product(s).

General Procedure B

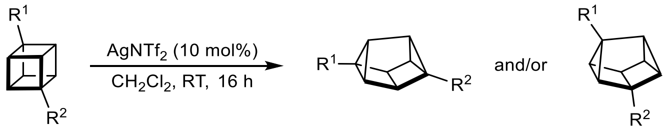

An oven-dried microwave vial equipped with a stirrer bar was charged with the appropriate cubane (0.20 mmol) and $AgNTf_2$ (7.8 mg, 0.02 mmol). The vial was sealed with a septum-lined cap and CH_2Cl_2 (2 mL) (undried, obtained from commercial vendors used without further purification) was added. The vial was wrapped in foil, and the reaction was stirred at room temperature for 16 h and concentrated *in vacuo.* Purification of the residue by column chromatography gave the cuneane product(s).

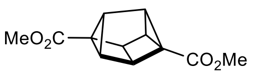

(±)-Dimethyl cuneane-2,6-dicarboxylate (2a). A slight modification of General Procedure A was followed using cubane **1a** (44 mg, 0.20 mmol) in that the reaction time was 20 h. The residue obtained after the solvent was removed was redissolved in CH_2Cl_2 and filtered through a plug of silica gel using CH_2Cl_2 as eluent to give cuneane **2a** as an off-white solid (41 mg, 93%), which displayed spectroscopic data consistent with those reported previously.[5] ^{1}H NMR (400 MHz, $CDCl_3$) δ 3.62 (6H, s, 2 × C**H**$_3$), 3.12–3.09 (4H, m, cuneanyl C**H**), 3.06–3.04 (2H, m, cuneanyl C**H**); ^{13}C NMR (126 MHz, $CDCl_3$) δ 170.8 (2 × C), 51.7 (2 × CH_3), 43.74 (2 × C), 43.71 (2 × CH), 40.7 (2 × CH), 38.6 (2 × CH).

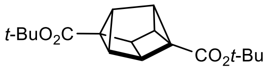

(±)-Di-*tert*-butyl cuneane-2,6-dicarboxylate (2b). General Procedure A was followed using cubane **1b** (61 mg, 0.20 mmol). Purification by column

chromatography (20% EtOAc/cyclohexane) gave *cuneane* **2b** as a white solid (48 mg, 80%). R_f = 0.23 (5% EtOAc/petrol); m.p. 105–106 °C (Et_2O); IR 2980, 1700 (C=O), 1382, 1366, 1286, 1227, 1156, 1095, 841, 746 cm^{-1}; ^1H NMR (500 MHz, $CDCl_3$) δ 3.02–2.99 (4H, m, cuneanyl C**H**), 2.98–2.95 (2H, m, cuneanyl C**H**), 1.40 (18H, s, 2 × C(C**H**$_3$)$_3$); ^{13}C NMR (126 MHz, $CDCl_3$) 170.1 (2 × C), 80.4 (2 × C), 44.7 (2 × C), 43.4 (2 × CH), 40.2 (2 × CH), 38.4 (2 × CH), 28.3 (6 × CH_3); HRMS (ESI) Exact mass calculated for $[C_{18}H_{24}NaO_4]^+$ $[M+Na]^+$ 327.1567, found 327.1573.

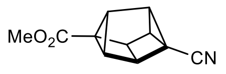

(±)-Methyl-6-cyanocuneane-2-carboxylate (2c). General Procedure A was followed using cubane **1c** (37.4 mg, 0.20 mmol). Purification by column chromatography (30% EtOAc/pentane) gave *cuneane* **2c** as a yellow oil (29.0 mg, 77%). R_f = 0.67 (50% EtOAc/petrol); IR 2953, 2224 (C≡N), 1717 (C=O), 1439, 1381, 1346, 1266, 1104, 853, 715 cm^{-1}; ^1H NMR (400 MHz, $CDCl_3$) δ 3.61 (3H, s, C**H**$_3$), 3.24–3.19 (2H, m, cuneanyl C**H**), 3.13–3.05 (4H, m, cuneanyl C**H**); ^{13}C NMR (101 MHz, $CDCl_3$) δ 169.6 (C), 117.8 (C), 51.8 (CH_3), 44.6 (C), 44.3 (CH), 41.8 (CH), 41.5 (CH), 40.7 (C), 39.4 (CH), 39.2 (CH), 26.1 (CH); HRMS (ESI) Exact mass calculated for $[C_{11}H_9NNaO_2]^+$ $[M+Na]^+$: 210.0525, found 210.0532.

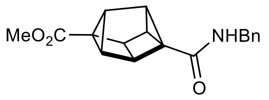

(±)-Methyl 6-(benzylcarbamoyl)cuneane-2-carboxylate (2d). General Procedure A was followed using cubane **1d** (59.0 mg, 0.20 mmol). Purification by column chromatography (100% EtOAc) gave *cuneane* **2d** as a beige oil (21.1 mg, 36%). **Note**: This compound was observed to be moderately unstable to silica gel. R_f = 0.26 (50% EtOAc/petrol); IR 3308 (NH), 1715 (C=O), 1632 (C=O), 1530, 1388, 1284, 1201, 1103, 726, 698 cm^{-1}; ^1H NMR (500 MHz, $CDCl_3$) δ 7.36–7.24 (5H, m, Ar**H**), 5.66 (1H, br s, N**H**), 4.43 (1H, dd, *J* = 14.6, 5.9 Hz, C**H**$_2$), 4.37 (1H, dd, *J* = 14.6, 5.7 Hz, C**H**$_2$), 3.62 (1H, s, C**H**$_3$), 3.14-3.07 (2H, m, cuneanyl C**H**), 3.06–2.98 (3H, m, cuneanyl C**H**), 2.95–2.91 (1H, m, cuneanyl C**H**); ^{13}C NMR (126 MHz, $CDCl_3$) δ 170.8 (C), 169.3 (C), 138.3 (C), 128.9 (2 × CH), 128.1 (2 × CH), 127.7 (CH), 51.7 (CH_3), 45.7 (C), 43.8 (C), 43.7 (CH_2), 43.4 (CH), 42.0 (CH), 40.8 (CH), 38.6 (CH), 38.7 (CH), 38.6 (CH); HRMS (ESI) Exact mass calculated for $[C_{18}H_{17}NNaO_3]^+$ $[M+Na]^+$: 318.1101, found 318.1093.

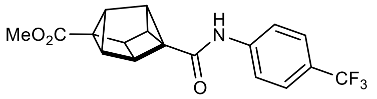

(±)-Methyl 6-{[4-(trifluoromethyl)phenyl]carbomoylcuneane-2-carboxylate (2e). General Procedure A was followed using cubane **1e** (69.9 mg, 0.20 mmol). Purification by column chromatography (100% EtOAc) gave *cuneane* **2e** as a beige solid (50.1 mg, 72%). R_f = 0.51 (50% EtOAc/pentane); m.p. 194–195 °C ($CDCl_3$); IR 3308 (NH), 1701 (C=O), 1654 (C=O), 1525, 1315, 1112, 1067, 909, 841, 731 cm^{-1}; ^1H NMR (500 MHz, $CDCl_3$) δ 7.58 (2H, d, *J* = 8.6 Hz, Ar**H**), 7.53 (2H, d, *J* = 8.6 Hz, Ar**H**), 7.39 (1H, br s, N**H**), 3.64 (3H, s, C**H**$_3$), 3.17–3.12 (4H, m, cuneanyl C**H**), 3.10–3.08 (2H, m, cuneanyl C**H**); ^{13}C NMR (126

MHz, $CDCl_3$) δ 170.6 (C), 168.1 (C), 140.8 (C), 126.3 (q, J_{C-F} = 3.8 Hz, C), 126.1 (q, J_{C-F} = 32.8 Hz, 2 × CH), 124.2 (q, J_{C-F} = 271.5 Hz, C), 119.5 (2 × CH), 51.8 (CH_3), 46.4 (C), 43.9 (C), 43.4 (CH), 43.1 (CH), 40.7 (CH), 38.9 (2 × CH), 38.6 (CH); ^{19}F NMR (376 MHz, $CDCl_3$) δ –62.1 (s, 3 × F); HRMS (ESI) Exact mass calculated for $[C_{18}H_{13}F_3NNaO_3]^+$ $[M+Na]^+$: 372.0818, found 372.0814.

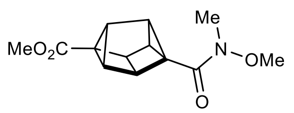

(±)-Methyl-6-[methoxy(methyl)carbamoyl]cuneane-2-carboxylate (2f). General Procedure A was followed using cubane **1f** (49.9 mg, 0.20 mmol). Purification by column chromatography (80% EtOAc/pentane) gave *cuneane* **2f** as a yellow oil (44.7 mg, 90%). R_f = 0.41 (80% EtOAc/petrol); IR 2952, 1719 (C=O), 1637 (C=O), 1439, 1385, 1333, 1265, 1183, 858, 721 cm^{-1}; ^{1}H NMR (400 MHz, $CDCl_3$) δ 3.63 (3H, s, OC**H**$_3$), 3.60 (3H, s, OC**H**$_3$), 3.17–3.13 (4H, m, NC**H**$_3$ and cuneanyl C**H**), 3.12–3.01 (5H, m, cuneanyl C**H**); ^{13}C NMR (101 MHz, $CDCl_3$) δ 170.8 (C), 170.4 (C), 61.6 (CH_3), 51.7 (CH_3), 45.4 (C), 44.3 (C), 44.1 (CH), 41.8 (CH), 41.2 (CH), 41.1 (CH), 40.8 (CH), 38.4 (CH), 32.9 (CH_3); HRMS (ESI) Exact mass calculated for $[C_{13}H_{16}NO_4]^+$ $[M+H]^+$: 250.1074, found 250.1075.

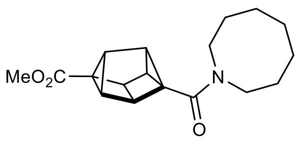

(±)-Methyl 6-(azocane-1-carbonyl)cuneane-2-carboxylate (2g). General Procedure A was followed using cubane **1g** (60.3 mg, 0.20 mmol). Purification by column chromatography (100% EtOAc) gave *cuneane* **2g** as a colorless oil (49.5 mg, 82%). R_f = 0.36 (50% EtOAc/pentane); IR 2924, 1717 (C=O), 1616 (C=O), 1437, 1425, 1262, 1200, 1181, 1098, 920, 727 cm^{-1}; ^{1}H NMR (500 MHz, $CDCl_3$) δ 3.60 (3H, s, C**H**$_3$), 3.42–3.30 (2H, m, NC**H**$_2$), 3.20-3.10 (1H, m, cuneanyl C**H**), 3.13–3.06 (2H, m, NC**H**$_2$), 3.06–3.02 (2H, m, cuneanyl C**H**), 3.00–2.97 (1H, m, cuneanyl C**H**), 2.96–2.93 (1H, m, cuneanyl C**H**), 2.76–2.72 (1H, m, cuneanyl C**H**), 1.74–1.41 (10H, m, (C**H**$_2$)$_5$CH$_2$N); ^{13}C NMR (126 MHz, $CDCl_3$) δ 170.6 (C), 168.7 (C), 51.6 (CH_3), 48.9 (CH_2), 47.0 (CH_2), 46.1 (C), 44.2 (C), 43.2 (CH), 41.8 (CH), 40.9 (CH), 39.1 (CH), 38.7 (CH), 37.7 (CH), 26.9 (CH_2), 26.7 (CH_2), 26.2 (CH_2), 26.0 (CH_2), 24.3 (CH_2); HRMS (ESI) Exact mass calculated for $[C_{18}H_{23}NNaO_3]^+$ $[M+Na]^+$: 324.1570, found 324.1571.

(±)-Methyl 6-(2-oxa-6-azaspiro[3.3]heptane-6-carbonyl)cuneane-2-carboxylate (**2h**)

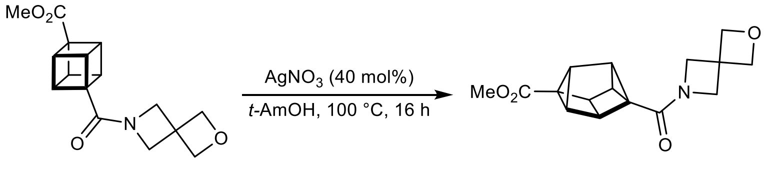

1h **2h**

An oven-dried microwave vial equipped with a stirrer bar was charged with cubane **1h** (57.5 mg, 0.20 mmol) and $AgNO_3$ (13.6 mg, 0.08 mmol). The vial was sealed with a septum-lined cap and *t*-AmylOH (2 mL) (undried, obtained from commercial vendors used without further purification) was added.

The reaction was stirred at 100 °C for 16 h, cooled to room temperature, and concentrated *in vacuo*. The resulting black oil was dissolved in THF (3 mL), LiCl (0.5 M in THF, 2 mL) was added, and the mixture was stirred for 5 min. The precipitated silver salts were removed by filtration through celite (CH_2Cl_2), and the orange filtrate was concentrated *in vacuo*. The resulting orange oil was dissolved in CH_2Cl_2 (5 mL), washed with H_2O (15 mL), and the aqueous layer extracted with CH_2Cl_2 (2 × 5 mL). The combined organic layers were washed with brine (10 mL), dried (Na_2SO_4), filtered, and concentrated *in vacuo* to leave *cuneane* **2h** (51.4 mg, 90%) as an orange solid. **Note:** Cuneane **2h** was found to be unstable on silica gel. m.p. 159–162 °C ($CDCl_3$); IR 2948, 2875, 1709 (C=O), 1625 (C=O), 1430, 1337, 1282, 1203, 971, 745 cm^{-1}; ^{1}H NMR (400 MHz, $CDCl_3$) δ 4.77 (4H, br s, 2 × C**H**$_2$O), 4.24–4.09 (4H, m, 2 × C**H**$_2$N), 3.63 (3H, s, C**H**$_3$), 3.13–2.98 (6H, cuneanyl C**H**); ^{13}C NMR (101 MHz, $CDCl_3$) δ 170.6 (C), 170.2 (C), 80.9 (2 × CH_2), 60.0 (CH_2), 58.4 (CH_2), 51.7 (CH_3), 44.6 (C), 43.7 (C), 43.6 (CH), 41.5 (CH), 40.7 (CH), 40.3 (CH), 39.5 (CH), 38.7 (C), 38.6 (CH); HRMS (ESI) Exact mass calculated for $[C_{16}H_{18}NO_4]^+$ $[M+H]^+$: 288.1230, found 288.1222.

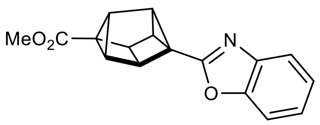

Methyl 6-(benzo[*d*]oxazol-2-yl)cuneane-2-carboxylate (2i). General Procedure A was followed using cubane **1i** (55.9 mg, 0.20 mmol). Purification by column chromatography (100% cyclohexane to 50% EtOAc/cyclohexane) gave *cuneane* **2i** as a white solid (51.3 mg, 92%); R_f = 0.40 (30% EtOAc/cyclohexane); m.p. 141–142 °C (Et_2O); IR 2950, 1717 (C=O), 1620, 1573, 1455, 1438, 1366, 1286, 1262, 1241 cm^{-1}; ^{1}H NMR (400 MHz, $CDCl_3$) δ 7.61–7.55 (1H, m, Ar**H**), 7.43–7.36 (1H, m, Ar**H**), 7.31–7.21 (2H, m, Ar**H**), 3.66 (3H, s, C**H**$_3$), 3.45–3.40 (1H, m, cuneanyl C**H**), 3.34–3.33 (1H, m, cuneanyl C**H**), 3.31–3.25 (2H, m, cuneanyl C**H**), 3.19–3.17 (1H, m, cuneanyl C**H**); ^{13}C NMR (75 MHz, $CDCl_3$) δ 170.6 (C), 164.0 (C), 150.6 (C), 141.7 (C), 124.4 (2 × CH), 119.2 (CH), 110.1 (CH), 51.7 (CH_3), 44.5 (C), 43.8 (CH), 43.4 (CH), 41.3 (CH), 40.8 (C), 39.9 (CH), 39.8 (CH), 39.4 (CH); HRMS (ESI) Exact mass calculated for $[C_{17}H_{14}NO_3]^+$ $[M+H]^+$: 280.0968, found 280.0968.

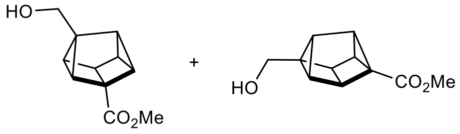

3b (tentative)

~24:1 ratio

(±)-Methyl 3-hydroxymethylcuneane-1-carboxylate (3b). General Procedure B was followed on a larger scale, using cubane **1j**[15] (200 mg, 1.04 mmol), $AgNTf_2$ (40 mg, 0.10 mmol), and CH_2Cl_2 (10 mL) for a reaction time of 10 min. Purification by column chromatography (50% EtOAc/pentane) gave a 24:1 inseparable mixture of *cuneane* **3b** and what we tentatively attribute to be the corresponding 2,6-disubstituted regioisomer as a colorless oil (190 mg, 95%). R_f = 0.36 (50% EtOAc/cyclohexane); IR 3391 (OH), 1707 (C=O), 1334, 1222, 1155, 1085, 1062, 990, 833, 731 cm^{-1}; HRMS (ES) Exact mass calculated for $[C_{11}H_{12}O_3]^+$ $[M+H]^+$: 193.0859, found 193.0859.

NMR data of major isomer ***3b***: ^{1}H NMR (500 MHz, $CDCl_3$) δ 4.04–3.99 (2H, m, C**H**$_2$OH), 3.74 (3H, s, C**H**$_3$), 3.05–3.01 (1H, m, cuneanyl C**H**), 2.49–2.43 (3H, m, cuneanyl C**H**), 2.35–2.28 (2H, m, cuneanyl C**H**); ^{13}C NMR (126 MHz, $CDCl_3$) δ 173.0 (C), 61.7 (CH_2), 54.0 (C), 52.0 (CH_3), 47.3 (C), 47.2 (CH), 36.6 (CH), 35.4 (CH), 32.2 (CH), 31.1 (CH), 30.8 (CH);

NMR data of 2,6-disubstituted isomer (tentative): ^{1}H NMR (500 MHz, $CDCl_3$) δ 3.61 (3H, C**H**$_3$), 2.96–2.94 (1H, m, cuneanyl C**H**), 2.82–2.79 (1H, m, cuneanyl C**H**); ^{13}C NMR (126 MHz, $CDCl_3$) δ 63.3 (CH_2), 51.6 (CH_3), 43.4 (CH), 41.1 (CH), 39.2 (CH), 39.0 (CH), 36.2 (CH), 34.3 (CH).

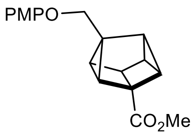

(±)-Methyl-3-[(4-methoxyphenoxy)methyl]cuneane-1-carboxylate (3c). General Procedure B was followed using cubane **1k** (59.6 mg, 0.20 mmol). Purification by preparative thin-layer chromatography (7.5% EtOAc/pentane) gave *cuneane* **3c** as a colorless oil (31.1 mg, 52%). R_f = 0.41 (20% EtOAc/petrol); IR 2950, 1725 (C=O), 1507, 1437, 1336, 1307, 1220, 1085, 825, 745 cm^{-1}; ^{1}H NMR (500 MHz, $CDCl_3$) δ 6.49–6.88 (2H, m, Ar**H**), 6.87–6.82 (2H, m, Ar**H**), 4.32 (2H, d, *J* = 10.3 Hz, C**H**$_a$H$_b$O), 4.28 (2H, d, *J* = 10.3 Hz, CH$_a$**H**$_b$O), 3.78 (3H, s, C**H**$_3$), 3.75 (3H, s, C**H**$_3$), 3.09-3.04 (1H, m, cuneanyl C**H**), 2.58–2.53 (1H, m, cuneanyl C**H**), 2.52–2.48 (2H, m, cuneanyl C**H**), 2.39–2.36(2H, m, cuneanyl C**H**); ^{13}C NMR (126 MHz, $CDCl_3$) δ 173.0 (C), 154.2 (C), 153.4 (C), 116.1 (2 × CH), 114.8 (2 × CH), 67.9 (CH_2), 55.9 (CH_3), 54.1 (C), 52.0 (CH_3), 47.3 (CH), 44.3 (C), 37.0 (CH), 35.9 (CH), 32.3 (CH), 31.5 (CH), 31.0 (CH); HRMS (ESI) Exact mass calculated for $[C_{18}H_{18}NaO_4]^+$ $[M+Na]^+$: 321.1097, found 321.1096.

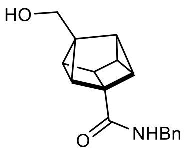

(±)-*N*-Benzyl-3-(hydroxymethyl)cuneane-1-carboxamide (3d). General Procedure B was followed using cubane **1l** (53.4 mg, 0.20 mmol). Purification by column chromatography (70% EtOAc/pentane to 100% EtOAc) gave *cuneane* **3d** as a colorless oil (45.0 mg, 84%). R_f = 0.29 (80% EtOAc/pentane); IR 3305 (OH), 3032, 1632 (C=O), 1524, 1336, 1231, 1019, 832, 747, 697 cm^{-1}; ^{1}H NMR (500 MHz, $CDCl_3$) δ 7.37–7.22 (5H, m, Ar**H**), 6.09 (1H, br s, N**H**), 4.48 (2H, d, *J* = 5.8 Hz, NC**H**$_2$), 4.02 (1H, d, *J* = 14.6 Hz, C**H**$_a$H$_b$OH), 3.86 (1H, d, *J* = 14.6 Hz, CH$_a$**H**$_b$OH), 3.00–2.94 (1H, m, cuneanyl C**H**), 2.50–2.43 (1H, m, cuneanyl C**H**), 2.43–2.35 (2H, m, cuneanyl C**H**), 2.34–2.27 (2H, m, cuneanyl C**H**), 2.24 (1H, br s, O**H**); ^{13}C NMR (126 MHz, $CDCl_3$) δ 171.9 (C), 138.4 (C), 128.8 (2 × CH), 127.9 (2 × CH), 127.7 (CH), 61.5 (CH_2), 56.2 (C), 47.7 (C), 47.1 (CH), 43.6 (CH_2), 36.9 (CH), 35.6 (CH), 32.4 (CH), 31.6 (CH), 31.0 (CH); HRMS (ESI) Exact mass calculated for $[C_{17}H_{17}NNaO_2]^+$ $[M+Na]^+$: 290.1151, found 290.1152.

(±)-3-(Hydroxymethyl)-*N*-(2,4,4-trimethylpentan-2-yl)cuneane-1-carboxamide (3e)

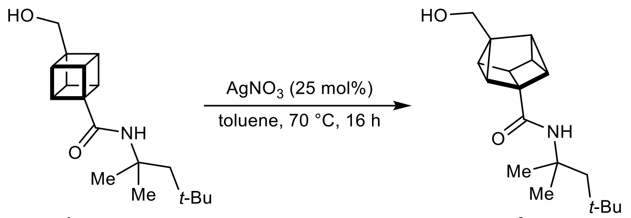

An oven-dried microwave vial equipped with a stirrer bar was charged with cubane **1m** (58 mg, 0.20 mmol) and $AgNO_3$ (8.5 mg, 0.05 mmol). The vial was sealed with a septum-lined cap and toluene (1 mL) (undried, obtained from commercial vendors used without further purification) was added. The reaction was stirred at 70 °C for 16 h, cooled to room temperature, and concentrated *in vacuo*. Purification of the residue by column chromatography (50% EtOAc/cyclohexane to 80% EtOAc/cyclohexane) gave *cubane* **3e** as a pale yellow oil (44 mg, 76%). R_f = 0.48 (100% EtOAc); IR 3326 (OH), 2951, 2870, 1642 (C=O), 1519, 1479, 1365, 1250, 1225, 1024 cm^{-1}; ^{1}H NMR (500 MHz, $CDCl_3$) δ 5.51 (1H, s, N**H**), 4.02 (1H, d, *J* = 11.8 Hz, C**H**$_a$H$_b$OH), 3.98 (1H, d, *J* = 11.8 Hz, CH$_a$**H**$_b$OH), 2.93–2.90 (1H, m, cuneanyl C**H**), 2.46–2.42 (1H, m, cuneanyl C**H**), 2.35–2.25 (4H, m, cuneanyl C**H**),1.74 (2H, s, C**H**$_2$), 1.42 (6H, s, C(C**H**$_3$)$_2$), 1.01 (9H, s, C(C**H**$_3$)$_3$); ^{13}C NMR (126 MHz, $CDCl_3$) δ 170.9 (C), 61.7 (CH_2), 57.2 (C), 55.3 (C), 52.2 (CH_2), 47.7 (C), 46.7 (CH), 36.9 (CH), 35.5 (CH), 32.4 (CH), 31.9 (C), 31.7 (3 × CH_3), 31.6 (CH), 30.9 (CH), 29.3 (2 × CH_3); HRMS (ESI) Exact mass calculated for $[C_{18}H_{16}NO_2]^-$ $[M–H]^-$: 288.1969, found 288.1962.

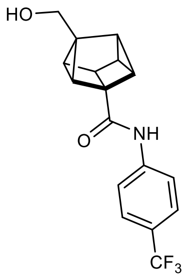

(±)-3-(Hydroxymethyl)-*N*-[4-(trifluoromethyl)phenyl]cuneane-1-carboxamide (3f). General Procedure B was followed using cubane **1n** (64.3 mg, 0.20 mmol). Purification by column chromatography (50% EtOAc/pentane) gave *cuneane* **3f** as a white solid (49.6 mg, 77%). R_f = 0.21 (60% EtOAc/petrol); m.p. 61–62 °C (Et_2O); IR 3307 (OH), 3050, 1659 (C=O), 1601, 1524, 1320, 1111, 1063, 1015, 836 cm^{-1}; ^{1}H NMR (400 MHz, $CDCl_3$) δ 7.71–7.64 (3H, m, Ar**H** and N**H**), 7.57 (2H, d, *J* = 8.5 Hz, Ar**H**), 4.11 (1H, d, *J* = 11.7 Hz, C**H**$_a$H$_b$OH), 4.00 (1H, d, *J* = 11.7 Hz, CH$_a$**H**$_b$OH), 3.13–3.09 (1H, m, cuneanyl C**H**), 2.57-2.49 (3H, m, cuneanyl C**H**), 2.43–2.36 (2H, m, cuneanyl C**H**), 2.13 (1H, br s, O**H**); ^{13}C NMR (101 MHz, $CDCl_3$) δ 170.5 (C), 141.0 (C), 126.4 (q, J_{C-F} = 3.9 Hz, 2 × CH), 126.1 (q, J_{C-F} = 32.6 Hz, C), 123.2 (q, J_{C-F} = 271.4 Hz, C), 119.5 (2 × CH), 61.4 (CH_2), 57.2 (C), 47.8 (C), 47.4 (CH), 36.7 (CH), 35.7 (CH), 32.4 (CH), 31.9 (CH), 31.3 (CH); ^{19}F NMR (376 MHz, $CDCl_3$) δ –62.1 (s, 3 × F); HRMS (ESI) Exact mass calculated for $[C_{17}H_{14}F_3NNaO_2]^+$ $[M+Na]^+$: 344.0869, found 344.0867.

Slow diffusion of *n*-heptane into a solution of **3f** in CH_2Cl_2 gave crystals suitable for X-ray crystallography:

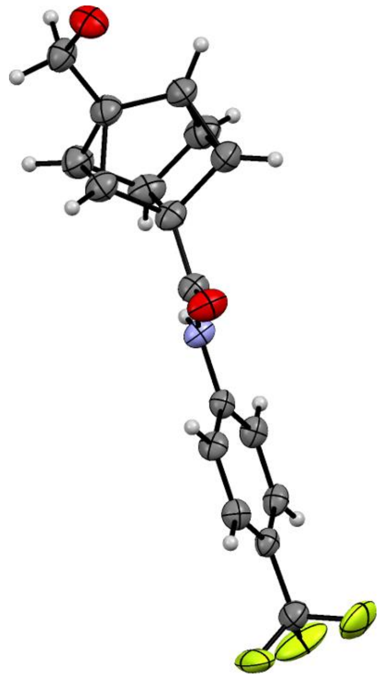

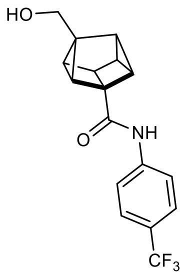

3f
CCDC 2248871

ORTEP with ellipsoid probabilities at 50%

Note: The racemic mixture of chiral entities crystallized in a centrosymmetric space group with opposite enantiomers orientationally disordered such that all molecules are coincident with each other, except for the hydroxymethyl groups which are disordered across the 3- and 4-positions of the cuneane. The hydroxyl oxygen atoms of the pair of disordered hydroxymethyl groups are further conformationally disordered in two positions each, resulting in a total of four hydroxymethyl group positions. The trifluoromethyl group is disordered over two orientations. The aforementioned disorder is not shown in the ORTEP image above, for clarity. Please see the .cif file for details.

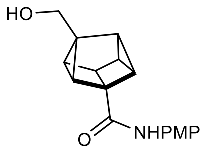

(±)-3-(Hydroxymethyl)-*N*-(4-methoxyphenyl)cuneane-1-carboxamide (3g). General Procedure B was followed using cubane **1o** (56.6 mg, 0.20 mmol) but for a reaction time of 32 h. Purification by column chromatography (60% EtOAc/pentane to 100% EtOAc) gave *cuneane* **3g** as a colorless foam (42.5 mg, 75%). R_f = 0.37 (100% EtOAc); m.p. 190–192 °C ($CDCl_3$); IR 3343 (NH), 3235 (OH), 2966, 1634 (C=O), 1597, 1514, 1233, 1027, 825, 520 cm^{-1}; ^1H NMR (500 MHz, $CDCl_3$) δ 7.47–7.43 (2H, m, Ar**H**), 7.43 (1H, br s, N**H**), 6.87–6.82 (2H, m, Ar**H**), 4.08 (1H, d, *J* = 11.8 Hz, C**H**$_a$H$_b$OH), 4.00 (1H, d, *J* = 11.8 Hz, CH$_a$**H**$_b$OH), 3.79 (3H, s, C**H**$_3$), 3.09–3.08 (1H, m, cuneanyl C**H**), 2.53–2.46 (3H, m, cuneanyl C**H**), 2.39–2.36 (1H, m, cuneanyl C**H**), 2.36-2.33 (1H, m, cuneanyl C**H**), 2.20 (1H, br s, O**H**); ^{13}C NMR (126 MHz, $CDCl_3$) δ 170.0 (C), 156.5 (C), 131.0 (2 × CH), 121.8 (2 × CH), 114.3 (CH), 61.5 (CH_2),

57.0 (C), 55.6 (CH_3), 47.8 (C), 47.2 (CH), 36.9 (CH), 35.7 (CH), 32.5 (CH), 31.8 (CH), 31.1 (CH); HRMS (ESI) calculated for $[C_{17}H_{18}NO_3]^+$ $[M+H]^+$ 284.1291, found 284.1281.

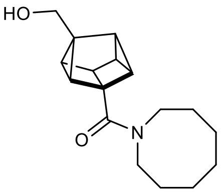

(±)-Azocan-1-yl[3-(hydroxymethyl)cunean-1-yl]methanone (3h). General Procedure B was followed using cubane **1p** (54.6 mg, 0.20 mmol). Purification by column chromatography (60% EtOAc/pentane to 100% EtOAc) gave *cuneane* **3h** as a colorless oil (52.0 mg, 95%). R_f = 0.35 (100% EtOAc); IR 3378 (OH), 2927, 1609 (C=O), 1425, 1358, 1047, 907, 824, 724, 645 cm^{-1}; ^{1}H NMR (500 MHz, $CDCl_3$) δ 4.05 (1H, d, J = 10.6 Hz, C**H**$_a$H$_b$OH), 4.00 (1H, d, J = 10.6 Hz, CH$_a$**H**$_b$OH), 3.47–3.36 (2H, m, NC**H**$_2$), 3.26–3.20 (3H, m, NC**H**$_2$ and cuneanyl C**H**), 2.49 (1H, br td, J = 5.5, 1.0 Hz, cuneanyl C**H**), 2.43-2.39 (2H, m, cuneanyl C**H**), 2.32–2.27 (2H, m, cuneanyl C**H**), 1.87 (1H, br s, O**H**), 1.80–1.71 (4H, m, azocanyl C**H**$_2$), 1.64–1.57 (2H, m, azocanyl C**H**$_2$), 1.57–1.46 (4H, m, azocanyl C**H**$_2$); ^{13}C NMR (126 MHz, $CDCl_3$) δ 169.8 (C), 61.8 (CH_2), 55.6 (C), 48.9 (C), 48.7 (CH_2), 45.9 (CH_2), 44.3 (CH), 39.3 (CH), 36.4 (CH), 34.8 (CH), 32.7 (CH), 31.8 (CH), 26.7 (CH_2), 26.7 (CH_2), 26.5 (CH_2), 25.5 (CH_2), 23.6 (CH_2); HRMS (ESI) calculated for $[C_{17}H_{24}NO_2]^+$ $[M+H]^+$ 274.1802, found 274.1799.

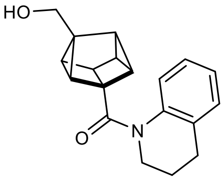

(±)-[3,4-Dihydroquinolin-1(2*H*)-yl][3-(hydroxymethyl)cunean-1-yl]methanone (3i). General Procedure B was followed using cubane **1q** (58.6 mg, 0.20 mmol) but for a reaction time of 32 h. Purification by column chromatography (60% EtOAc/pentane to 100% EtOAc) gave *cuneane* **3i** as a colorless oil (46.6 mg, 80%). R_f = 0.49 (100% EtOAc); IR 3404 (NH), 3038, 2938, 2876, 1633 (C=O), 1492, 1389, 1208, 1023, 759 cm^{-1}; ^{1}H NMR (400 MHz, DMSO-D_6, 100 °C) δ 7.63–7.56 (1H, m, Ar**H**), 7.18–7.10 (2H, m, Ar**H**), 7.10–7.02 (1H, m, Ar**H**), 3.88 (1H, br s, O**H**), 3.83 (2H, s, C**H**$_2$OH), 3.62–3.59 (2H, m, C**H**$_2$N), 3.09–3.05 (1H, m, cuneanyl C**H**), 2.77 (2H, t, J = 6.8 Hz, ArC**H**$_2$), 2.45–2.41 (1H, m, cuneanyl C**H**), 2.34–2.29 (1H, m, cuneanyl C**H**), 2.27–2.20 (2H, m, cuneanyl C**H**), 1.98–1.92 (2H, m, CH_2C**H**$_2$$CH_2$); ^{13}C NMR (101 MHz, DMSO-D_6, 100 °C) δ 168.6 (C), 138.2 (C), 129.8 (C), 128.0 (CH), 125.0 (CH), 123.7 (CH), 122.9 (CH), 58.7 (CH_2), 55.6 (C), 48.1 (C), 43.8 (CH_2), 43.1 (CH), 38.7 (CH), 35.1 (CH), 34.0 (CH), 31.8 (CH), 30.2 (CH), 25.7 (CH_2), 23.0 (CH_2); HRMS (ESI) calculated for $[C_{19}H_{19}NNaO_2]^+$ $[M+Na]^+$: 316.1308, found 316.1308.

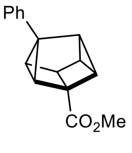

(±)-Methyl 3-phenylcuneane-1-carboxylate (3j). General Procedure B was followed using cubane **1r**[13] (47.7 mg, 0.20 mmol). Purification by column chromatography (100% pentane to 10% EtOAc/pentane) gave *cuneane* **3j** as a colorless liquid (46.9 mg, 98%). R_f = 0.47 (10% EtOAc/pentane); IR 3046, 2950, 1725 (C=O), 1435, 1332, 1221, 1117, 1084, 751, 696 cm^{-1}; ^{1}H NMR (500 MHz, $CDCl_3$) δ 7.29–7.24 (4H, m, Ar**H**), 7.19–7.14 (1H, m, Ar**H**), 3.74

(3H, s, C**H**$_3$), 3.16–3.13 (1H, m, cuneanyl C**H**), 2.77–2.74 (1H, m, cuneanyl C**H**), 2.69 (1H, td, *J* = 5.4, 1.0 Hz, cuneanyl C**H**), 2.58–2.52 (2H, m, cuneanyl C**H**), 2.41–2.37 (1H, m, cuneanyl C**H**); ^{13}C NMR (126 MHz, CDCl$_3$) δ 172.9 (C), 138.9 (C), 128.5 (2 × CH), 126.9 (2 × CH), 126.0 (CH), 54.8 (C), 52.0 (CH$_3$), 48.6 (C), 48.0 (CH), 41.9 (CH), 41.0 (CH), 33.5 (CH), 32.2 (CH), 30.9 (CH); HRMS (ESI) Exact mass calculated for $[C_{16}H_{15}O_2]^+$ $[M+H]^+$: 239.1067, found 239.1060.

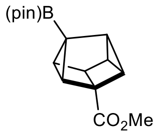

(±)-Methyl-3-(4,4,5,5-tetramethyl-1,3,2-dioxaborolan-2-yl)cuneane-1-carboxylate (3k). General Procedure B was followed using cubane **1s**[14] (47.7 mg, 0.20 mmol). Purification by column chromatography (100% pentane to 5% Et$_2$O/pentane) gave *cuneane* **3k** as an off-white solid (16.5 mg, 29%). R$_f$ = 0.67 (50% Et$_2$O/petrol); m.p. 73–74 °C (Et$_2$O); IR 3047, 2978, 1729 (C=O), 1418, 1331, 1223, 1145, 1051, 1015, 846 cm^{-1}; ^{1}H NMR (500 MHz, CDCl$_3$) δ 3.73 (3H, s, OC**H**$_3$), 3.04–3.02 (1H, m, cuneanyl C**H**), 2.61 (1H, d, *J* = 4.1 Hz, cuneanyl C**H**), 2.49–2.44 (2H, m, cuneanylC**H**), 2.41 (1H, t, *J* = 4.8 Hz, cuneanyl C**H**), 2.27–2.24 (1H, m, cuneanyl C**H**), 1.26 (12H, s, 2 × C(C**H**$_3$)$_2$); ^{13}C NMR (126 MHz, CDCl$_3$) δ 173.2 (C), 83.6 (2 × C), 54.3 (C), 51.9 (CH$_3$), 47.3 (CH), 39.8 (CH), 38.5 (CH), 33.7 (CH), 32.4 (CH), 32.3 (CH), 24.93 (2 × CH$_3$), 24.87 (2 × CH$_3$), the quaternary carbon (C) next to boron was not observed due to quadrupolar coupling effects of ^{11}B; ^{11}B NMR (128 MHz, CDCl$_3$) δ 33.3; HRMS (ESI) Exact mass calculated for $[C_{16}H_{21}BNaO_4]^+$ $[M+Na]^+$: 311.1425, found 311.1431.

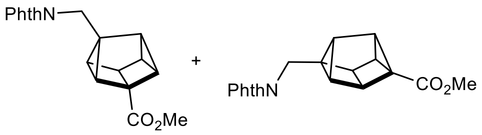

3k (3:1 mixture) **2k**

(±)-Methyl 3-[(1,3-dioxoisoindolin-2-yl)methyl]cuneane-1-carboxylate (3l) and (±)-methyl 6-[(1,3-dioxoisoindolin-2-yl)methyl]cuneane-2-carboxylate (2l). General Procedure B was followed using cubane **1t** (64.3 mg, 0.20 mmol). Purification by column chromatography (100% cyclohexane to 50% EtOAc/cyclohexane) gave a 3:1 mixture of *cuneanes* **3l** and **2l** as a white solid. R$_f$ (major isomer **3l**) = 0.45 (30% EtOAc/cyclohexane); R$_f$ (minor isomer **2l**) = 0.40 (30% EtOAc/cyclohexane); IR 1701 (C=O), 1434, 1404, 1382, 1330, 1263, 1223, 1081, 1008 cm^{-1}; HRMS (ESI) Exact mass calculated for $[C_{19}H_{15}NO_4Na]^+$ $[M+Na]^+$: 344.0893, found 344.0894.

*NMR data of major isomer **3l***: ^{1}H NMR (600 MHz, CDCl$_3$) δ 7.89–7.87 (2H, m, ArC**H**), 7.74–7.73 (2H, m, ArC**H**), 4.16 (1H, d, *J* = 14.6 Hz, C**H**$_a$H$_b$N), 4.13 (1H, d, *J* = 14.6 Hz, CH$_a$**H**$_b$N), 3.72 (3H, s, C**H**$_3$), 2.98 (1H, s, cuneanyl C**H**), 2.59 (1H, d, *J* = 4.2 Hz, cuneanyl C**H**), 2.56 (1H, d, *J* = 5.4 Hz, cuneanyl C**H**), 2.43–2.42 (1H, m, cuneanyl C**H**), 2.39 (1H, t, *J* = 4.6 Hz, cuneanyl C**H**), 2.26–2.24 (1H, m, cuneanyl C**H**); ^{13}C NMR (151 MHz, CDCl$_3$) δ 172.9 (C), 168.7 (2 × C), 134.2 (2 × CH), 132.3 (2 × C), 123.5 (2 × CH), 53.9 (C), 52.0 (CH$_3$), 47.0 (CH), 44.7 (C), 37.8 (CH), 37.1 (CH$_2$), 36.8 (CH), 32.2 (CH), 31.7 (CH), 31.0 (CH).

*NMR data of minor isomer **2l***: ^{1}H NMR (600 MHz, $CDCl_3$) δ 7.86–7.84 (2H, m, ArC**H**), 7.74–7.73 (2H, m, ArC**H**), 3.81 (1H, d, *J* = 14.8 Hz, C$\mathbf{H}_a$$H_b$N), 3.81 (1H, d, *J* = 14.8 Hz, C$H_a$$\mathbf{H}_b$N), 3.58 (3H, s, C$\mathbf{H}_3$), 2.98 (1H, s, cuneanyl C**H**), 2.90 (1H, br s, cuneanyl C**H**), 2.82–2.73 (2H, m, cuneanyl C**H**), 2.51 (1H, d, *J* = 5.1 Hz, 1H, cuneanyl C**H**), 2.46–2.44 (1H, m, cuneanyl C**H**); ^{13}C NMR (151 MHz, $CDCl_3$) δ 171.3 (C), 168.4 (2 × C), 134.1 (2 × CH), 132.2 (2 × C), 123.5 (2 × CH), 51.5 (CH_3), 44.9 (C), 44.3 (C), 43.4 (CH), 41.2 (CH), 40.7 (CH), 39.1 (CH), 38.2 (CH_2), 37.3 (CH), 35.1 (CH).

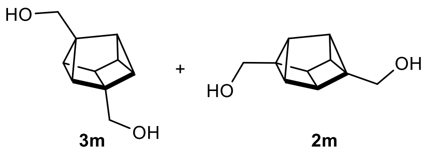

91% (19:1 mixture of **3l**:**2l**)

(±)-(Cuneane-1,3-diyl)dimethanol (3m) and (±)-(cuneane-2,6-diyl)dimethanol (2m). General Procedure B was followed using cubane **1u** (32.8 mg, 0.20 mmol). Purification by column chromatography (5% MeOH/CH_2Cl_2) gave a 19:1 inseparable mixture of *cuneanes* **3m** and **2m** as a colorless oil (30.0 mg, 91%). R_f = 0.18 (2% MeOH/CH_2Cl_2); IR 3315 (OH), 3033, 2865, 1327, 1120, 1089, 1008, 869, 803, 648 cm^{-1}; HRMS (ESI) Exact mass calculated for $[C_{10}H_{12}O_2Na]^+$ $[M+Na]^+$: 187.0730, found 187.0726.

*NMR data of major isomer **3m***: ^{1}H NMR (500 MHz, $CDCl_3$) δ 4.02 (1H, d, *J* = 11.7 Hz, C$\mathbf{H}_a$$H_b$OH), 3.94 (1 H, d, *J* = 11.7 Hz, C$H_a$$\mathbf{H}_b$OH), 3.84 (2H, s, C$\mathbf{H}_2$OH), 2.56 (1H, dq, *J* = 2.6, 1.3 Hz, cuneanyl C**H**), 2.37 (1H, t, *J* = 5.6 Hz, cuneanyl C**H**), 2.21–2.09 (6H, m, cuneanyl C**H**);^{13}C NMR (126 MHz, $CDCl_3$) δ 62.3 (CH_2), 62.2 (CH_2), 55.0 (C), 47.7 (C), 42.6 (CH), 37.5 (CH), 35.1 (CH), 32.7 (CH), 31.7 (CH), 30.2 (CH).

*Characteristic NMR data of minor isomer **2m***: ^{1}H NMR (500 MHz, $CDCl_3$) δ 3.67 (2H, d, *J* = 12.1 Hz, 2 × C$\mathbf{H}_a$$H_b$OH), 3.56 (2H, d, *J* = 12.1 Hz, 2 × C$H_a$$\mathbf{H}_b$OH), 2.73 (2H, t, *J* = 1.0 Hz, cuneanyl C**H**); 2.24 (2H, dt, *J* = 5.7, 1.0 Hz, cuneanyl C**H**); ^{13}C NMR (126 MHz, $CDCl_3$) δ 62.6 (2 × CH_2), 48.4 (2 × C), 39.5 (2 × CH), 35.2 (2 × CH), 34.3 (2 × CH).

Methyl-1-bromohomocubyl-4-carboxylate (6)

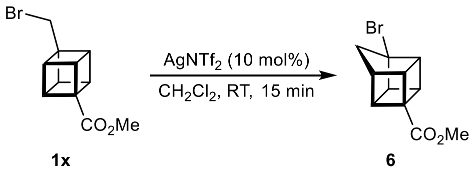

An oven-dried microwave vial equipped with a stirrer bar was charged with cubane **1x** (51.0 mg, 0.20 mmol) and $AgNTf_2$ (7.8 mg, 0.02 mmol). The vial was sealed with a septum-lined cap and CH_2Cl_2 (2 mL) (undried, obtained from commercial vendors used without further purification) was added. The reaction was stirred at room temperature for 15 min and concentrated *in vacuo* to leave a crude homocubane **6** and residual $AgNTf_2$ as a colorless oil (49.8 mg), which displayed spectroscopic data consistent with those reported previously.[19] None of the starting cubane **1x** was detected in the

^{1}H NMR spectrum of the crude material (>95% conversion). R_f = 0.59 (20% EtOAc/petrol); IR 2992, 1725 (C=O), 1434, 1328, 1222, 1196, 1074, 934, 775, 727 cm^{-1}; ^{1}H NMR (400 MHz, $CDCl_3$) δ 3.69–3.61 (3H, m, homocubyl C**H**), 3.67 (3H, s, C**H**$_3$), 3.45–3.41 (2H, m, homocubyl C**H**), 3.33–3.29 (1H, m, homocubyl C**H**), 2.16 (2H, d, *J* = 1.7 Hz, C**H**$_2$); ^{13}C NMR (126 MHz, $CDCl_3$) δ 172.3 (C), 62.7 (C), 52.9 (CH_2), 51.7 (CH_3), 50.1 (2 × CH), 48.5 (C), 48.4 (2 × CH), 43.4 (CH), 41.1 (CH); HRMS (ESI) Exact mass calculated for $[C_{11}H_{11}BrNaO_2]^+$ [M+Na]$^+$: 276.9835, found 276.9838.

2-Methoxycarbonylcuneane-6-carboxylic acid (14)

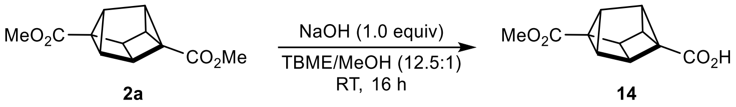

Cubane **2a** (700 mg, 3.18 mmol) was dissolved in TBME (20 mL) in a flask equipped with a stirrer bar. NaOH (2 M in MeOH, 1.59 mL, 3.18 mmol) was added dropwise and the resulting suspension was stirred at room temperature for 16 h. The reaction was concentrated *in vacuo* and the residue was redissolved in deionised H_2O (15 mL) and washed with CH_2Cl_2 (3 × 15 mL). The aqueous layer was acidifed to pH 2 and the resulting suspension was extracted with CH_2Cl_2 (3 × 40 mL). The combined organic extracts were dried (Na_2SO_4), filtered, and concentrated *in vacuo* to give *cuneane* **14** as a white solid (435 mg, 66%). m.p. 169–170 °C ($CDCl_3$); IR 1719 (C=O), 1669 (C=O), 1438, 1375, 1289, 1229, 1203, 1097, 1069 cm^{-1}; ^{1}H NMR (400 MHz, $CDCl_3$) δ 3.63 (3H, s, C**H**$_3$), 3.21–3.19 (1H, m, cuneanyl C**H**), 3.15–3.06 (5H, m, cuneanyl C**H**); ^{13}C NMR (75 MHz, $CDCl_3$) δ 176.5 (C), 170.6 (C), 51.7 (CH_3), 44.9 (CH), 43.7 (C), 43.6 (CH), 43.5 (C), 41.7 (CH), 40.4 (CH), 38.5 (CH), 38.2 (CH); HRMS (ESI) Exact mass calculated for $[C_{11}H_9O_4]^-$ [M−H]$^-$: 205.0506, found 205.0508.

Slow evaporation of a solution of **14** in $CDCl_3$ gave crystals that were suitable for X-ray crystallography:

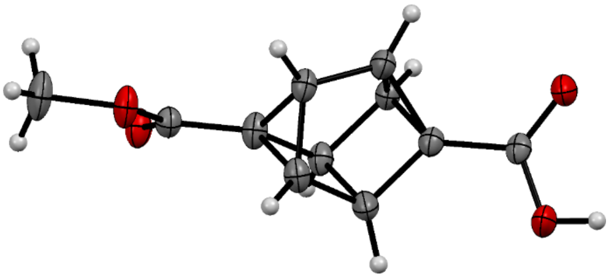

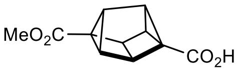

14
CCDC 2248872

ORTEP with ellipsoid probabilities at 50%

Note: Disorder was observed in the structure of the two racemates crystallized with their ester and carboxylic acid moieties overlapping. This disorder is not shown in the ORTEP image above, for clarity. Please see the .cif file for details.

4. Computational Structural Analysis

Computational studies of the following compounds were performed using BIOVIA Discovery Studio and Python, using MM2 for energy minimization.

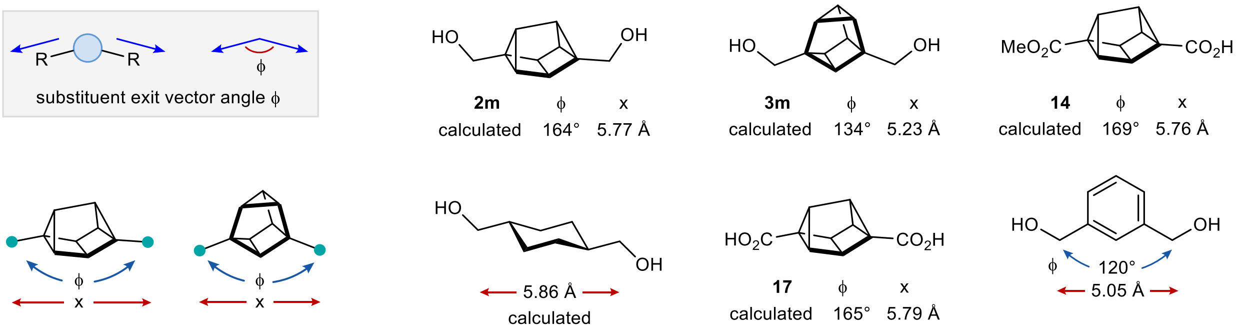

To calculate exit vector angles ϕ and distances x, the following protocol was applied:

The molecule was loaded using RDKit within Python and 501 conformers were generated and minimized. The atoms of interest for exit vector angle analysis were identified and flagged. Coordinates for the 4 points were extracted and 2 vectors were created. The exit vector angle ϕ was calculated using arccosine of the following formula:

$$\cos \phi = \frac{a \cdot b}{|a||b|}$$

The distance x was measured between the two carbon atoms linked directly to the cuneane, cyclohexane, or benzene.

5. Synthesis of a Cuneane Analog of Sonidegib

1-Iodo-4-[4-(trifluoromethoxy)phenyl]cubane (1z)

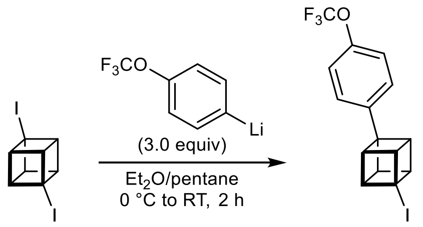

1y **1z**

Safety consideration: Although we used 1,4-diiodocubane (**1y**) without incident, its impact sensitivity and thermal decomposition behavior should be considered when using this compound.[20a]

An oven-dried round-bottom flask equipped with a stirrer bar was sealed with a septum and purged with nitrogen for 15 min. 1-Bromo-4-(trifluoromethoxy)benzene (1.63 g, 2.64 mL, 6.76 mmol) and anhydrous Et_2O (26 mL) were added and the resulting solution was cooled to –78 °C. *t*-BuLi (1.7 M in pentane, 8.75 mL, 14.9 mmol) was added dropwise. The cooling bath was removed and the mixture was allowed to warm naturally over 15 min, before being cooled to 0 °C. Powdered 1,4-diiodocubane **1y**[16] (800 mg, 2.24 mmol) was added in one portion and the resulting suspension was stirred at room temperature for 2 h. The reaction was then cooled to 0 °C and quenched carefully with MeOH (2 mL). The mixture was diluted with Et_2O (25 mL), washed with H_2O (25 mL) and brine (25 mL), dried (Na_2SO_4), and concentrated *in vacuo*. Purification of the residue by column chromatography (100% cyclohexane) gave *cubane* **1z** as a white crystalline solid (534 mg, 61%). R_f = 0.57 (100 cyclohexane); m.p. 169–170 °C ($CDCl_3$); ^{1}H NMR (400 MHz, $CDCl_3$) δ 7.22–7.17 (4H, m, Ar**H**), 4.32 (6H, br s, cubyl C**H**); ^{13}C NMR (126 MHz, $CDCl_3$) δ 147.7 (d, J_{CF} = 1.4 Hz, C), 140.5 (C), 126.4 (2 × CH), 121.3 (2 × CH), 120.6 (q, J_{CF} = 256.6Hz, CF_3), 60.1 (C), 54.3 (3 × CH), 52.1 (3 × CH), 38.6 (C); ^{19}F NMR (376 MHz, $CDCl_3$) δ –58.2 (s, 3 × F); HRMS (ESI) No expected mass ions could be observed under positive or negative ionization modes.

4-[4-(Trifluoromethoxy)phenyl]cubane-1-carboxylic acid (1aa), (±)-3-[4-(trifluoromethoxy)phenyl]cuneane-1-carboxylic acid (3n) and (±)-*N*-{4-[(2*S*,6*R*)-2,6-dimethylmorpholino]pyridin-3-yl}-3-[4-(trifluoromethoxy)phenyl]-cuneane-1-carboxamide (19)

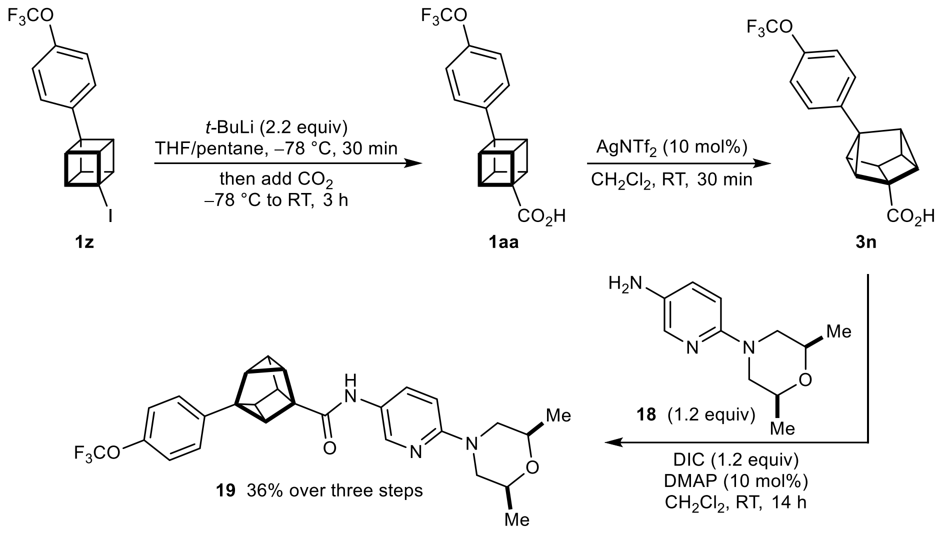

A solution of cubane **1z** (300 mg, 0.77 mmol) was added to a round-bottom flask equipped with a stirrer bar. The flask was sealed with a septum and purged with nitrogen for 15 min. Anhydrous Et_2O (12 mL) was added and the resulting solution was cooled to –78 °C. *t*-BuLi (1.7 M in pentane, 1.0 mL, 1.7 mmol) was added dropwise. The cooling bath was removed and the mixture was allowed to warm naturally over 20 min, before being re-cooled to –78 °C. Solid CO_2 (7.4 g) was added in one portion. The reaction was warmed slowly to room temperature over 5 h, quenched carefully with 2 M aqueous HCl solution (30 mL), and extracted with CH_2Cl_2 (3 × 20 mL). The combined organic layers were dried (Na_2SO_4), filtered, and concentrated *in vacuo* to afford *carboxylic acid* **1aa** (219 mg) as a pale yellow solid, which was used in the next step without purification. ^{1}H NMR (400 MHz, $CDCl_3$) δ 7.22–7.20 (4H, m, Ar**H**), 4.33–4.30 (3H, m, cubyl C**H**), 4.20–4.18 (3H, m, cubyl C**H**); ^{19}F NMR (376 MHz, $CDCl_3$) δ –57.9 (s, 3 × F).

The crude carboxylic acid **1aa** was transferred to an oven-dried microwave vial equipped with a stirrer bar, using CH_2Cl_2 (7 mL) to aid the transfer (rinsing the flask). $AgNTf_2$ (27.5 mg, 0.071 mmol) was added to the solution and the vial was sealed with a Teflon-lined cap. The mixture was stirred vigorously with protection from light for 30 min. The reaction was diluted with CH_2Cl_2 (30 mL) and washed with H_2O (20 mL) and brine (5 × 10 mL). The organic layer was dried (Na_2SO_4), filtered, and concentrated *in vacuo*. Trituration of the residue with a small quantity of ice-cold pentane gave *cuneane* **3n** as a pale yellow oil (164 mg), which was used in the next step without purification. ^{1}H NMR (400 MHz, $CDCl_3$) δ 7.34–7.30 (2H, m, Ar**H**), 7.18–7.15 (2H, m, Ar**H**), 4.29–4.27 (1H, m, cuneanyl C**H**), 2.82–2.81 (1H, m, cuneanyl C**H**), 2.73–2.71 (1H, m, cuneanyl C**H**), 2.64–2.61 (1H,

m, cuneanyl C**H**), 2.51–2.48 (1H, m, cuneanyl C**H**); ^{19}F NMR (376 MHz, $CDCl_3$) δ –58.2 (s, 3 × F); HRMS (ESI) Exact mass calculated for $[C_{16}H_{10}F_3O_3]^-$ $[M{-}H]^-$: 307.0588, found 307.0585.

A portion of cuneane **3n** from the previous reaction (100 mg out of the 164 mg obtained) was dissolved in CH_2Cl_2 (0.5 mL), and added to a solution of amine **18**[21] (80 mg, 0.39 mmol) and DMAP (3.9 mg, 0.032 mmol) in CH_2Cl_2 (1.5 mL) in a microwave vial equipped with a stirrer bar, using additional CH_2Cl_2 (0.5 mL) as a rinse. DIC (49 mg, 61 µL, 0.39 mmol) was added, the vial was sealed with septum, and the mixture was stirred for 14 h at room temperature. The reaction was concentrated *in vacuo*. Purification of the residue by column chromatography (100% cyclohexane to 50% EtOAc/cyclohexane) gave *amide* **19** contaminated with some *N*,*N*'-diisopropylurea. This material was dissolved in Et_2O (50 mL), washed with H_2O (3 × 20 mL) and brine (20 mL), dried (Na_2SO_4), filtered, and concentrated *in vacuo* to leave give *amide* **19** as a colorless foam (84 mg, 36% over three steps from **1z**). R_f = 0.41 (50% EtOAc/cyclohexane); m.p. 73–74 °C (Et_2O); IR 3294 (NH), 2978, 1611 (C=O), 1513, 1491, 1452, 1391, 1251, 1223, 1174 cm^{-1}; ^{1}H NMR (600 MHz, $CDCl_3$) δ 8.21 (1H, d, *J* = 2.7 Hz, Ar**H**), 7.98 (1H, dd, *J* = 9.3, 2.6 Hz, Ar**H**), 7.38 (1H, br s, N**H**), 7.33–7.32 (2H, m, Ar**H**), 7.16 (2H, d, *J* =8.2 Hz, Ar**H**), 6.66 (1H, d, *J* = 9.1 Hz, Ar**H**), 4.00–3.97 (2H, m, C**H**$_2$), 3.76–3.71 (2H, m, 2 × C**H**CH_3), 3.26 (1H, br s, cuneanyl C**H**), 2.83 (1H, d, *J* = 4.2 Hz, cuneanyl C**H**), 2.77–2.74 (1H, app t, *J* = 5.5 Hz, cuneanyl C**H**), 2.68–2.67 (1H, m, cuneanyl C**H**), 2.62 (1H, app t, *J* = 4.9 Hz, cuneanyl C**H**), 2.54–2.48 (3H, m, cuneanyl C**H** and C**H**$_2$), 1.27 (6H, d, *J* = 6.3 Hz, 2 × C**H**$_3$); ^{13}C NMR (151 MHz, $CDCl_3$) δ 170.0 (C), 156.5 (C), 147.6 (C), 139.8 (C), 137.5 (C), 131.8 (C), 128.3 (2 × CH), 125.7 (C), 121.2 (2 × CH), 120.6 (q, J_{CF} = 256.8 Hz, C), 107.2 (CH), 71.7 (2 × CH), 57.6 (C), 51.4 (2 × CH_2), 48.6 (C), 48.2 (CH), 42.2 (CH), 41.3 (CH), 34.4 (CH), 32.6 (CH), 31.5 (CH), 19.1 (2 × CH_3); ^{19}F NMR (376 MHz, $CDCl_3$) δ –57.9 (s, 3 × F); HRMS (ESI) Exact mass calculated for $[C_{27}H_{27}F_3N_3O_3]^+$ $[M+H]^+$: 498.1999, found 498.2002.

Slow evaporation of a EtOAc/pentane solution of **19** that had been subjected to HPLC ($MeCN/H_2O$, 0.1% TFA) followed by lyophilization gave crystals of the TFA monohydrate salt that were suitable for X-ray crystallography:

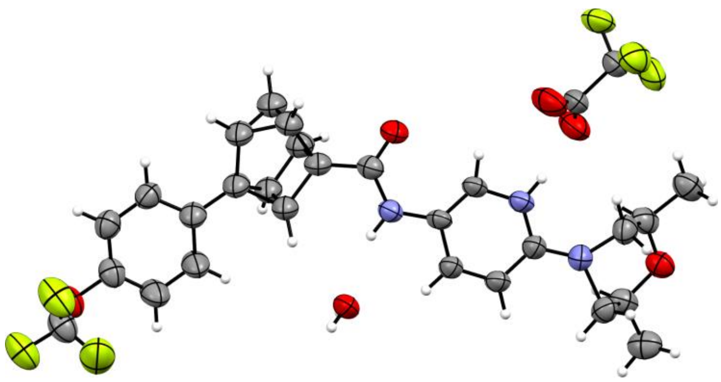

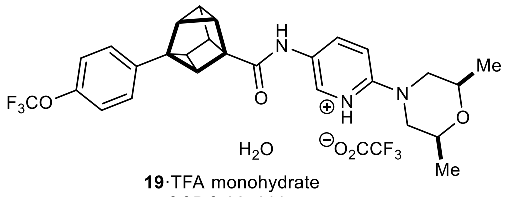

19·TFA monohydrate
CCDC 2279035

Conformational disorder is modelled for the pyridinium-morpholine moiety. The occupancies of the disorder components were refined and constrained to sum to unity giving values of 0.58(1) and 0.42(1), respectively. All chemically identical bond lengths and angles within and between the disordered moieties were restrained to be similar (SAME, SADI). Geometry of the substitution positions of the pyridinium ring were restrained to be planar (FLAT). The anisotropic displacement parameters of the closely lying pyridinium carbon atoms C46/A were constrained to be identical (EADP). Please see the .cif file for details.

6. Physicochemical Measurements

The logP, pK_a in H_2O, and aqueous solubility were performed on a Sirius T3 instrument (Pion). The device consists of a pH-meter electrode, a robot that prepares the solutions needed to perform the measurements, and a UV-vis spectrophotometer. Basic titration is performed with a 0.5 M KOH buffer. Acidic titration is performed with a 0.5 M HCl buffer.

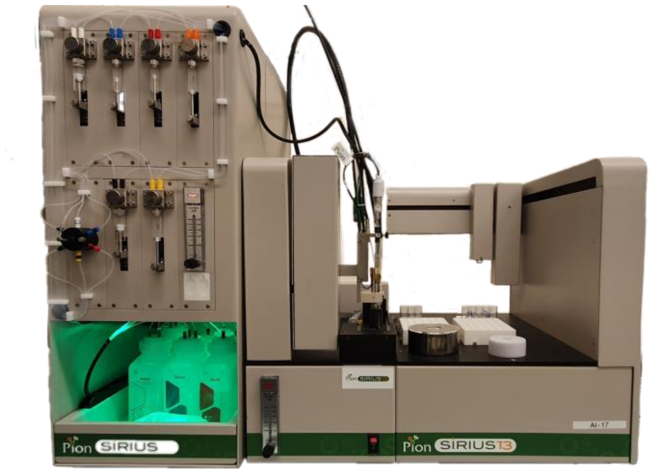

Log P measurements

Log P was determined potentiometrically by using the "Shake flask" method, which consists of dissolving part of the solute in question in a volume of *n*-octanol and water, then performing pH titration.

pK_a Measurements

To obtain the pK_a values, a pH-metric titration experiment was carried out. It can measure pK_a values between 2 and 12. An acidic or base titrant was added to reach a starting pH at which the sample is ionized. Then, small volumes of 0.5 M HCl or KOH titrant were added and the pH was measured after each addition. Potentiometric measurement of basic compounds were done from pH 2 to pH 12 by adding 0.5 M KOH solution. Potentiometric measurement of acidic compounds (**20**, **S1**, and **14**) were done from pH 12 to pH 2 by adding 0.5 M HCl solution. Measurement points were done every 0.2 pH value. After each addition, the sample was stirred for 60 seconds and the pH value was then collected. The titration experiments were done 3 times and the stated values are averages of the 3 measurements.

Solubility Measurements

Solubility measurements were performed using the CheqSol method developed by Pion.[22] Because in most cases, compounds are more soluble at pH values where they ionized, the analyte was solubilized in acidic water before pH-titration was performed, measuring the UV-vis spectrum after each addition. The precipitation point was detected from the reduction of the light transmission during UV-vis measurement, or manually when not obvious. The pH-titration was repeated several times around the precipitation point to determine the solubility value by averaging.

Results

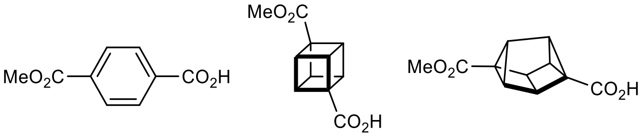

20 S1 14

	20	S1	14
MW	180.16	206.20	206.20
logP	2.02 ± 0.02	0.02 ± 0.01	0.72 ± 0.01
pK_a in H_2O	3.84± 0.01	3.96± 0.01	3.99± 0.02
aqueous solubility (mM)	1.384	148.8	209.8

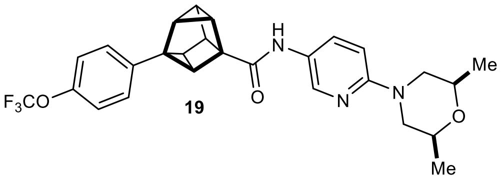

19

	19
MW	497.52
logP	4.74 ± 0.23
aqueous solubility (mM)	–[a]

[a] Too low to be measured.

7. Microsome Stability Measurements for the Cuneane Analog of Sonidegib

Microsome stability measurements were performed by Sygnature Discovery (Nottingham, United Kingdom).

Protocol Summary

The microsomal stability of a test compound is assessed by spiking compound into tissue microsomes containing NADPH in buffer and sampling for up to 1 h at 37 °C. Following protein precipitation and centrifugation, the samples are analyzed by UPLC-MS-MS.

Objective

To assess the metabolic stability of a test compound in tissue microsomes.

Requirements

- Compound identifier and molecular formula
- 20 µL of 1 mM test compound in DMSO or 1 mg solid

Experimental Procedure

Dilutions of a 1 mM test compound DMSO stock solution are prepared in 0.01 M phosphate buffered saline (pH7.4)) so that the final DMSO concentration is 0.1% and the final test compound concentration tested is 1 µM. Following addition of protein (0.5 mg/mL final) and NADPH, the samples are pre-incubated for 10 min prior to the start of the incubation by the addition of test compound. The test compound will be incubated (n = 2; 1 µM final concentration) at 37 °C with tissue microsomes. Aliquots will be sampled at several time-points and mixed with acetonitrile (containing 1 µM Labetalol as internal standard) to terminate the reaction and precipitate the proteins. All the samples will be mixed, centrifuged and the supernatants analysed by UPLC-MS-MS.

Data Analysis

The intrinsic clearance (Cl_{int}) and half-life ($t_{1/2}$) values will be determined from the slope of the parent depletion curve. Control compounds for low, moderate, and high intrinsic clearance, respectively, in microsomes from either human and/or mouse will be included.

Study Design Summary

Description	Units	Variable
Test System	-	Liver Microsomes
Total Incubation Volume	mL	0.5
Test Concentration	mM	1
Replicate	-	2
Pre-Incubation Period	min	10
Incubation Period	min	0, 5, 15, 30, 45
[NADPH]	mM	1
[DMSO]	%	0.1
[Protein]	mg/mL	0.5
[MeCN]	%	0.45

Materials

Description	Supplier	Code
Human liver microsomes	Corning	452117
CD1 mouse liver microsomes	Corning	452701
DPBS (Dulbecco's phosphate buffered saline)	Sigma	D8537
DMSO	Sigma	34869
Acetonitrile	Fisher	A/0627/17
NADPH (tetrasodium salt)	Sigma	N7505
Labetalol hydrochloride	Sigma	PHR1335
Verapamil hydrochloride	Alfa Aesar	J61535.03
Diltiazem hydrochloride	Sigma	D2521
Diphenhydramine hydrochloride	Sigma	D3630

Results

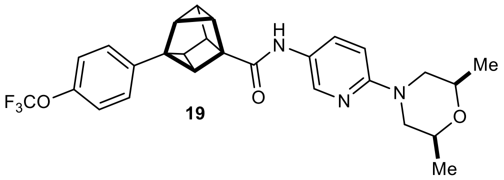

Human liver microsomes		Mouse liver microsomes	
Cl_{int} (µL/min/mg)	$t_{1/2}$ (min)	Cl_{int} (µL/min/mg)	$t_{1/2}$ (min)
76	19	61	23

8. NMR Spectra

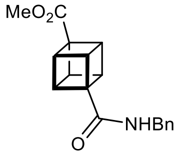

1d

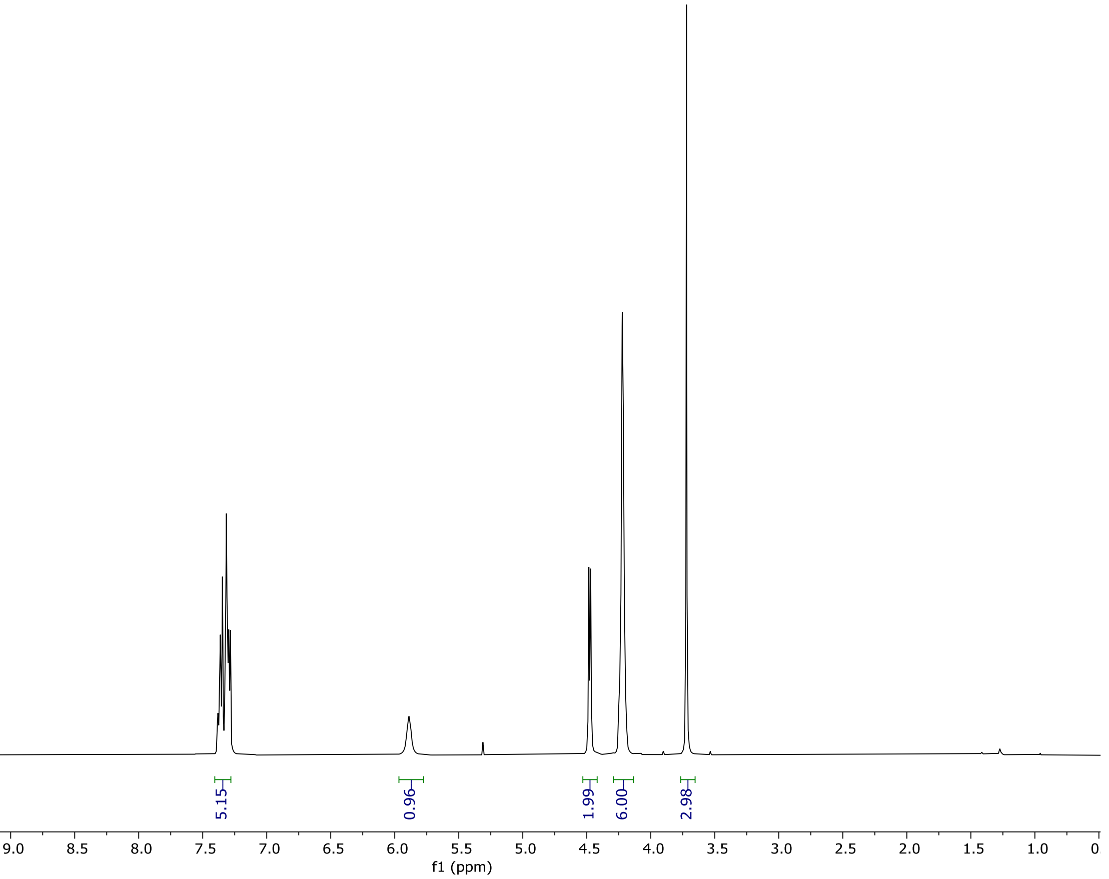

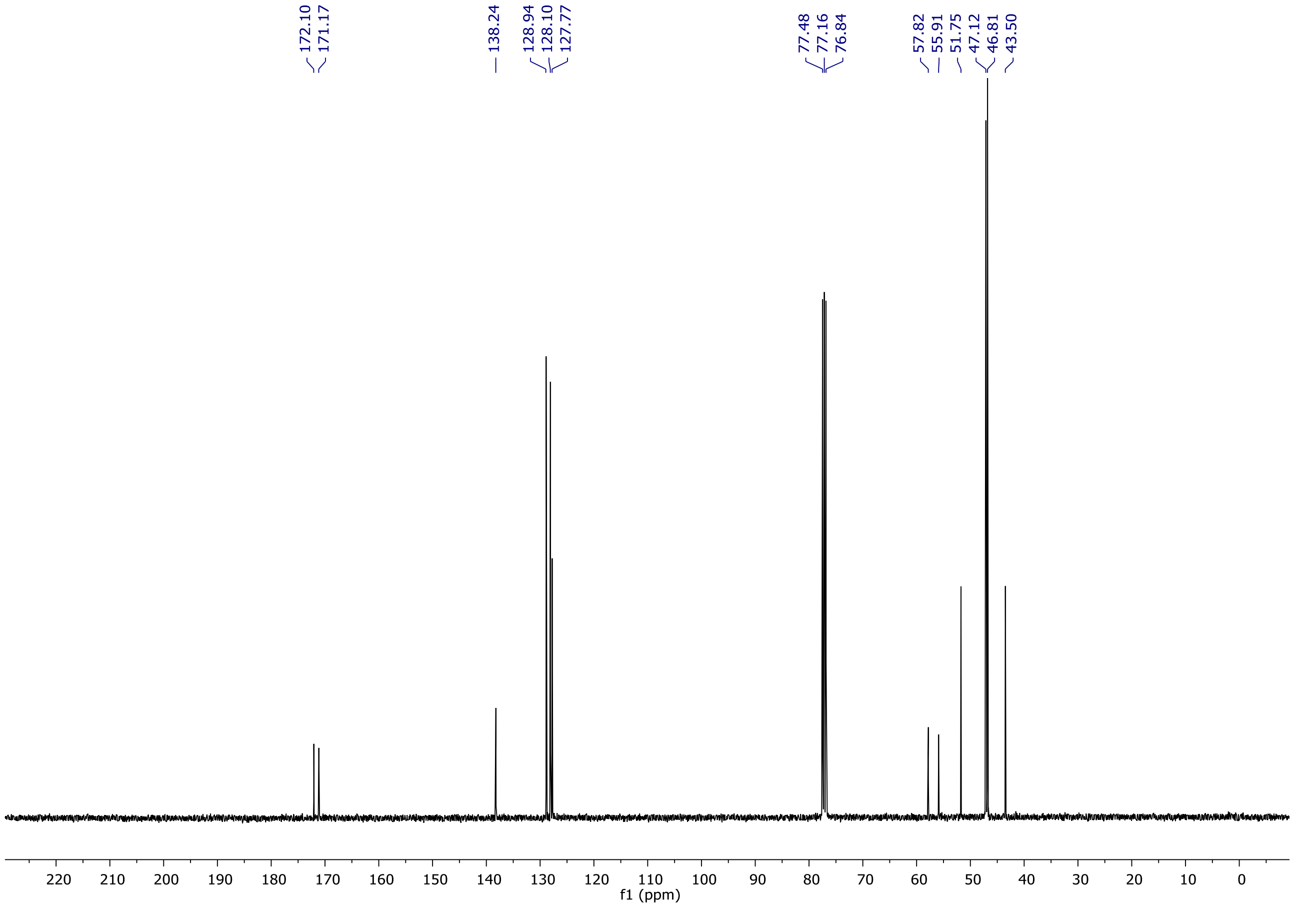

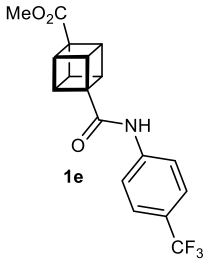
MeO2C
NH
O
CF3

1e

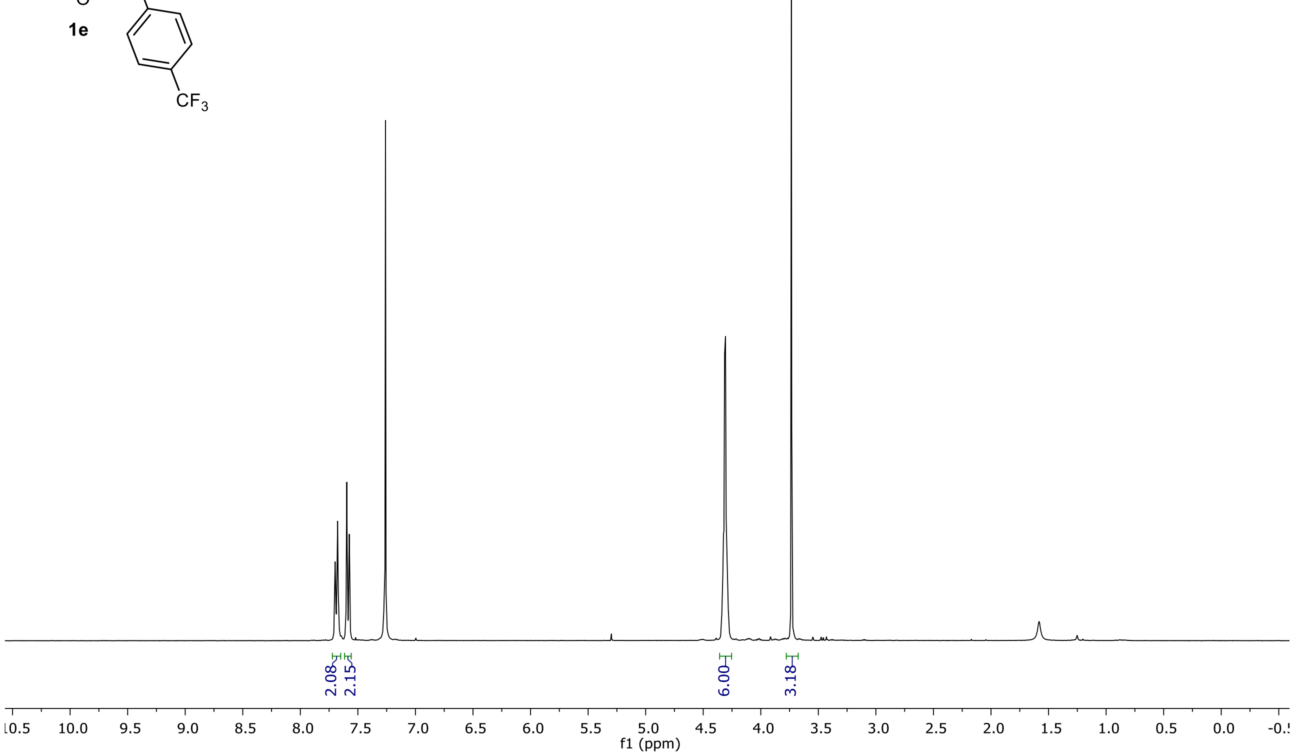
2.08
2.15
6.00
3.18
10.5 10.0 9.5 9.0 8.5 8.0 7.5 7.0 6.5 6.0 5.5 5.0 4.5 4.0 3.5 3.0 2.5 2.0 1.5 1.0 0.5 0.0
f1 (ppm)

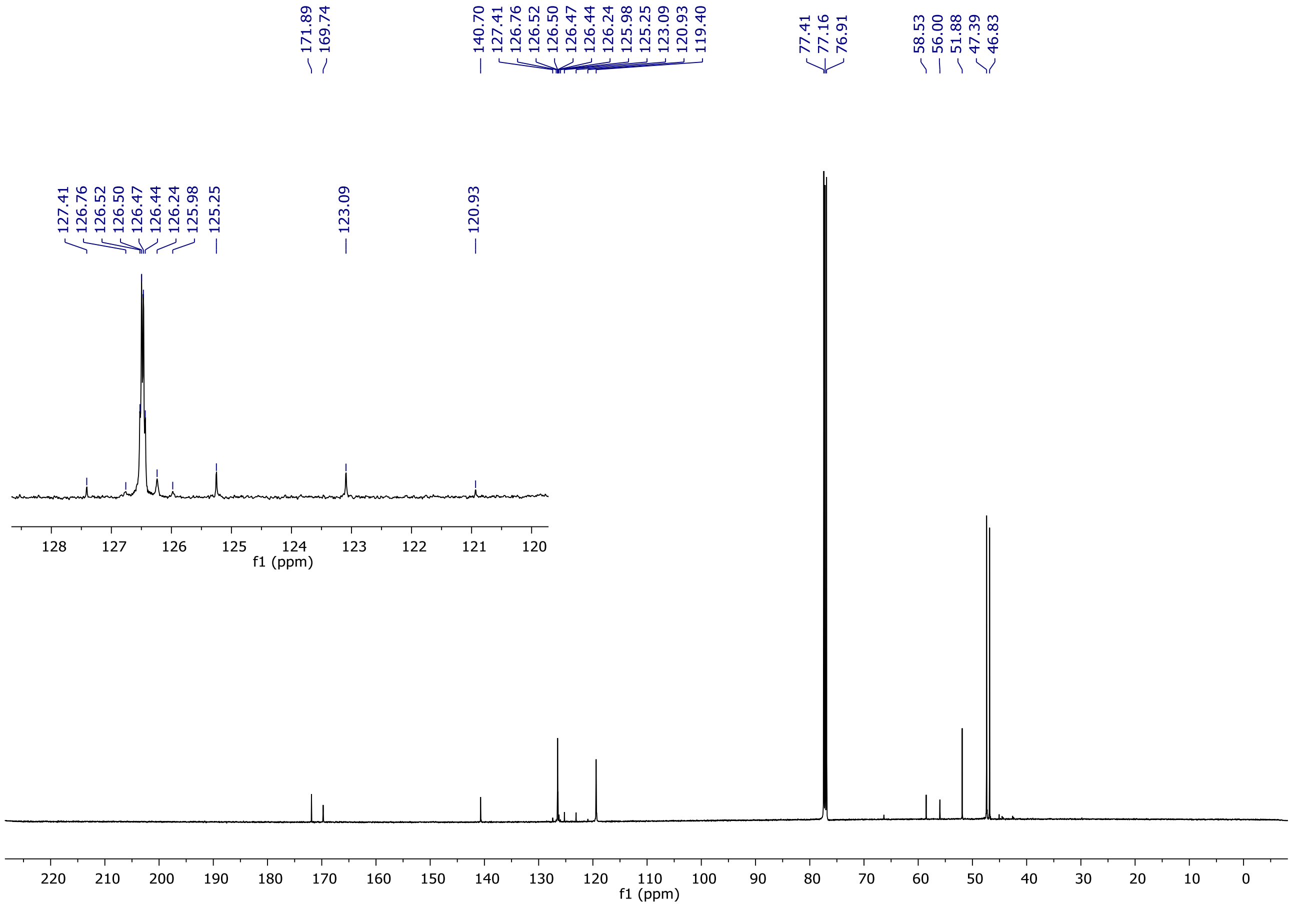
171.89
169.74
140.70
127.41
126.76
126.52
126.50
126.47
126.44
126.24
125.98
125.25
123.09
120.93
119.40
77.41
77.16
76.91
58.53
56.00
51.88
47.39
46.83
128 127 126 125 124 123 122 121 120
f1 (ppm)
220 210 200 190 180 170 160 150 140 130 120 110 100 90 80 70 60 50 40 30 20 10 0
f1 (ppm)

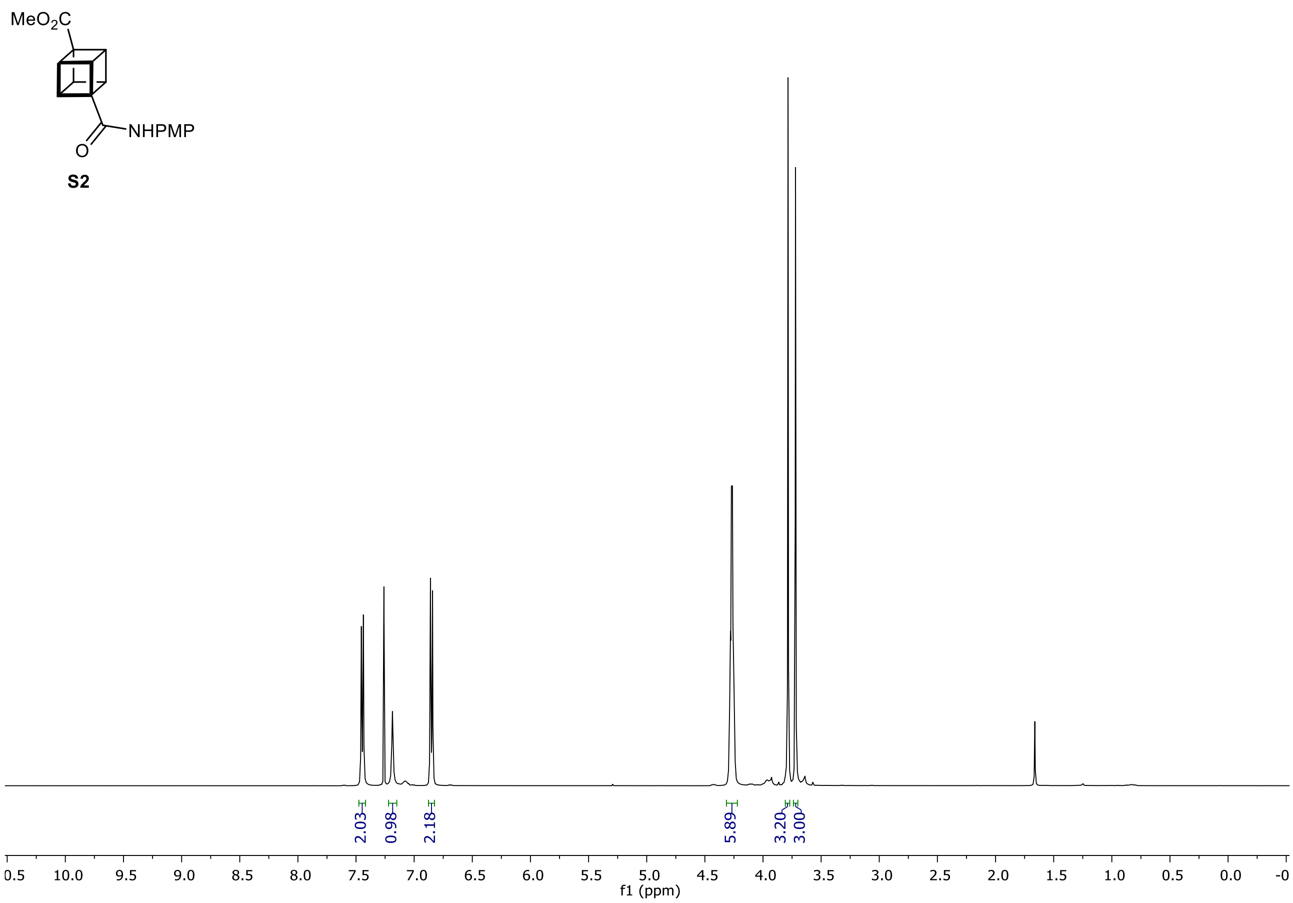
MeO2C
NHPMP
O
S2
2.03
0.98
2.18
5.89
3.20
3.00
f1 (ppm)

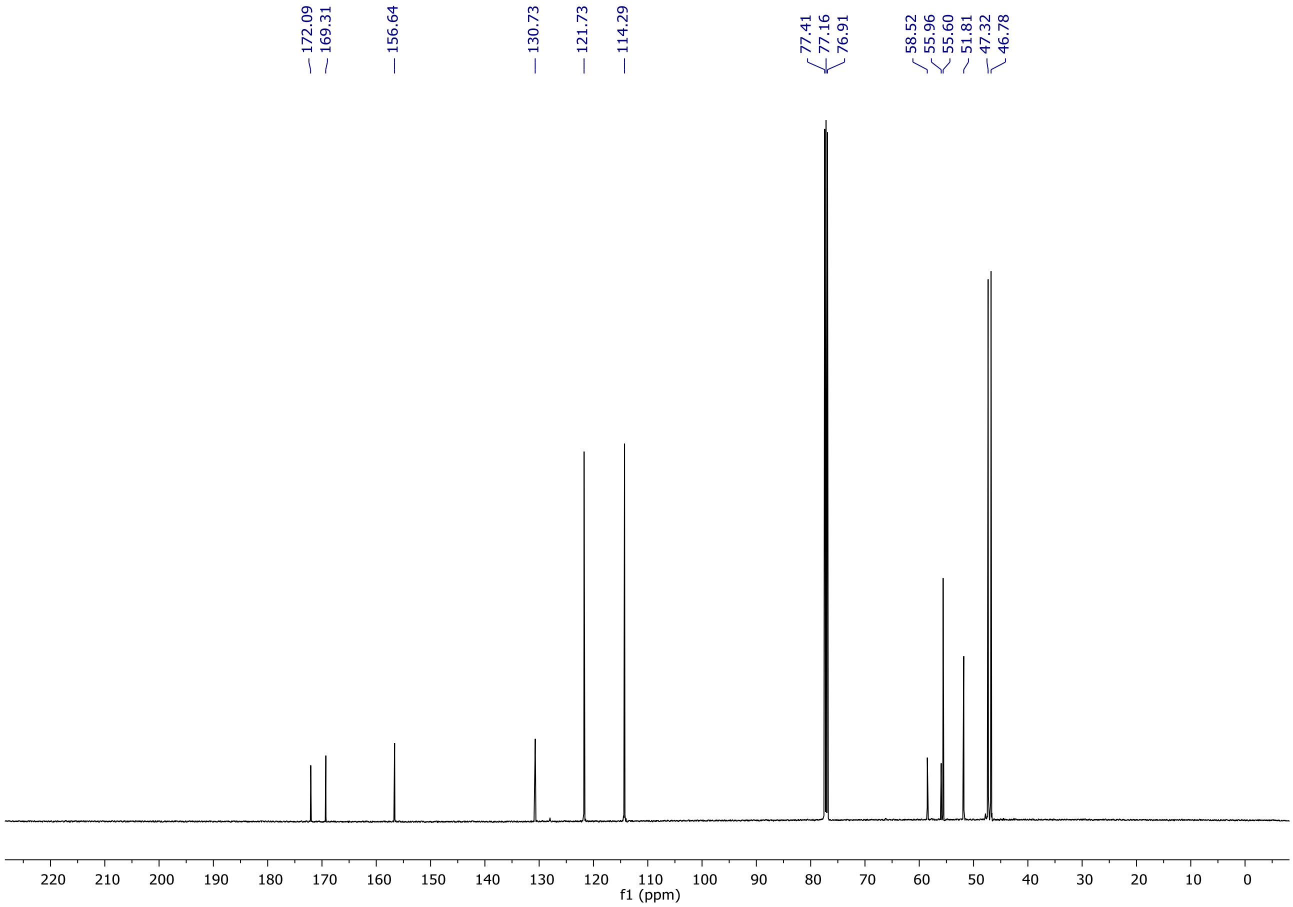
172.09
169.31
156.64
130.73
121.73
114.29
77.41
77.16
76.91
58.52
55.96
55.60
51.81
47.32
46.78
f1 (ppm)

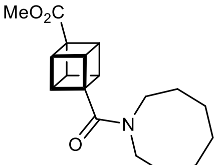
MeO2C
N
O

1g

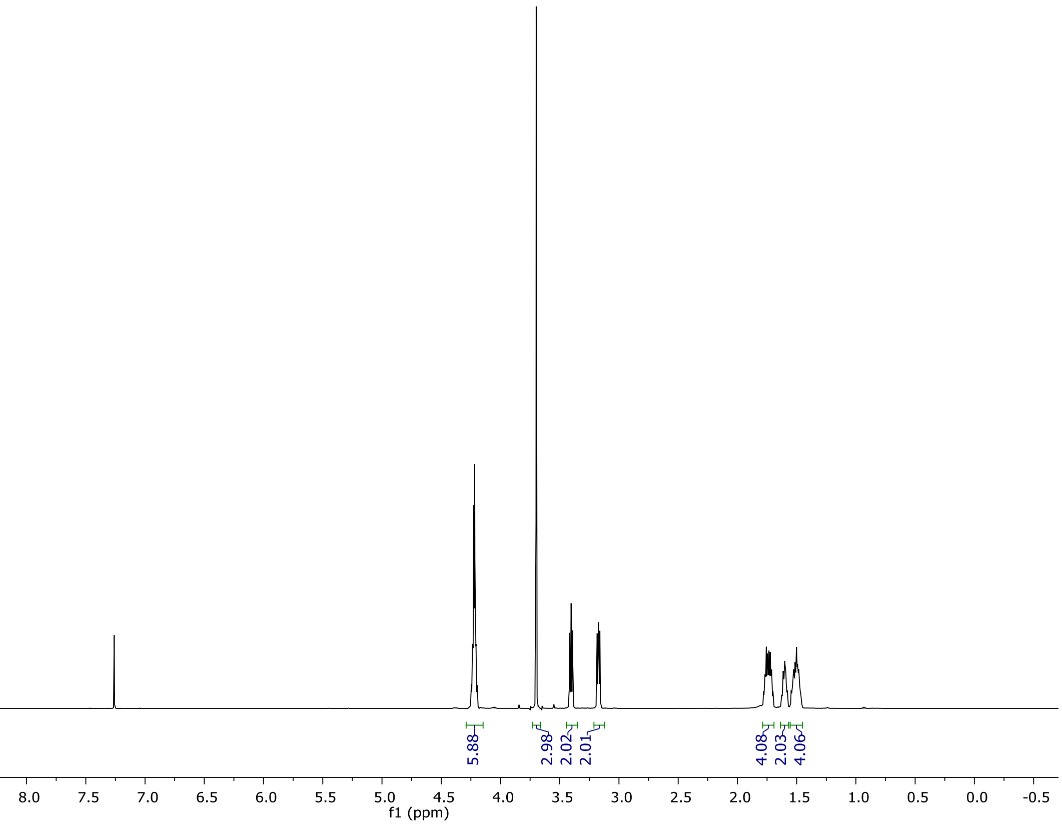
5.88
2.98
2.02
2.01
4.08
2.03
4.06
10.0 9.5 9.0 8.5 8.0 7.5 7.0 6.5 6.0 5.5 5.0 4.5 4.0 3.5 3.0 2.5 2.0 1.5 1.0 0.5 0.0 -0.5
f1 (ppm)

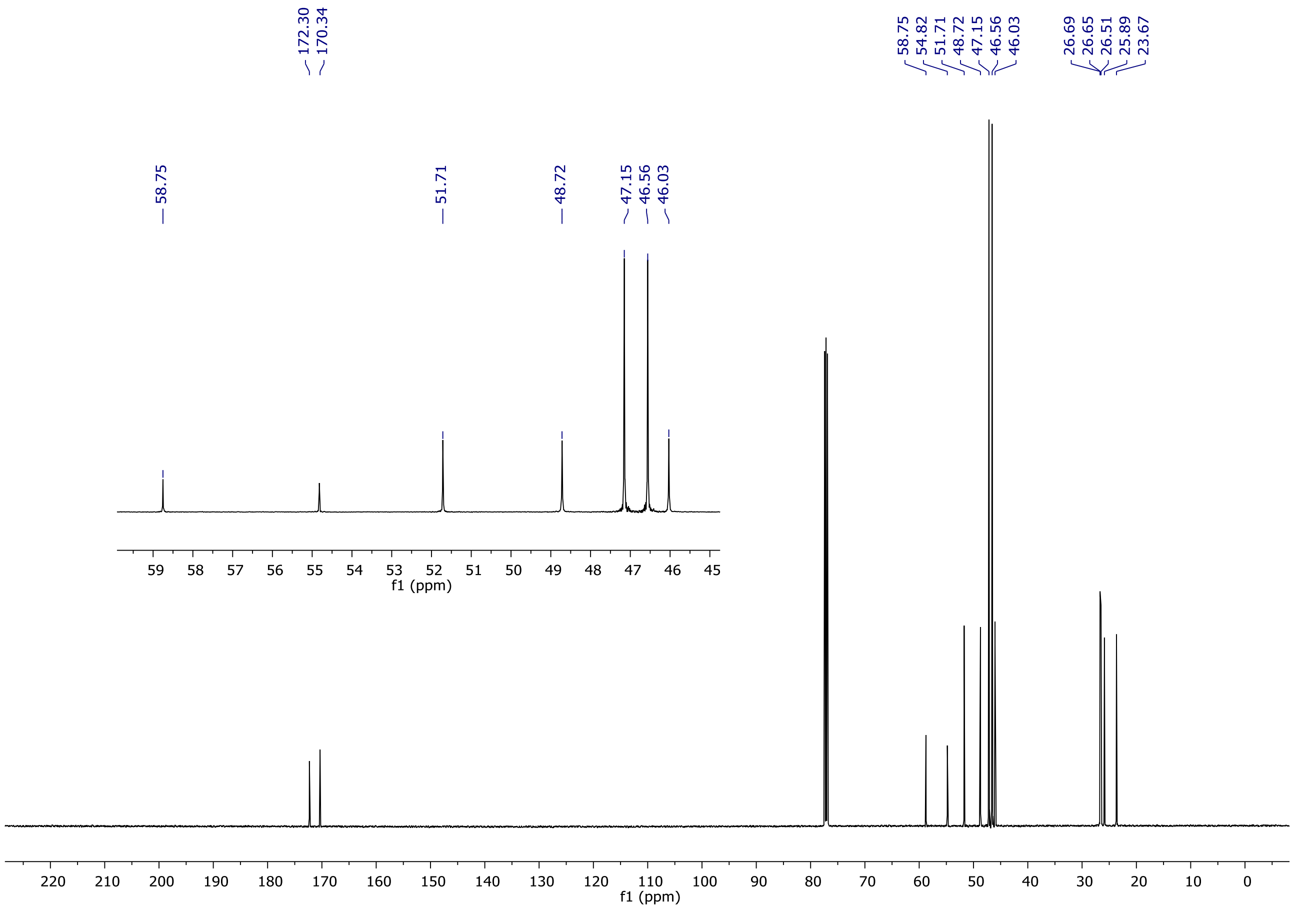
172.30
170.34
58.75
54.82
51.71
48.72
47.15
46.56
46.03
26.69
26.65
26.51
25.89
23.67
58.75
51.71
48.72
47.15
46.56
46.03
59 58 57 56 55 54 53 52 51 50 49 48 47 46 45
f1 (ppm)
220 210 200 190 180 170 160 150 140 130 120 110 100 90 80 70 60 50 40 30 20 10 0
f1 (ppm)

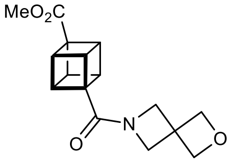
MeO2C
N
O
O

1h

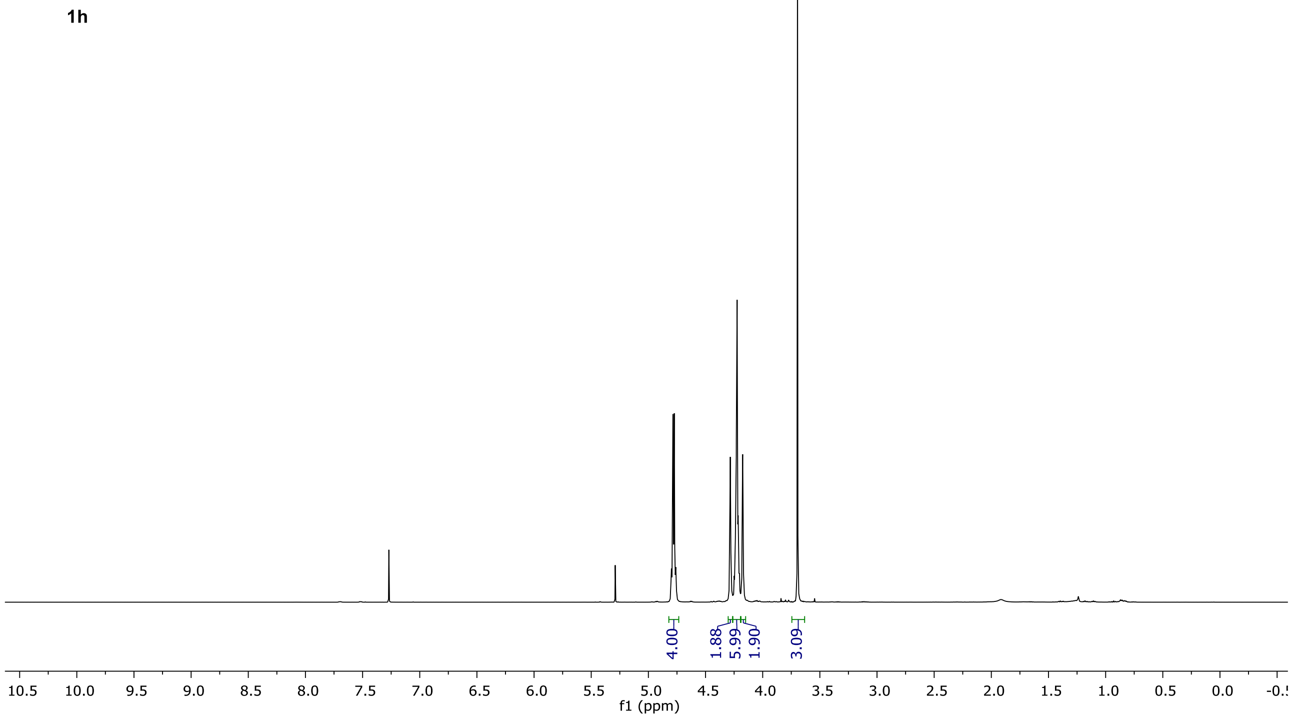
4.00
1.88
5.99
1.90
3.09
10.5 10.0 9.5 9.0 8.5 8.0 7.5 7.0 6.5 6.0 5.5 5.0 4.5 4.0 3.5 3.0 2.5 2.0 1.5 1.0 0.5 0.0
f1 (ppm)

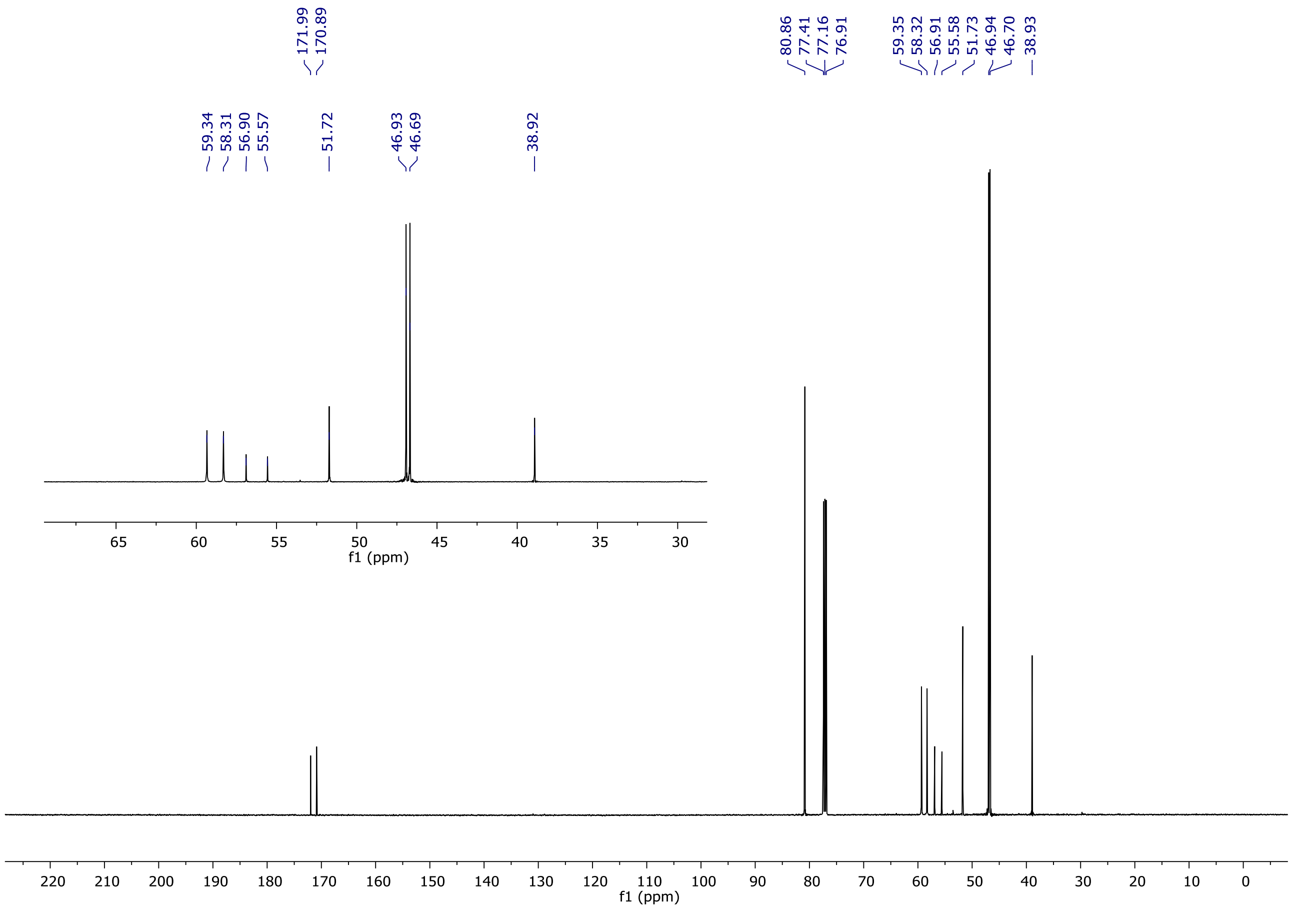
171.99
170.89
80.86
77.41
77.16
76.91
59.35
58.32
56.91
55.58
51.73
46.94
46.70
38.93
59.34
58.31
56.90
55.57
51.72
46.93
46.69
38.92
65 60 55 50 45 40 35 30
f1 (ppm)
220 210 200 190 180 170 160 150 140 130 120 110 100 90 80 70 60 50 40 30 20 10 0
f1 (ppm)

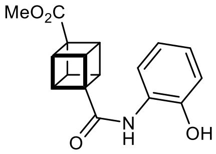
MeO2C
N
H
O
OH

S3

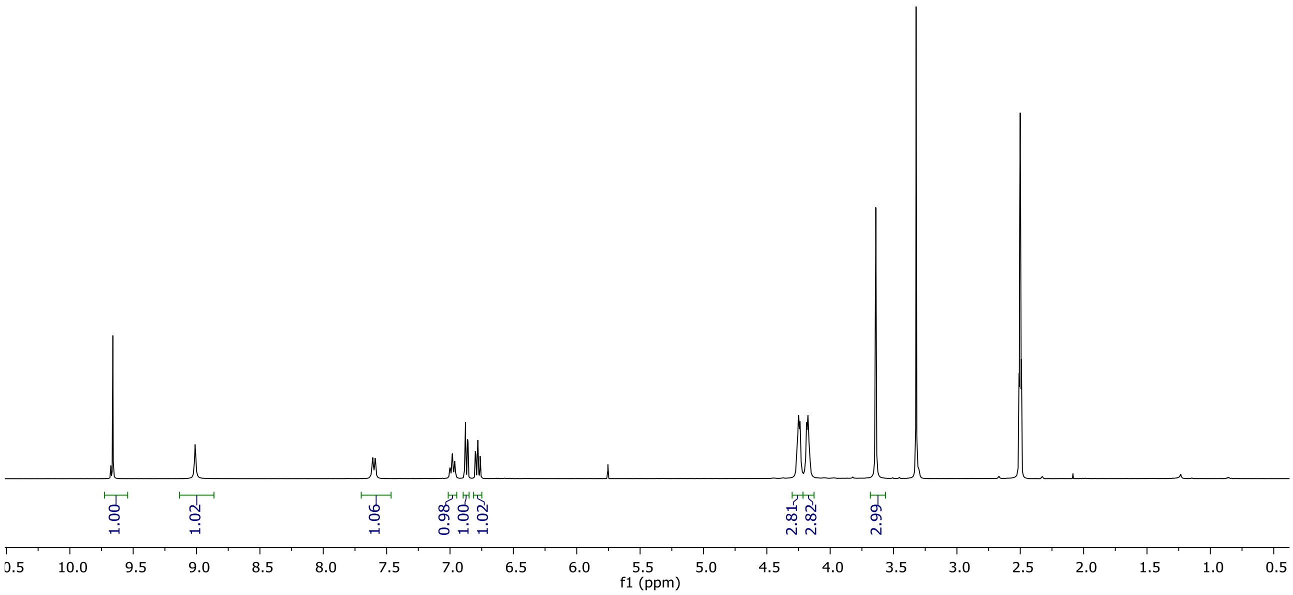
1.00
1.02
1.06
0.98
1.00
1.02
2.81
2.82
2.99
10.0
9.5
9.0
8.5
8.0
7.5
7.0
6.5
6.0
5.5
5.0
4.5
4.0
3.5
3.0
2.5
2.0
1.5
1.0
0.5
f1 (ppm)

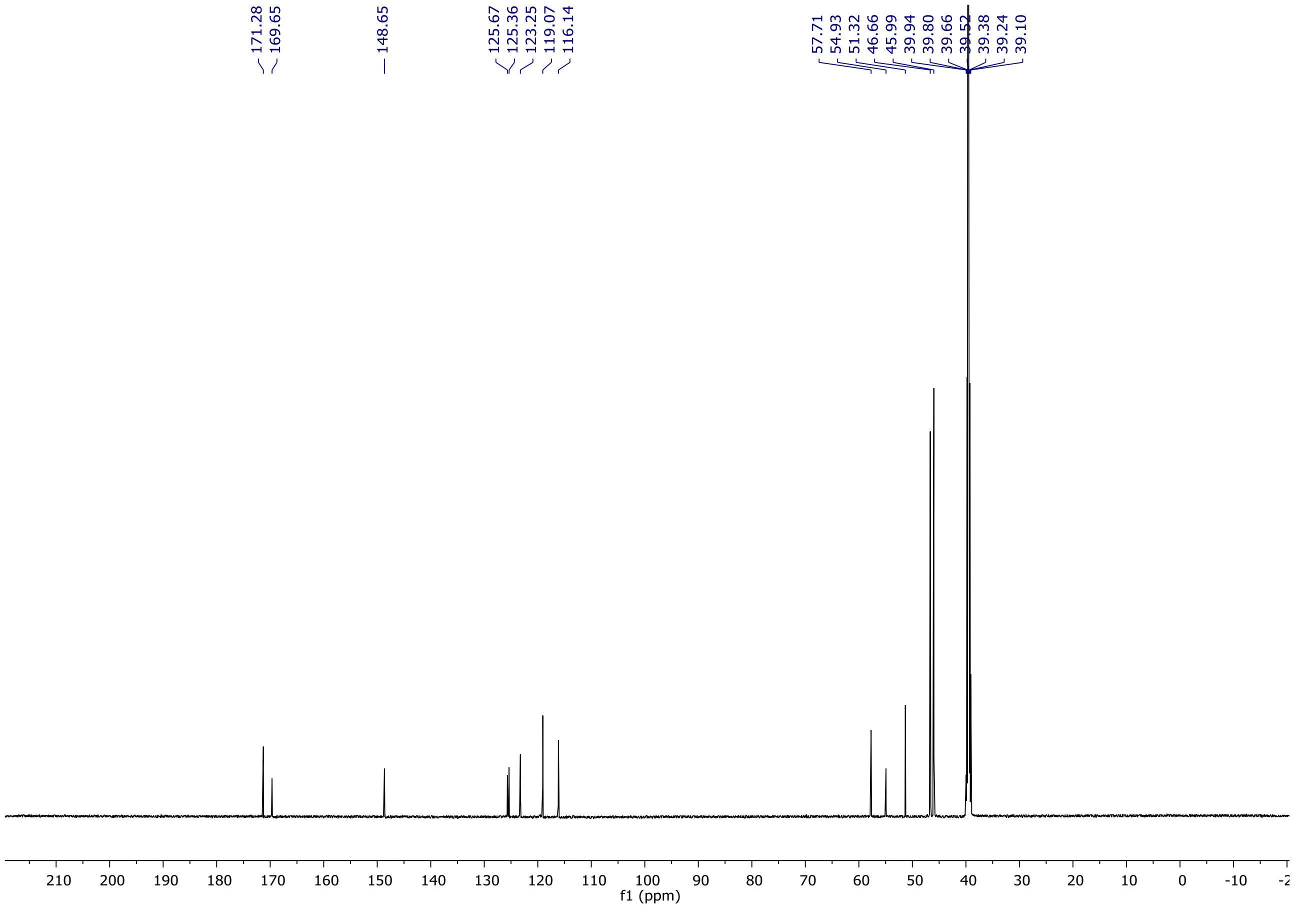
171.28
169.65
148.65
125.67
125.36
123.25
119.07
116.14
57.71
54.93
51.32
46.66
45.99
39.94
39.80
39.66
39.52
39.38
39.24
39.10
210
200
190
180
170
160
150
140
130
120
110
100
90
80
70
60
50
40
30
20
10
0
-10
f1 (ppm)

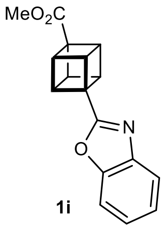

1i

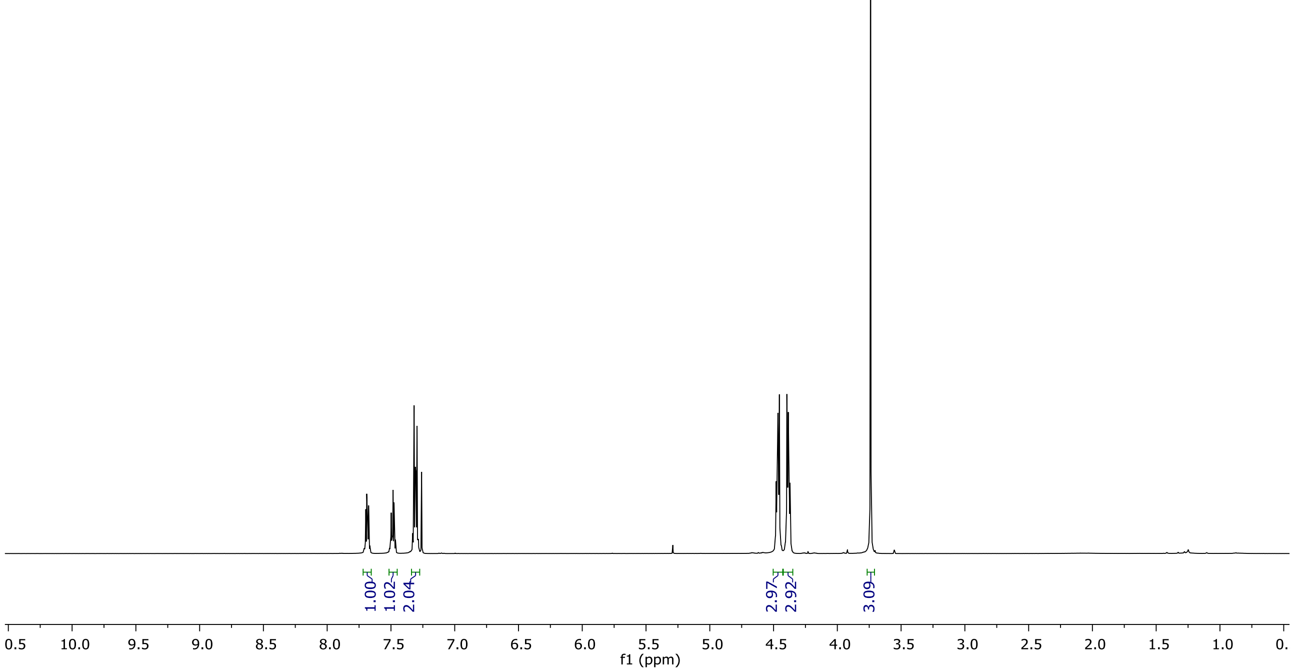
1.00
1.02
2.04
2.97
2.92
3.09
0.5 10.0 9.5 9.0 8.5 8.0 7.5 7.0 6.5 6.0 5.5 5.0 4.5 4.0 3.5 3.0 2.5 2.0 1.5 1.0 0.
f1 (ppm)

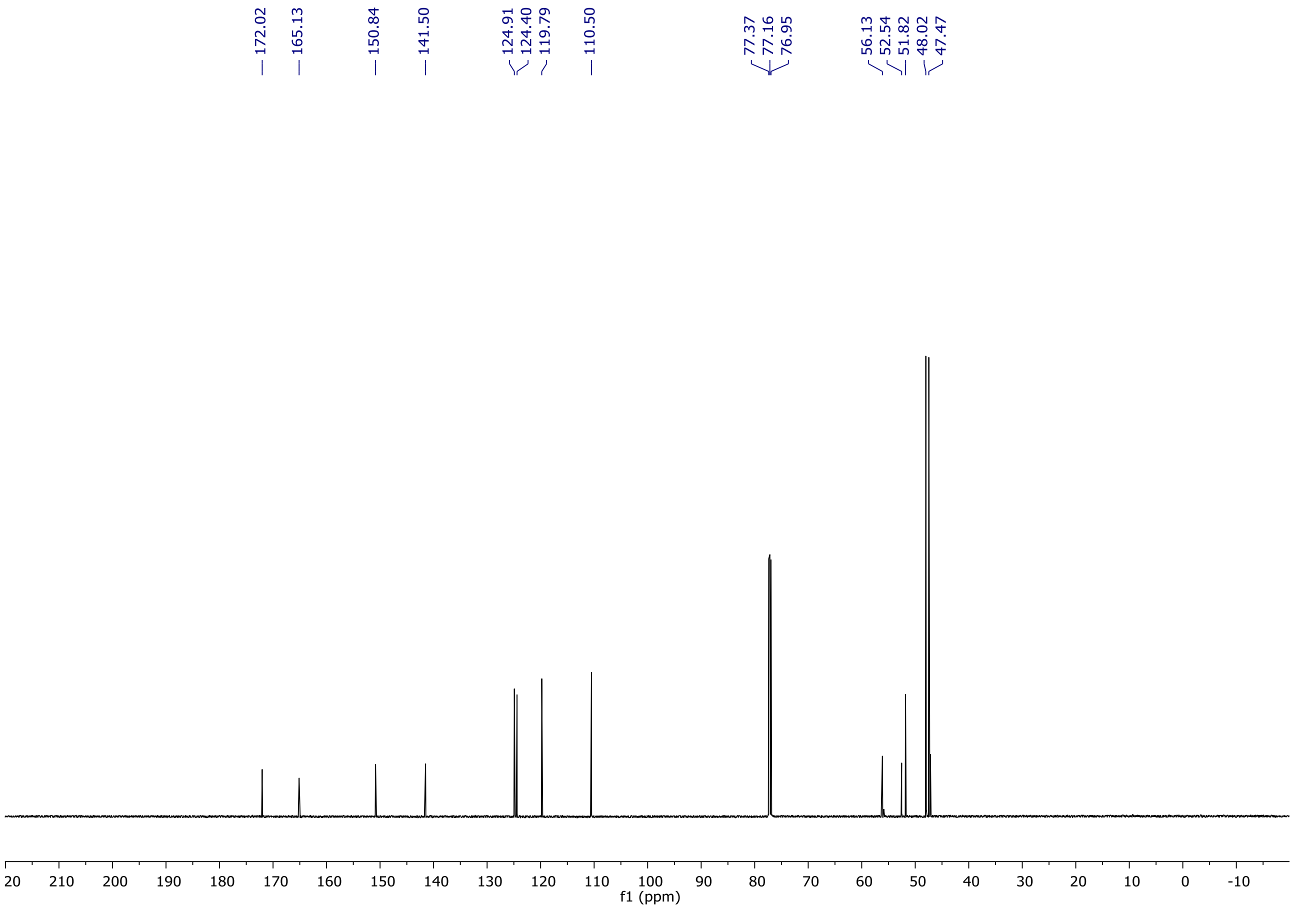
172.02
165.13
150.84
141.50
124.91
124.40
119.79
110.50
77.37
77.16
76.95
56.13
52.54
51.82
48.02
47.47
20 210 200 190 180 170 160 150 140 130 120 110 100 90 80 70 60 50 40 30 20 10 0 -10
f1 (ppm)

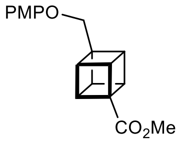
PMPO
CO2Me

1k

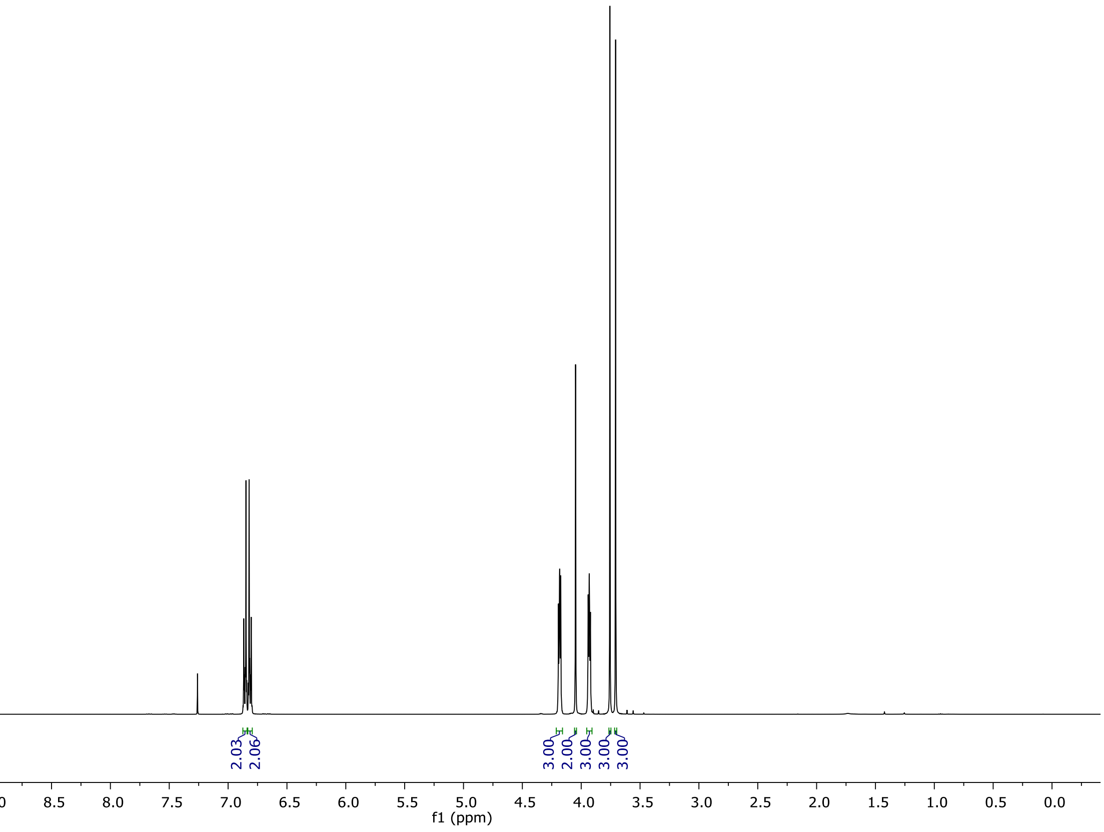
2.03
2.06
3.00
2.00
3.00
3.00
3.00
10.0 9.5 9.0 8.5 8.0 7.5 7.0 6.5 6.0 5.5 5.0 4.5 4.0 3.5 3.0 2.5 2.0 1.5 1.0 0.5 0.0
f1 (ppm)

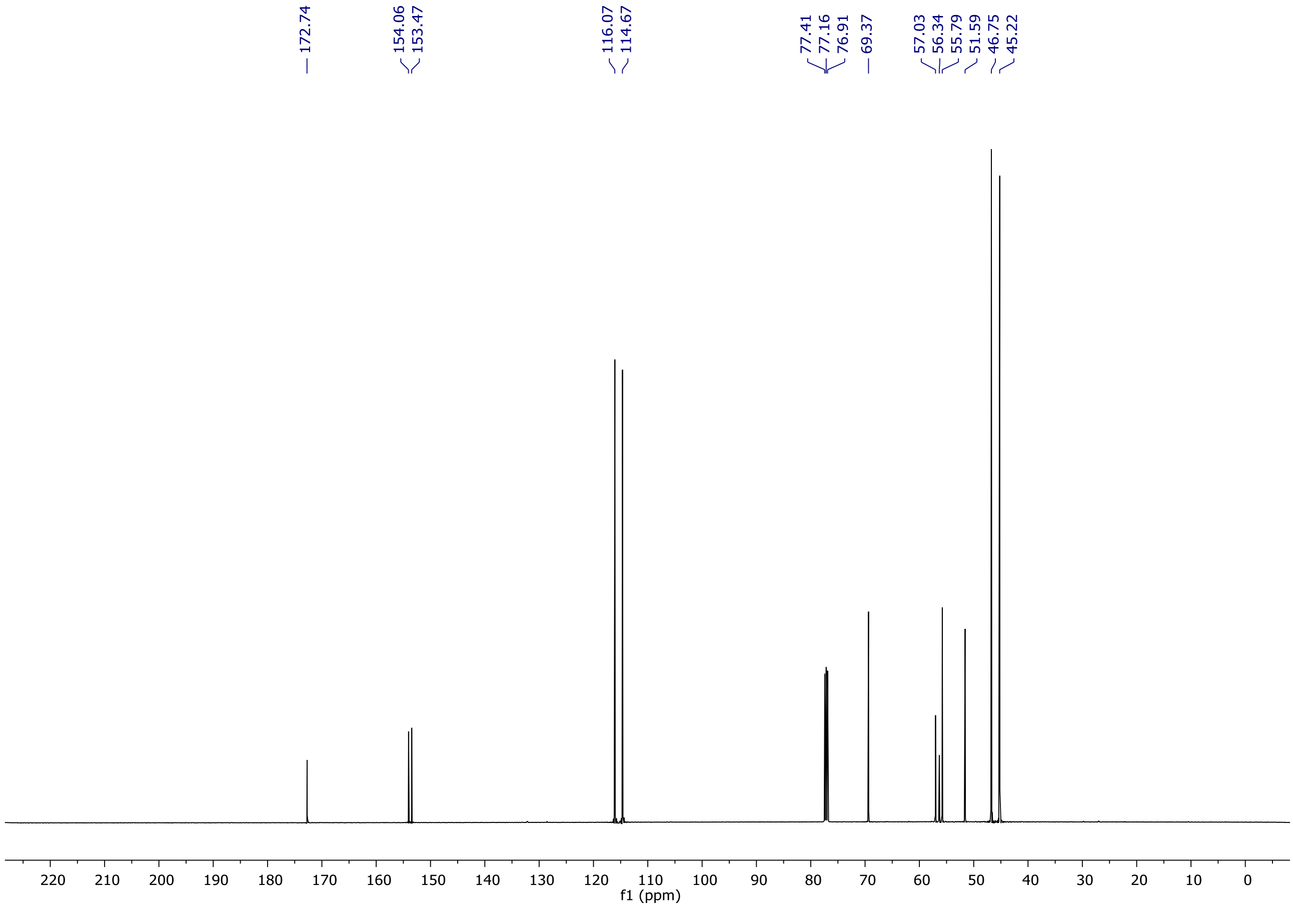
172.74
154.06
153.47
116.07
114.67
77.41
77.16
76.91
69.37
57.03
56.34
55.79
51.59
46.75
45.22
220 210 200 190 180 170 160 150 140 130 120 110 100 90 80 70 60 50 40 30 20 10 0
f1 (ppm)

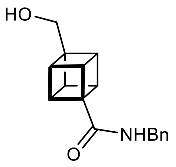
HO
NHBn
O

1l

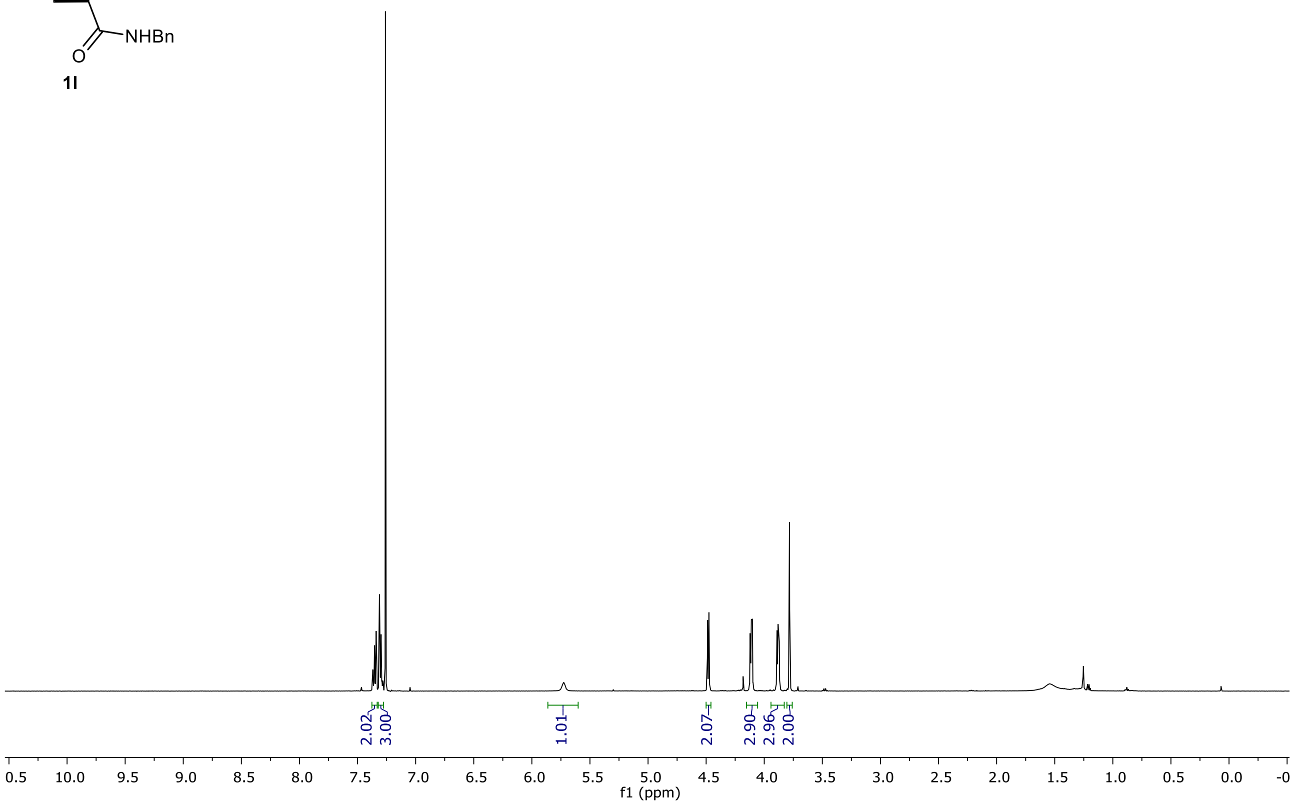
2.02
3.00
1.01
2.07
2.90
2.96
2.00
0.5 10.0 9.5 9.0 8.5 8.0 7.5 7.0 6.5 6.0 5.5 5.0 4.5 4.0 3.5 3.0 2.5 2.0 1.5 1.0 0.5 0.0 -0
f1 (ppm)

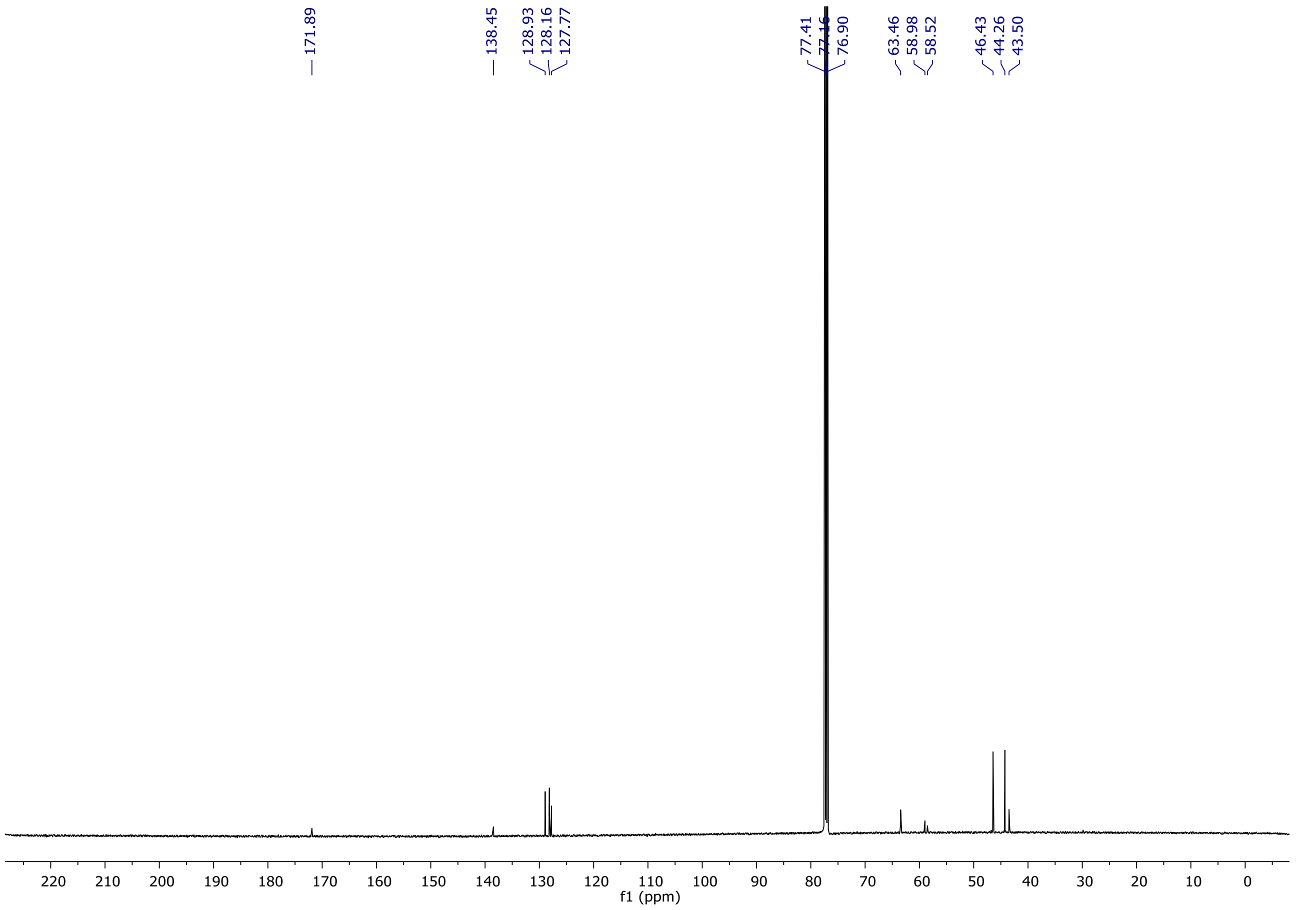
171.89
138.45
128.93
128.16
127.77
77.41
77.16
76.90
63.46
58.98
58.52
46.43
44.26
43.50
220 210 200 190 180 170 160 150 140 130 120 110 100 90 80 70 60 50 40 30 20 10 0
f1 (ppm)

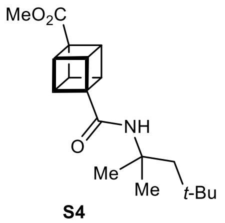
MeO2C
NH
O
Me
Me
t-Bu

S4

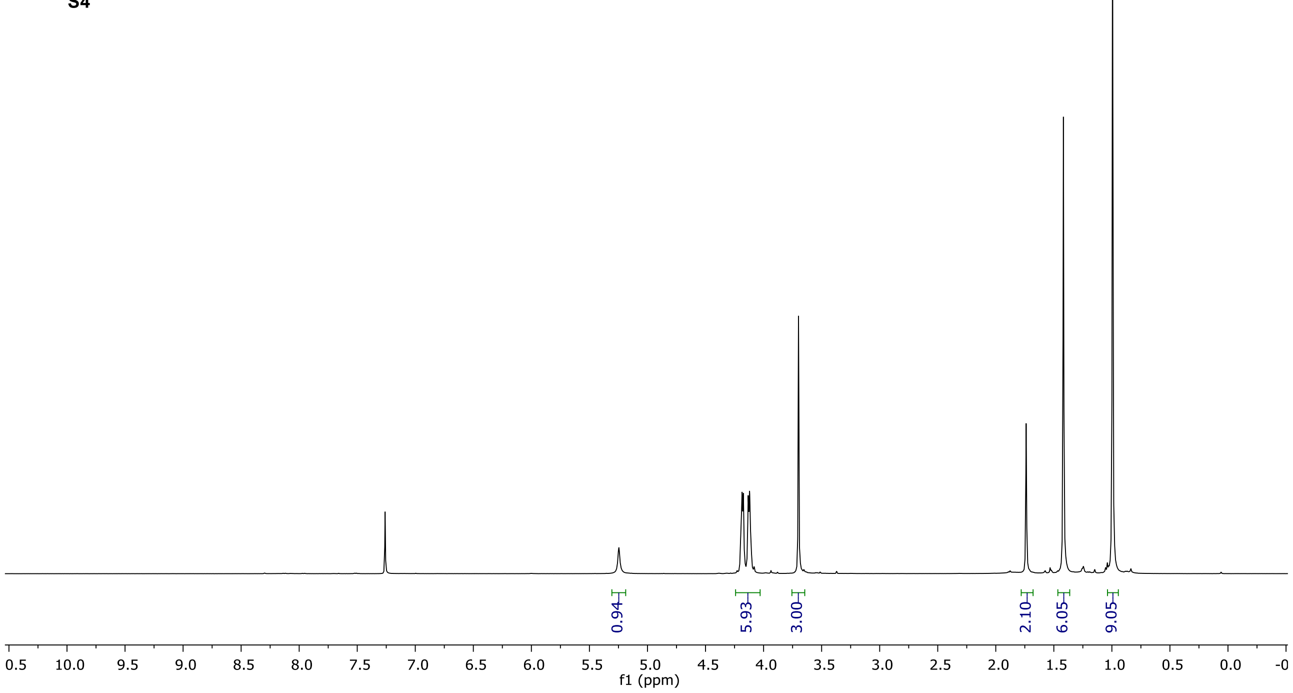
0.94
5.93
3.00
2.10
6.05
9.05
f1 (ppm)

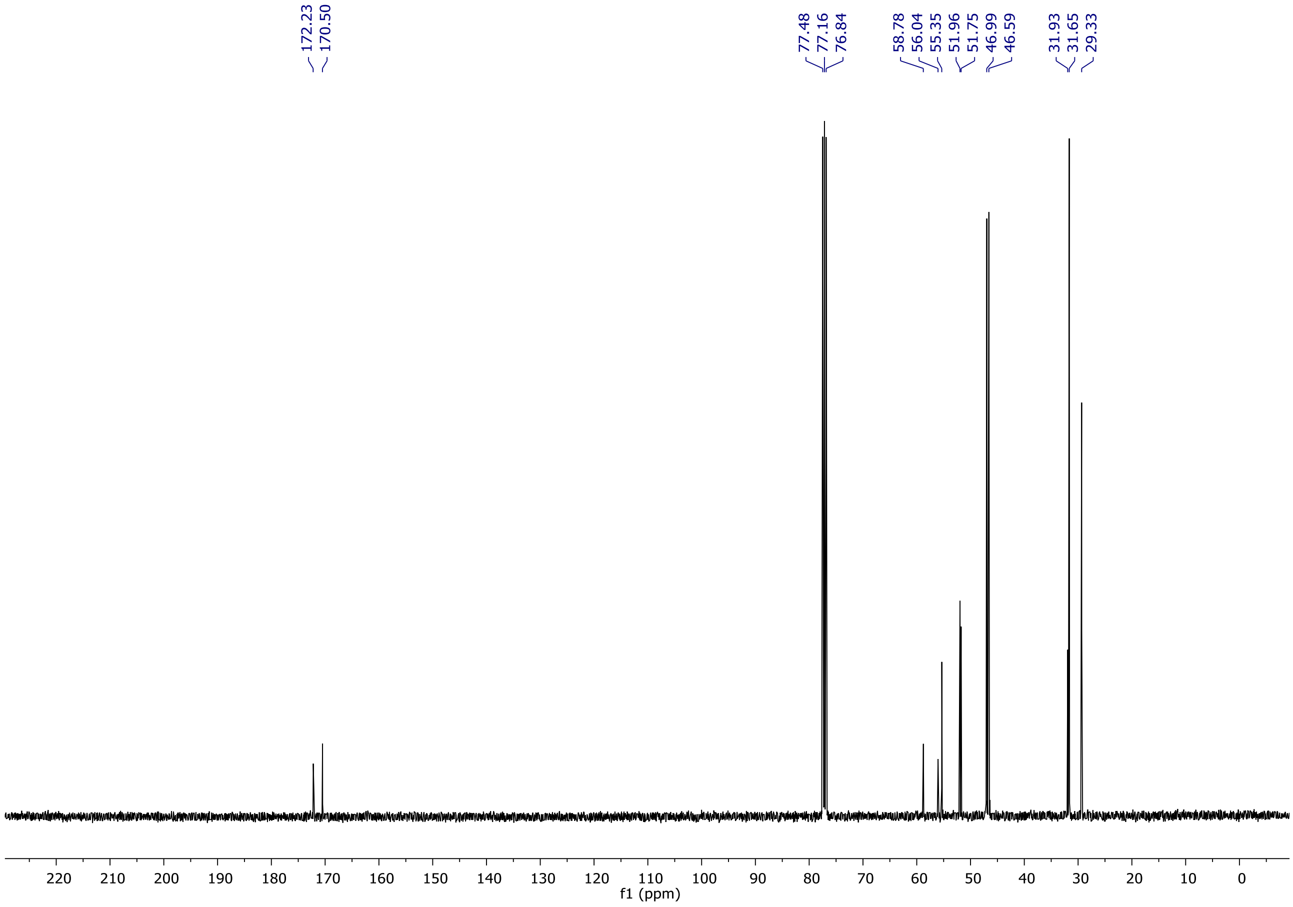
172.23
170.50
77.48
77.16
76.84
58.78
56.04
55.35
51.96
51.75
46.99
46.59
31.93
31.65
29.33
f1 (ppm)

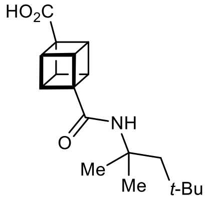
HO2C
NH
O
Me
Me
t-Bu

S5

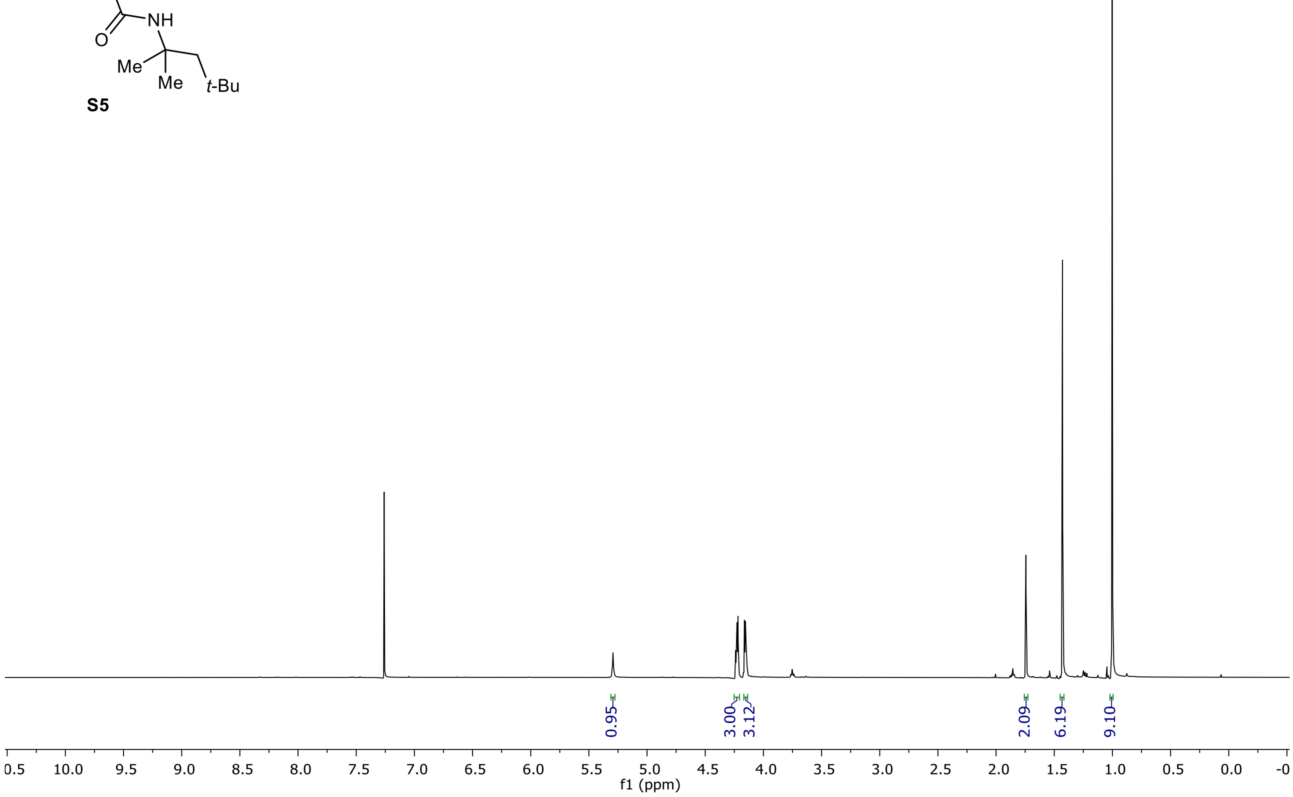
0.95
3.00
3.12
2.09
6.19
9.10
f1 (ppm)

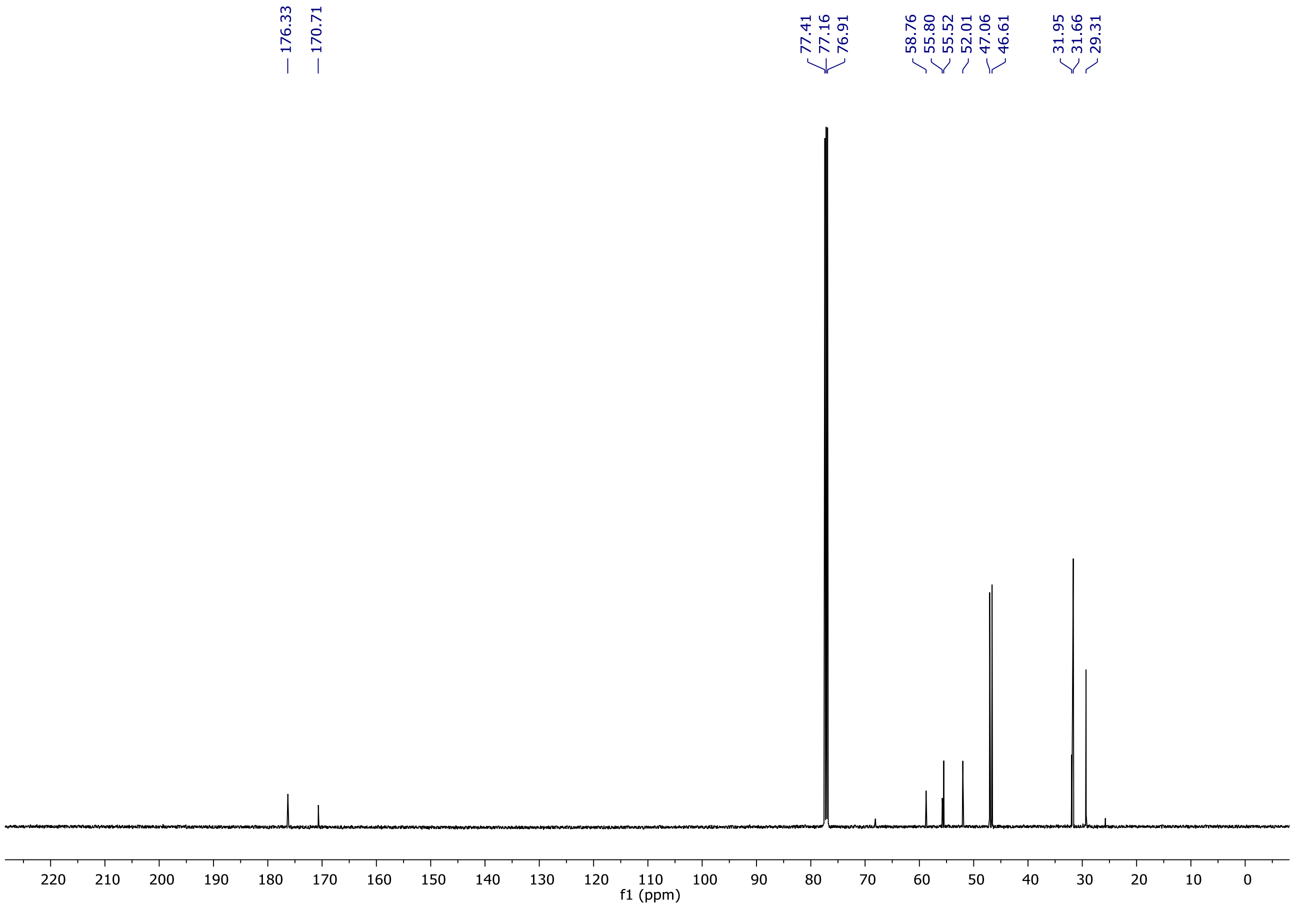
176.33
170.71
77.41
77.16
76.91
58.76
55.80
55.52
52.01
47.06
46.61
31.95
31.66
29.31
f1 (ppm)

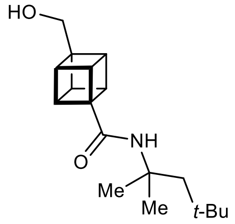
HO
NH
O
Me
Me
t-Bu

1m

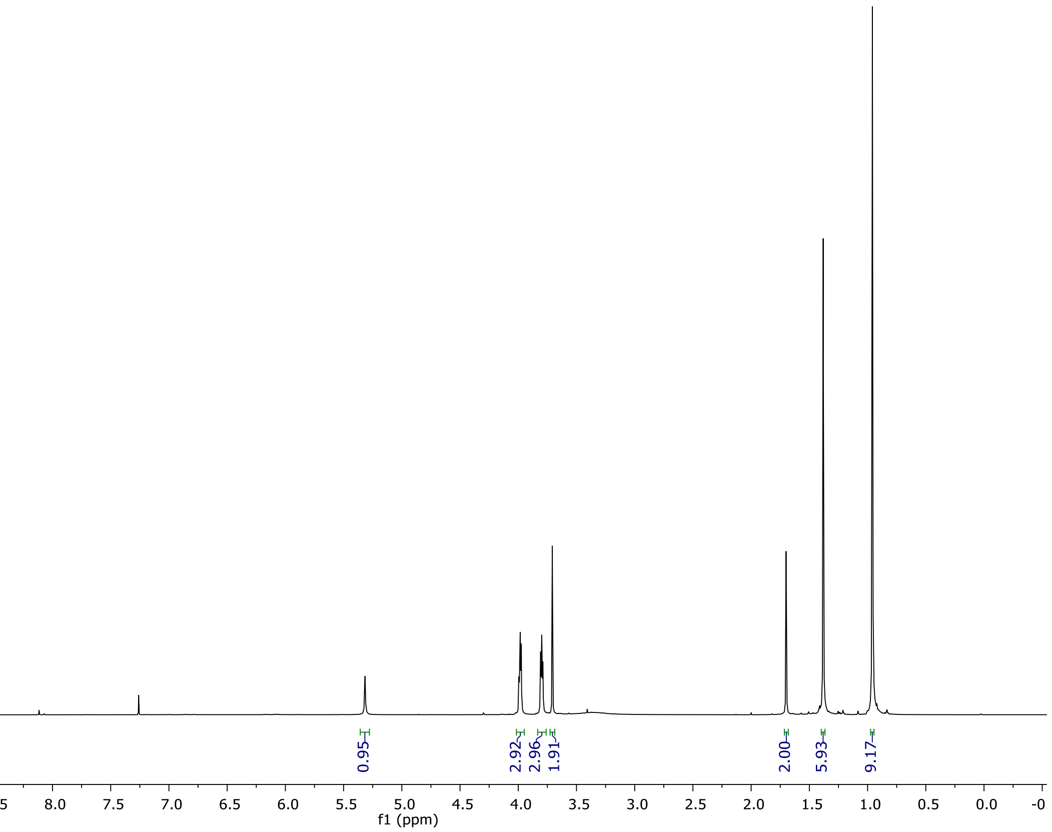
0.95
2.92
2.96
1.91
2.00
5.93
9.17
10.0 9.5 9.0 8.5 8.0 7.5 7.0 6.5 6.0 5.5 5.0 4.5 4.0 3.5 3.0 2.5 2.0 1.5 1.0 0.5 0.0 -0
f1 (ppm)

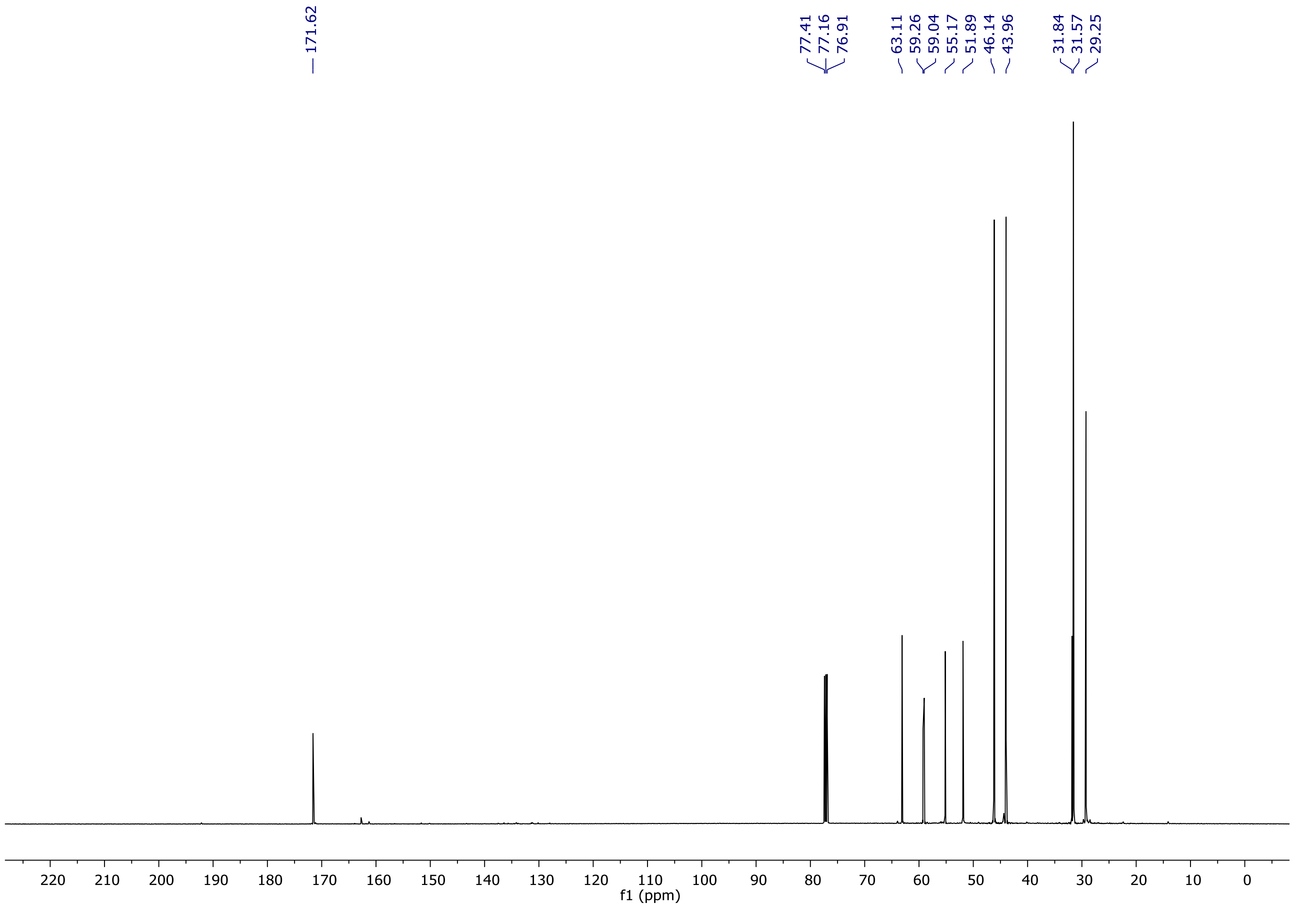
171.62
77.41
77.16
76.91
63.11
59.26
59.04
55.17
51.89
46.14
43.96
31.84
31.57
29.25
220 210 200 190 180 170 160 150 140 130 120 110 100 90 80 70 60 50 40 30 20 10 0
f1 (ppm)

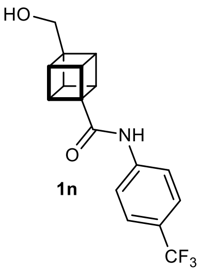
HO
NH
O
CF3

1n

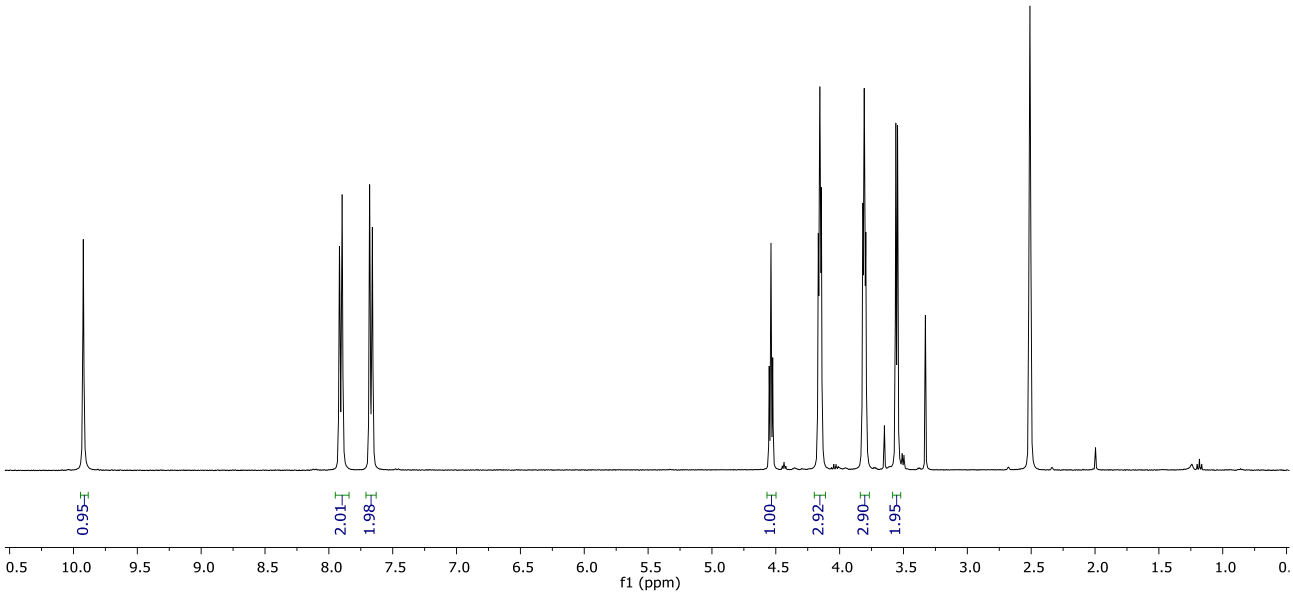
0.95
2.01
1.98
1.00
2.92
2.90
1.95
0.5 10.0 9.5 9.0 8.5 8.0 7.5 7.0 6.5 6.0 5.5 5.0 4.5 4.0 3.5 3.0 2.5 2.0 1.5 1.0 0.
f1 (ppm)

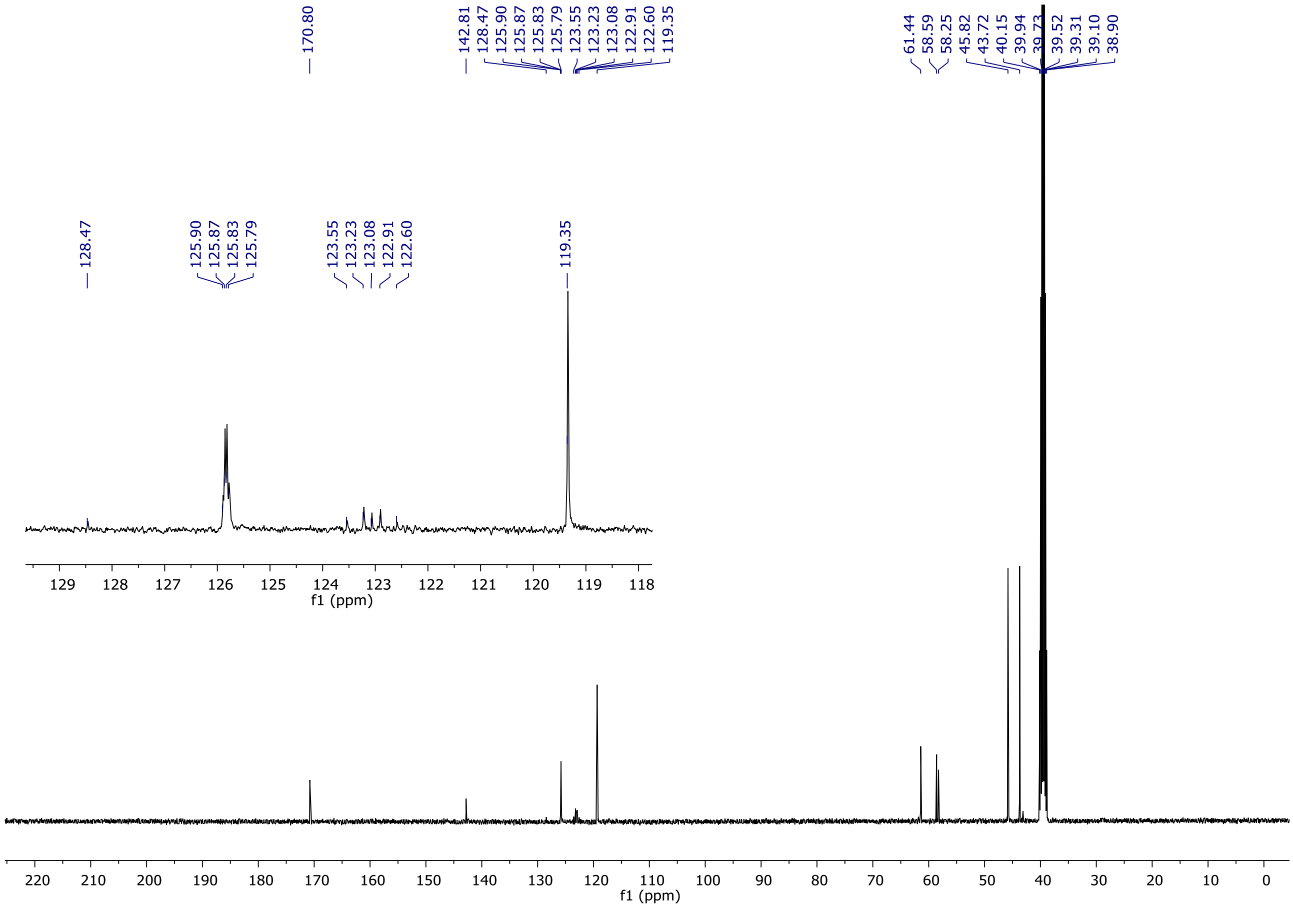
170.80
142.81
128.47
125.90
125.87
125.83
125.79
123.55
123.23
123.08
122.91
122.60
119.35
61.44
58.59
58.25
45.82
43.72
40.15
39.94
39.73
39.52
39.31
39.10
38.90
128.47
125.90
125.87
125.83
125.79
123.55
123.23
123.08
122.91
122.60
119.35
129 128 127 126 125 124 123 122 121 120 119 118
f1 (ppm)
220 210 200 190 180 170 160 150 140 130 120 110 100 90 80 70 60 50 40 30 20 10 0
f1 (ppm)

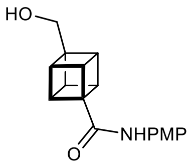
HO
NHPMP
O

1o

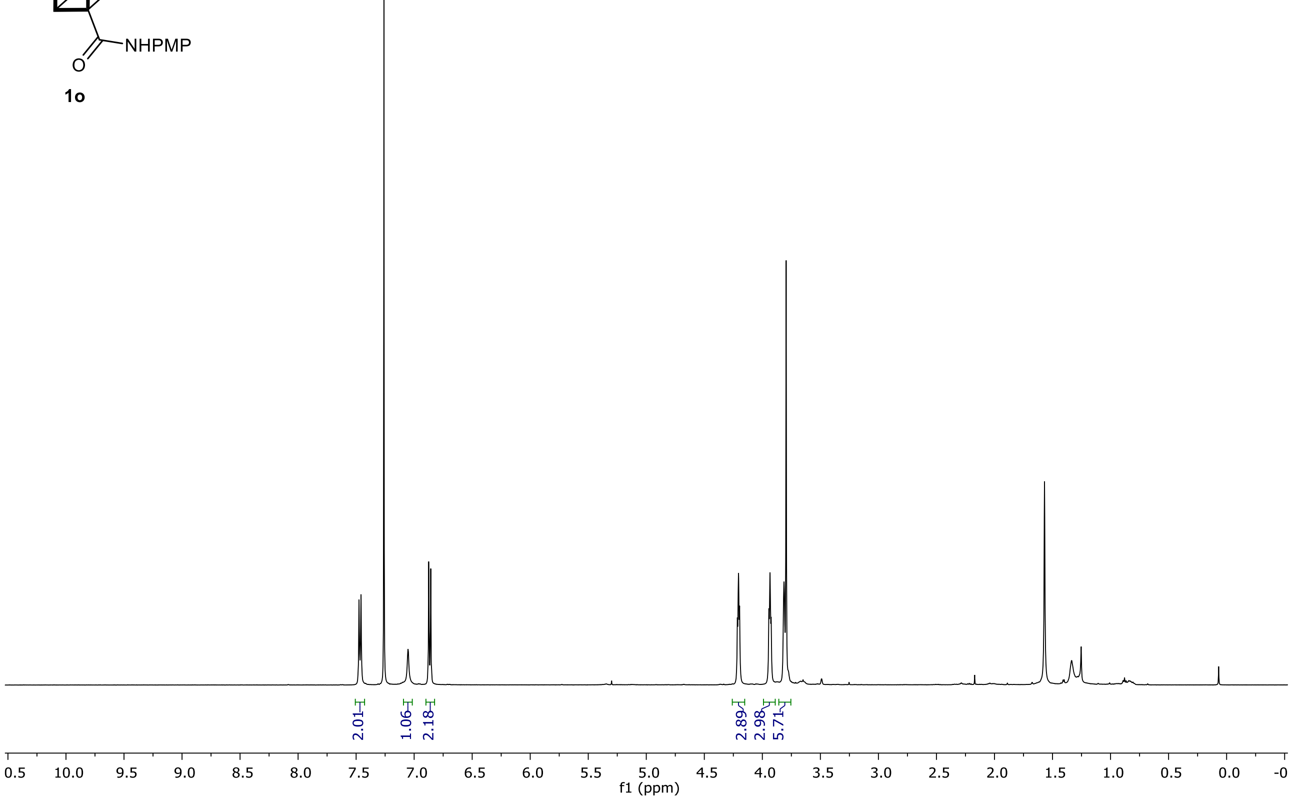
2.01
1.06
2.18
2.89
2.98
5.71
f1 (ppm)

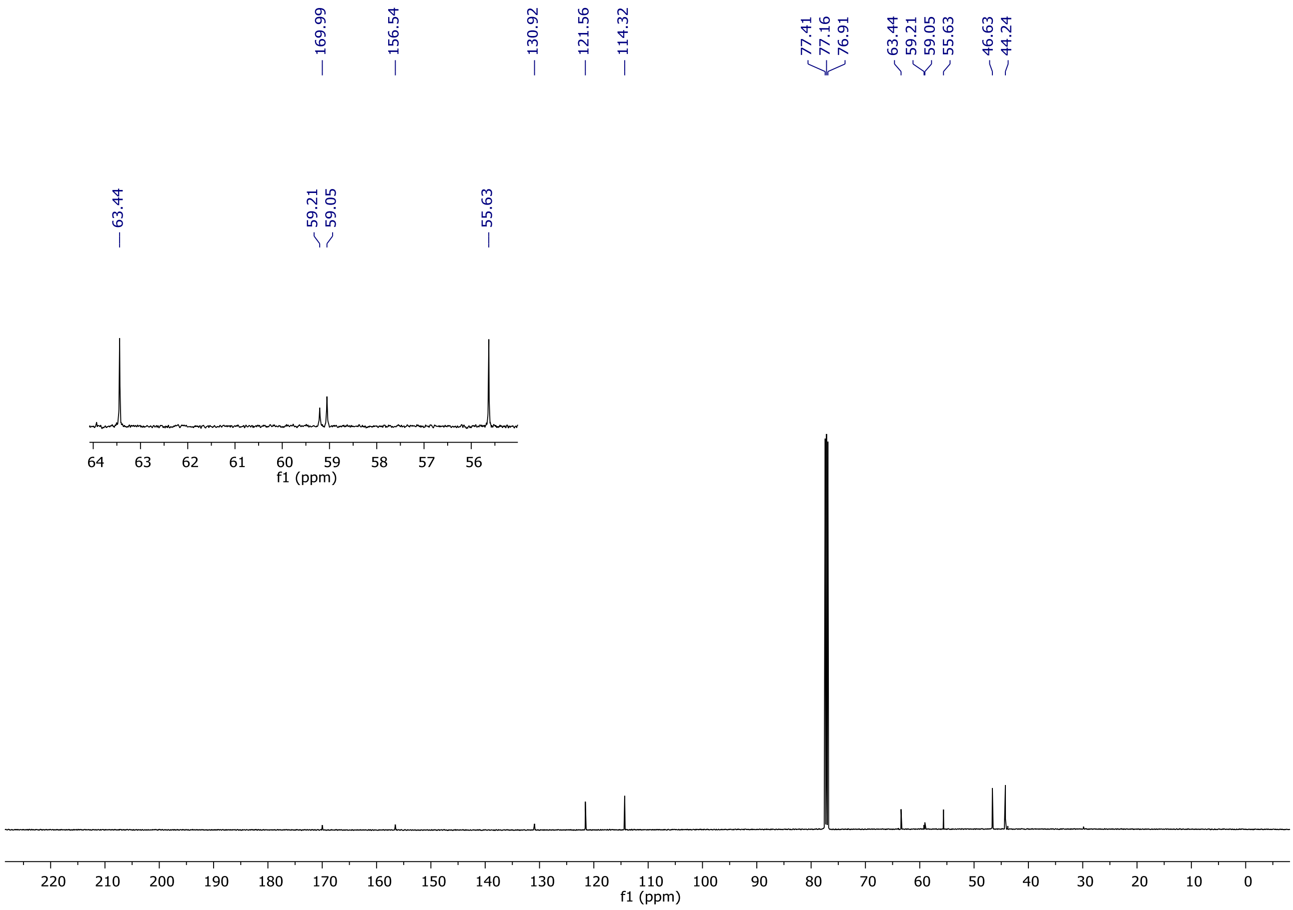
169.99
156.54
130.92
121.56
114.32
77.41
77.16
76.91
63.44
59.21
59.05
55.63
46.63
44.24
63.44
59.21
59.05
55.63
f1 (ppm)
f1 (ppm)

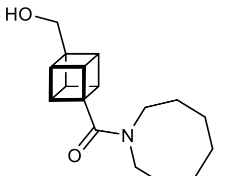
HO
N
O

1p

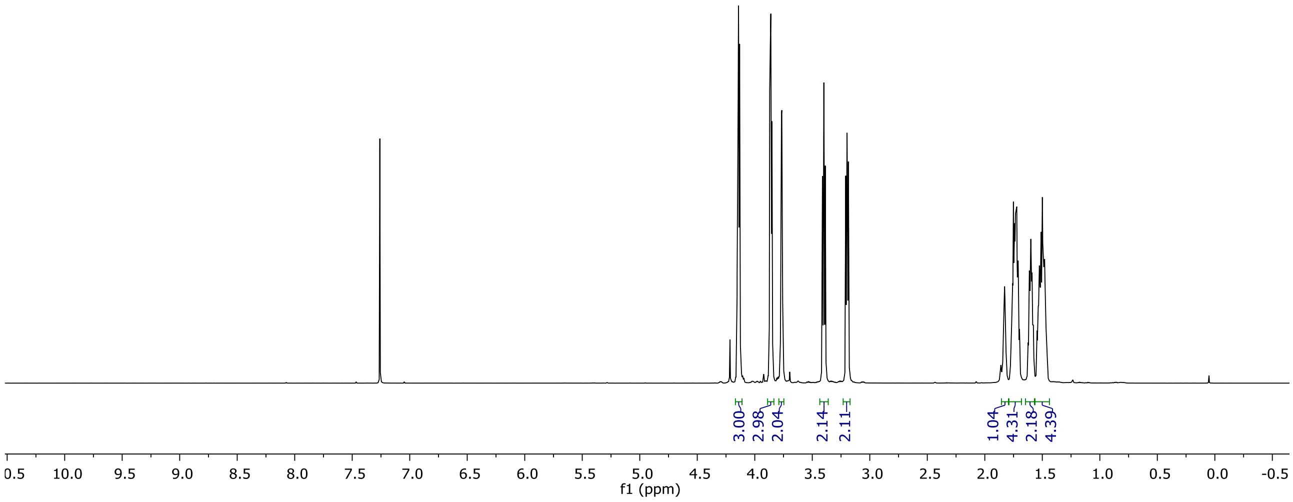
3.00
2.98
2.04
2.14
2.11
1.04
4.31
2.18
4.39
0.5 10.0 9.5 9.0 8.5 8.0 7.5 7.0 6.5 6.0 5.5 5.0 4.5 4.0 3.5 3.0 2.5 2.0 1.5 1.0 0.5 0.0 -0.5
f1 (ppm)

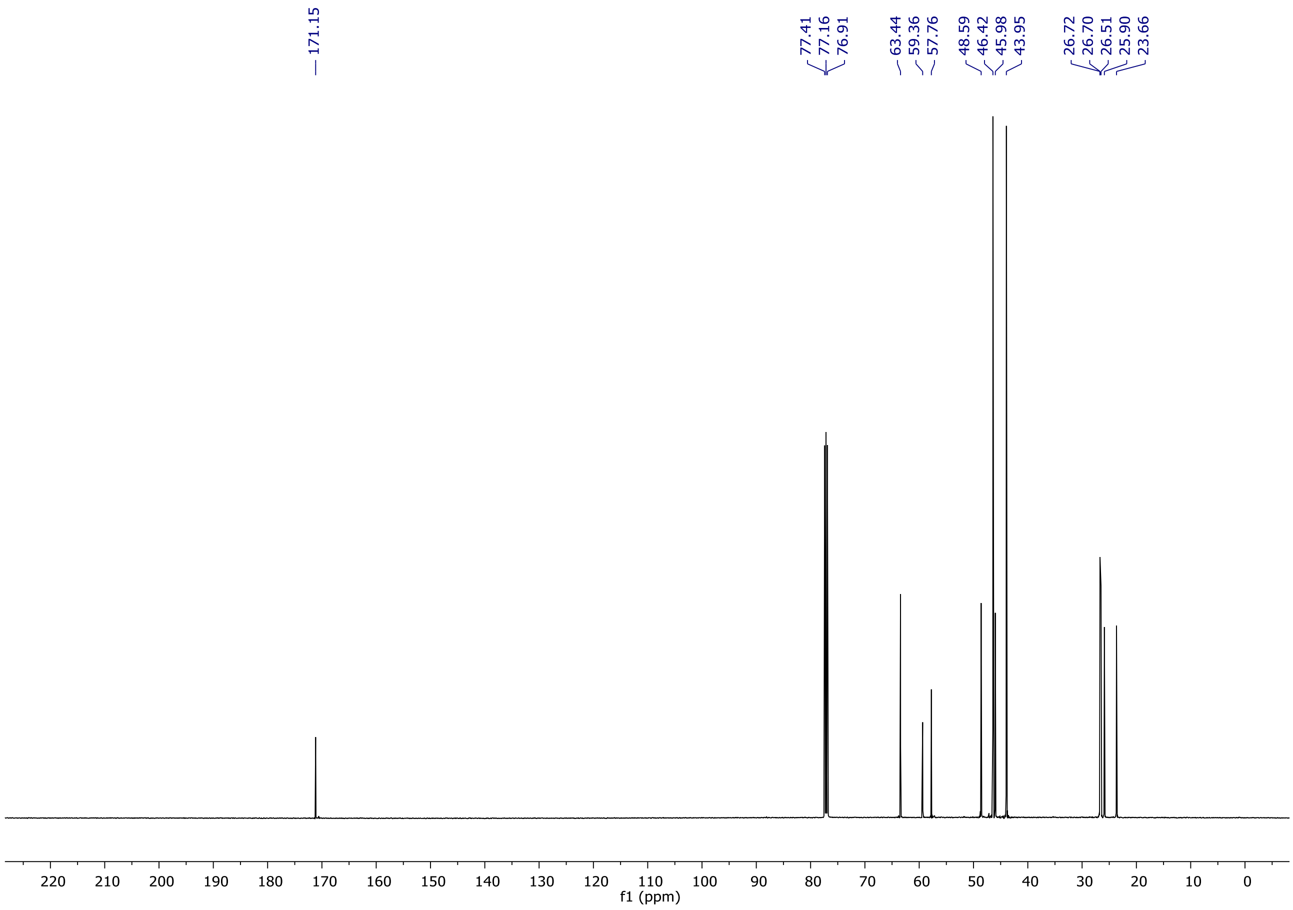
171.15
77.41
77.16
76.91
63.44
59.36
57.76
48.59
46.42
45.98
43.95
26.72
26.70
26.51
25.90
23.66
220 210 200 190 180 170 160 150 140 130 120 110 100 90 80 70 60 50 40 30 20 10 0
f1 (ppm)

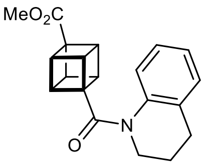
MeO2C
N
O

S6

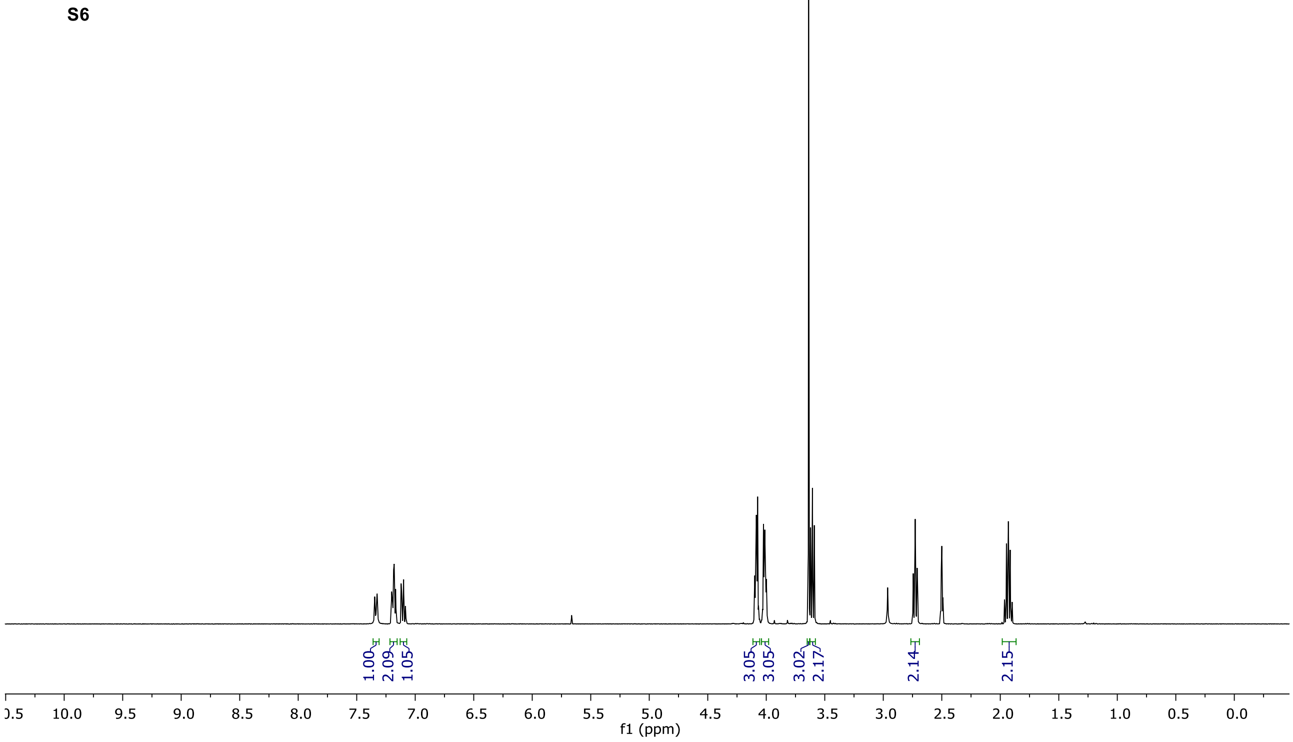
1.00
2.09
1.05
3.05
3.05
3.02
2.17
2.14
2.15
f1 (ppm)

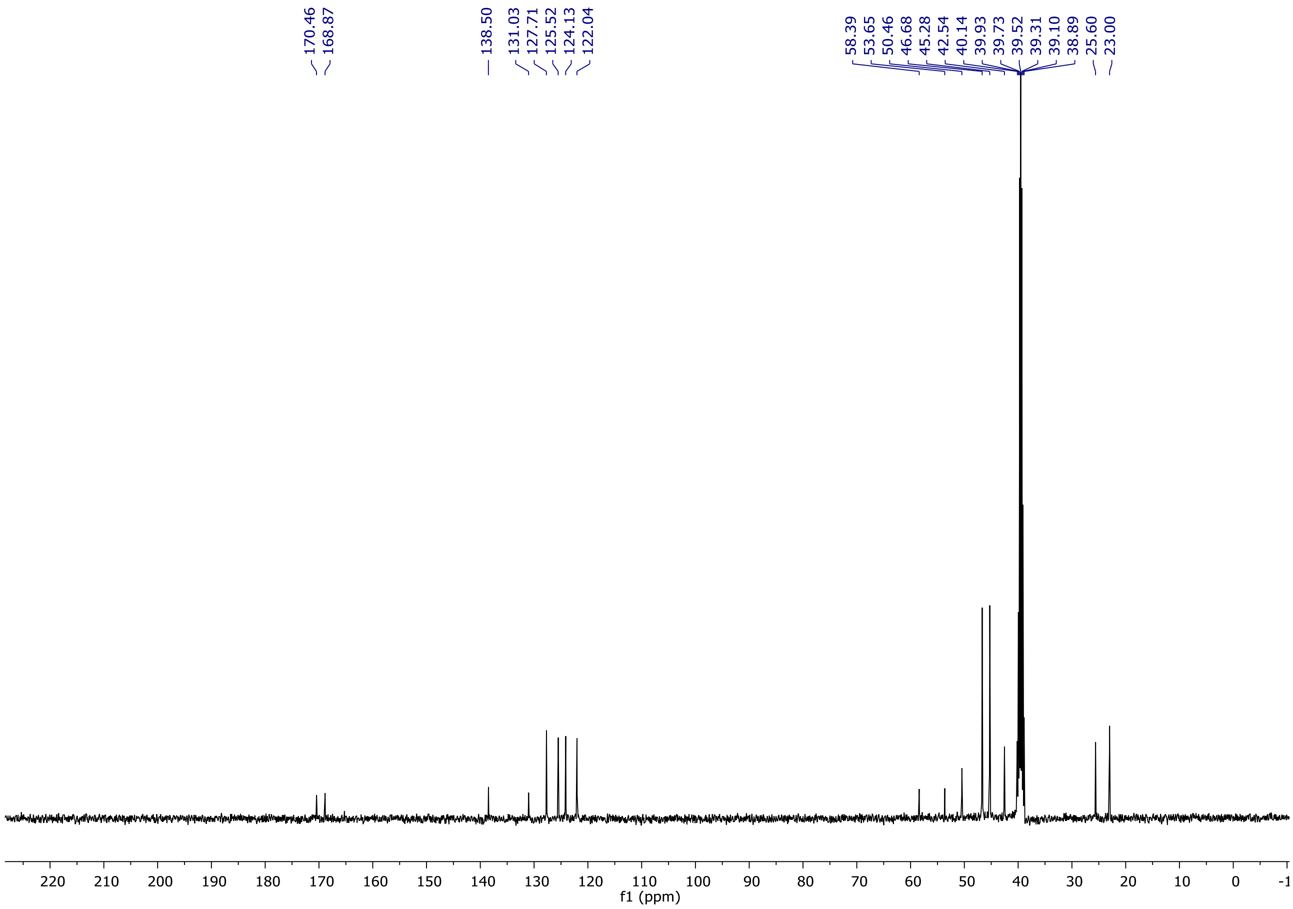
170.46
168.87
138.50
131.03
127.71
125.52
124.13
122.04
58.39
53.65
50.46
46.68
45.28
42.54
40.14
39.93
39.73
39.52
39.31
39.10
38.89
25.60
23.00
f1 (ppm)

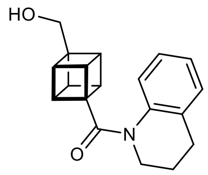

HO
N
O

1q

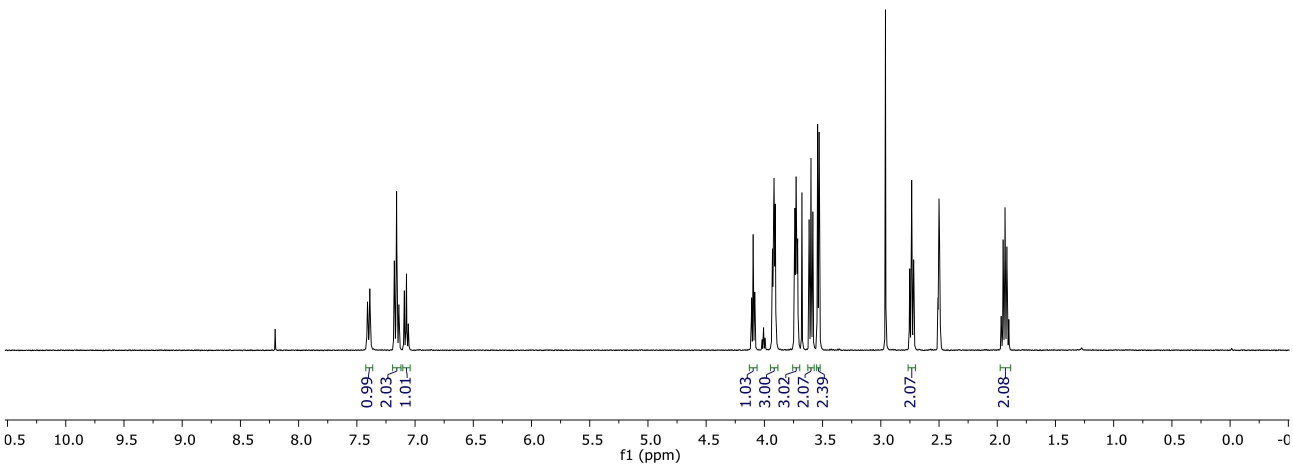

0.99
2.03
1.01
1.03
3.00
3.02
2.07
2.39
2.07
2.08
f1 (ppm)

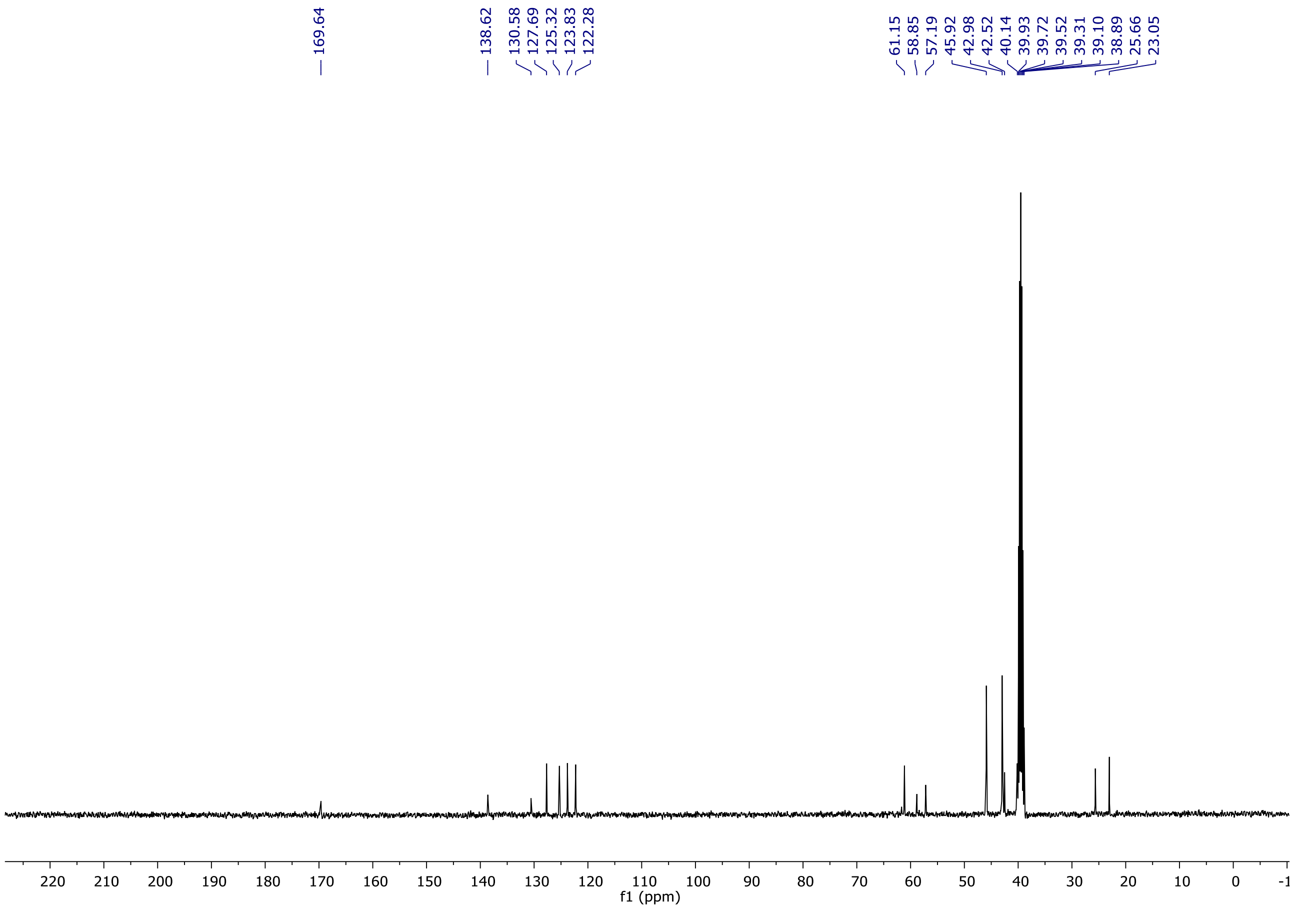

169.64
138.62
130.58
127.69
125.32
123.83
122.28
61.15
58.85
57.19
45.92
42.98
42.52
40.14
39.93
39.72
39.52
39.31
39.10
38.89
25.66
23.05
f1 (ppm)

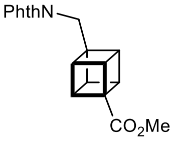
PhthN
CO2Me

1t

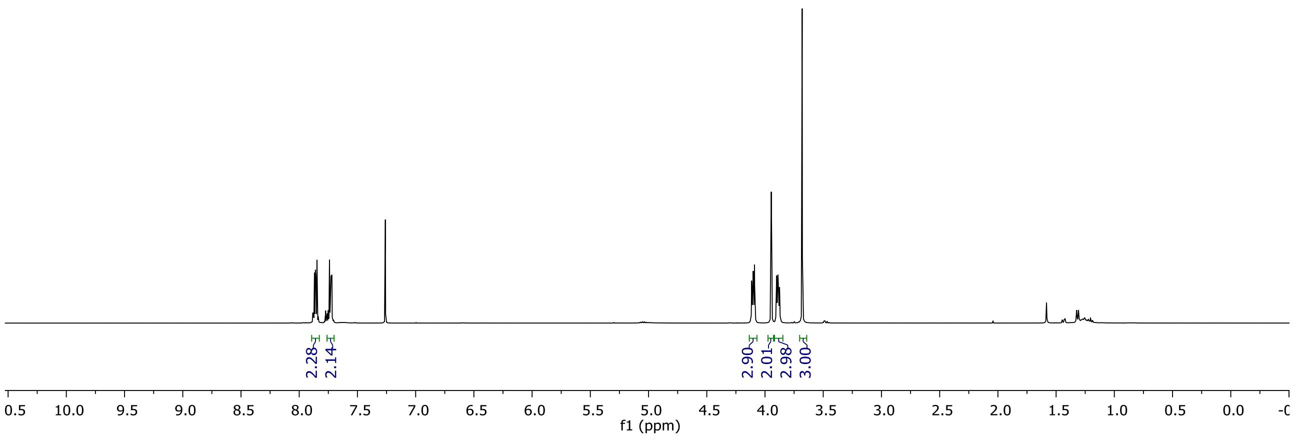
2.28
2.14
2.90
2.01
2.98
3.00
f1 (ppm)

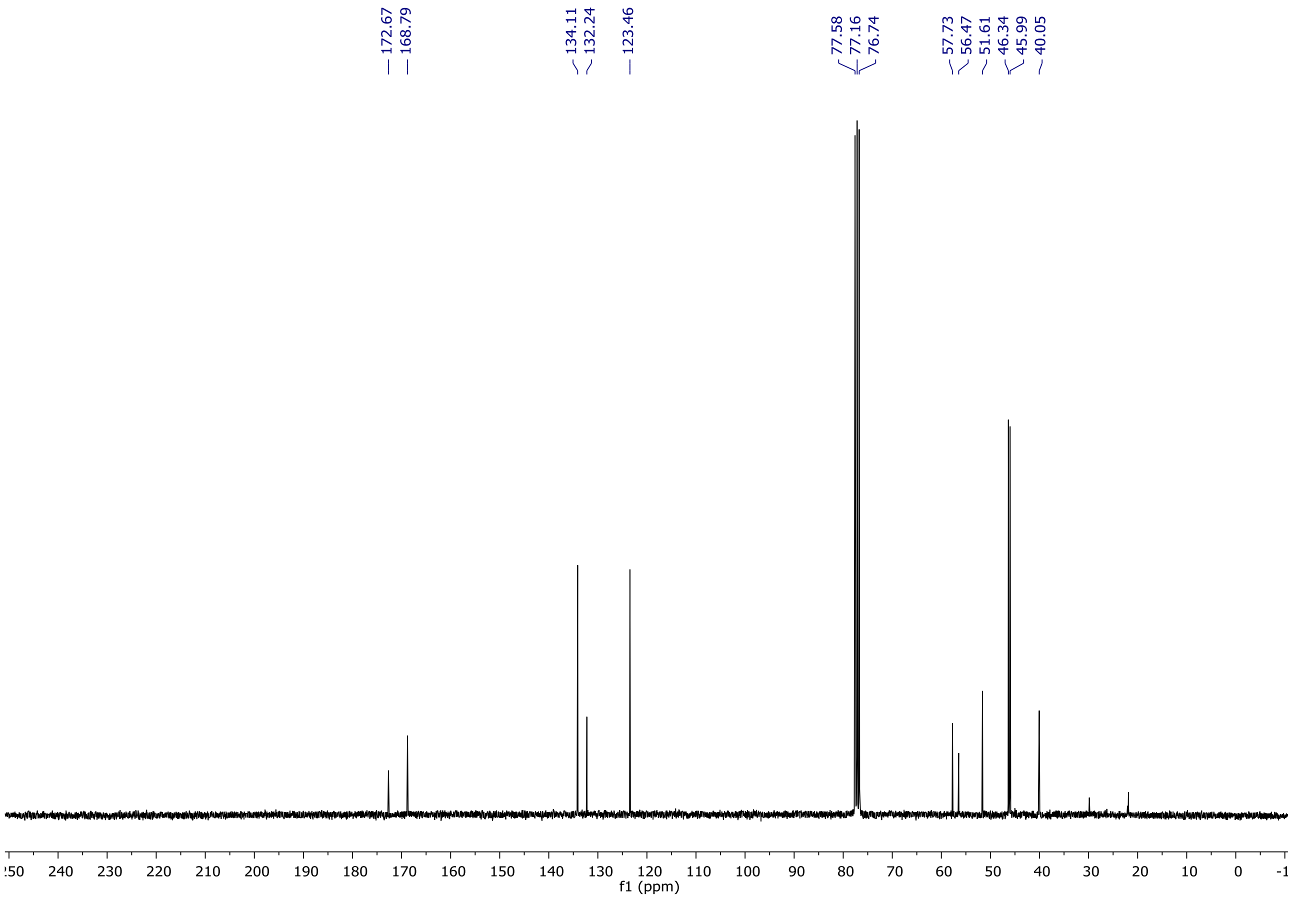
172.67
168.79
134.11
132.24
123.46
77.58
77.16
76.74
57.73
56.47
51.61
46.34
45.99
40.05
f1 (ppm)

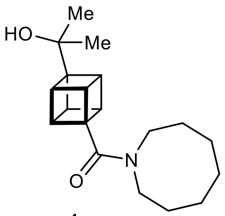

HO
Me
Me
N
O

1v

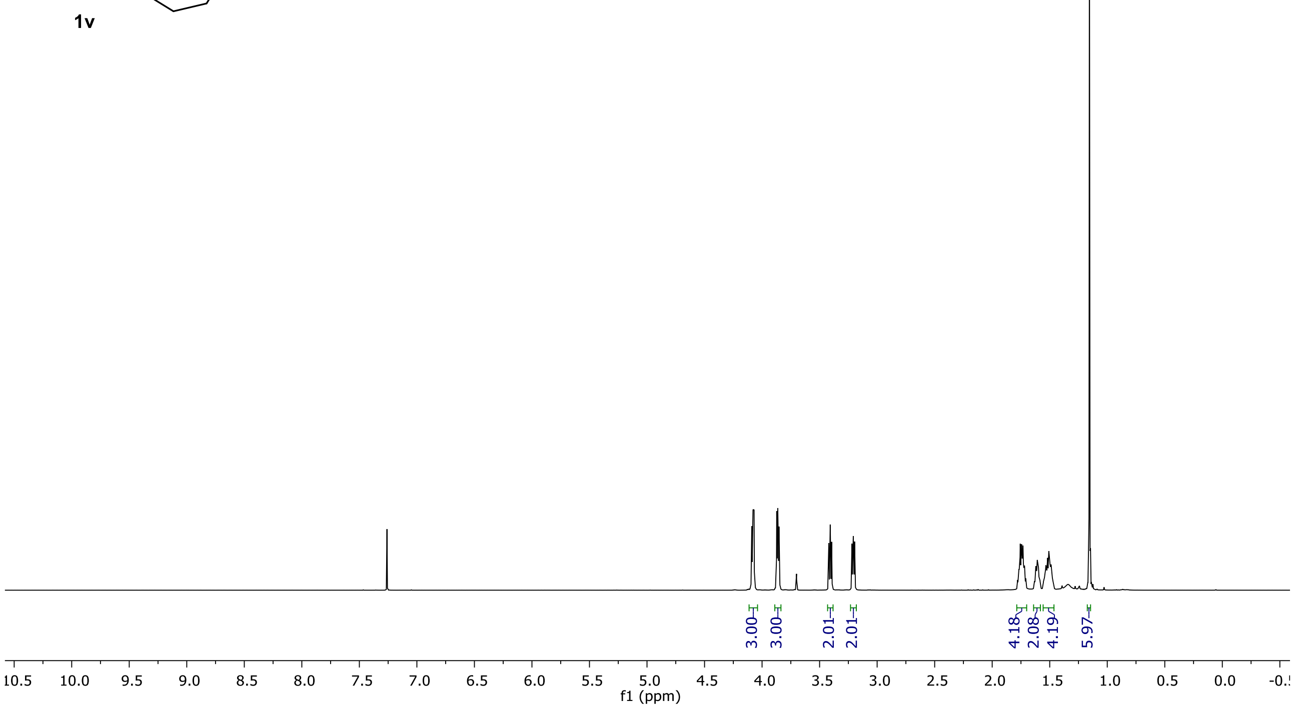

3.00
3.00
2.01
2.01
4.18
2.08
4.19
5.97
f1 (ppm)

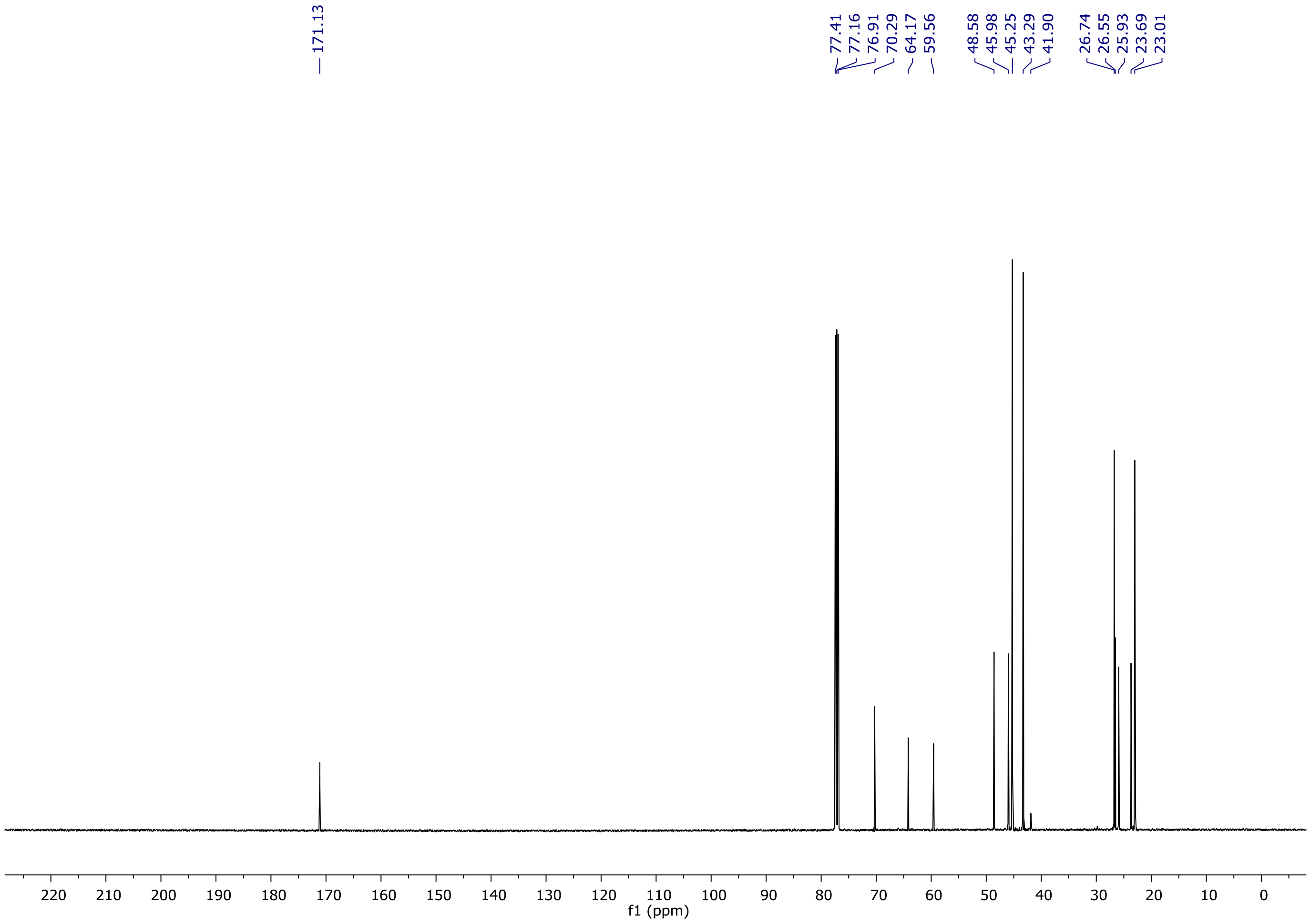

171.13
77.41
77.16
76.91
70.29
64.17
59.56
48.58
45.98
45.25
43.29
41.90
26.74
26.55
25.93
23.69
23.01
f1 (ppm)

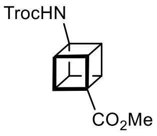
TrocHN
CO_2Me

1w

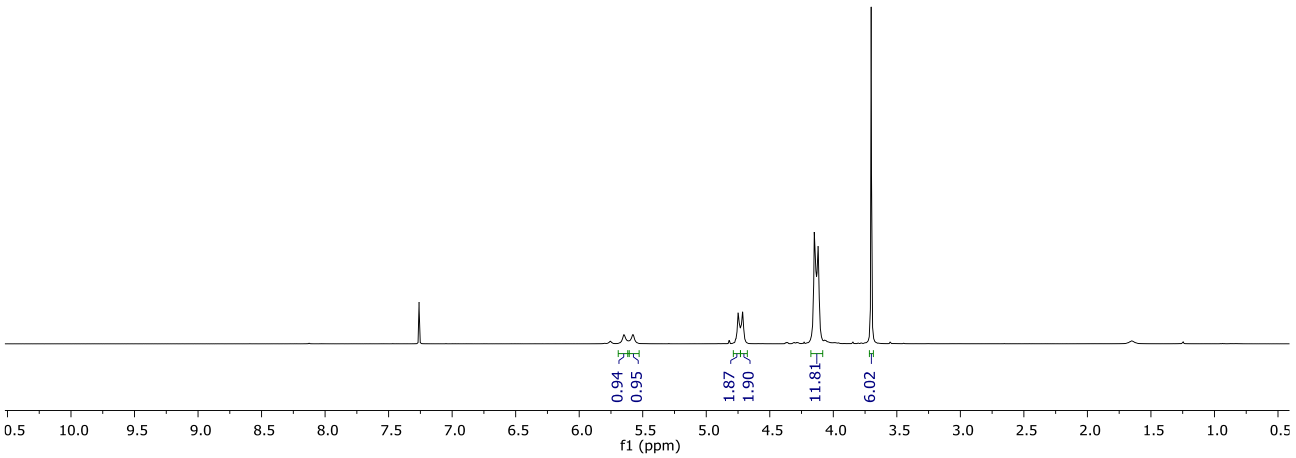
0.94
0.95
1.87
1.90
11.81
6.02
10.5 10.0 9.5 9.0 8.5 8.0 7.5 7.0 6.5 6.0 5.5 5.0 4.5 4.0 3.5 3.0 2.5 2.0 1.5 1.0 0.5
f1 (ppm)

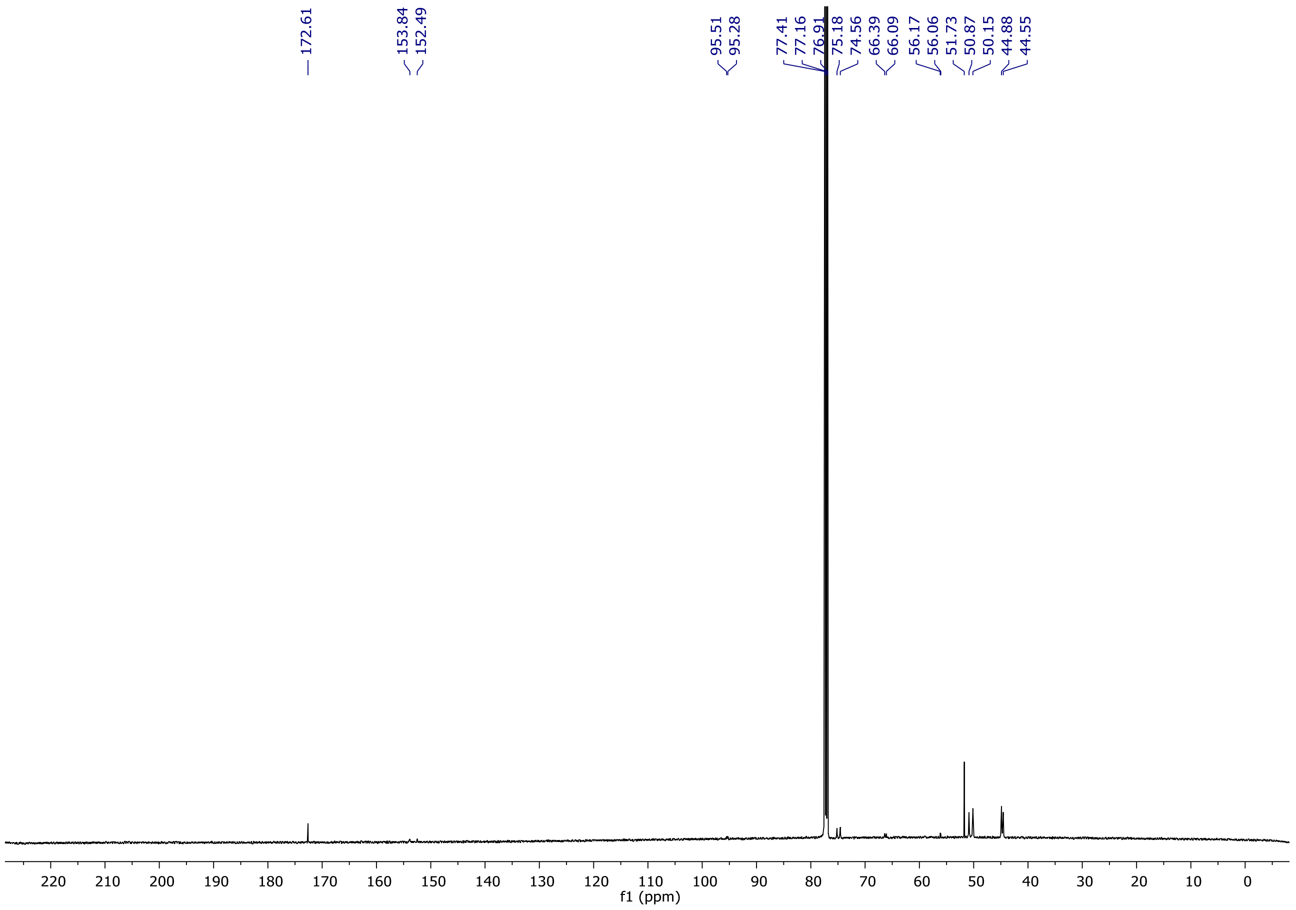
172.61
153.84
152.49
95.51
95.28
77.41
77.16
76.91
75.18
74.56
66.39
66.09
56.17
56.06
51.73
50.87
50.15
44.88
44.55
220 210 200 190 180 170 160 150 140 130 120 110 100 90 80 70 60 50 40 30 20 10 0
f1 (ppm)

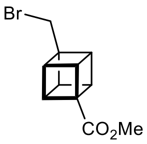
Br
CO2Me

1x

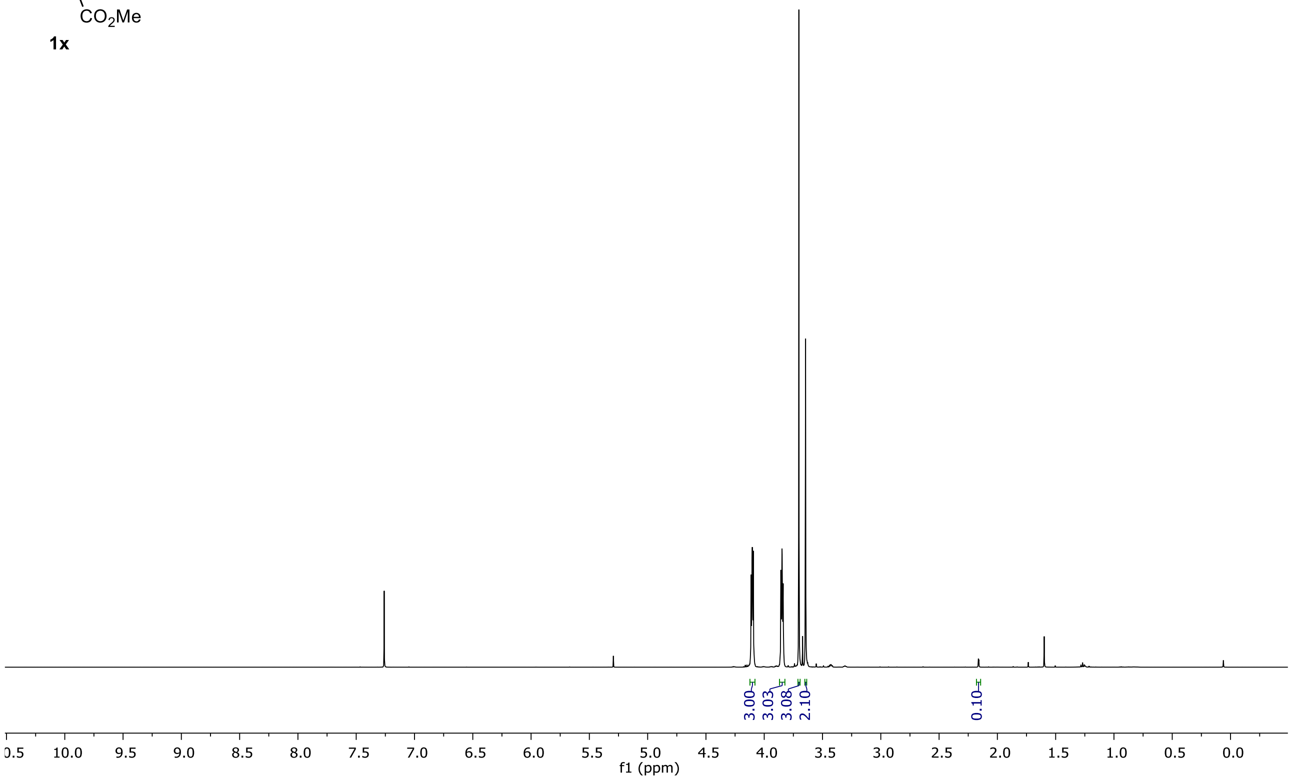
3.00
3.03
3.08
2.10
0.10
0.5
10.0
9.5
9.0
8.5
8.0
7.5
7.0
6.5
6.0
5.5
5.0
4.5
4.0
3.5
3.0
2.5
2.0
1.5
1.0
0.5
0.0
f1 (ppm)

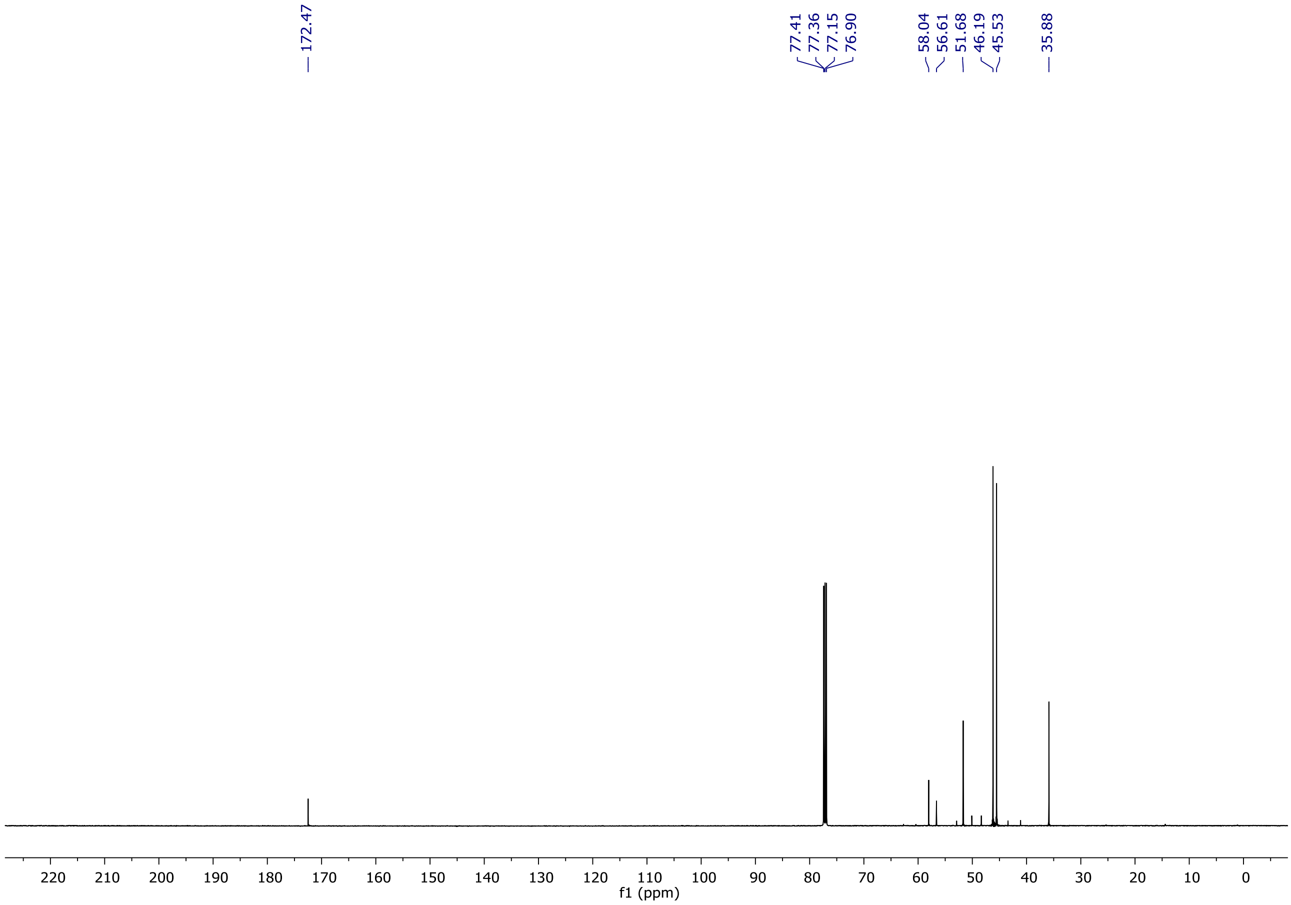
172.47
77.41
77.36
77.15
76.90
58.04
56.61
51.68
46.19
45.53
35.88
220
210
200
190
180
170
160
150
140
130
120
110
100
90
80
70
60
50
40
30
20
10
0
f1 (ppm)

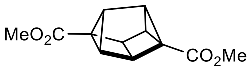
MeO2C
CO2Me

2a

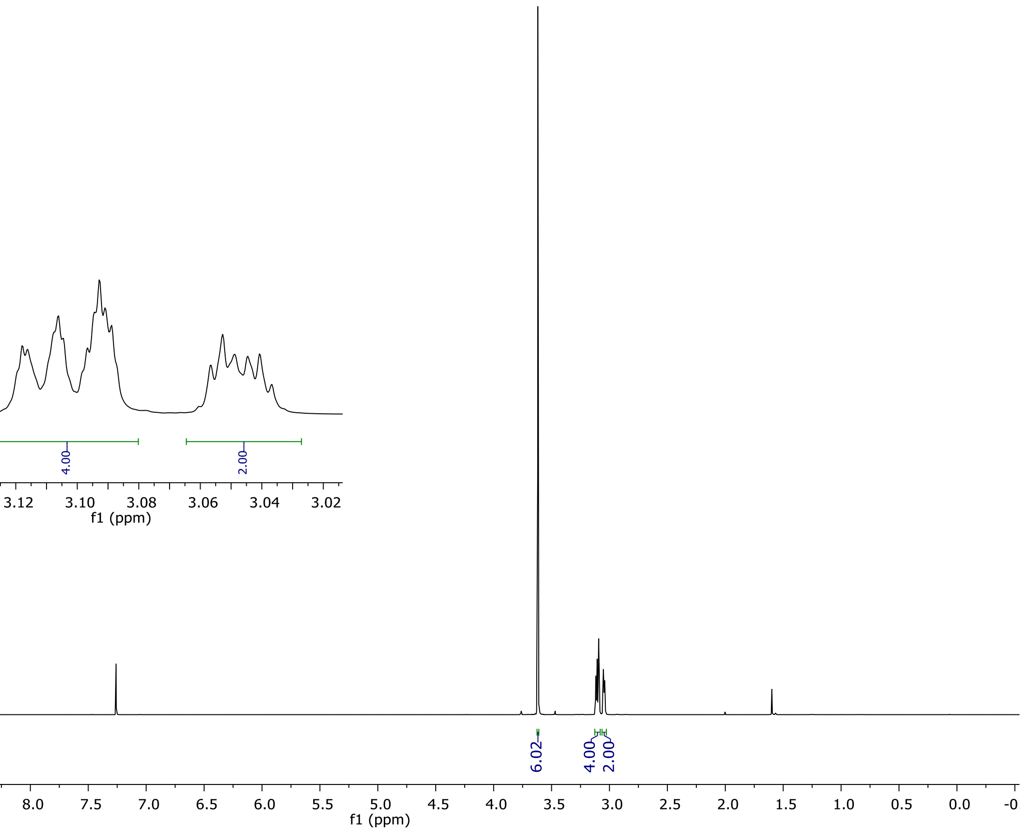
4.00
2.00
3.14 3.12 3.10 3.08 3.06 3.04 3.02
f1 (ppm)
6.02
4.00
2.00
.0.5 10.0 9.5 9.0 8.5 8.0 7.5 7.0 6.5 6.0 5.5 5.0 4.5 4.0 3.5 3.0 2.5 2.0 1.5 1.0 0.5 0.0 -0
f1 (ppm)

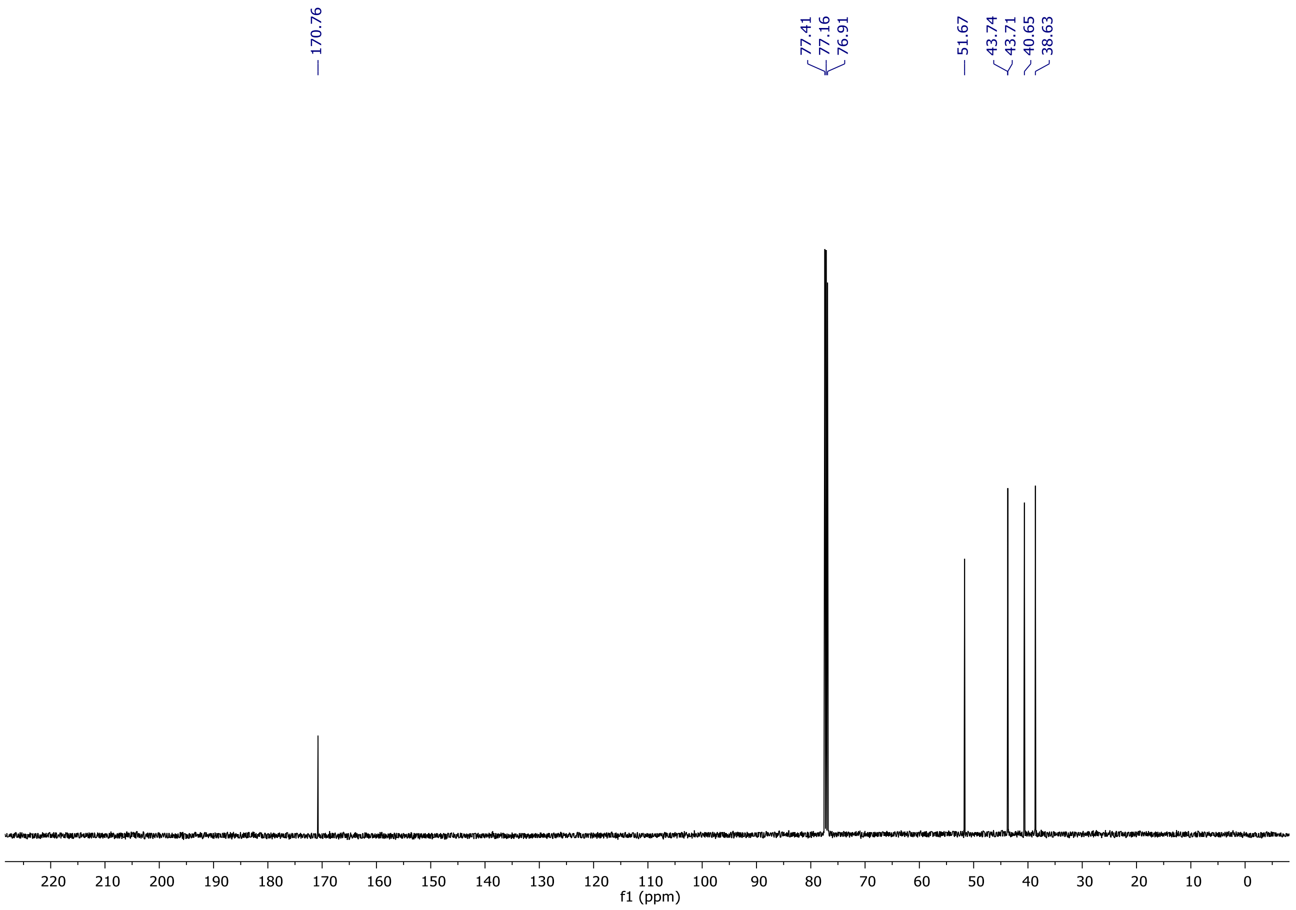
170.76
77.41
77.16
76.91
51.67
43.74
43.71
40.65
38.63
220 210 200 190 180 170 160 150 140 130 120 110 100 90 80 70 60 50 40 30 20 10 0
f1 (ppm)

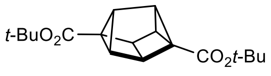
t-BuO2C
CO2t-Bu

2b

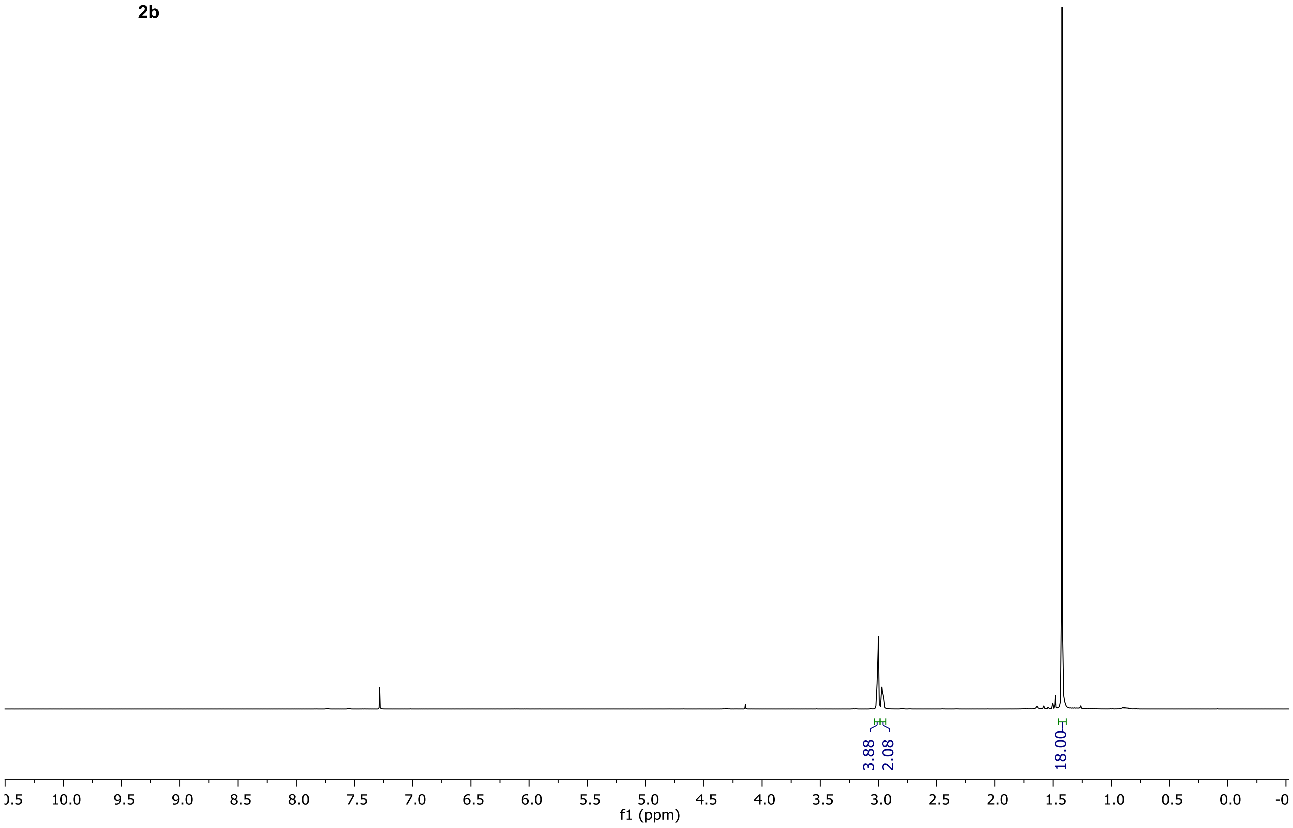
3.88
2.08
18.00
f1 (ppm)

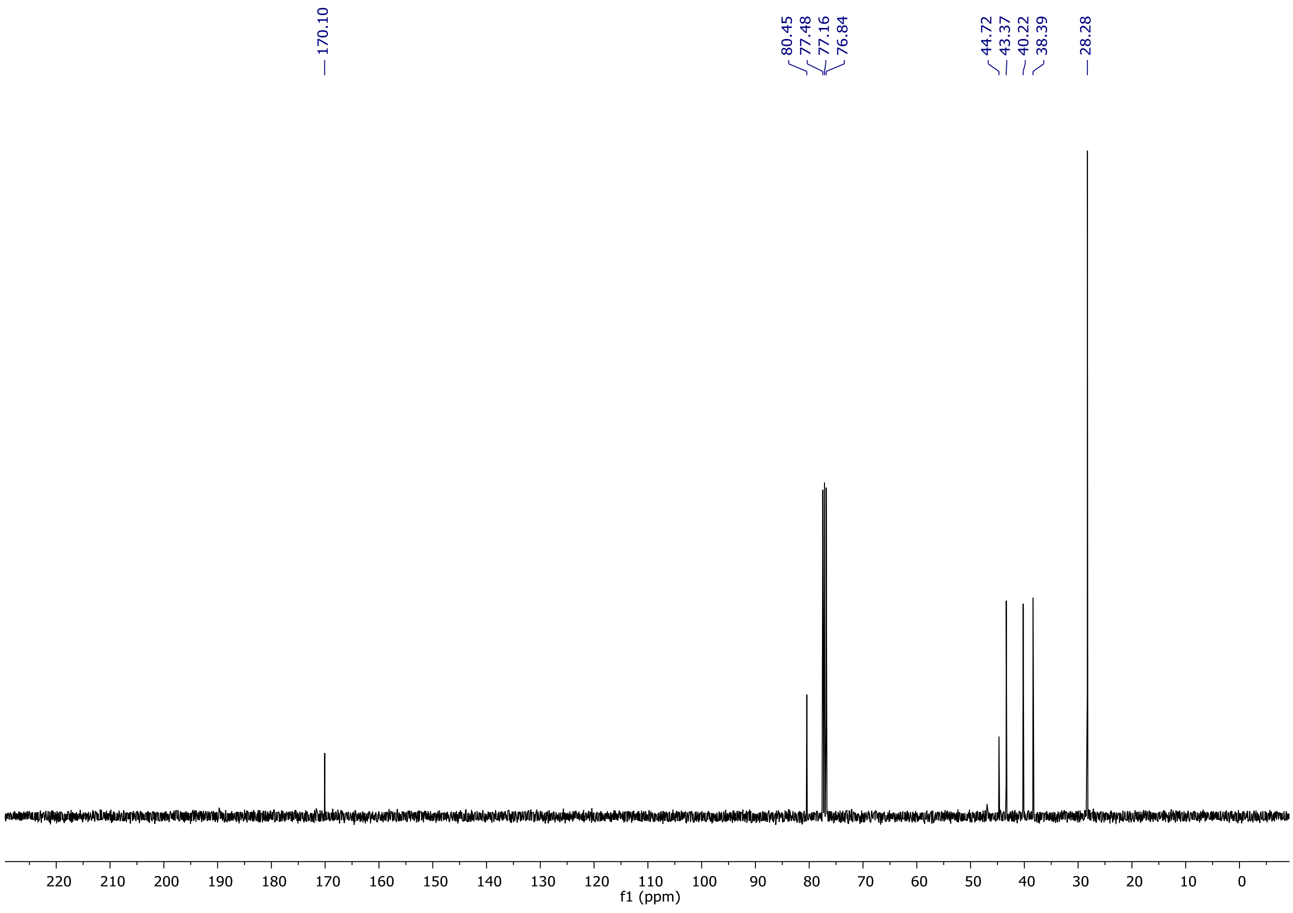
170.10
80.45
77.48
77.16
76.84
44.72
43.37
40.22
38.39
28.28
f1 (ppm)

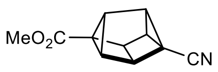
MeO2C
CN

2c

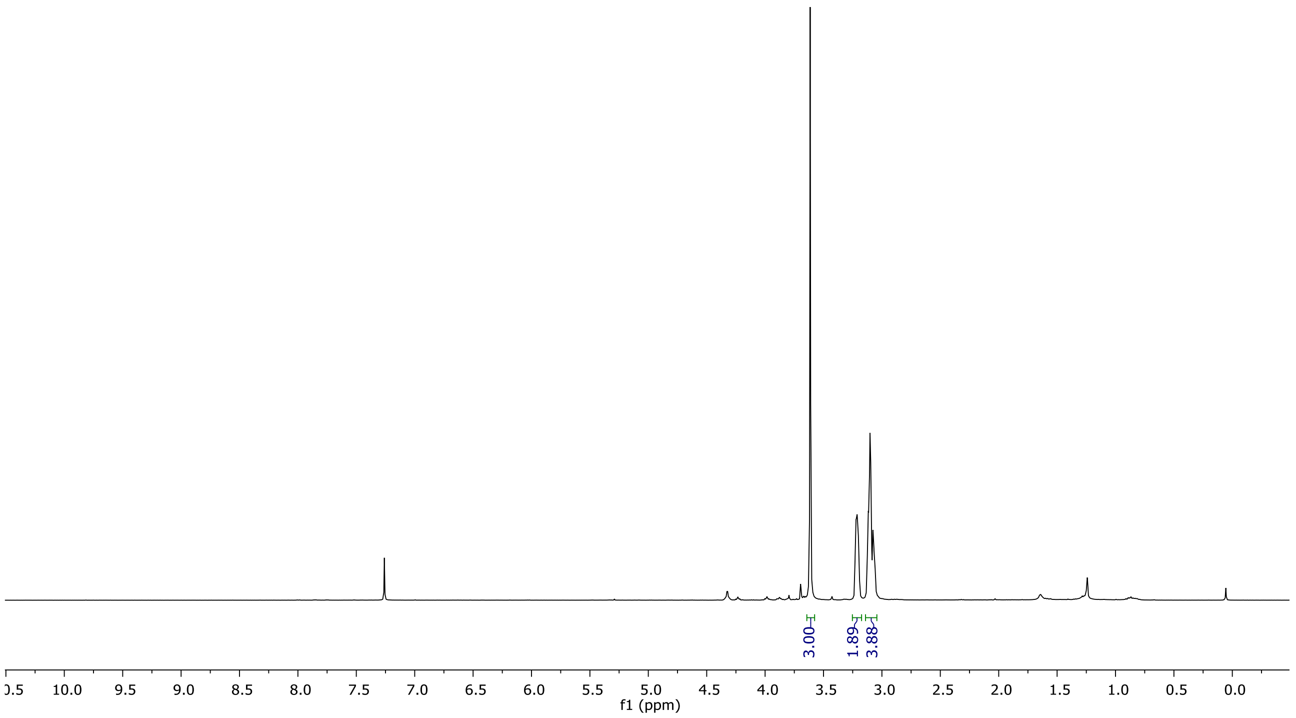
3.00
1.89
3.88
f1 (ppm)

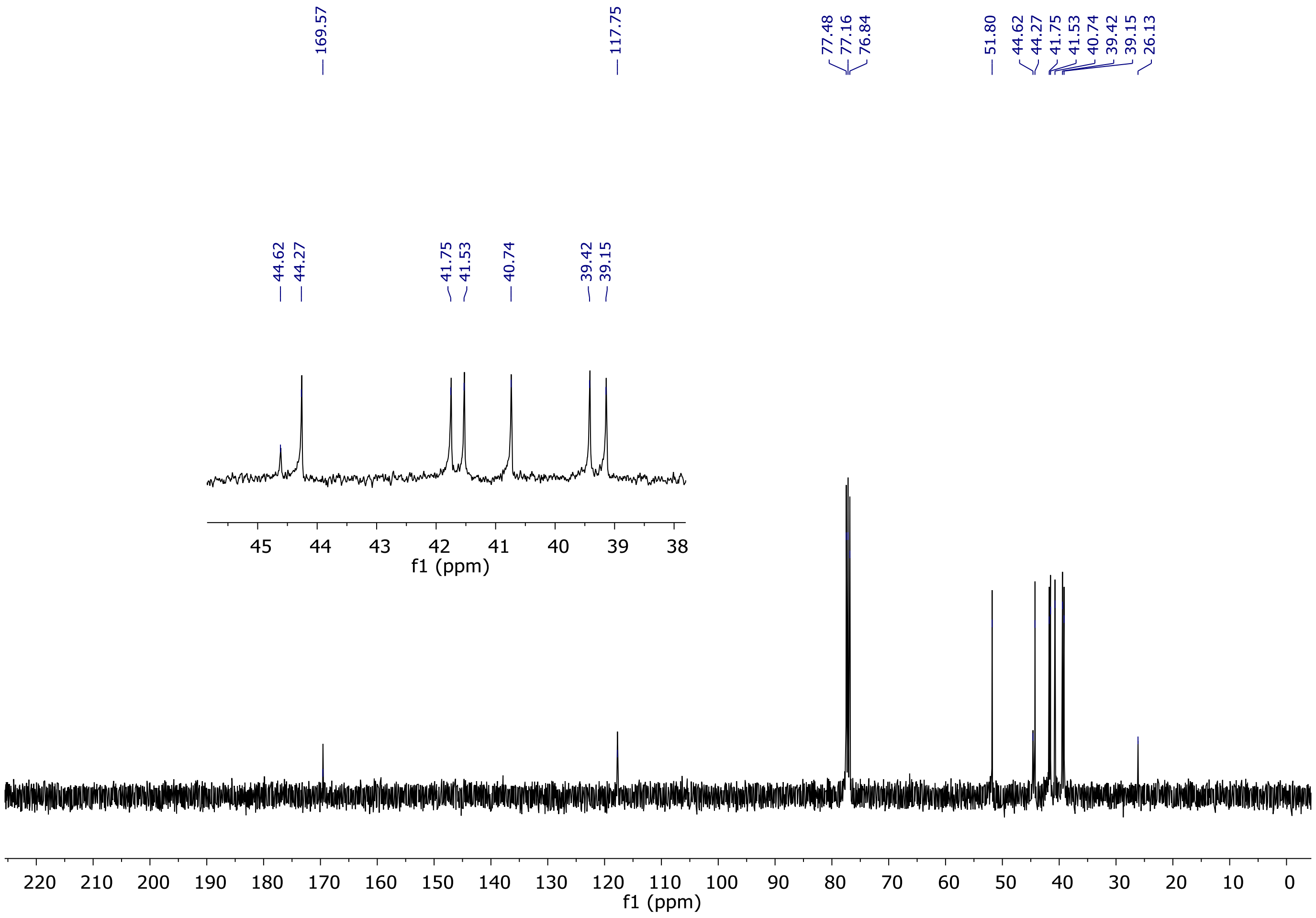
169.57
117.75
77.48
77.16
76.84
51.80
44.62
44.27
41.75
41.53
40.74
39.42
39.15
26.13
f1 (ppm)

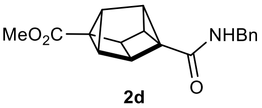
MeO2C
NHBn
O

2d

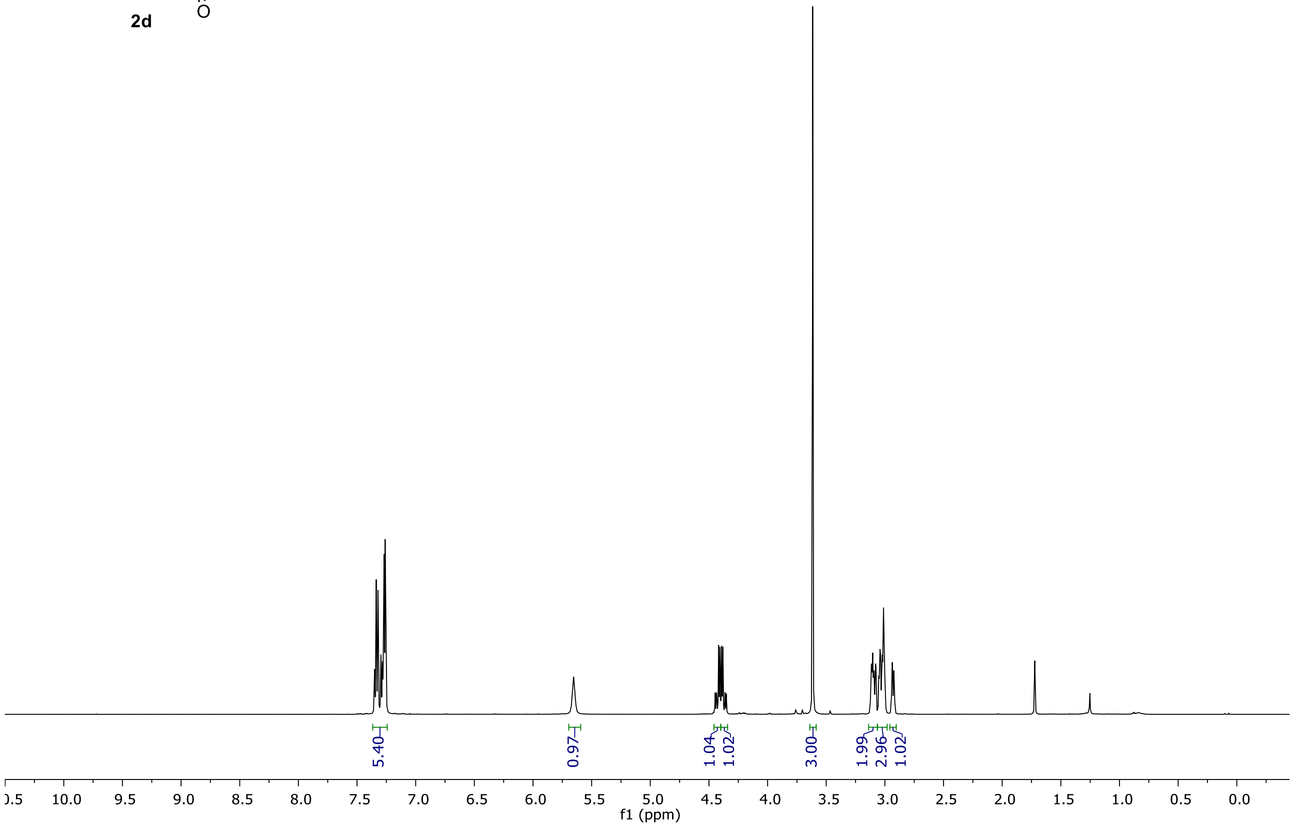
5.40
0.97
1.04
1.02
3.00
1.99
2.96
1.02
f1 (ppm)

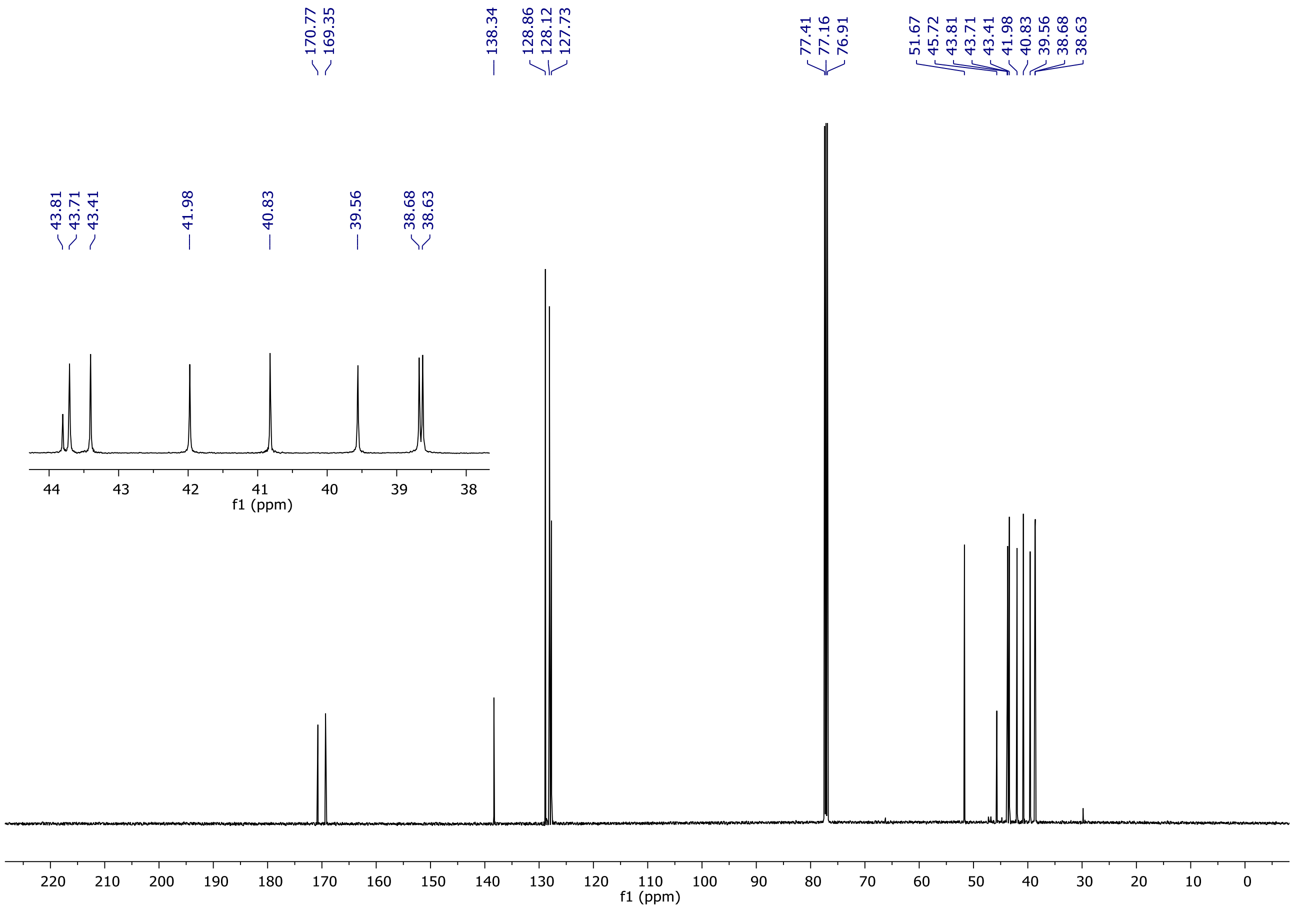
170.77
169.35
138.34
128.86
128.12
127.73
77.41
77.16
76.91
51.67
45.72
43.81
43.71
43.41
41.98
40.83
39.56
38.68
38.63
f1 (ppm)

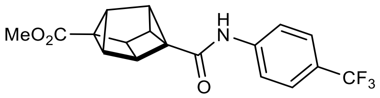
MeO2C
H
N
O
CF3

2e

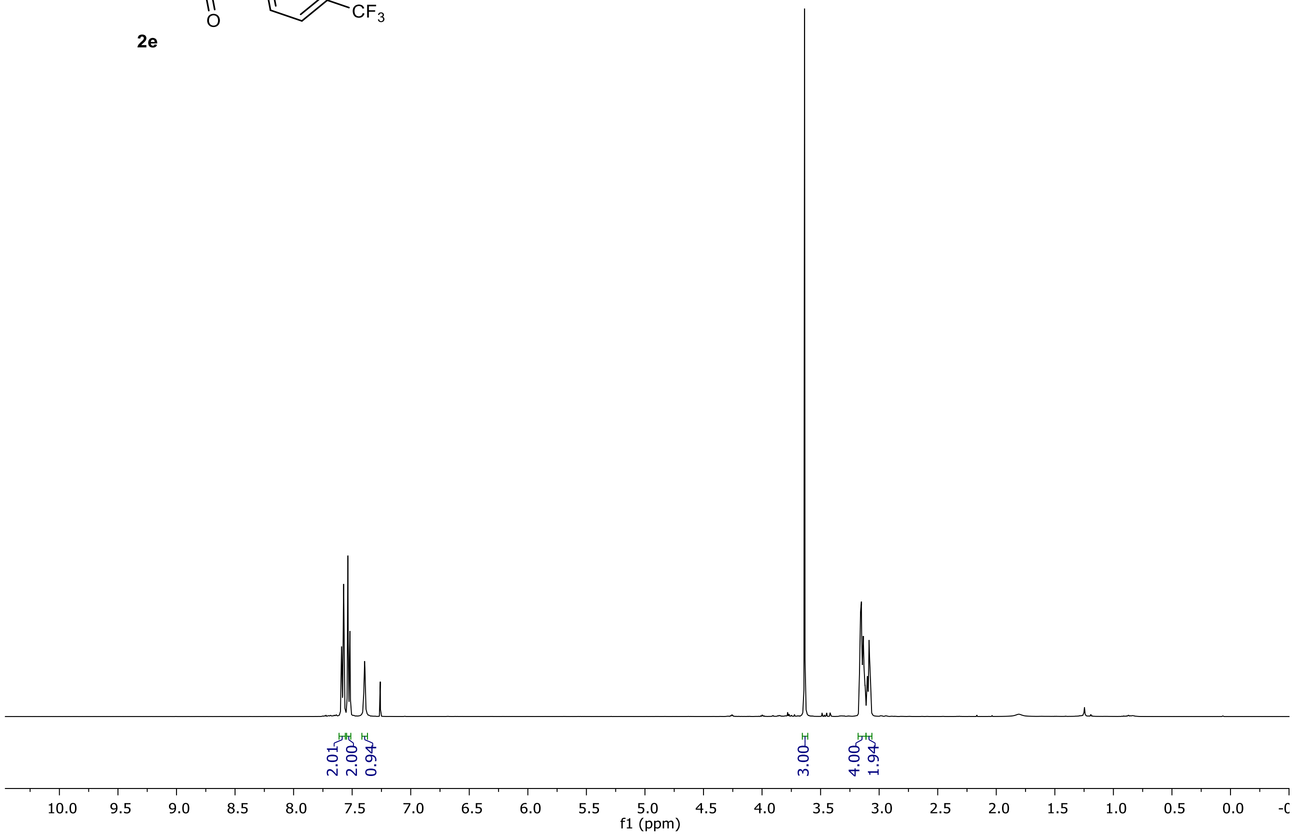
2.01
2.00
0.94
3.00
4.00
1.94
f1 (ppm)

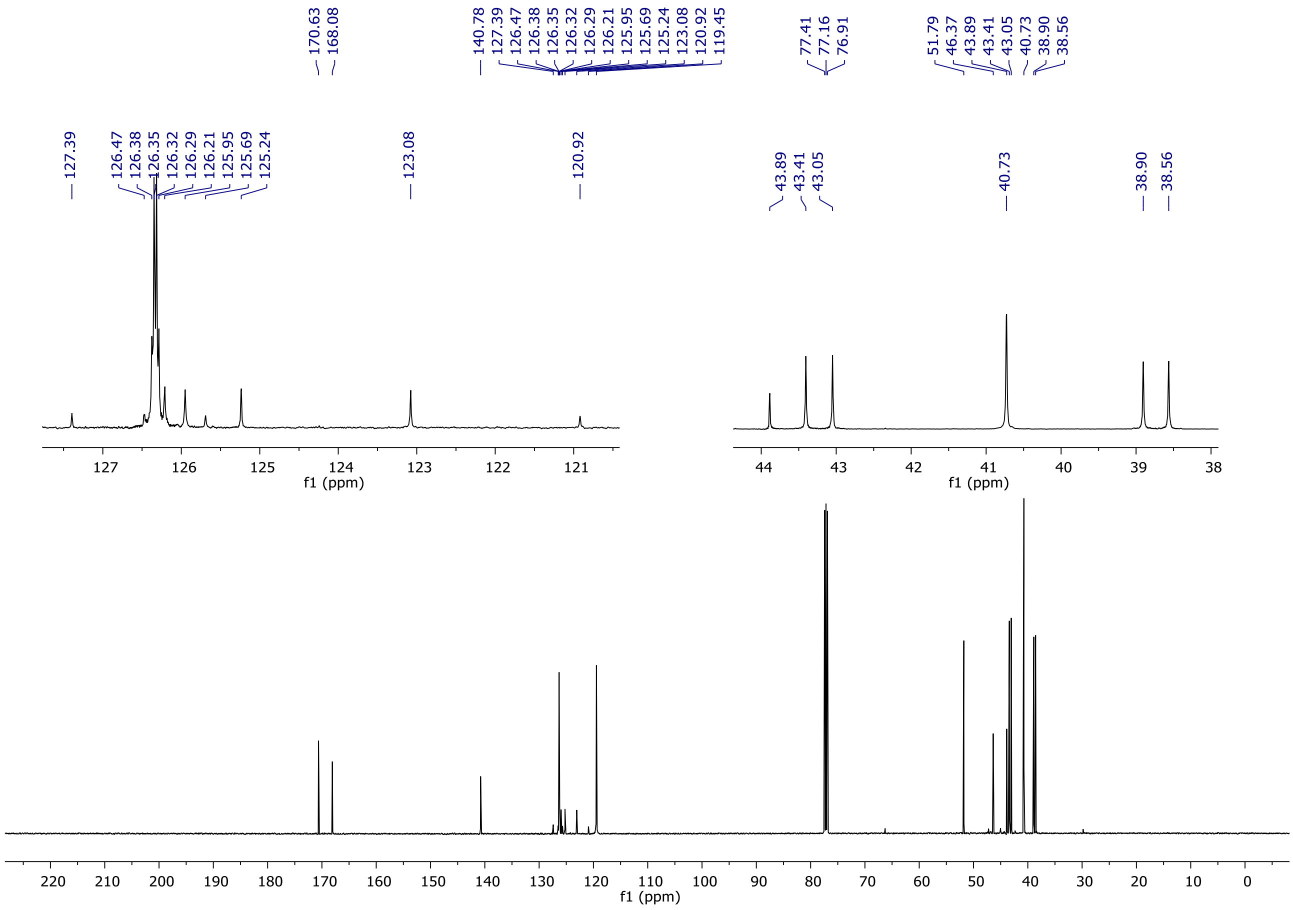
170.63
168.08
140.78
127.39
126.47
126.38
126.35
126.32
126.29
126.21
125.95
125.69
125.24
123.08
120.92
119.45
77.41
77.16
76.91
51.79
46.37
43.89
43.41
43.05
40.73
38.90
38.56
f1 (ppm)

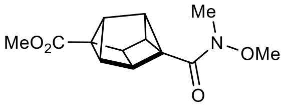
MeO2C
Me
N
OMe
O

2f

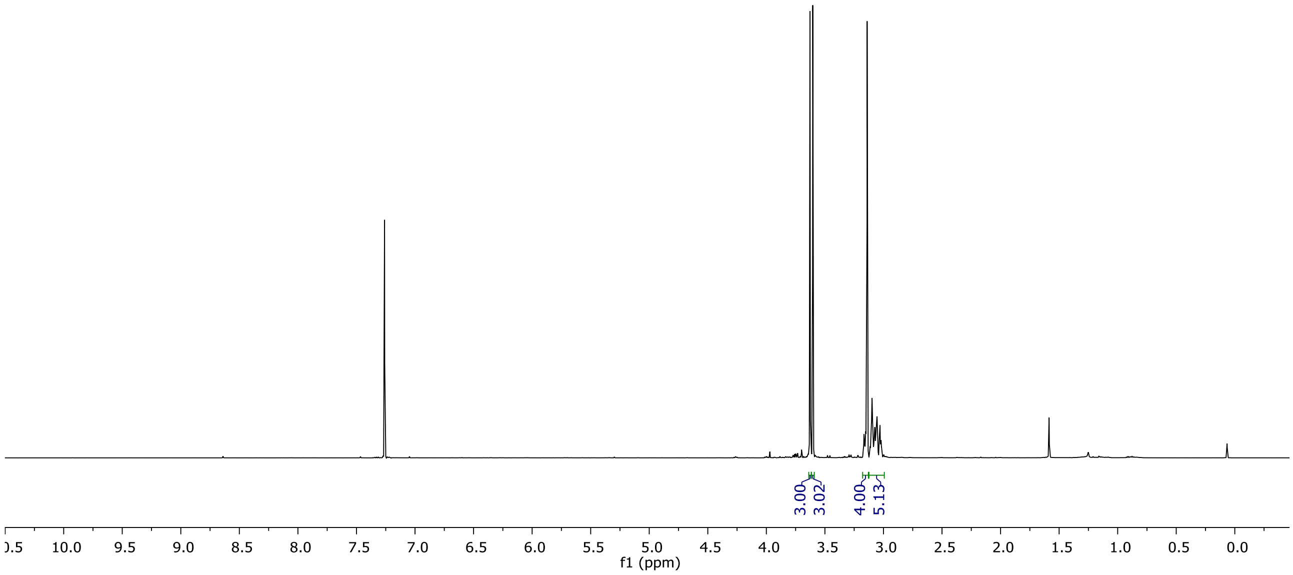
3.00
3.02
4.00
5.13
f1 (ppm)

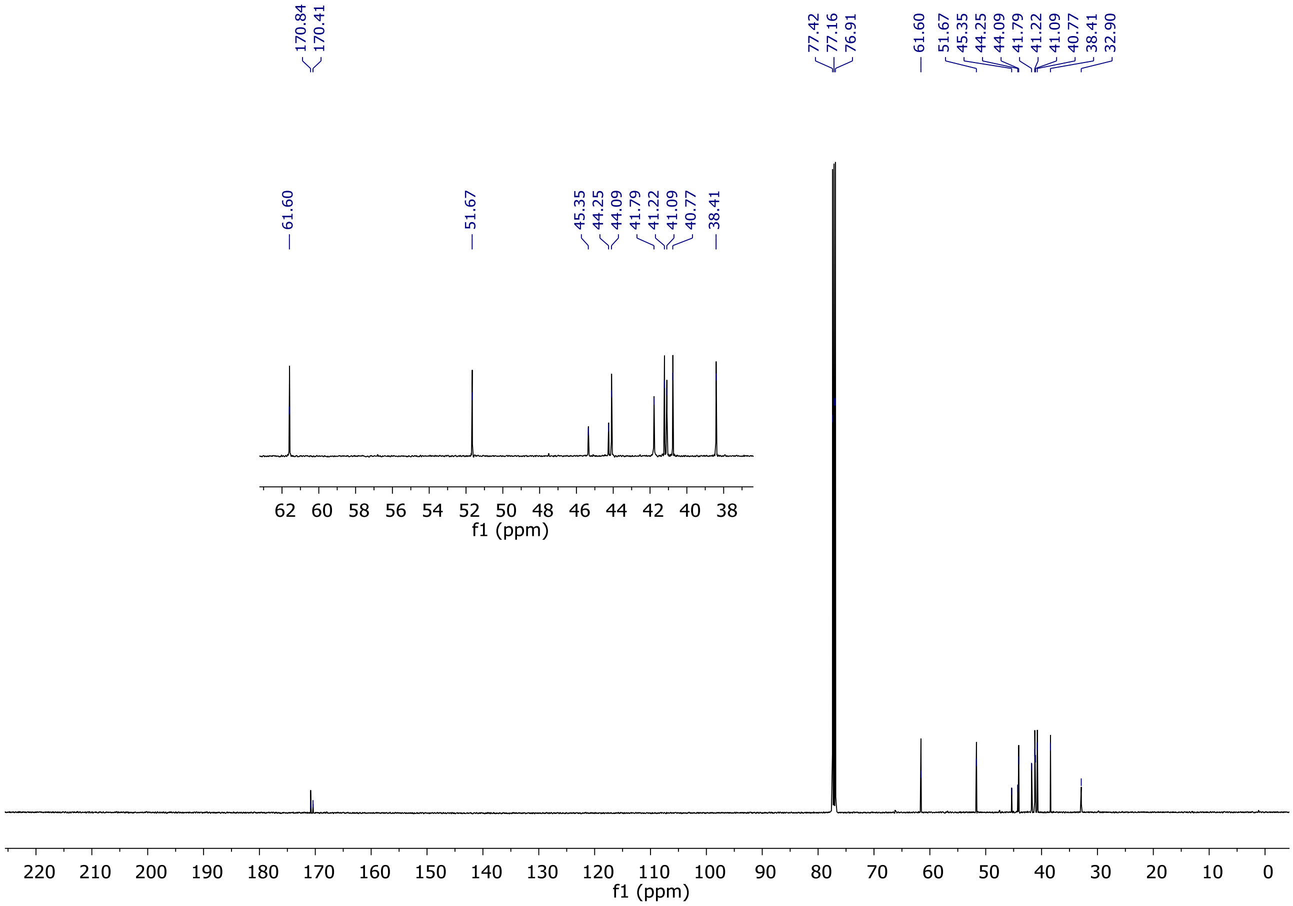
170.84
170.41
77.42
77.16
76.91
61.60
51.67
45.35
44.25
44.09
41.79
41.22
41.09
40.77
38.41
32.90
f1 (ppm)

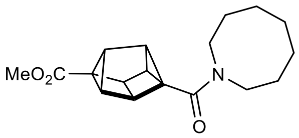
MeO2C
N
O

2g

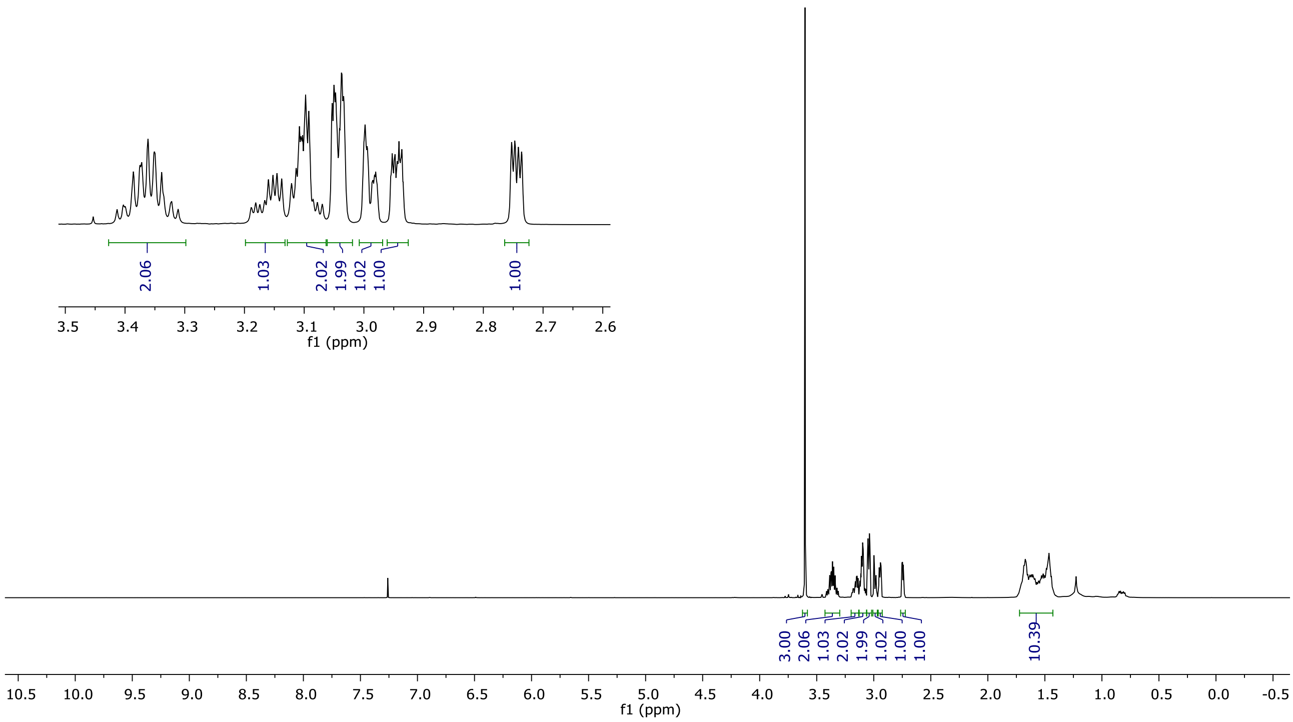
2.06
1.03
2.02
1.99
1.02
1.00
1.00
3.5 3.4 3.3 3.2 3.1 3.0 2.9 2.8 2.7 2.6
f1 (ppm)
3.00
2.06
1.03
2.02
1.99
1.02
1.00
1.00
10.39
10.5 10.0 9.5 9.0 8.5 8.0 7.5 7.0 6.5 6.0 5.5 5.0 4.5 4.0 3.5 3.0 2.5 2.0 1.5 1.0 0.5 0.0 -0.5
f1 (ppm)

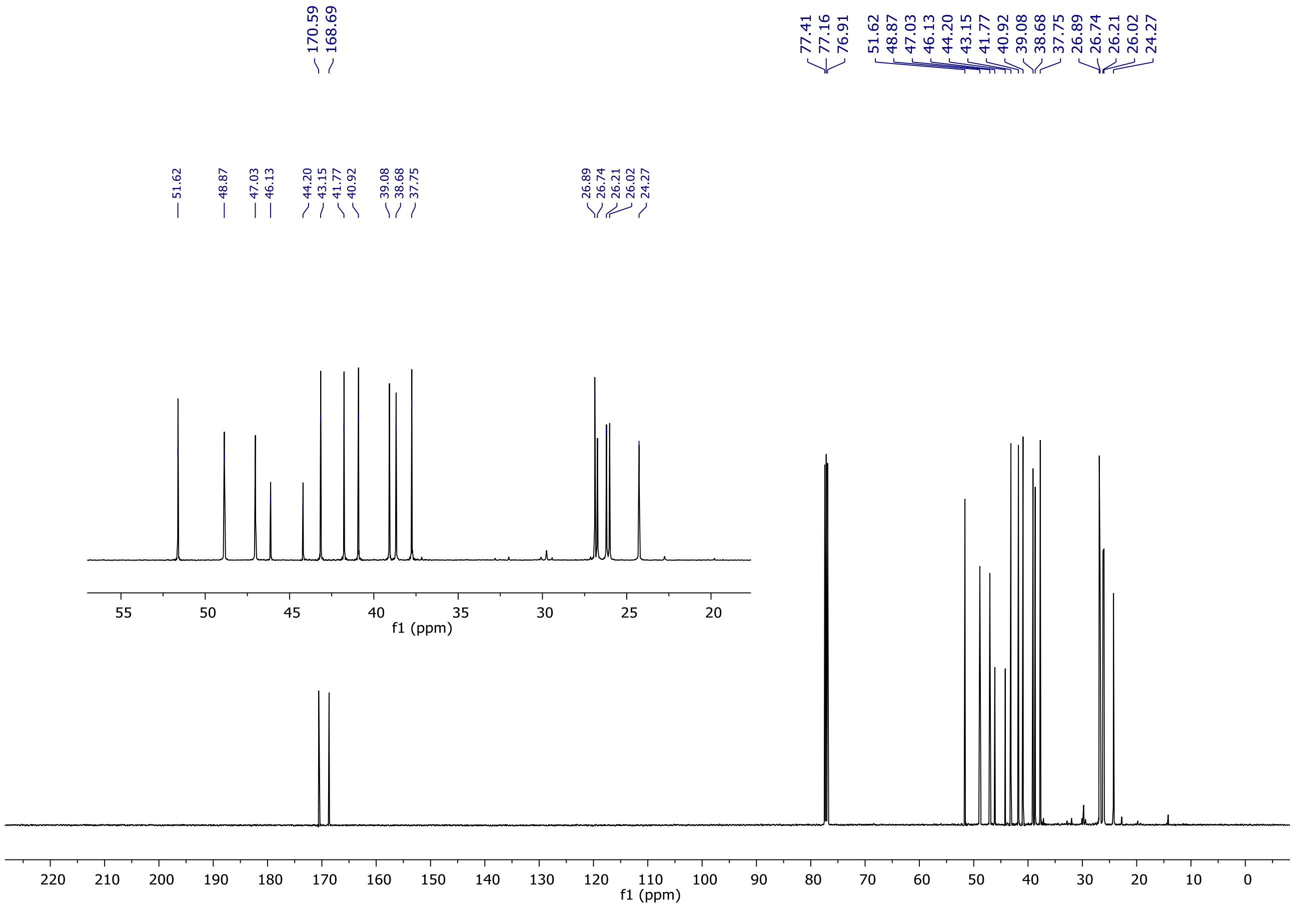
170.59
168.69
77.41
77.16
76.91
51.62
48.87
47.03
46.13
44.20
43.15
41.77
40.92
39.08
38.68
37.75
26.89
26.74
26.21
26.02
24.27
55 50 45 40 35 30 25 20
f1 (ppm)
220 210 200 190 180 170 160 150 140 130 120 110 100 90 80 70 60 50 40 30 20 10 0
f1 (ppm)

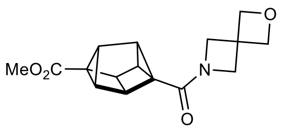
MeO2C
N
O
O

2h

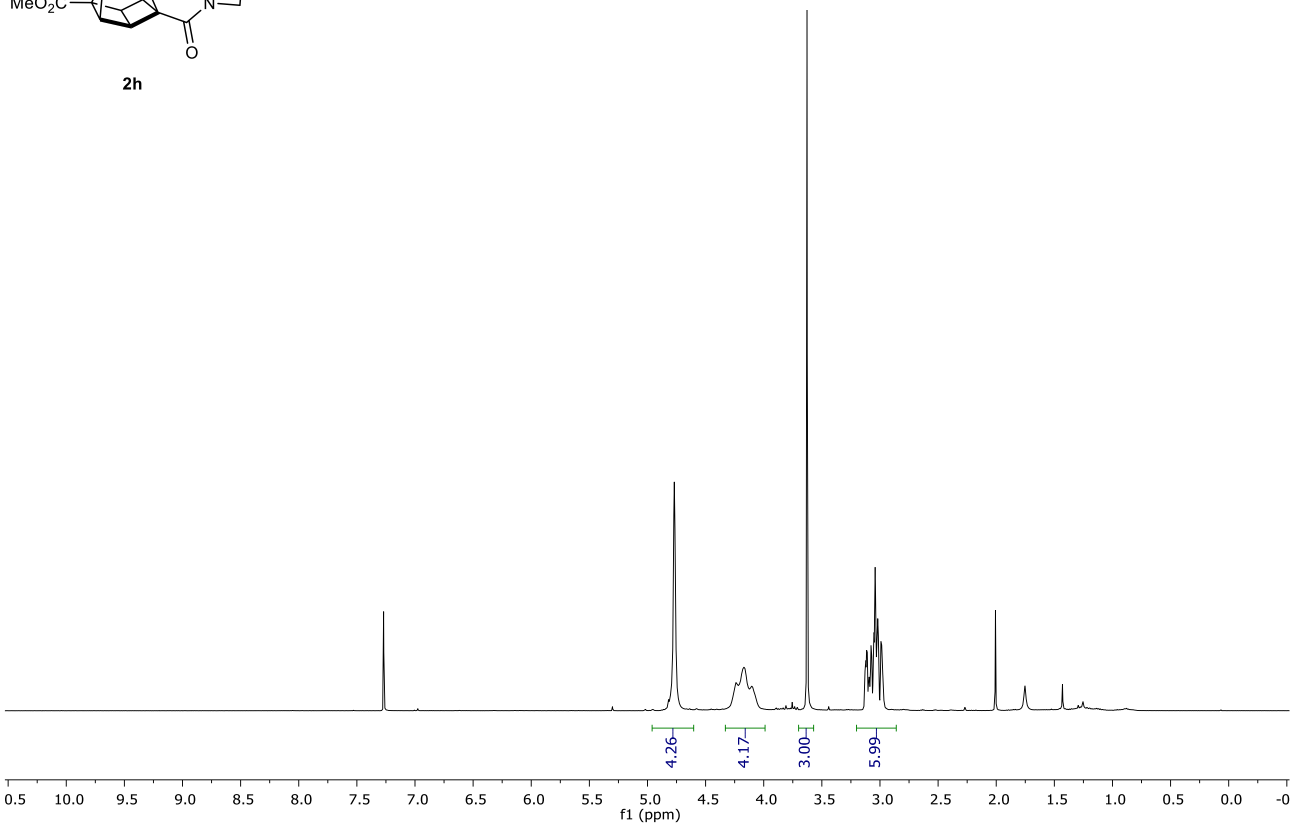
4.26
4.17
3.00
5.99
0.5 10.0 9.5 9.0 8.5 8.0 7.5 7.0 6.5 6.0 5.5 5.0 4.5 4.0 3.5 3.0 2.5 2.0 1.5 1.0 0.5 0.0 -0
f1 (ppm)

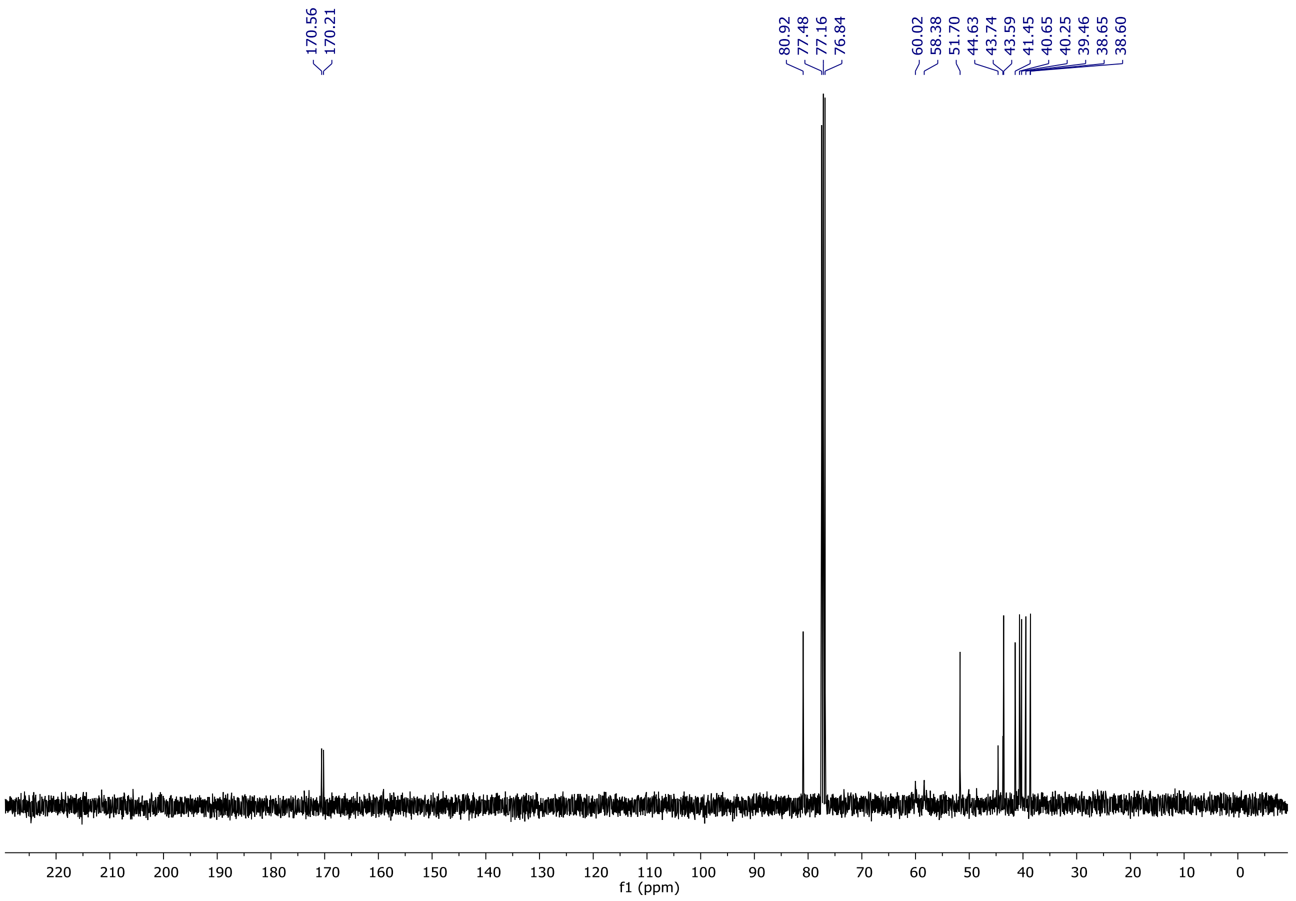
170.56
170.21
80.92
77.48
77.16
76.84
60.02
58.38
51.70
44.63
43.74
43.59
41.45
40.65
40.25
39.46
38.65
38.60
220 210 200 190 180 170 160 150 140 130 120 110 100 90 80 70 60 50 40 30 20 10 0
f1 (ppm)

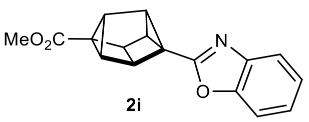
MeO2C
N
O

2i

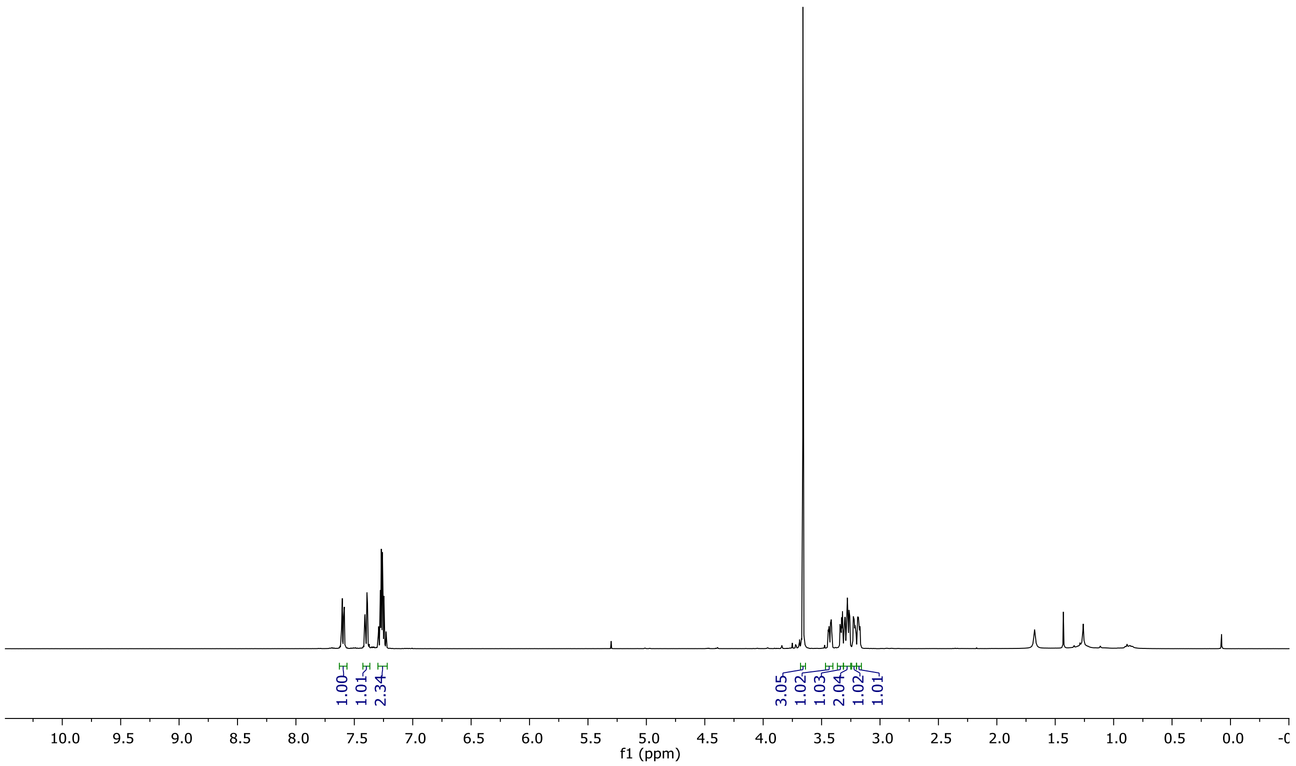
1.00
1.01
2.34
3.05
1.02
1.03
2.04
1.02
1.01
10.0 9.5 9.0 8.5 8.0 7.5 7.0 6.5 6.0 5.5 5.0 4.5 4.0 3.5 3.0 2.5 2.0 1.5 1.0 0.5 0.0
f1 (ppm)

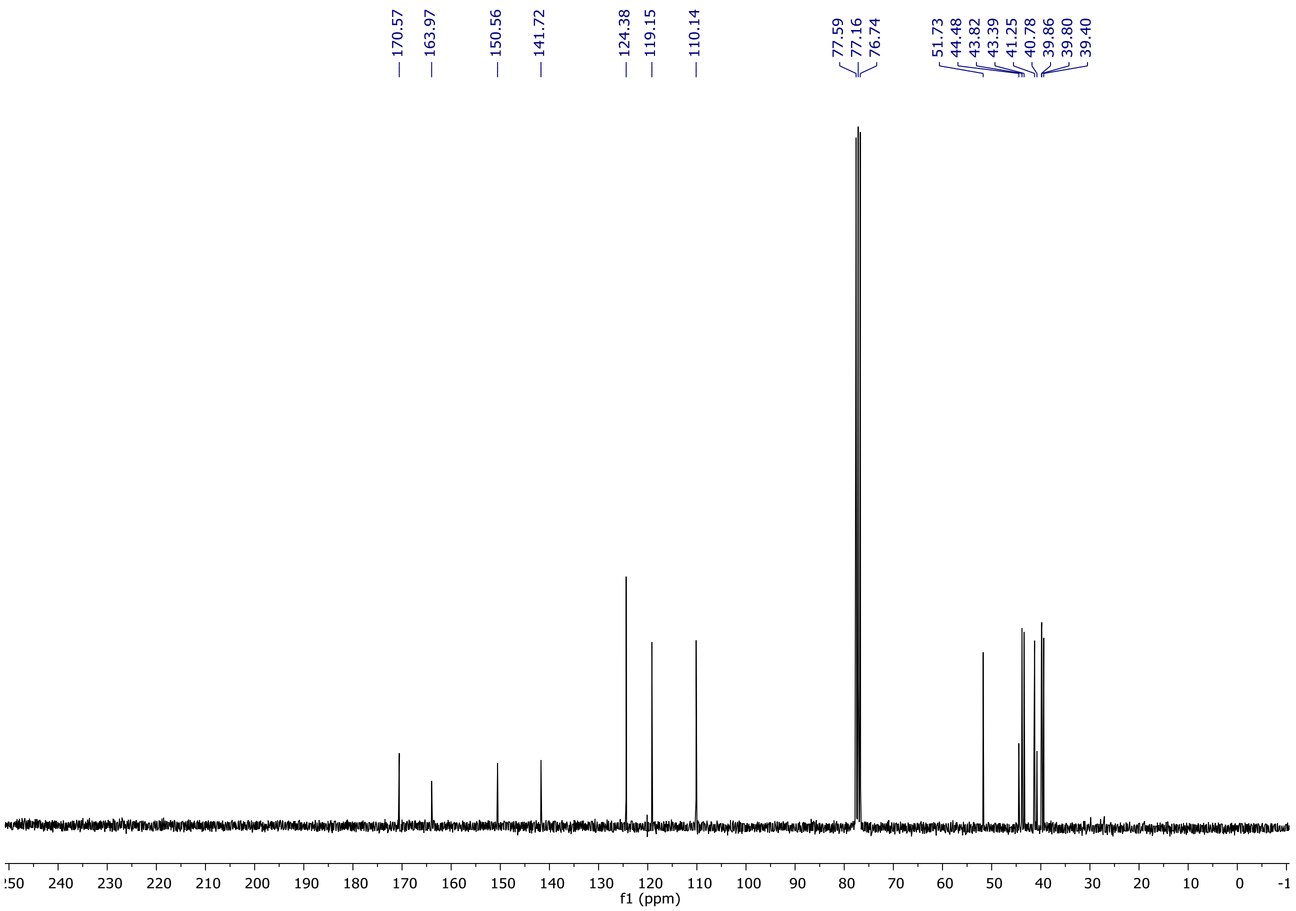
170.57
163.97
150.56
141.72
124.38
119.15
110.14
77.59
77.16
76.74
51.73
44.48
43.82
43.39
41.25
40.78
39.86
39.80
39.40
240 230 220 210 200 190 180 170 160 150 140 130 120 110 100 90 80 70 60 50 40 30 20 10 0
f1 (ppm)

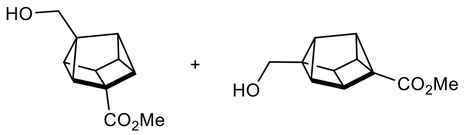

3b (tentative)

~24:1 ratio

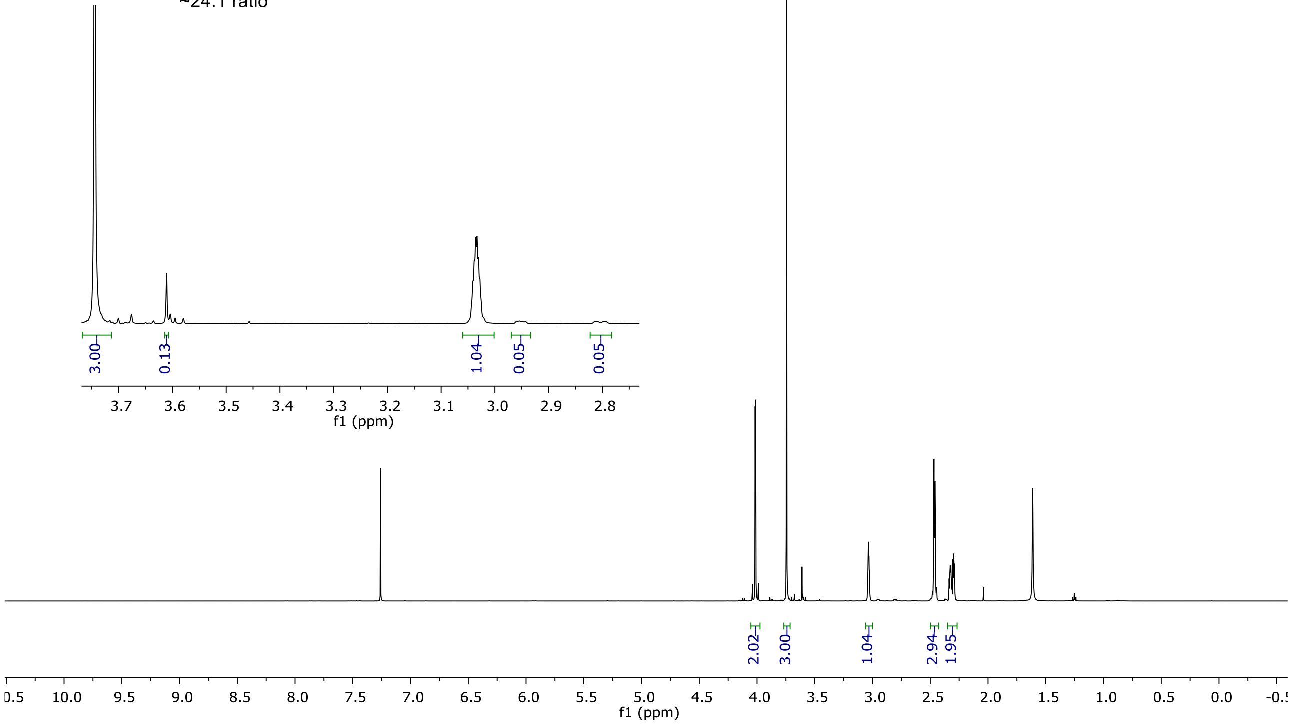

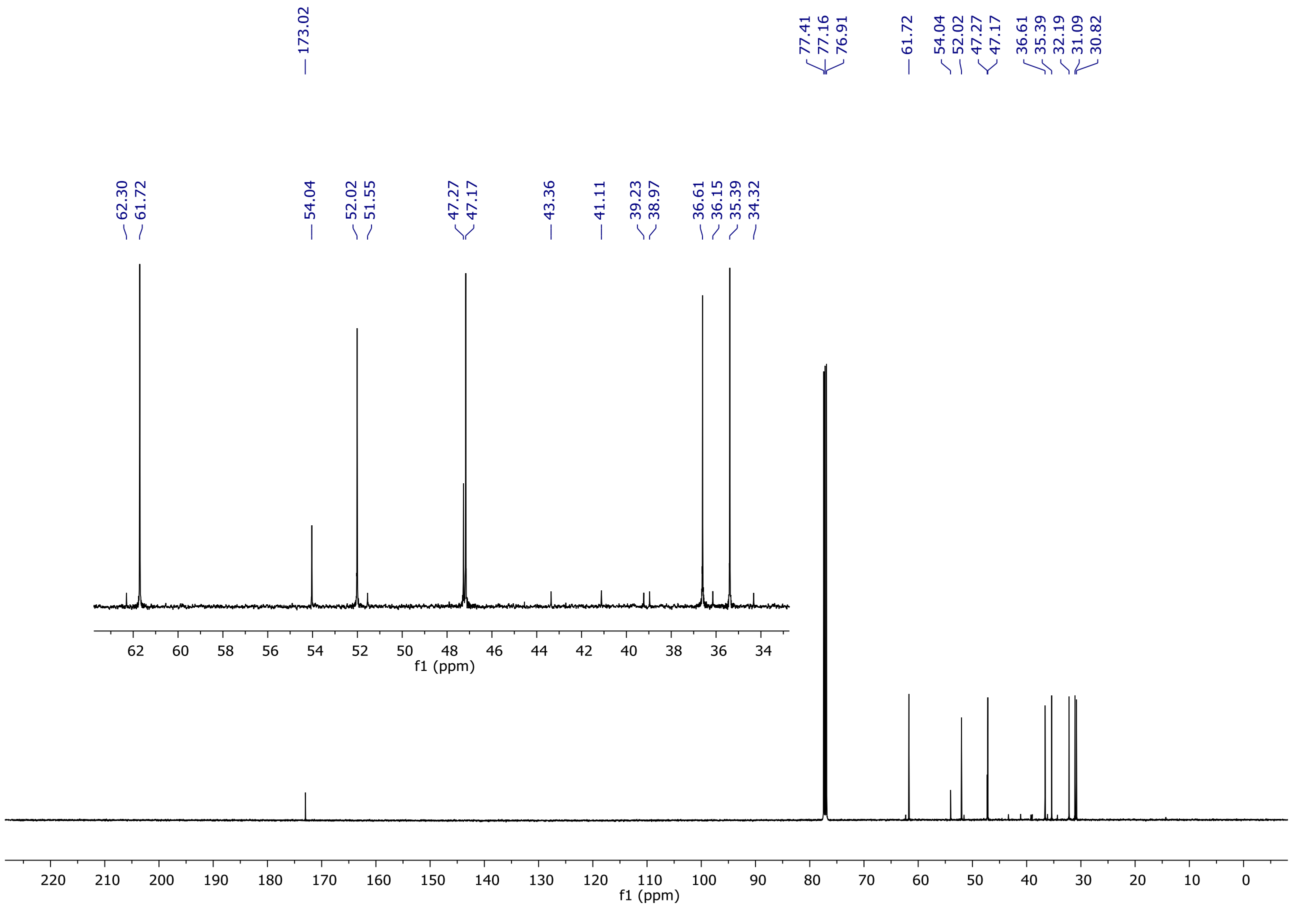

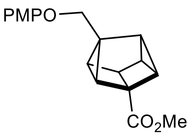
PMPO
CO_2Me

3c

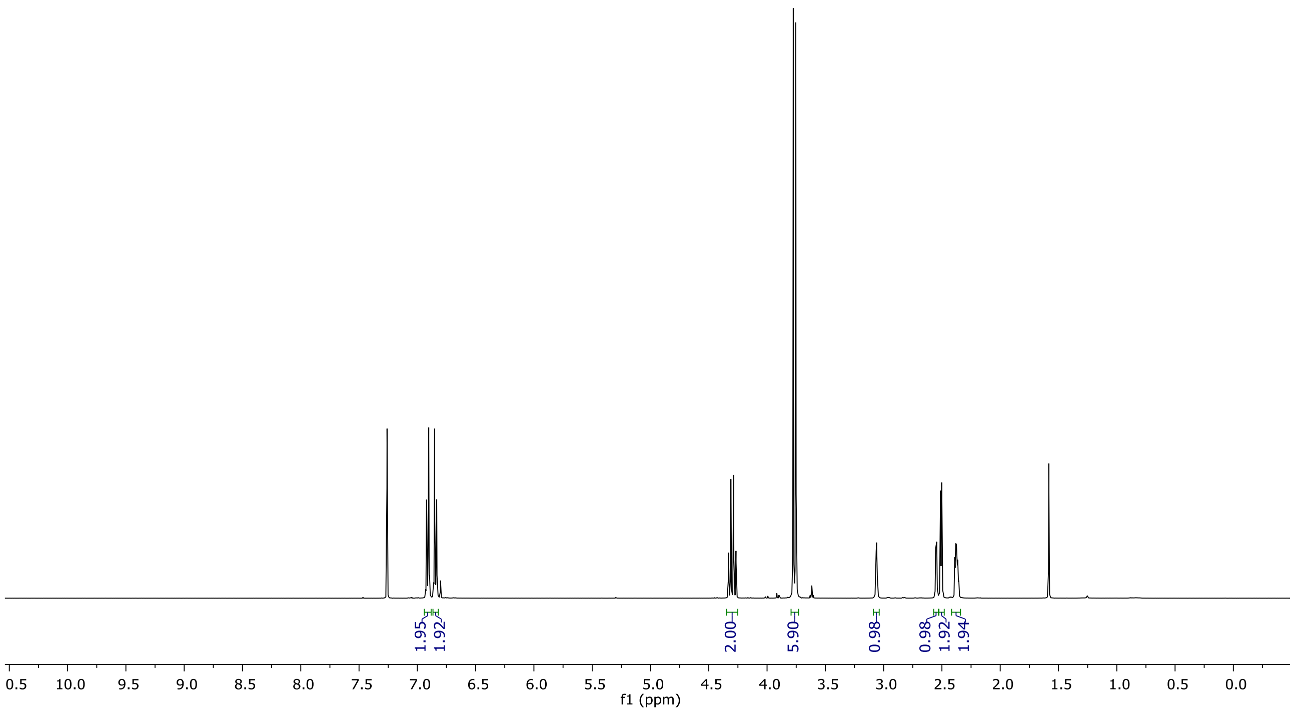
1.95
1.92
2.00
5.90
0.98
0.98
1.92
1.94
0.5 10.0 9.5 9.0 8.5 8.0 7.5 7.0 6.5 6.0 5.5 5.0 4.5 4.0 3.5 3.0 2.5 2.0 1.5 1.0 0.5 0.0
f1 (ppm)

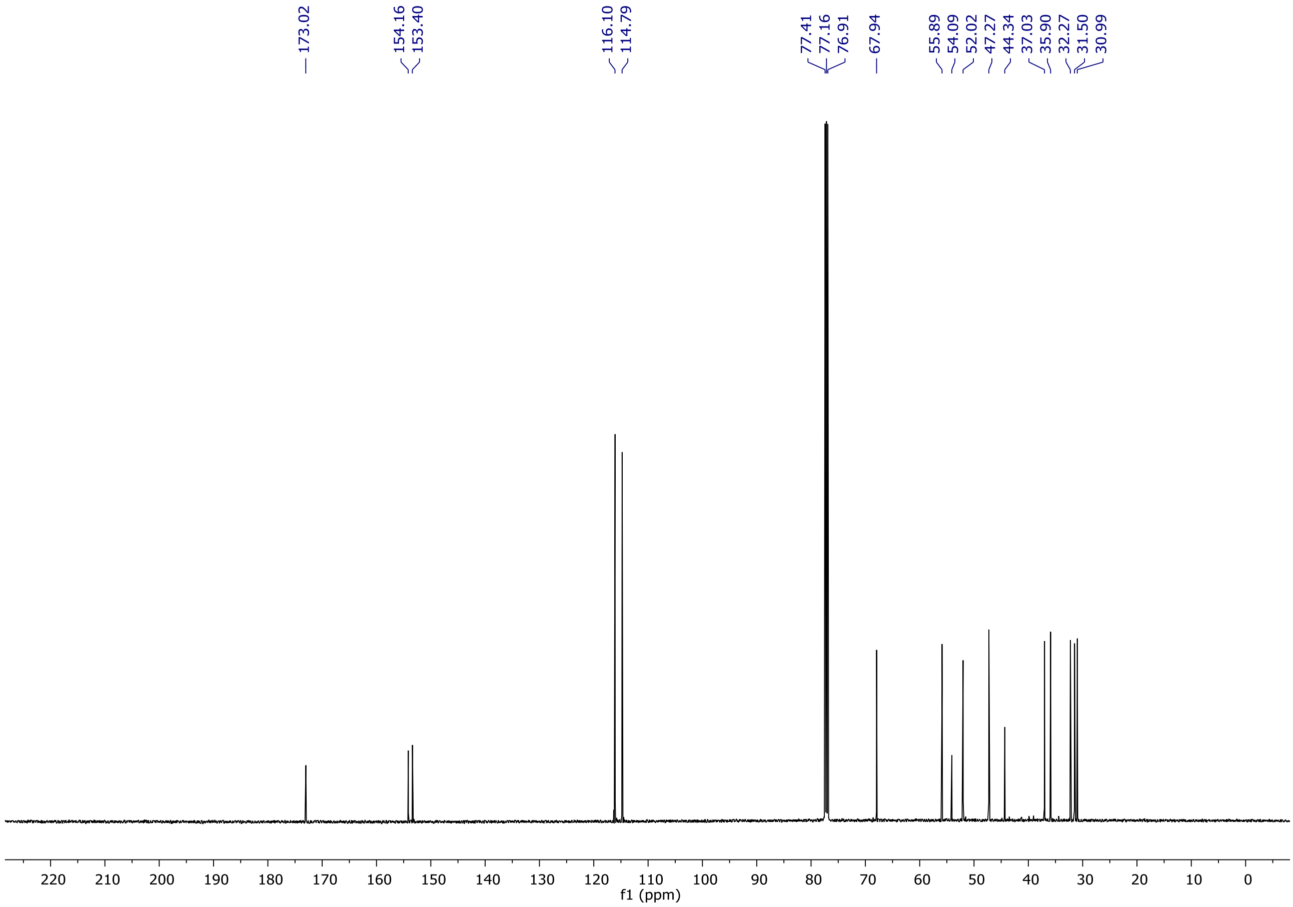
173.02
154.16
153.40
116.10
114.79
77.41
77.16
76.91
67.94
55.89
54.09
52.02
47.27
44.34
37.03
35.90
32.27
31.50
30.99
220 210 200 190 180 170 160 150 140 130 120 110 100 90 80 70 60 50 40 30 20 10 0
f1 (ppm)

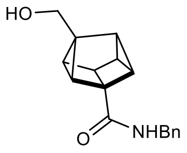
HO
O
NHBn

3d

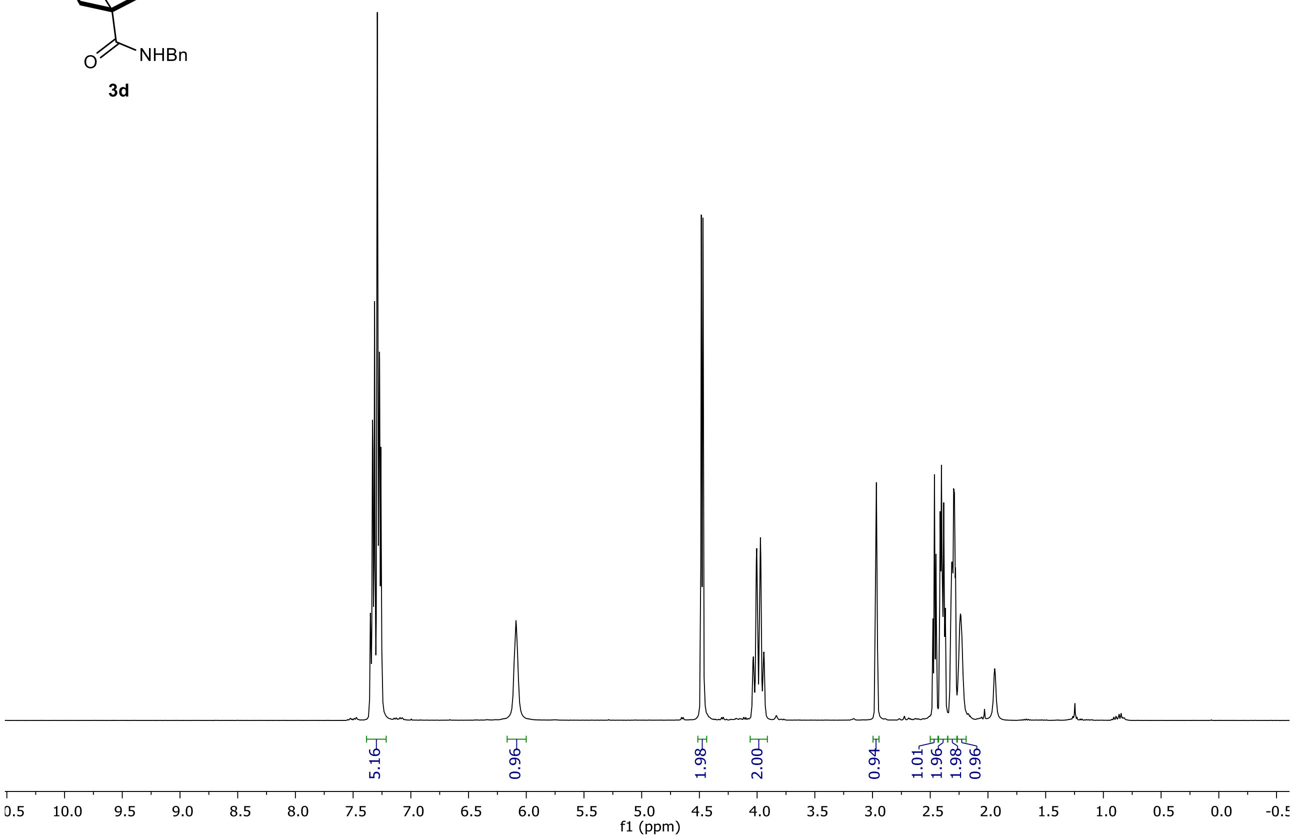
5.16
0.96
1.98
2.00
0.94
1.01
1.96
1.98
0.96
f1 (ppm)

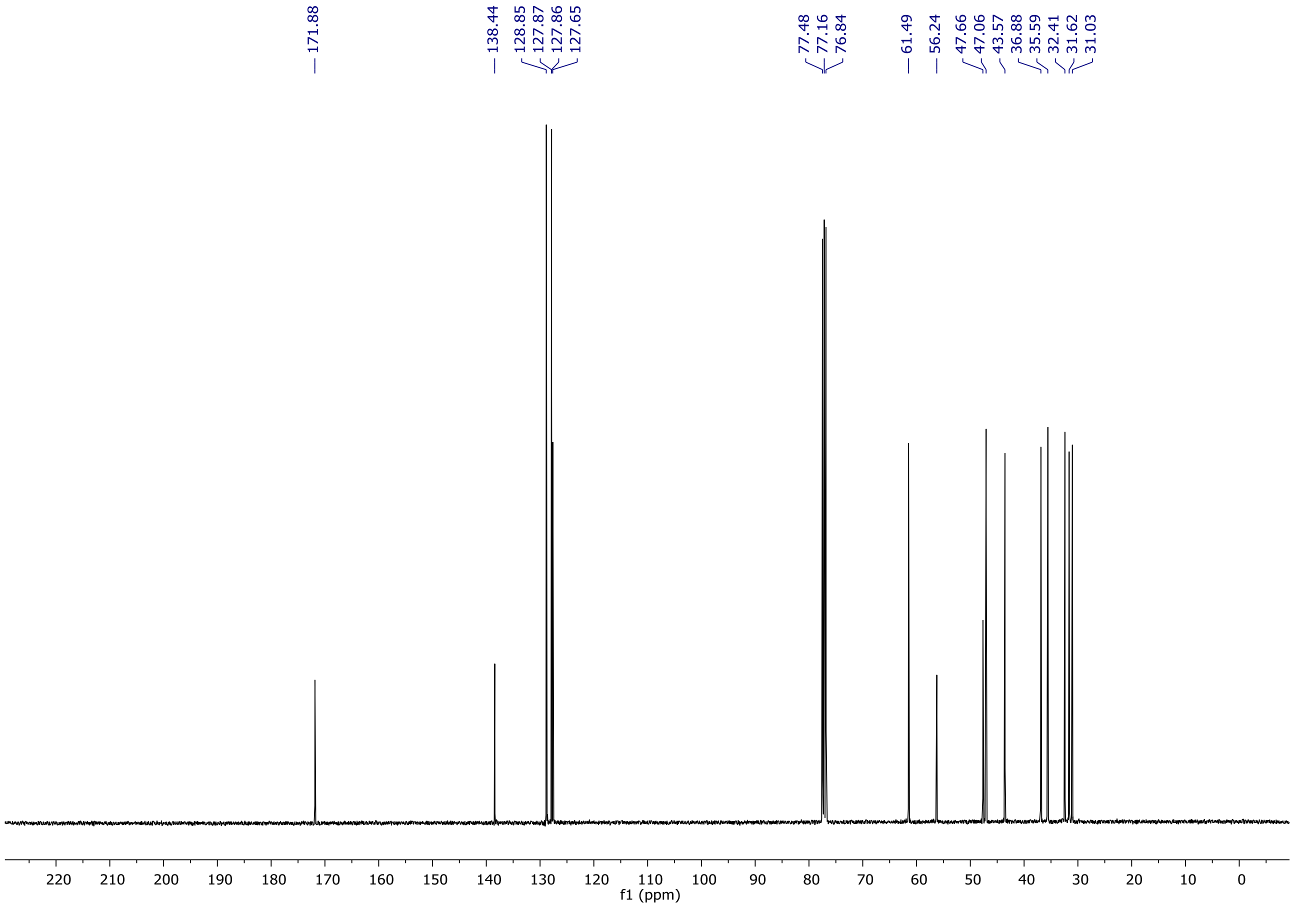
171.88
138.44
128.85
127.87
127.86
127.65
77.48
77.16
76.84
61.49
56.24
47.66
47.06
43.57
36.88
35.59
32.41
31.62
31.03
f1 (ppm)

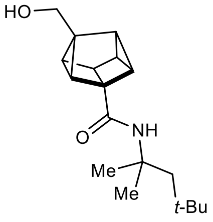
HO
O
NH
Me
Me
t-Bu

3e

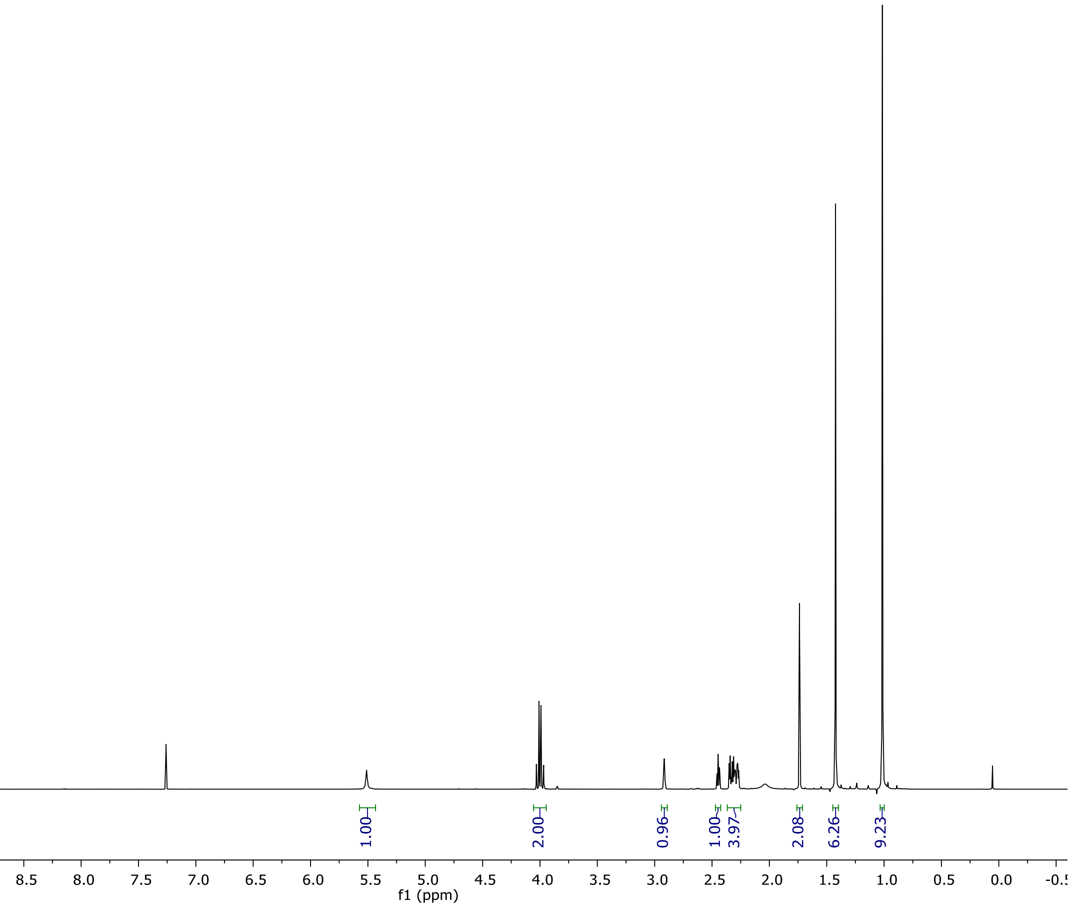
1.00
2.00
0.96
1.00
3.97
2.08
6.26
9.23
10.5 10.0 9.5 9.0 8.5 8.0 7.5 7.0 6.5 6.0 5.5 5.0 4.5 4.0 3.5 3.0 2.5 2.0 1.5 1.0 0.5 0.0
f1 (ppm)

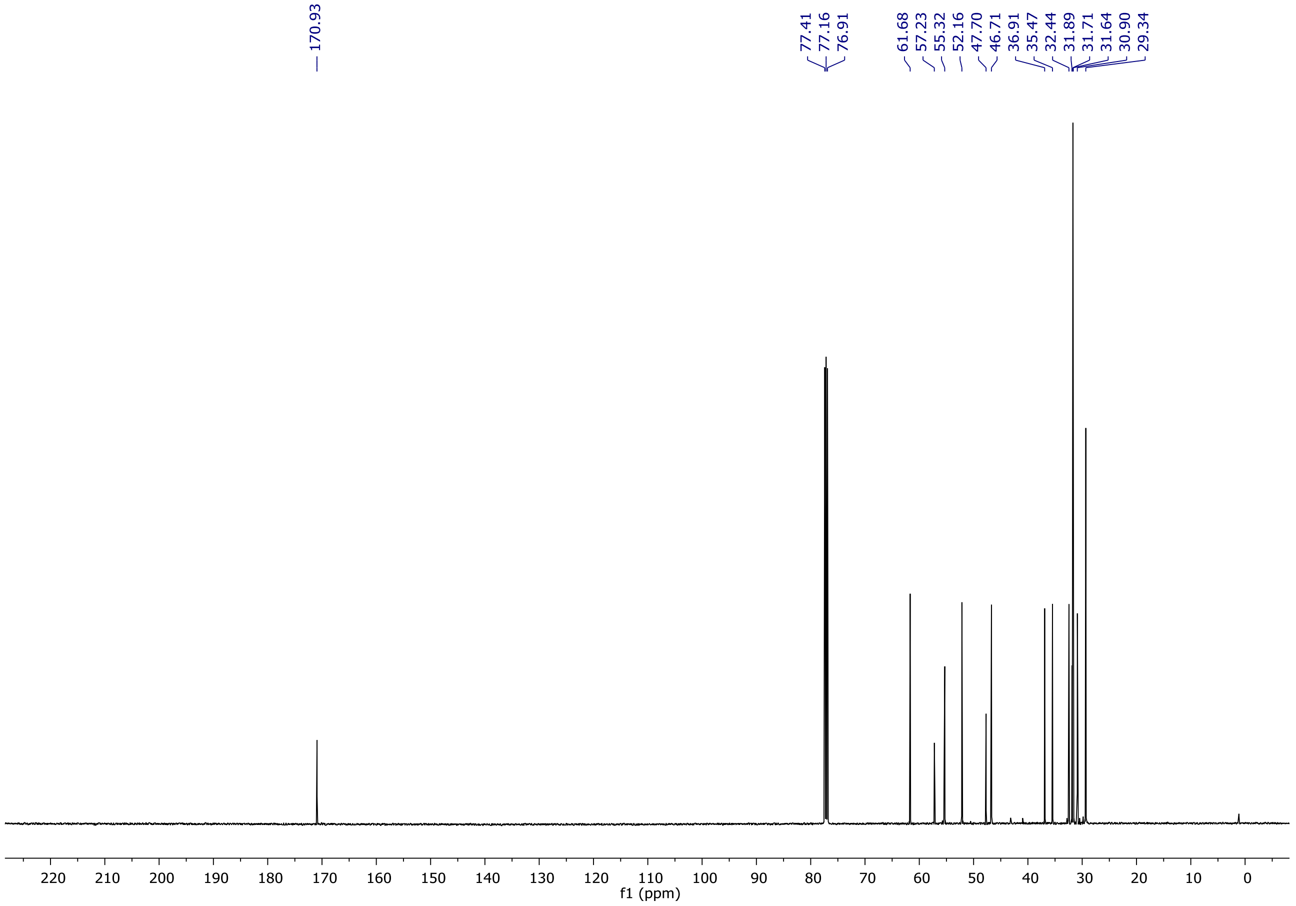
170.93
77.41
77.16
76.91
61.68
57.23
55.32
52.16
47.70
46.71
36.91
35.47
32.44
31.89
31.71
31.64
30.90
29.34
220 210 200 190 180 170 160 150 140 130 120 110 100 90 80 70 60 50 40 30 20 10 0
f1 (ppm)

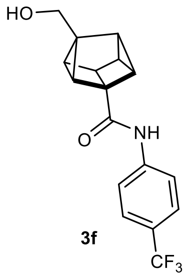
HO
O
NH
CF3

3f

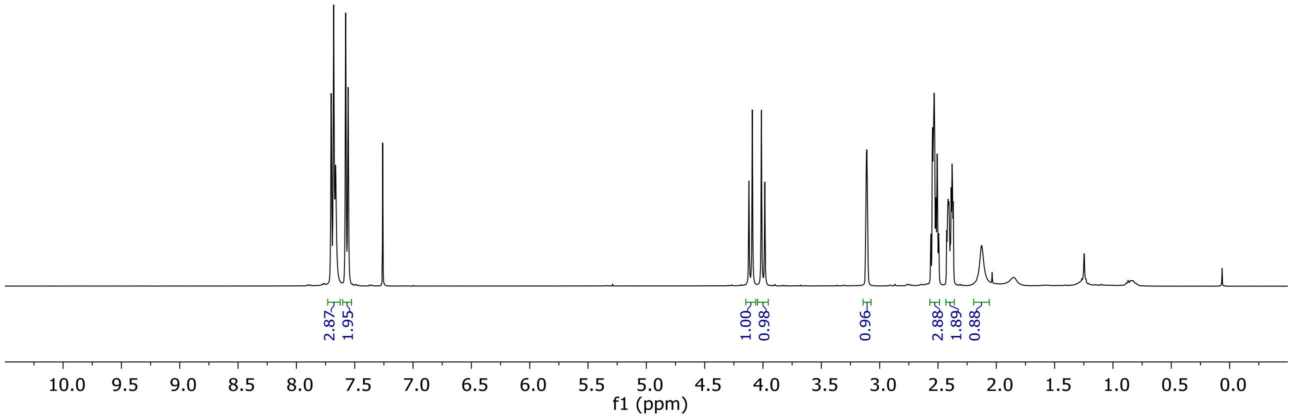
2.87
1.95
1.00
0.98
0.96
2.88
1.89
0.88
10.0 9.5 9.0 8.5 8.0 7.5 7.0 6.5 6.0 5.5 5.0 4.5 4.0 3.5 3.0 2.5 2.0 1.5 1.0 0.5 0.0
f1 (ppm)

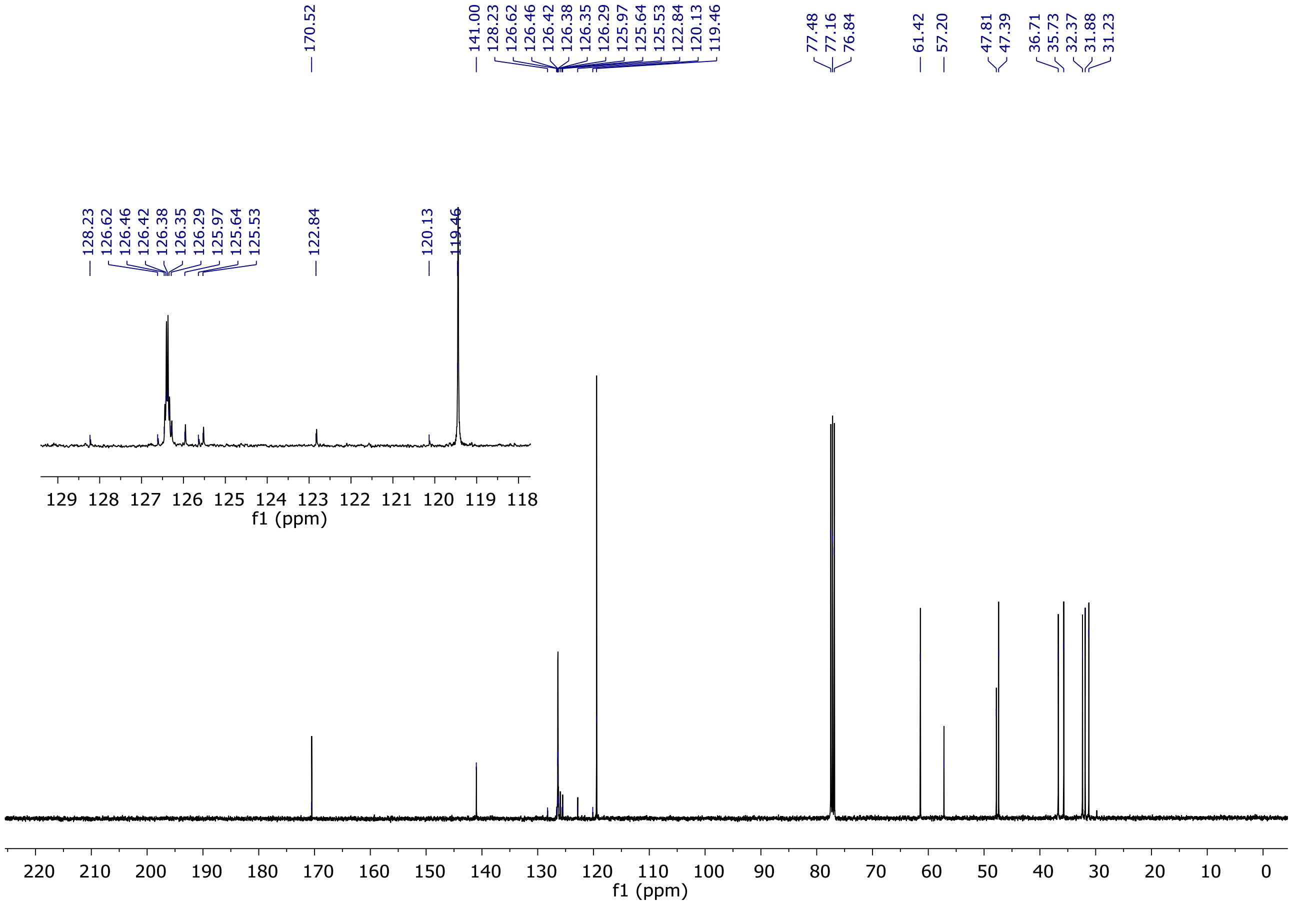
170.52
141.00
128.23
126.62
126.46
126.42
126.38
126.35
126.29
125.97
125.64
125.53
122.84
120.13
119.46
77.48
77.16
76.84
61.42
57.20
47.81
47.39
36.71
35.73
32.37
31.88
31.23
128.23
126.62
126.46
126.42
126.38
126.35
126.29
125.97
125.64
125.53
122.84
120.13
119.46
129 128 127 126 125 124 123 122 121 120 119 118
f1 (ppm)
220 210 200 190 180 170 160 150 140 130 120 110 100 90 80 70 60 50 40 30 20 10 0
f1 (ppm)

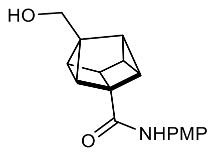
HO
O
NHPMP

3g

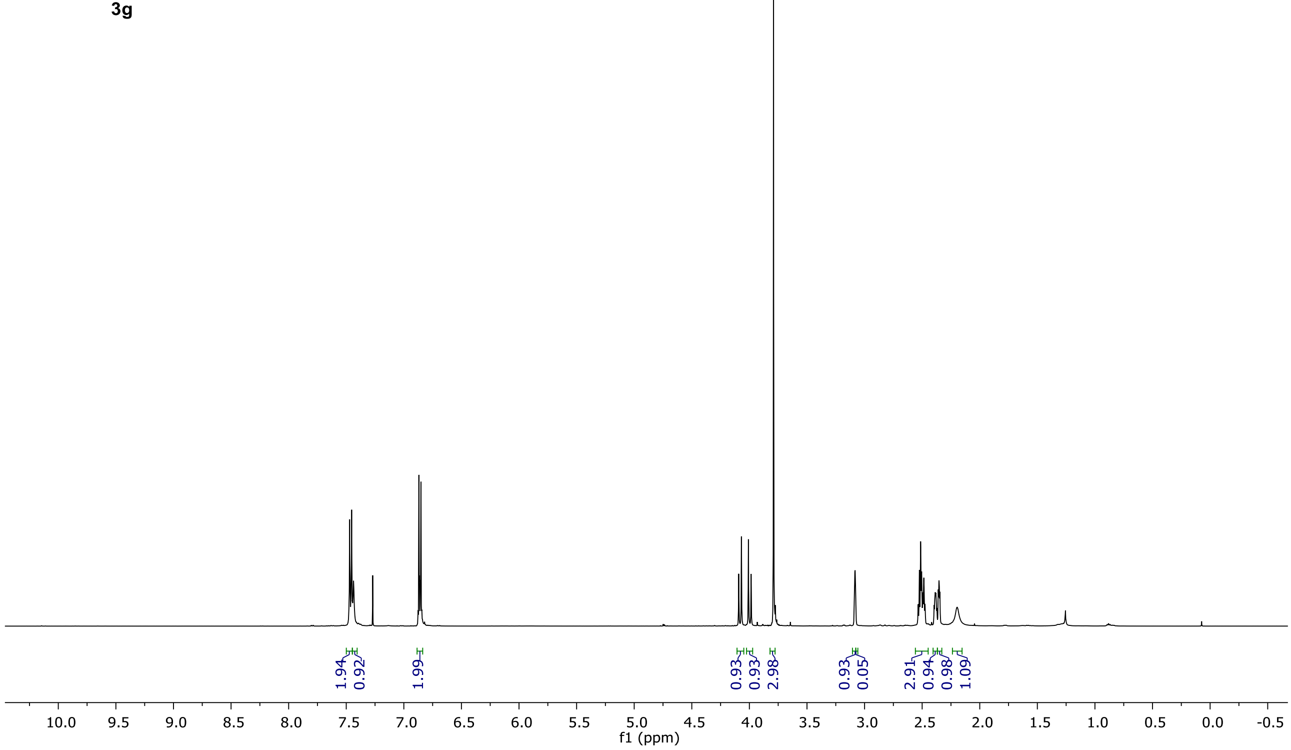
1.94
0.92
1.99
0.93
0.93
2.98
0.93
0.05
2.91
0.94
0.98
1.09
10.0 9.5 9.0 8.5 8.0 7.5 7.0 6.5 6.0 5.5 5.0 4.5 4.0 3.5 3.0 2.5 2.0 1.5 1.0 0.5 0.0 -0.5
f1 (ppm)

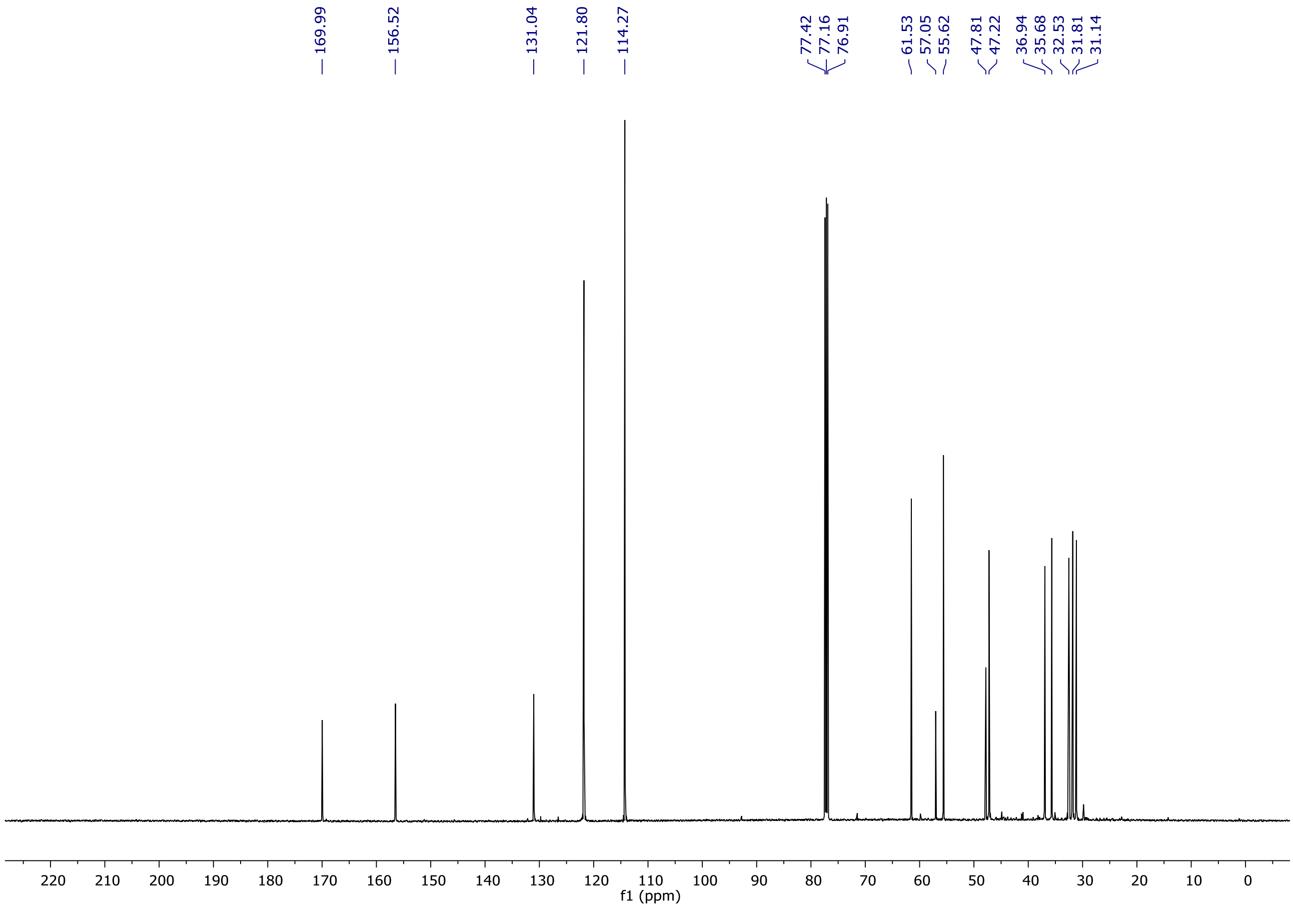
169.99
156.52
131.04
121.80
114.27
77.42
77.16
76.91
61.53
57.05
55.62
47.81
47.22
36.94
35.68
32.53
31.81
31.14
220 210 200 190 180 170 160 150 140 130 120 110 100 90 80 70 60 50 40 30 20 10 0
f1 (ppm)

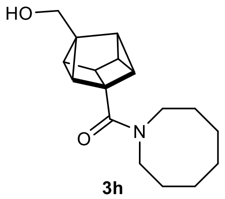
HO
O
N

3h

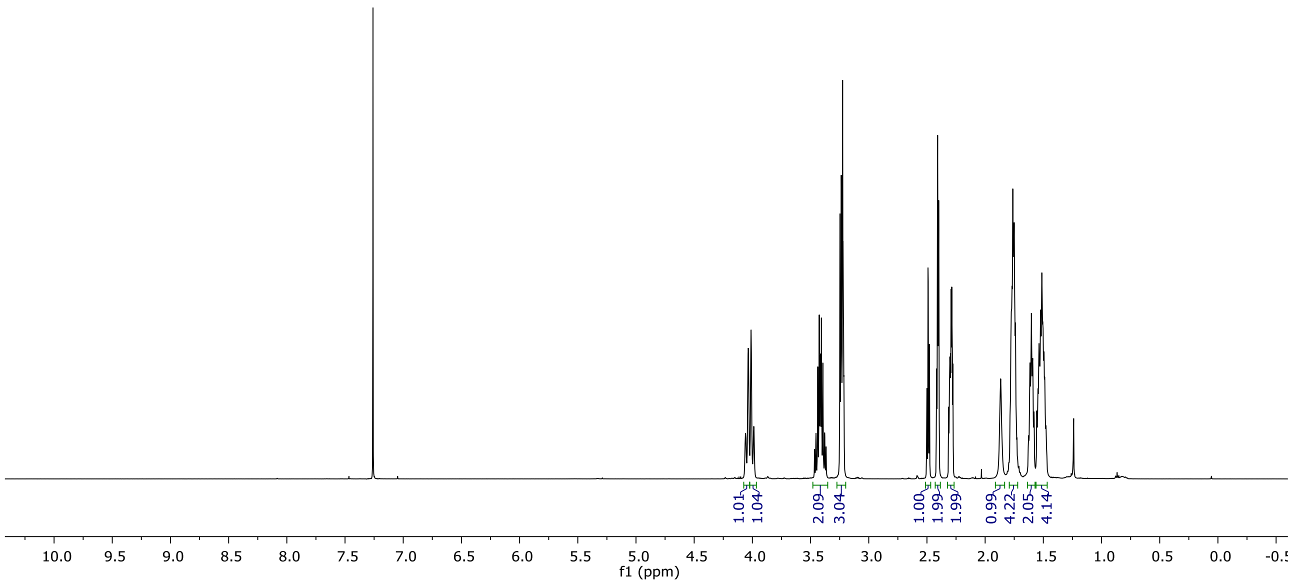
1.01
1.04
2.09
3.04
1.00
1.99
1.99
0.99
4.22
2.05
4.14
10.0 9.5 9.0 8.5 8.0 7.5 7.0 6.5 6.0 5.5 5.0 4.5 4.0 3.5 3.0 2.5 2.0 1.5 1.0 0.5 0.0
f1 (ppm)

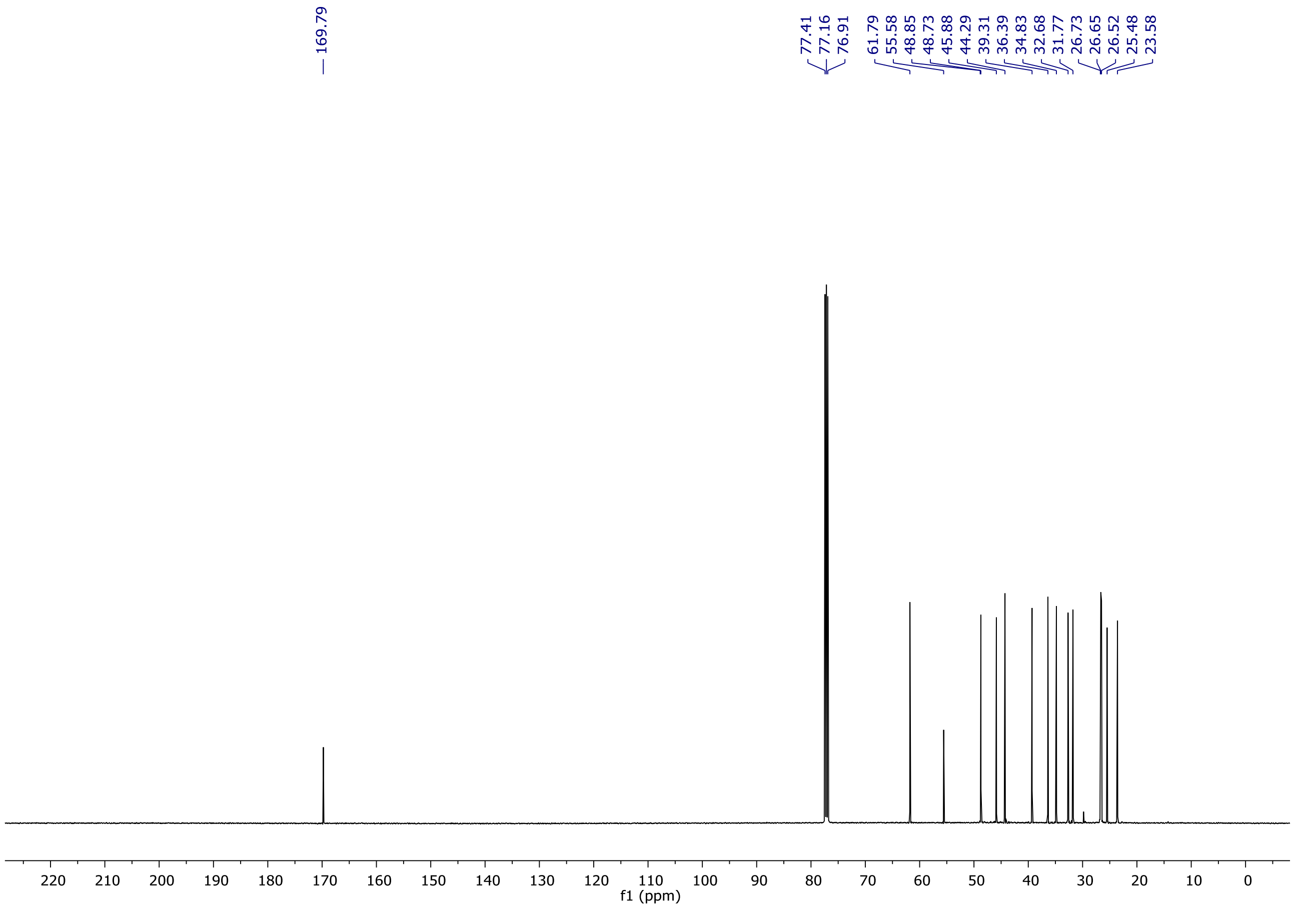
169.79
77.41
77.16
76.91
61.79
55.58
48.85
48.73
45.88
44.29
39.31
36.39
34.83
32.68
31.77
26.73
26.65
26.52
25.48
23.58
220 210 200 190 180 170 160 150 140 130 120 110 100 90 80 70 60 50 40 30 20 10 0
f1 (ppm)

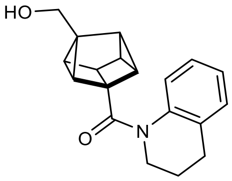
HO
O
N

3i

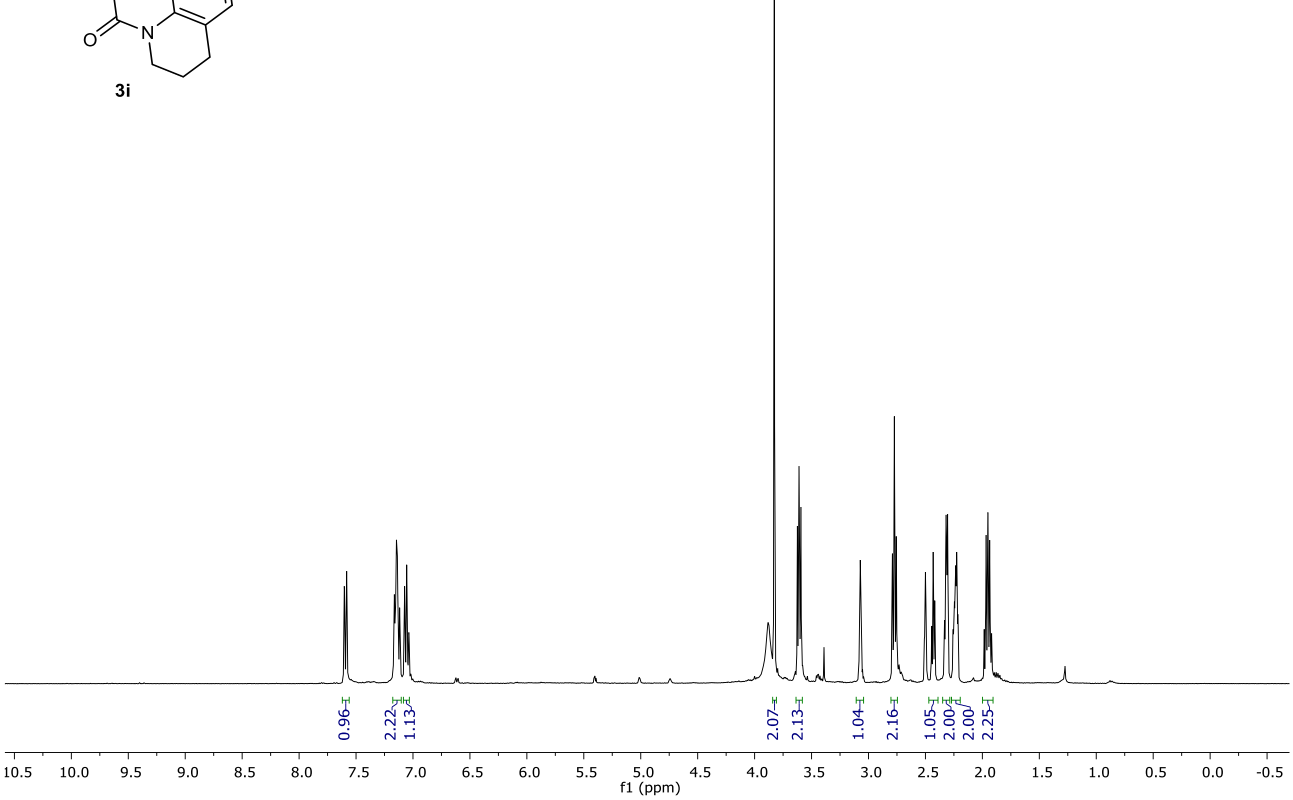
0.96
2.22
1.13
2.07
2.13
1.04
2.16
1.05
2.00
2.00
2.25
10.5 10.0 9.5 9.0 8.5 8.0 7.5 7.0 6.5 6.0 5.5 5.0 4.5 4.0 3.5 3.0 2.5 2.0 1.5 1.0 0.5 0.0 -0.5
f1 (ppm)

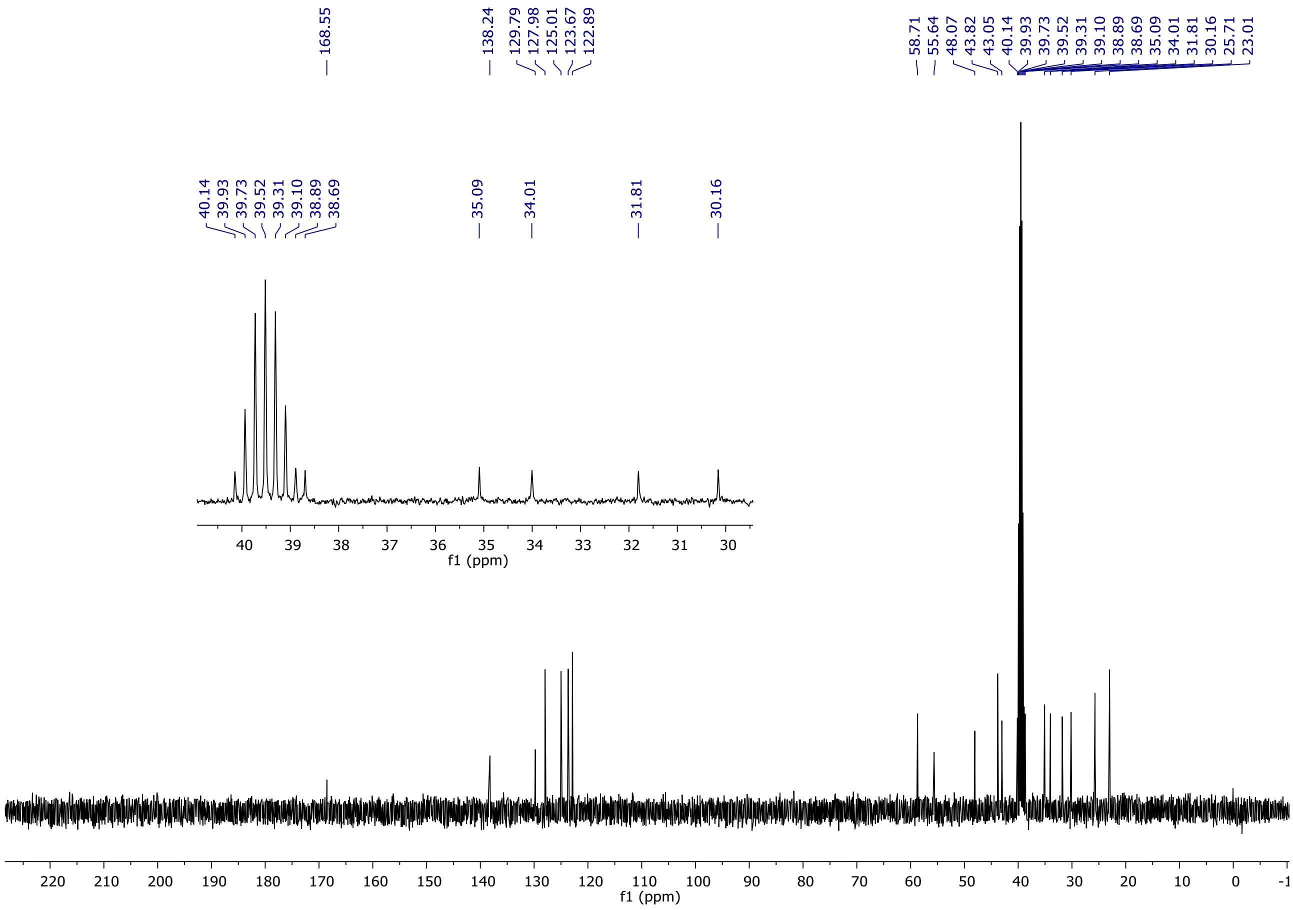
168.55
138.24
129.79
127.98
125.01
123.67
122.89
58.71
55.64
48.07
43.82
43.05
40.14
39.93
39.73
39.52
39.31
39.10
38.89
38.69
35.09
34.01
31.81
30.16
25.71
23.01
40.14
39.93
39.73
39.52
39.31
39.10
38.89
38.69
35.09
34.01
31.81
30.16
40 39 38 37 36 35 34 33 32 31 30
f1 (ppm)
220 210 200 190 180 170 160 150 140 130 120 110 100 90 80 70 60 50 40 30 20 10 0 -1
f1 (ppm)

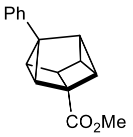
Ph
CO2Me

3j

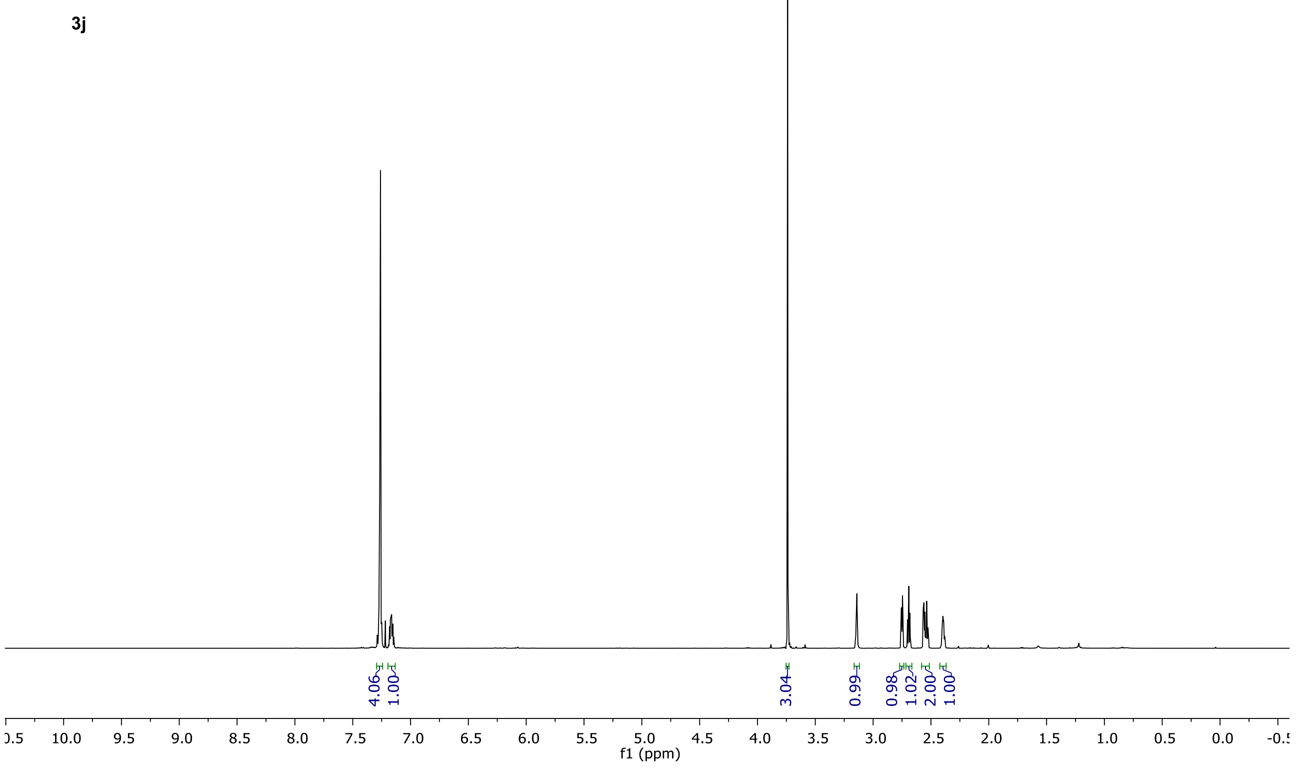
4.06
1.00
3.04
0.99
0.98
1.02
2.00
1.00
f1 (ppm)

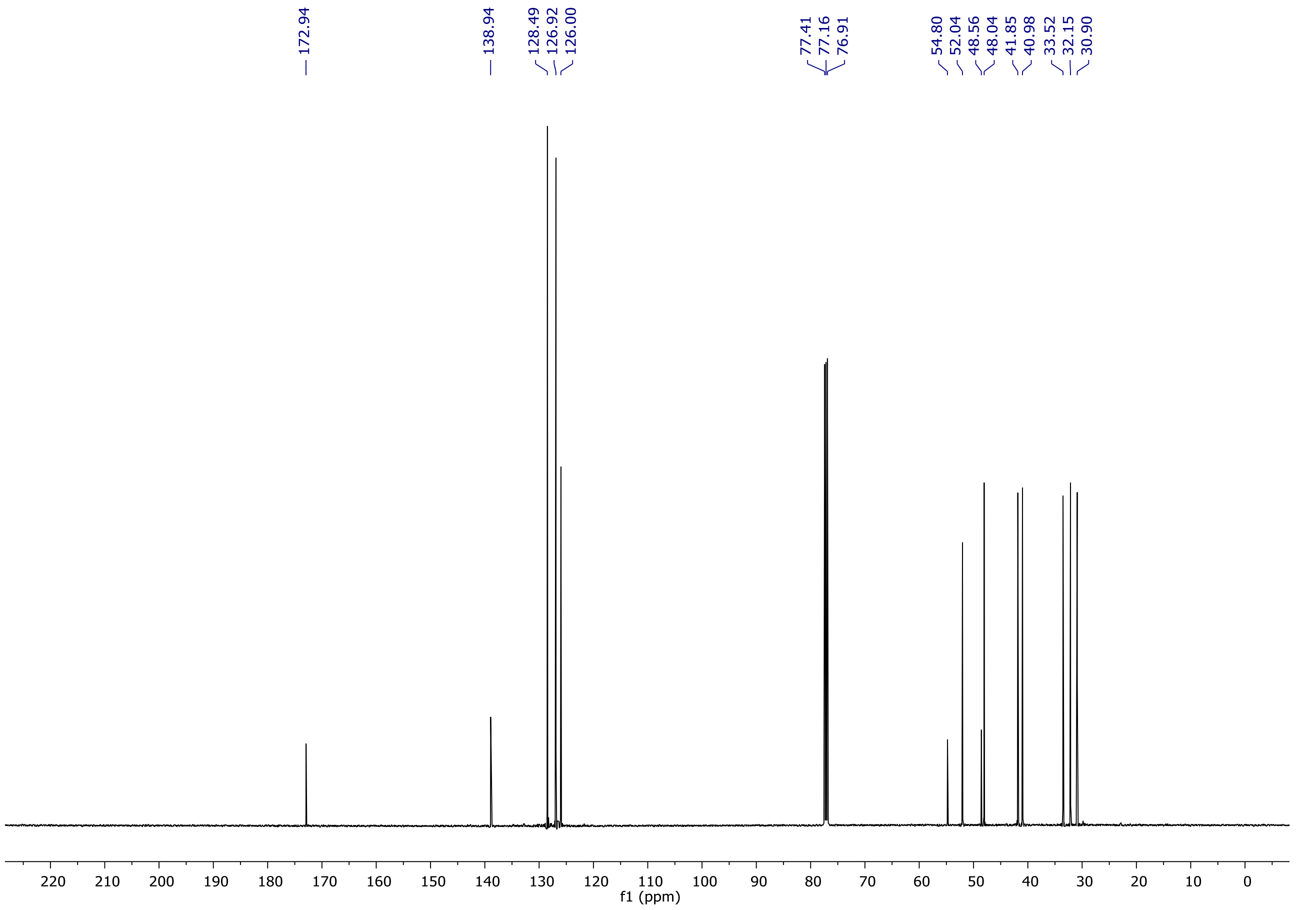
172.94
138.94
128.49
126.92
126.00
77.41
77.16
76.91
54.80
52.04
48.56
48.04
41.85
40.98
33.52
32.15
30.90
f1 (ppm)

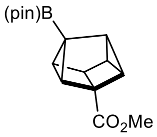

(pin)B
CO2Me

3k

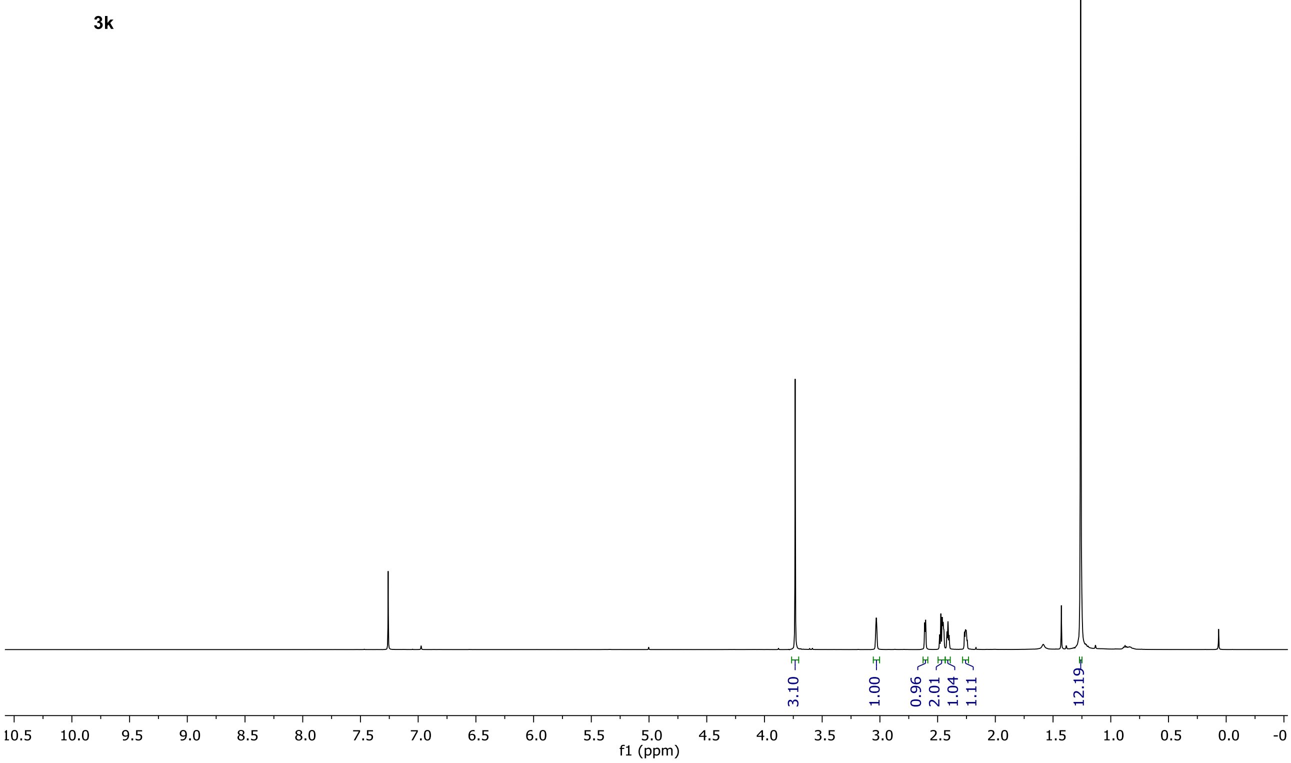

3.10
1.00
0.96
2.01
1.04
1.11
12.19
f1 (ppm)

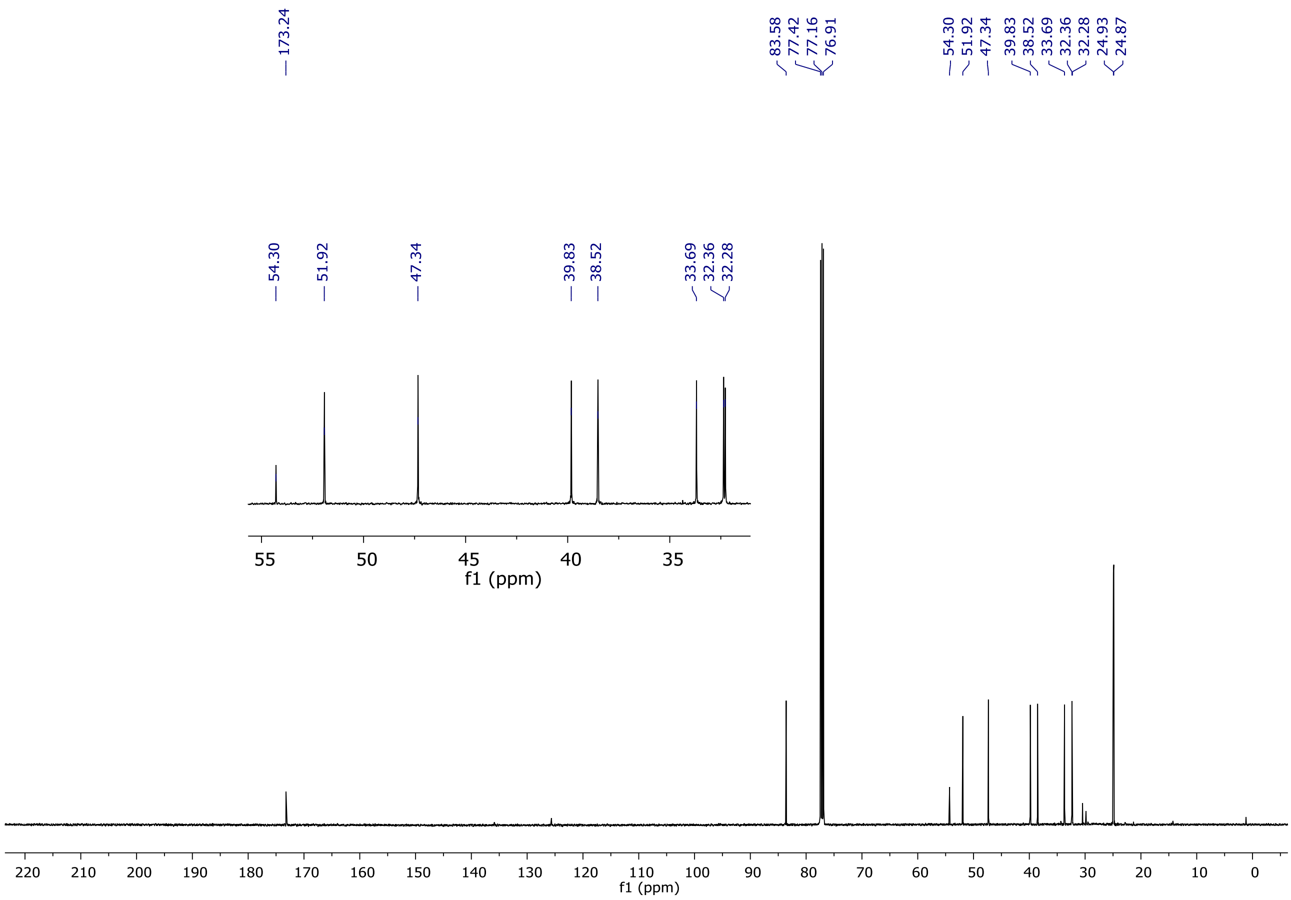

173.24
83.58
77.42
77.16
76.91
54.30
51.92
47.34
39.83
38.52
33.69
32.36
32.28
24.93
24.87
f1 (ppm)

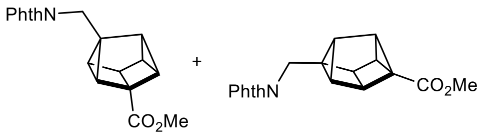

3l (3:1 mixture) **2l**

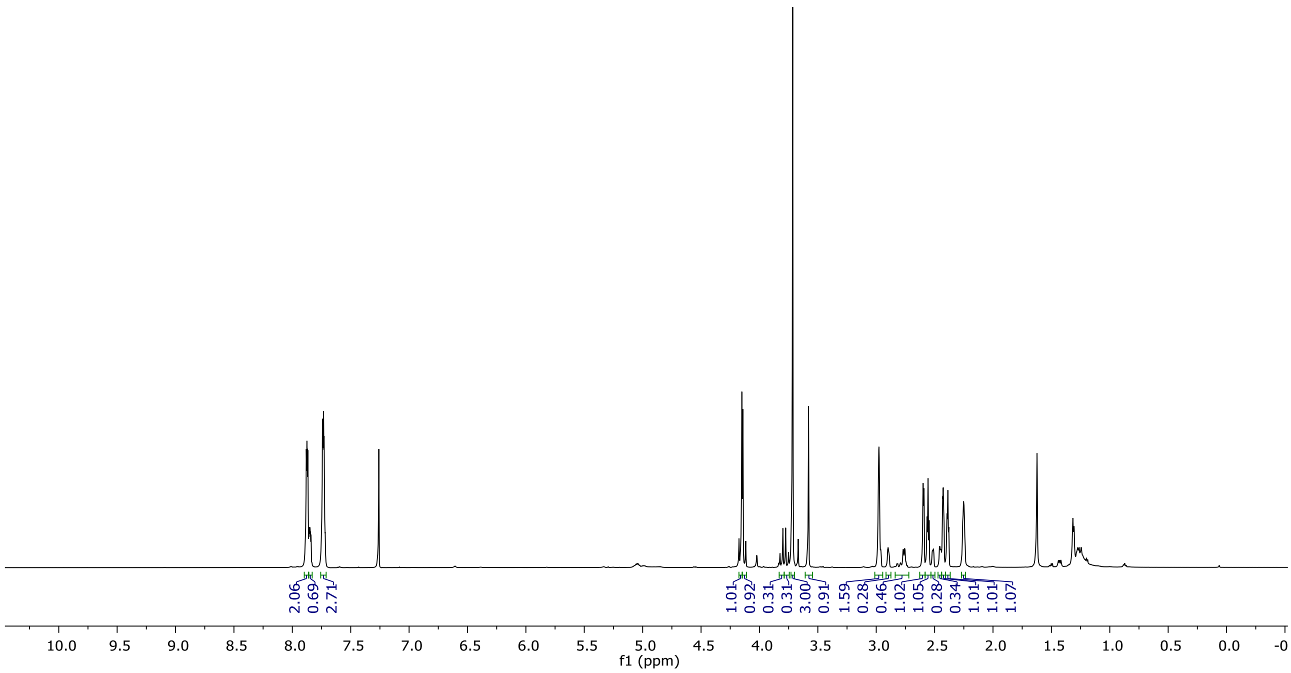

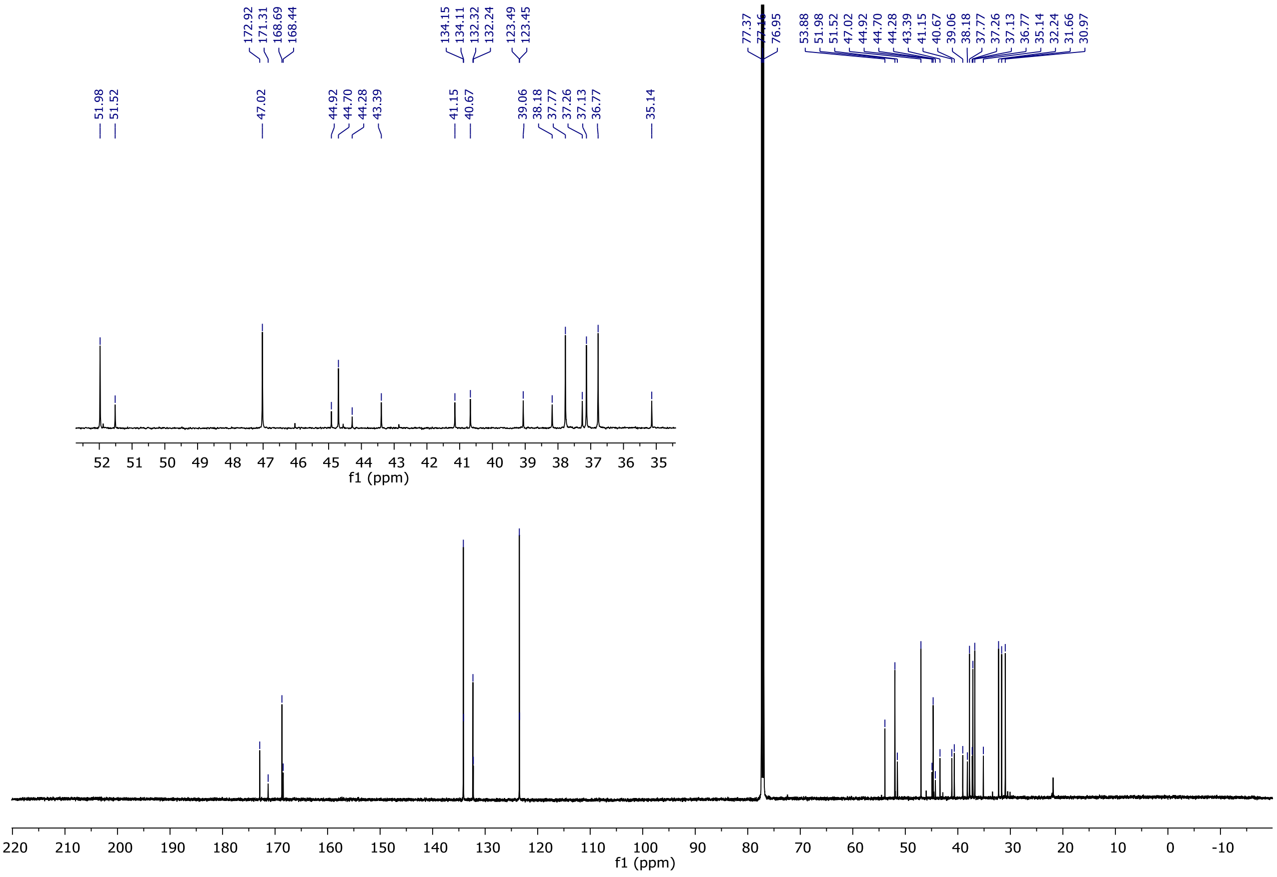

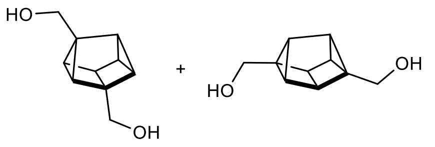

3m (19:1 mixture) **2m**

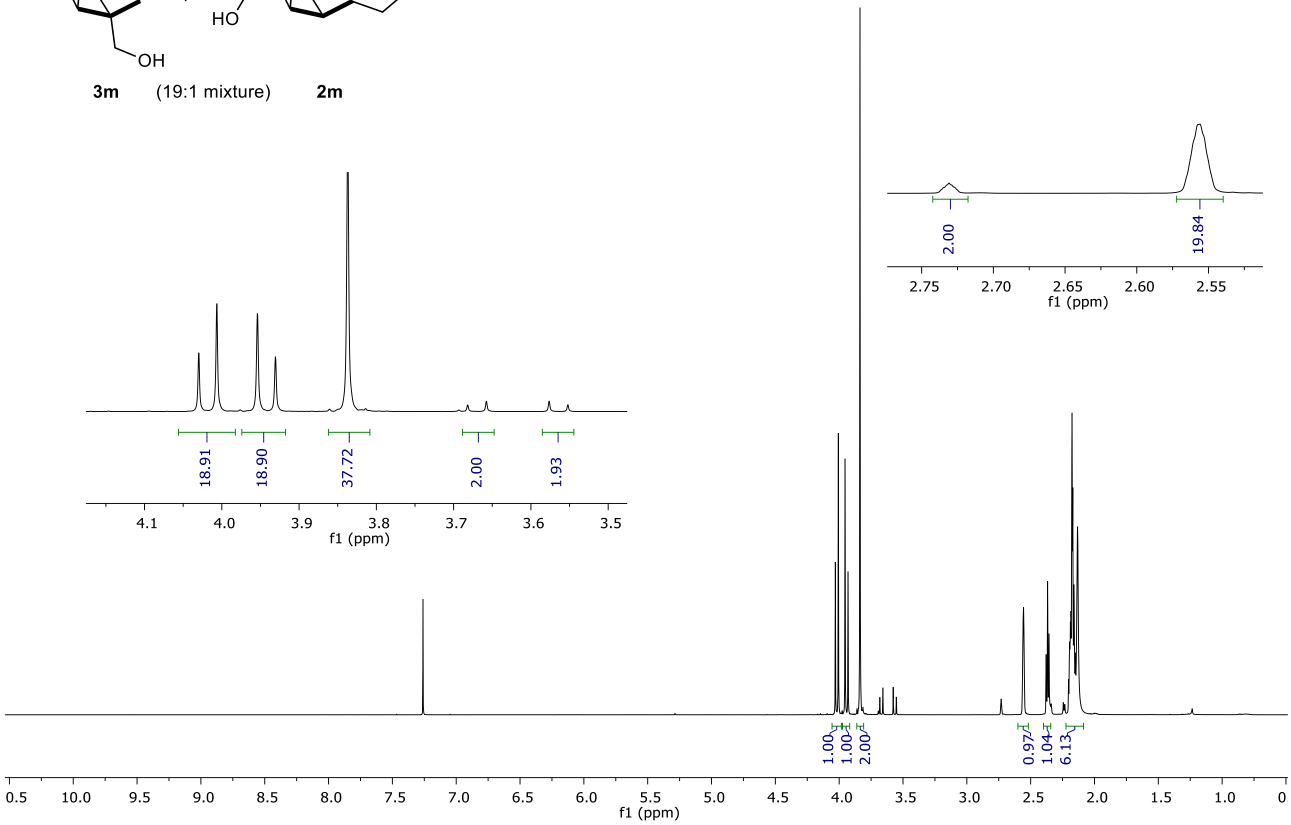

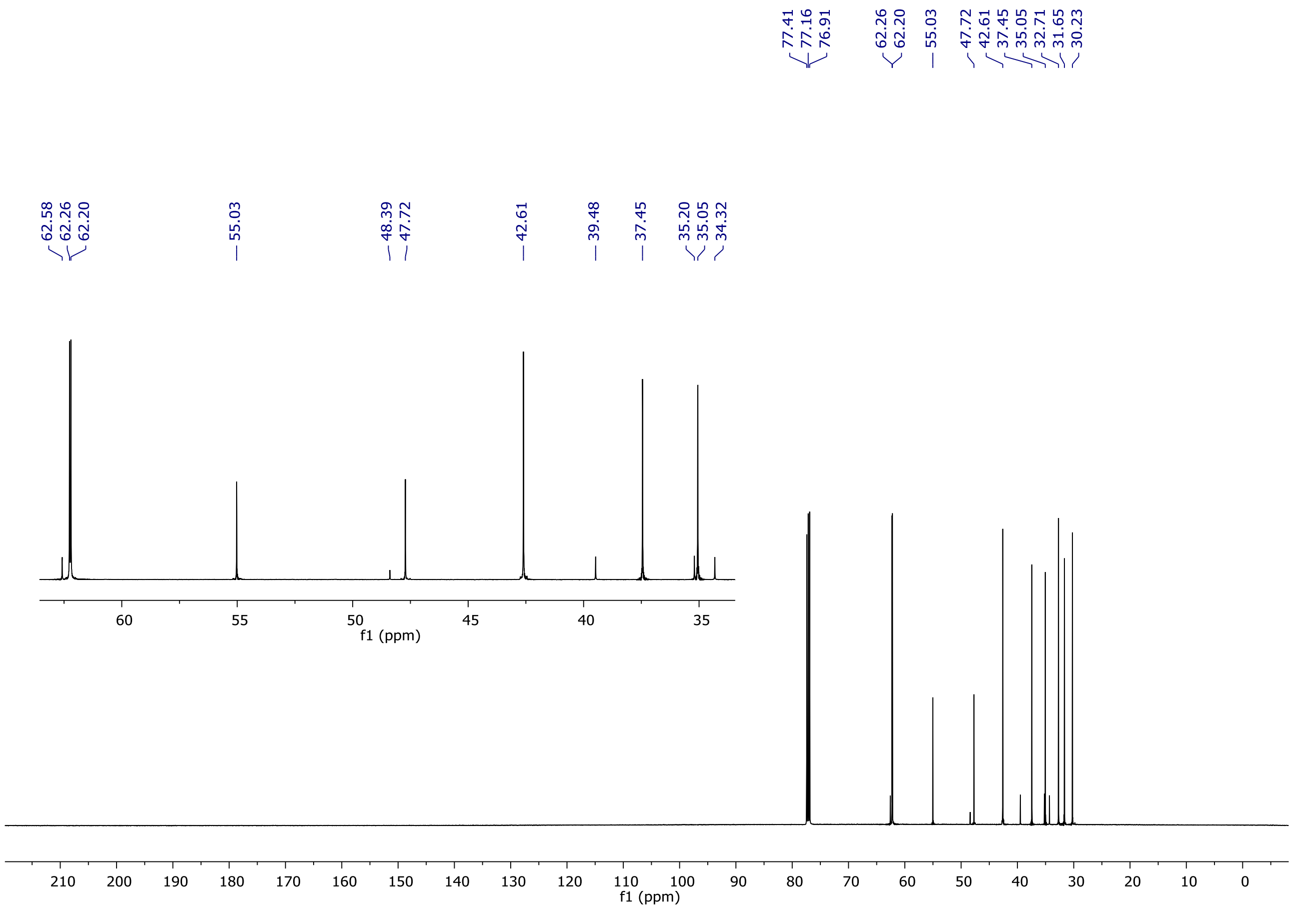

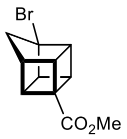
Br
CO2Me

6

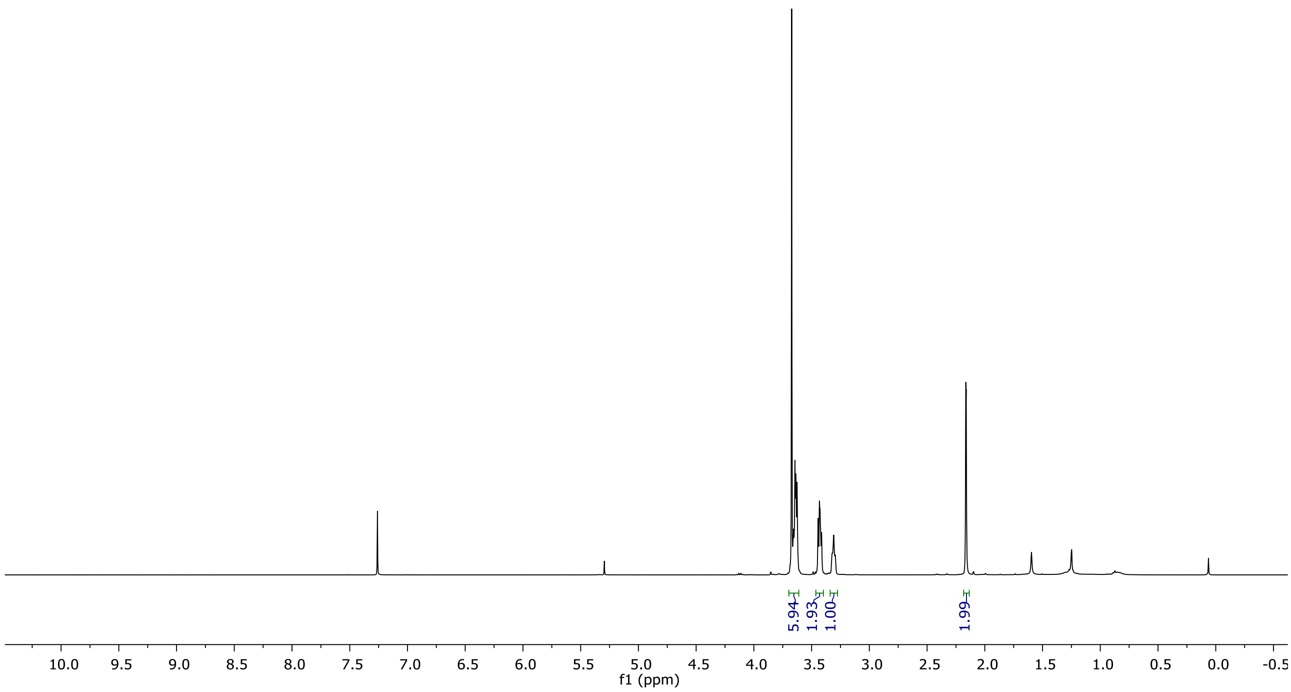
5.94
1.93
1.00
1.99
10.0 9.5 9.0 8.5 8.0 7.5 7.0 6.5 6.0 5.5 5.0 4.5 4.0 3.5 3.0 2.5 2.0 1.5 1.0 0.5 0.0
f1 (ppm)

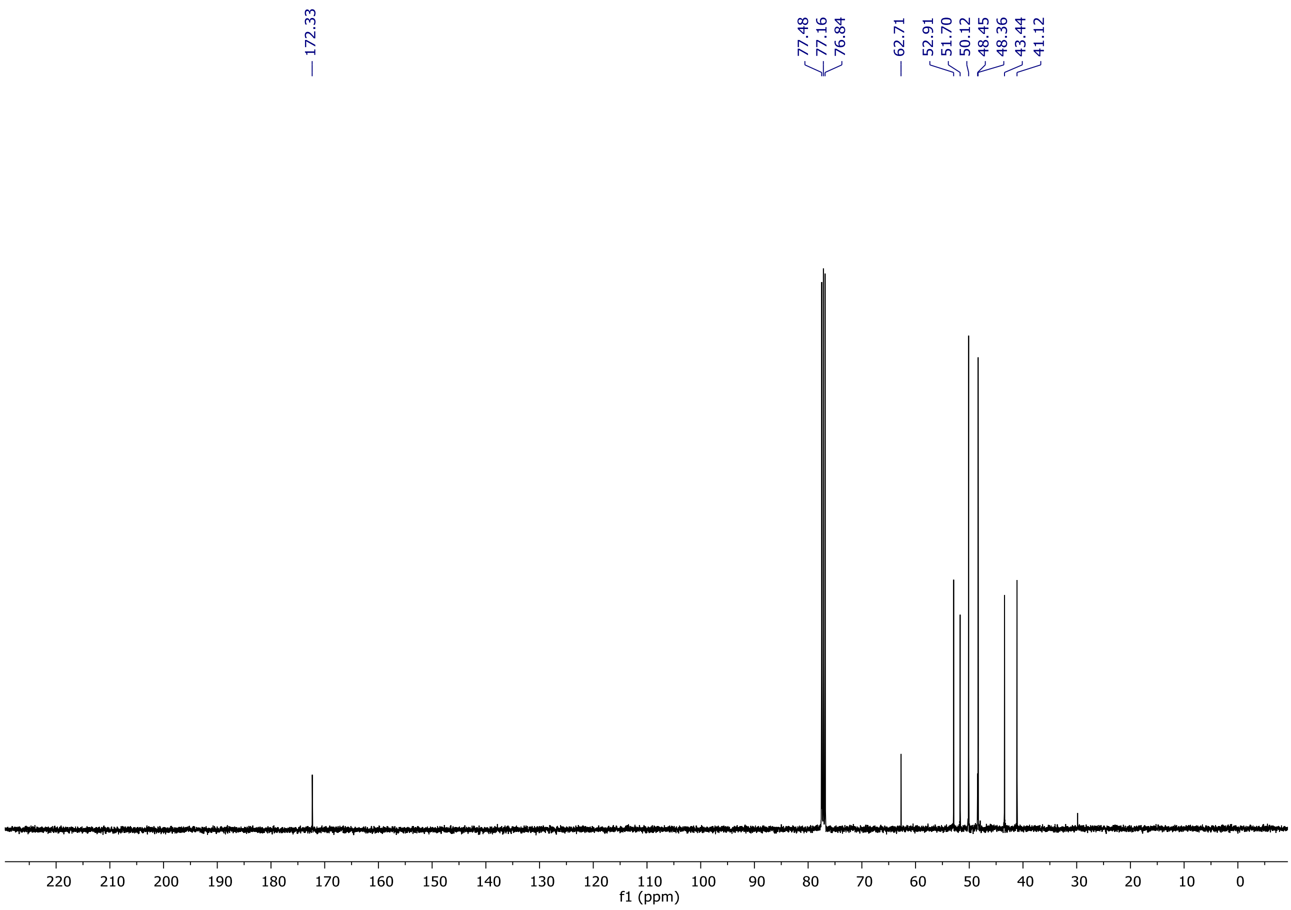
172.33
77.48
77.16
76.84
62.71
52.91
51.70
50.12
48.45
48.36
43.44
41.12
220 210 200 190 180 170 160 150 140 130 120 110 100 90 80 70 60 50 40 30 20 10 0
f1 (ppm)

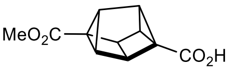
MeO$_2$C
CO$_2$H

14

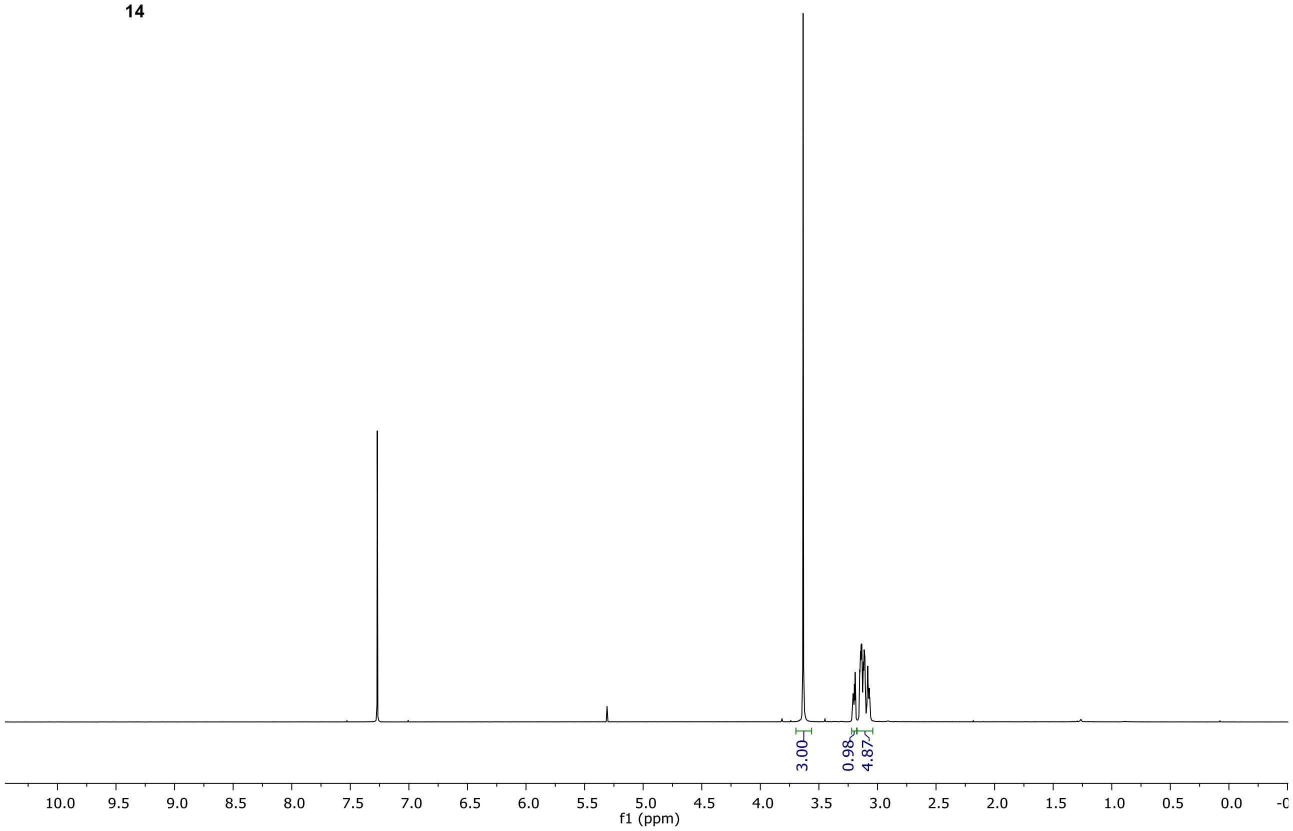
3.00
0.98
4.87
10.0 9.5 9.0 8.5 8.0 7.5 7.0 6.5 6.0 5.5 5.0 4.5 4.0 3.5 3.0 2.5 2.0 1.5 1.0 0.5 0.0
f1 (ppm)

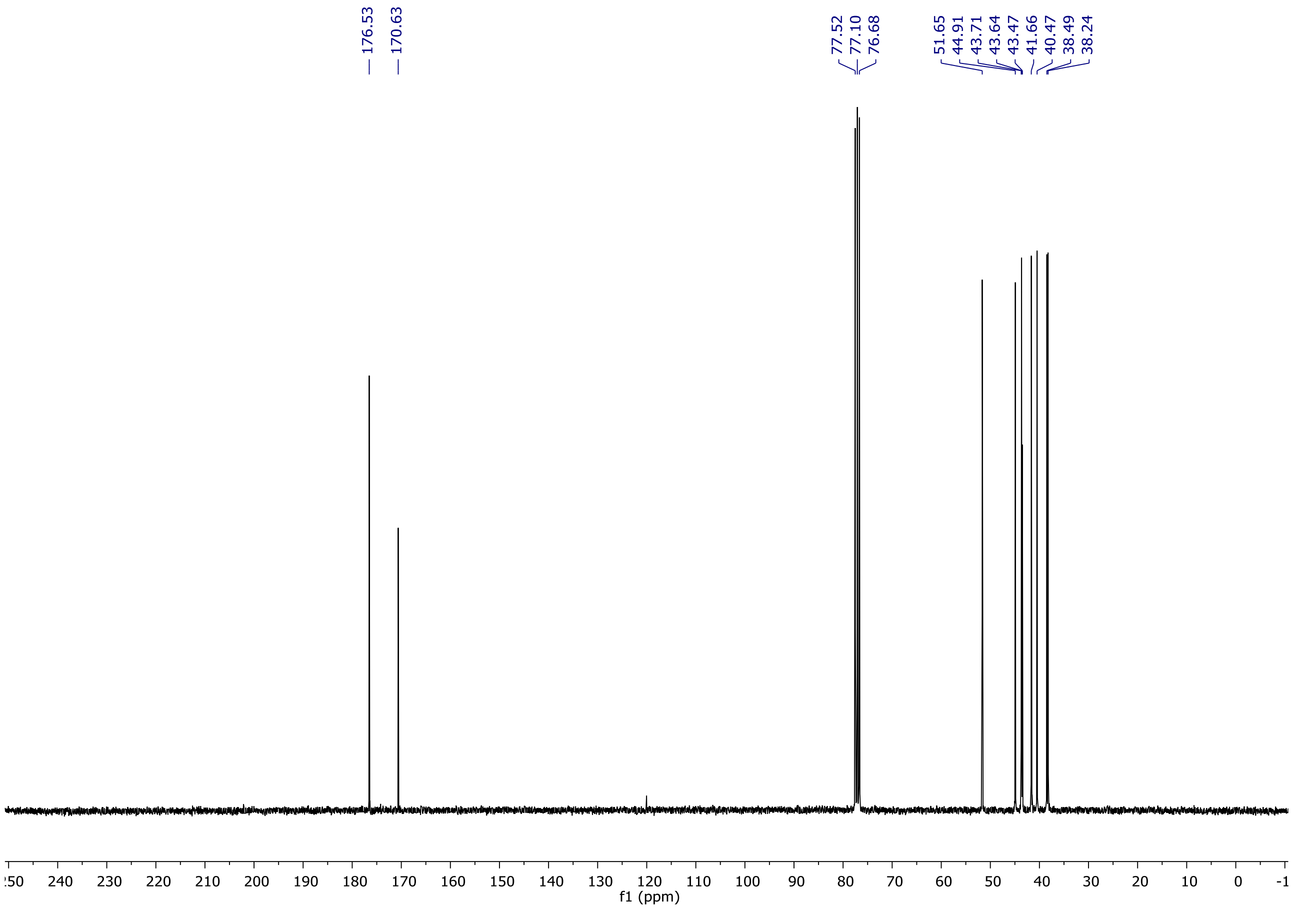
176.53
170.63
77.52
77.10
76.68
51.65
44.91
43.71
43.64
43.47
41.66
40.47
38.49
38.24
240 230 220 210 200 190 180 170 160 150 140 130 120 110 100 90 80 70 60 50 40 30 20 10 0
f1 (ppm)

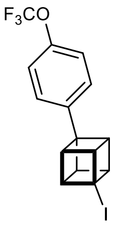
F3CO
I

1z

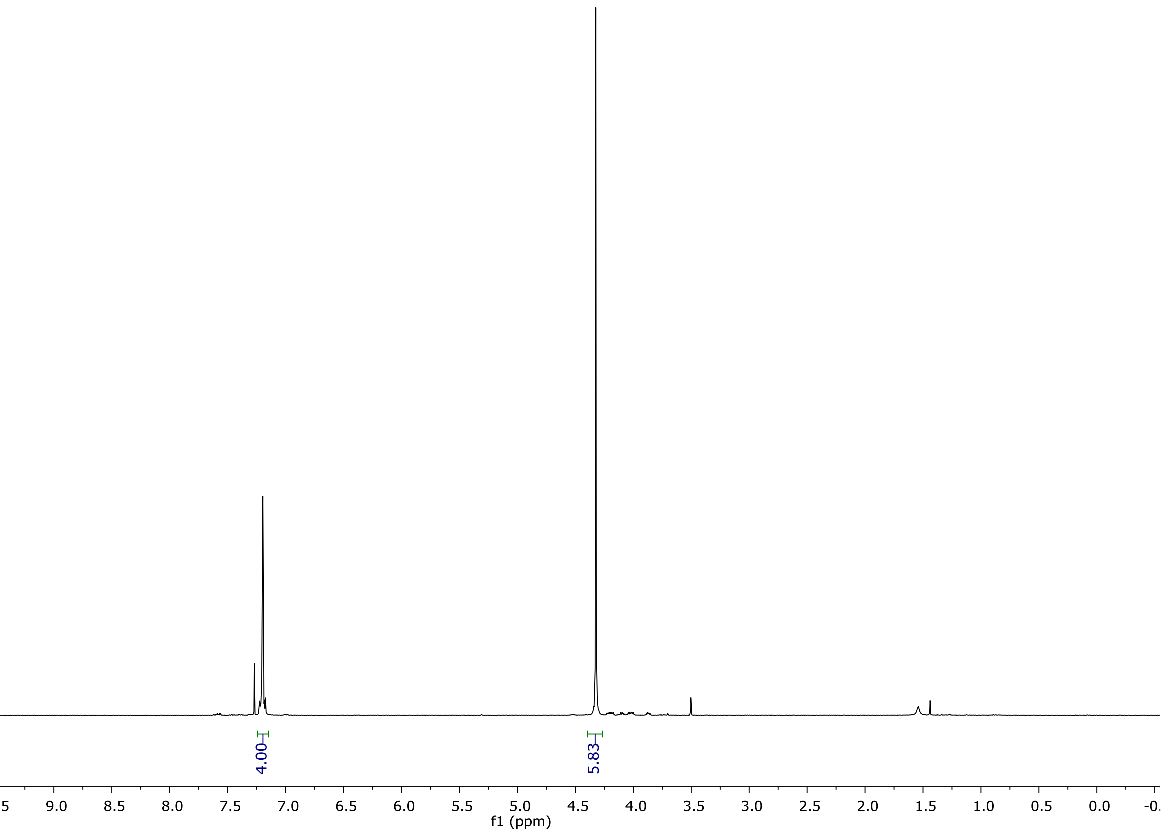
4.00
5.83
f1 (ppm)

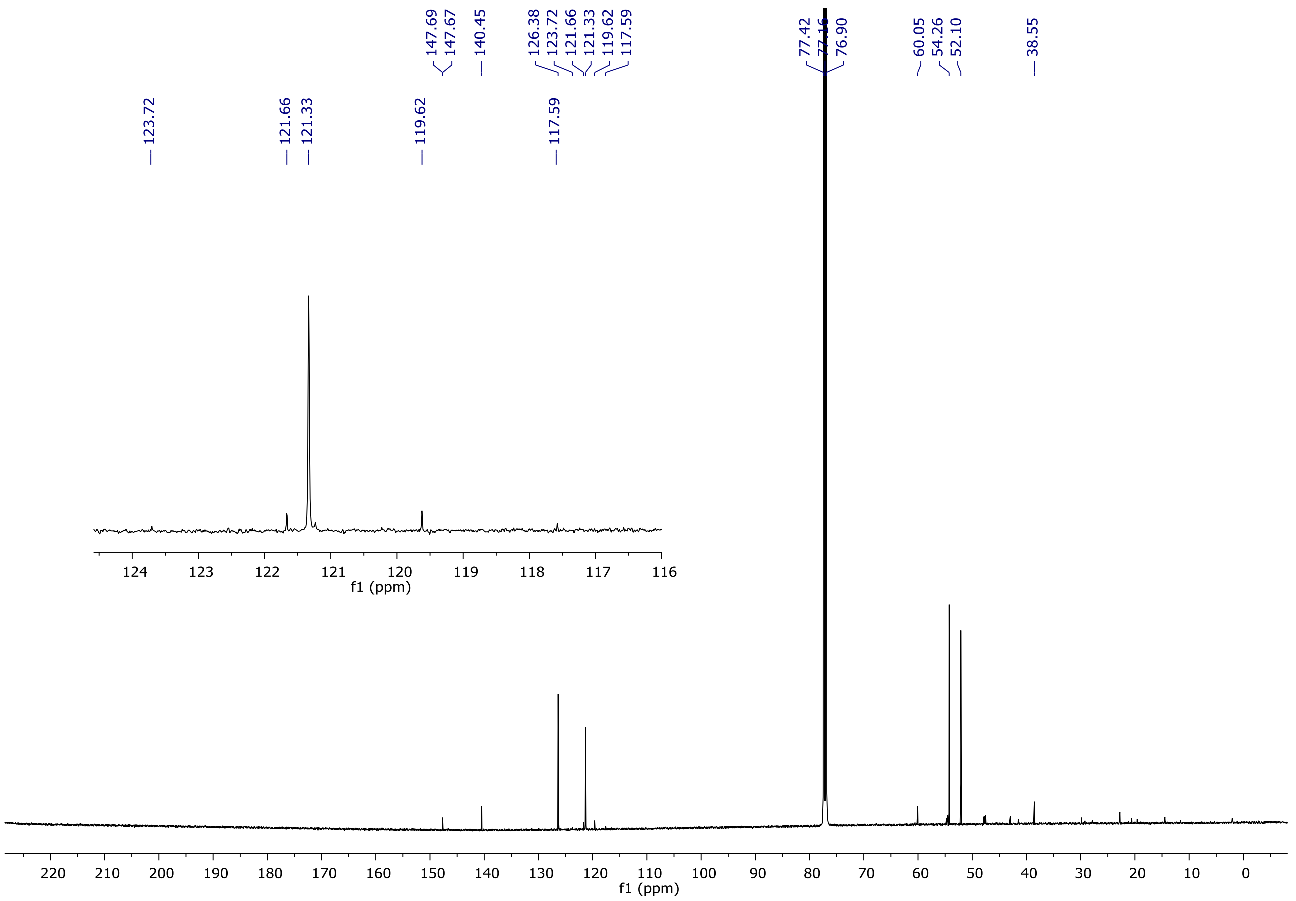
147.69
147.67
140.45
126.38
123.72
121.66
121.33
119.62
117.59
77.42
76.90
60.05
54.26
52.10
38.55
123.72
121.66
121.33
119.62
117.59
f1 (ppm)
f1 (ppm)

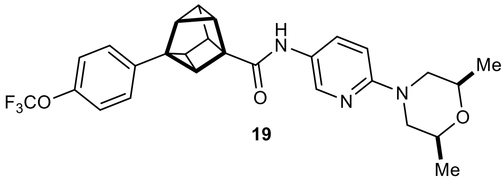
F3CO
H
N
O
N
N
O
Me
Me

19

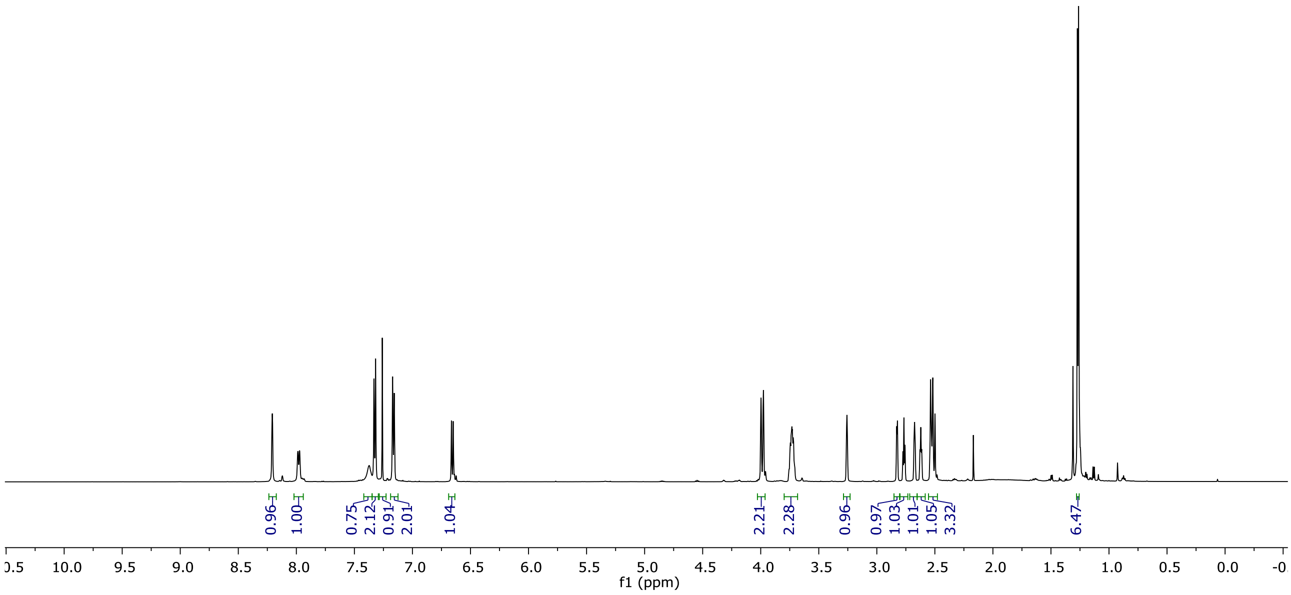
0.96
1.00
0.75
2.12
0.91
2.01
1.04
2.21
2.28
0.96
0.97
1.03
1.01
1.05
3.32
6.47
f1 (ppm)

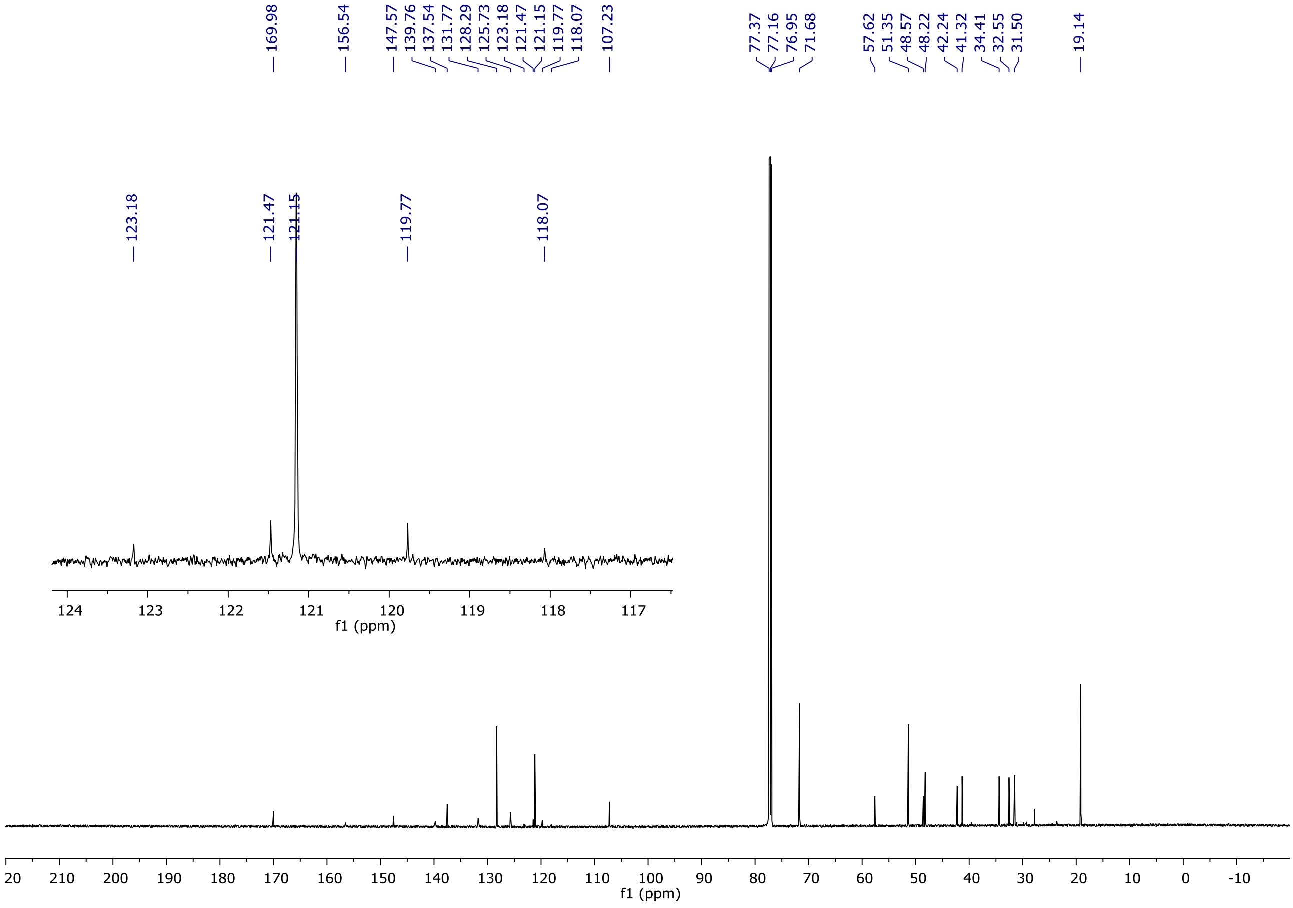
169.98
156.54
147.57
139.76
137.54
131.77
128.29
125.73
123.18
121.47
121.15
119.77
118.07
107.23
77.37
77.16
76.95
71.68
57.62
51.35
48.57
48.22
42.24
41.32
34.41
32.55
31.50
19.14
123.18
121.47
121.15
119.77
118.07
f1 (ppm)
f1 (ppm)

9. References

1. J. Cosier, A. M. Glazer, *J. Appl. Crystallogr.* **1986**, *19*, 105-107.
2. Rigaku Oxford Diffraction, (2018), CrysAlisPro Software system, version 1.171.40.45a, Rigaku Corporation, Oxford, UK.
3. O. V. Dolomanov, L. J. Bourhis, R. J. Gildea, J. A. K. Howard, H. Puschmann, *J. Appl. Crystallogr.* **2009**, *42*, 339-341.
4. G. M. Sheldrick, *Acta Crystallogr. A* **2015**, *71*, 3-8.
5. G. M. Sheldrick, *Acta Crystallogr. C* **2015**, *71*, 3-8.
6. D. R. Allan, H. Nowell, S. A. Barnett, M. R. Warren, A. Wilcox, J. Christensen, L. K. Saunders, A. Peach, M. T. Hooper, L. Zaja, S. Patel, L. Cahill, R. Marshall, S. Trimnell, A. J. Foster, T. Bates, S. Lay, M. A. Williams, P. V. Hathaway, G. Winter, M. Gerstel, R. W. Wooley, *Crystals* **2017**, *7*, 336.
7. G. Winter, D. G. Waterman, J. M. Parkhurst, A. S. Brewster, R. J. Gildea, M. Gerstel, L. Fuentes-Montero, M. Vollmar, T. Michels-Clark, I. D. Young, N. K. Sauter, G. Evans, *Acta. Crystallogr. D Struct. Biol* **2018**, *74*, 85-97.
8. P. R. Evans, G. N. Murshudov, *Acta Crystallogr. D* **2013**, *69*, 1204-1214.
9. M. Bliese, J. Tsanaktsidis, *Aust. J. Chem.* **1997**, *50*, 189-192.
10. J. Wlochal, R. D. M. Davies, J. Burton, *Org. Lett.* **2014**, *16*, 4094-4097.
11. L. T. Eremenko, L. B. Romanova, M. E. Ivanova, A. V. Shastin, I. L. Eremenko, S. E. Nefedov, *Russ. Chem. Bull.* **1998**, *47*, 441-446.
12. S. D. Houston, H. Xing, P. V. Bernhardt, T. J. Vanden Berg, J. Tsanaktsidis, G. P. Savage, C. M. Williams, *Chem. Eur. J.* **2019**, *25*, 2735-2739.
13. H. Takebe, S. Matsubara, *Eur. J. Org. Chem.* **2022**, e202200567.
14. C. Li, J. Wang, L. M. Barton, S. Yu, M. Q. Tian, D. S. Peters, M. Kumar, A. W. Yu, K. A. Johnson, A. K. Chatterjee, M. Yan, P. S. Baran, *Science* **2017**, *356*, eaam7355.
15. P. E. Eaton, Y. C. Yip, *J. Am. Chem. Soc.* **1991**, *113*, 7692-7697.
16. Prepared as described in: (a) P. E. Eaton, K. Pramod, T. Emrick, R. Gilardi, *J. Am. Chem. Soc.* **1999**, *121*, 4111-4123. The analytical data are reported in: (b) E. Honegger, E. Heilbronner, T. Urbanek, H.-D. Martin, *Helv. Chim. Acta* **1985**, *68*, 23-38.
17. Y. Zhang, X. Ge, H. Lu, G. Li, *Angew. Chem., Int. Ed.* **2021**, *60*, 1845-1852.
18. R. Al Hussainy, J. Verbeek, D. van der Born, J. Booij, J. D. M. Herscheid, *Eur. J. Med. Chem.* **2011**, *46*, 5728-5735.
19. A. J. H. Klunder, B. Zwanenburg, *Tetrahedron* **1973**, *29*, 161-166.
20. (a) M. A. Dallaston, S. D. Houston, C. M. Williams, *Chem. Eur. J.* **2020**, *26*, 11966-11970. For another study of the thermal and impact stability of cubanes, see: (b) M. A. Dallaston, J. S. Brusnahan, C. Wall, C. M. Williams, *Chem. Eur. J.* **2019**, *25*, 8344-8352.
21. Prepared as described in: D. Bhattarai, J. H. Jung, S. Han, H. Lee, S. J. Oh, H. W. Ko, K. Lee, *Eur. J. Med. Chem.* **2017**, *125*, 1036-1050.
22. https://pion-inc.com/resources/resource-library/multi-lab-intrinsic-solubility-measurement-reproducibility-in-cheqsol-and-shake-flask-methods
